# MMTV RNA packaging requires an extended long-range interaction for productive Gag binding to packaging signals

**DOI:** 10.1371/journal.pbio.3002827

**Published:** 2024-10-03

**Authors:** Suresha G. Prabhu, Vineeta N. Pillai, Lizna Mohamed Ali, Valérie Vivet-Boudou, Akhil Chameettachal, Serena Bernacchi, Farah Mustafa, Roland Marquet, Tahir A. Rizvi

**Affiliations:** 1 Department of Microbiology & Immunology, College of Medicine and Health Sciences (CMHS), United Arab Emirates University (UAEU), Al Ain, United Arab Emirates; 2 Université de Strasbourg, CNRS, Architecture et Réactivité de l’ARN, UPR 9002, Strasbourg, France; 3 Department of Biochemistry & Molecular Biology, College of Medicine and Health Sciences (CMHS), United Arab Emirates University (UAEU), Al Ain, United Arab Emirates; 4 Zayed bin Sultan Center for Health Sciences, United Arab Emirates University, Al Ain, United Arab Emirates; 5 ASPIRE Research Institute in Precision Medicine, Abu Dhabi, United Arab Emirates; Ulm University Medical Center, GERMANY

## Abstract

The packaging of genomic RNA (gRNA) into retroviral particles relies on the specific recognition by the Gag precursor of packaging signals (*Psi*), which maintain a complex secondary structure through long-range interactions (LRIs). However, it remains unclear whether the binding of Gag to *Psi* alone is enough to promote RNA packaging and what role LRIs play in this process. Using mouse mammary tumor virus (MMTV), we investigated the effects of mutations in 4 proposed LRIs on gRNA structure and function. Our findings revealed the presence of an unsuspected extended LRI, and hSHAPE revealed that maintaining a wild-type–like *Psi* structure is crucial for efficient packaging. Surprisingly, filter-binding assays demonstrated that most mutants, regardless of their packaging capability, exhibited significant binding to Pr77^Gag^, suggesting that Gag binding to *Psi* is insufficient for efficient packaging. Footprinting experiments indicated that efficient RNA packaging is promoted when Pr77^Gag^ binds to 2 specific sites within *Psi*, whereas binding elsewhere in *Psi* does not lead to efficient packaging. Taken together, our results suggest that the 3D structure of the *Ps*i/Pr77^Gag^ complex regulates the assembly of viral particles around gRNA, enabling effective discrimination against other viral and cellular RNAs that may also bind Gag efficiently.

## Abstract 2

The role of long-range interactions (LRI) in retroviral RNA packaging is unclear. This study found that a specific packaging signal (Psi) structure, maintained by an extended LRI, is crucial for efficient RNA packaging, with Gag binding to key Psi sites essential for selectivity.

## Introduction

The vast majority of virus species selectively package their DNA or RNA genome into viral particles. While DNA viruses typically assemble a procapsid first, followed by packaging of the genome in an energy-dependent process, many RNA viruses, including significant human pathogens such as retroviruses, influenza viruses, and coronaviruses, undergo concerted processes of genomic RNA (gRNA) packaging and viral assembly [1,2].

In retroviruses, the selective packaging of gRNA, which exists as a dimer of single-stranded RNA molecules noncovalently linked near their 5′-end [3,4], from the cellular environment where it represents only a small fraction (around 1%) of total mRNA, is facilitated by the recognition of *cis*-acting sequences known as packaging signal (*Psi;* ψ) typically located at the 5′ end of the retroviral genome, sometimes extending into the Gag open reading frame (ORF) [4–27]. While the nucleocapsid (NC) domain of Gag is crucial for selective packaging [12–17,21,22,28,29], in the case of human immunodeficiency virus (HIV-1), the matrix (MA) domain [30,31], capsid domain (CA; [32,33]), p6 domain [28], and spacer peptide sp1 [31,34] also participate in the specific recognition of *Psi* by Gag.

These retroviral *Psi* sequences adopt complex higher-order structures containing various structural motifs that are essential for Gag binding and retroviral RNA packaging [4,9,10,12,14–18,21,26,35–46]. Notably, *Psi* sequences in all retroviruses feature long-range interactions (LRIs) that maintain their overall secondary structure and, in several instances, are critical for Gag binding and gRNA packaging [3,9,10,26,36,43,45–55]. It is important to note that *Psi* sequences are significantly longer than typical protein-binding sites, and without a high-resolution 3D structure of the *Psi*/Gag complex, the precise role of LRIs in these functions remains unclear. Additionally, although it is well established that *Psi*/Gag interactions are necessary for retroviral gRNA packaging [4,12,14–19,21], it is not yet clear whether these interactions alone are sufficient for this process to occur.

**Fig 1:**
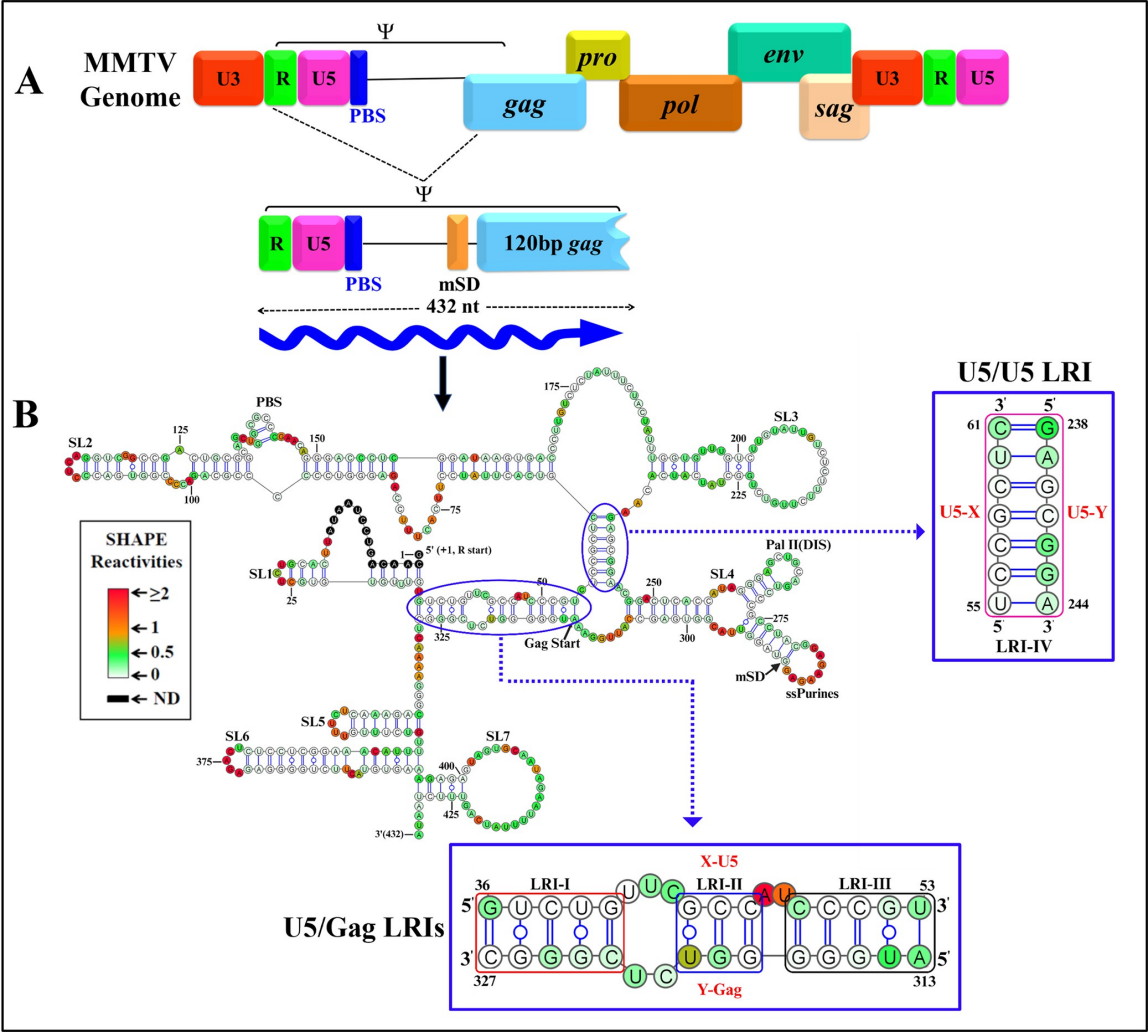
Schematic representation of the MMTV genome depicting the 5′ end harboring RNA packaging sequences (Ψ), along with its current RNA secondary structure model. ** (A)** Genome organization of MMTV and its RNA packaging signal (Ψ) beginning from the R region to 120 nucleotides of Gag, denoted by a blue wavy arrow. (**B)** hSHAPE-validated RNA secondary structure of MMTV packaging sequences located at the 5′ end, including LRIs-I-IV depicted in blue insets. Nucleotides are color-coded according to hSHAPE reactivity, with features annotated, such as stem-loops (SL1-7), PBS, DIS, single-stranded purines (ssPurines), mSD, and Gag start site. DIS, dimerization initiation site; LRI, long-range interaction; MMTV, mouse mammary tumor virus; mSD, major splice donor; PBS, primer binding site.

In this study, we tackled these questions using mouse mammary tumor virus (MMTV) as a model. MMTV, a *betaretrovirus*, induces breast cancer and, in certain instances, T-cell lymphomas in mice [56–58]. Unlike the majority of retroviruses, including lentiviruses, MMTV assembles in the cytoplasm into immature particles that later translocate to the plasma membrane for budding [59,60]. The MMTV *Psi* resides within the 5′ UTR and the first 120 nts of the *gag* gene [61], and recent studies have elucidated the structure–function relationships of several structural motifs within *Psi* [36,41,53]. Based on SHAPE (selective 2′ hydroxyl acylation analyzed by primer extension) data, the current secondary structure model of MMTV *Psi* suggests the presence of 4 evolutionary conserved LRIs [36,41,53]: LRIs I-III involve complementary base-pairing between U5 and Gag sequences that are approximately 291 nucleotides apart, while LRI IV is formed by complementary base-pairing of sequences within U5 that are approximately 190 nucleotides apart (Fig 1).

Briefly, we introduced mutations that disrupted LRIs I-IV and compensatory mutations designed to restore them, then analyzed the impact of these mutations on gRNA packaging, gRNA structure, and Pr77^Gag^ binding to *Psi*. Our findings indicate the presence of only 2 LRIs, which together form an extended LRI that significantly influences gRNA packaging, prompting us to propose a revised secondary structure model for MMTV *Psi*. Maintaining a *Psi* structure akin to the wild type (WT) emerges as crucial for efficient packaging. Surprisingly, most mutants displayed notable binding to Pr77^Gag^ regardless of their packaging ability, suggesting that Gag binding to *Psi* alone is insufficient for efficient packaging. Intriguingly, SHAPE footprinting experiments revealed that efficient RNA packaging correlated with Pr77^Gag^ binding to 2 specific sites within *Psi*, whereas binding elsewhere in *Psi* did not result in efficient packaging. These findings underscore the necessity for Pr77^Gag^ to bind to specific nucleotides within the correct structural context of *Psi* and support a model wherein the 3D structure of the *Psi*/Pr77^Gag^ complex governs the assembly of viral particles around gRNA, thereby facilitating effective discrimination against other viral and cellular RNA species that may also bind Gag efficiently.

## Results

### Experimental strategy

#### The three-plasmid genetic complementation assay

Conventional studies on retroviral RNA packaging using WT viruses are constrained by the involvement of the 5′ end of *gag* in *Psi*. This makes analyzing packaging using full-length WT viruses challenging, as mutations in *Psi* can affect the Gag ORF. While introducing point mutations is feasible, it often causes unexpected *cis* effects like mRNA nuclear export alterations [62–66]. To address these limitations and study the effects of mutations in MMTV *Psi* on gRNA packaging and propagation, we used a three-plasmid genetic complementation assay (S1 Fig) [36,41,53,61,67]. This involved co-transfecting a transfer vector (DA024) containing necessary *cis*-acting sequences and a marker gene [67], a packaging construct (JA10) expressing *gag-pro-pol* genes [67], and a VSV-G expression plasmid (MD.G) [68]. This split genome strategy generates pseudotyped virus particles from JA10 and MD.G, while the transfer vector produces packageable RNA. The replication of packaged RNA is restricted to a single round, allowing monitoring of vector RNA propagation via the *hygromycin resistance* gene. RNA packaging efficiency is assessed through real-time quantitative PCR (RT-qPCR), correlating hygromycin-resistant colonies with viral RNA content. This approach allows manipulation of RNA secondary structure sequences involved in MMTV gRNA packaging without affecting *gag-pro-pol* sequences.

#### A TaqMan assay for estimating relative packaging efficiency (RPE)

A new tailor-made MMTV TaqMan real-time qPCR assay was developed based on principles described earlier [61]. This new MMTV TaqMan assay was used along with a commercially available endogenous β-actin TaqMan assay to quantify both mutant and WT MMTV gRNAs expressed in the cytoplasm and packaged into virions. The amplification efficiency of MMTV and β-actin TaqMan assays was tested on serially diluted DA024 and a β-actin-expressing plasmid DNAs (S2A Fig; panels I and II). If the 2 assays have similar amplification efficiencies, the slope of the log input amount versus ΔCT should ideally be ≤0.1. Under our experimental conditions, the slope was 0.1076 (S2C Fig).

### Structure–function analysis of the long-range interactions support a new secondary structure model of the 5′ region of MMTV gRNA

#### Effects of mutations designed to disrupt and restore the U5/Gag LRIs (LRI I, II, and III) on MMTV gRNA packaging and propagation

**Fig 2:**
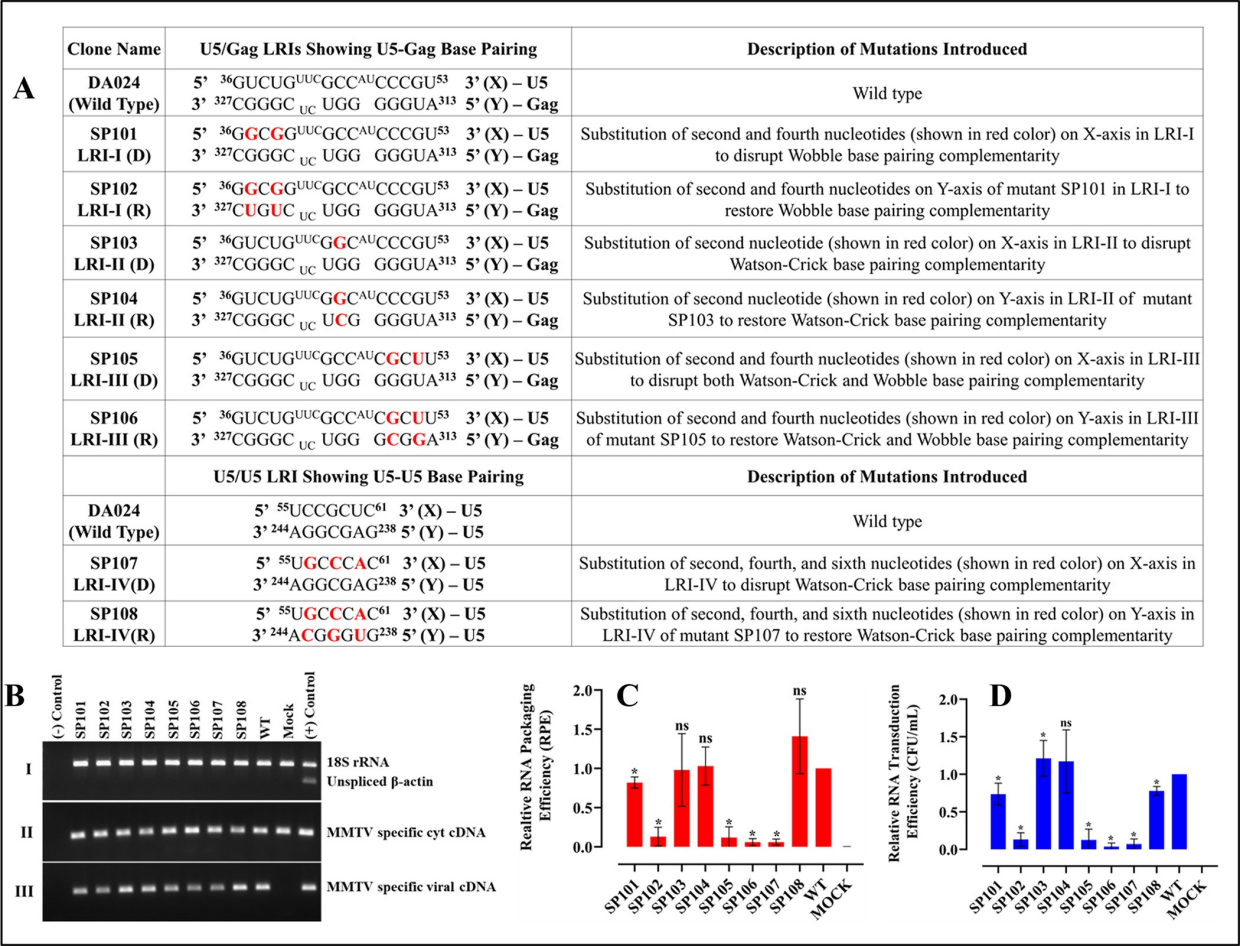
Role of LRIs-I-IV in MMTV gRNA packaging and propagation. (**A**) List of substitutions in the U5/Gag and U5/U5 LRIs, with mutations highlighted in red. **(B)** Representative gel images of the controls necessary for validating different aspects of the three-plasmid in vivo packaging and propagation assay: (I) multiplex amplification for nucleocytoplasmic fractionation technique, (II) PCR amplification for cDNAs prepared from cytoplasmic RNA fraction validating stability and nuclear export of transfer vector RNA, and (III) PCR amplification of packaged transfer vector RNA. **(C)** Packaging efficiency of mutant transfer vector RNAs relative to the wild type (DA024). **(D)** Relative propagation of MMTV transfer vector RNAs measured as normalized hygromycin resistant CFUs/ml for mutant transfer vectors compared to the wild type (DA024) vector. Mock samples contained only the transfer vector and no packaging construct. Data are presented as mean ± standard deviation from a minimum of 3 independent experiments performed in triplicates for RNA packaging (panel C) and in duplicates for RNA propagation (panel D). Differences compared to the wild-type were considered significant when *p* < 0.05 according to the nonparametric Mann–Whitney U test. The data underlying this Fig 2C and 2D can be found in S1 Data. CFU, colony-forming unit; gRNA, genomic RNA; LRI, long-range interaction; MMTV, mouse mammary tumor virus.

Considering the role of LRI sequences in anchoring and stabilizing RNA structures [10,35,39,52,54], we hypothesized that disrupting the base-pairing between the U5 and Gag sequences within LRI-I-IV could potentially negatively affect RNA packaging. To the contrary, stabilizing these interactions should restore structure and hence RNA packaging. To test this hypothesis, we first designed mutant SP101, in which the U•G wobble base pairs of LRI-I were disrupted (Fig 2A). Next, in mutant SP102, we restored 2 G•U wobble base pairing in LRI-I (Fig 2A).

Using our previously established three-plasmid genetic complementation assay [67], we evaluated the relative packaging efficiency (RPE) of the WT and LRI-I mutant RNAs by quantifying the amount of RNA packaged into the virions. To ensure the stable expression and successful transport of each vector RNA to the cytoplasm, nuclear and cytoplasmic RNA fractions were isolated from transfected cells and quantified. The quality of cell fractionation was assessed by the presence of unspliced β-actin mRNA in the nuclear and not in the cytoplasmic fractions [69]. Multiplex RT-PCR did not detect amplification of unspliced β-actin mRNA in the cytoplasmic fraction (Fig 2B; panel I), in contrast to the presence of 18S rRNA (Fig 2B; panel I) confirming the absence of nuclear leakage. Complementary DNAs (cDNAs) prepared from cytoplasmic RNA fractions and pelleted viral particles were amplified using specific primers (Fig 2B; panels II and III, respectively). Amplification of the desired region across all samples validated the efficient and stable expression as well as appropriate transport of transfer vector RNAs from the nucleus to the cytoplasm (Fig 2B; panel II). Finally, WT or mutant transfer vector RNAs in the cytoplasm and in the pelleted virus particles were quantified using RT-qPCR [36,41,53,61]. Part of the transfected supernatant was also used to infect HeLa CD4+ cells to evaluate the ability of the produced virions to transduce the packaged RNA into the target cells. This was achieved by monitoring the emergence of hygromycin-resistant colonies following selection of the infected cultures with hygromycin B-containing medium.

Compared to the wild-type, SP101 RNA (Fig 2A) exhibited only moderate reductions in gRNA packaging (RPE = 0.82 ± 0.07; *p*-value < 0.05; Fig 2C) and propagation (CFU = 0.74± 0.14; *p*-value < 0.05; Fig 2D). Conversely, mutant SP102 showed 87% reduction in both RNA packaging (RPE = 0.13± 0.11; *p*-value < 0.05; Fig 2C) and propagation (CFU = 0.13 ± 0.08; *p*-value < 0.05; Fig 2D). These results indicate that mutations in the X and Y strands of LRI-I (Fig 2A) have cooperative (or additive) rather than compensatory effects suggesting that LRI-I does not exist.

Analysis of mutants SP103 and SP104, which were designed to disrupt and restore LRI-II, respectively (Fig 2A), did not allow to conclude about the existence of this LRI. Indeed, compared to the wild type, mutant SP103 did not demonstrate any defects in both RNA packaging (RPE = 0.98 ± 0.46; *p*-value > 0.05; Fig 2C) as well as in RNA propagation (CFU = 1.21 ± 0.23; *p*-value < 0.05; Fig 2D). Similarly, mutant SP104 revealed nearly identical results as the wild type for both RNA packaging and propagation (RPE = 1.03 ± 0.24; *p*-value > 0.05; CFU = 1.17 ± 0.41; *p*-value > 0.05; Fig 2C and 2D). These results indicate that the sequences mutated in SP103 and SP104 play no significant role in RNA packaging, irrespective of the existence, or not, of LRI-II.

Mutants SP105 and SP106 were designed to disrupt and restore LRI-III, respectively (Fig 2A). RNA packaging and propagation of these mutants were almost completely abolished, regardless of whether the base pairing sequence complementarity of LRI-III was disrupted (RPE = 0.12 ± 0.13; *p*-value < 0.05; CFU = 0.12 ± 0.14; *p*-value < 0.05; Fig 2C and 2D) or restored (RPE = 0.06 ± 0.03; *p*-value < 0.05; CFU = 0.03 ± 0.04; *p*-value < 0.05; Fig 2C and 2D). These results suggest that either the identity of the sequences forming LRI-III is crucial, or the proposed LRI-III may not actually exist.

#### Effects of mutations designed to disrupt and restore the U5/U5 LRI (LRI-IV) on MMTV gRNA packaging and propagation

To investigate the role of LRI-IV, the longest LRI in MMTV gRNA *Psi* (Fig 1B), on viral replication, we created mutants SP107 and SP108, designed to disrupt and restore 3 of the 7 base-pairs of LRI-IV, respectively (Fig 2A). SP107 revealed nearly complete abrogation of RNA packaging (RPE = 0.06 ± 0.03; *p*-value < 0.05; Fig 2C) and propagation (CFU = 0.07 ± 0.06; *p*-value < 0.05; Fig 2D). On the other hand, SP108 showed restoration of both RNA packaging (RPE = 1.41 ± 0.47; *p*-value > 0.05; Fig 2C) and RNA propagation (CFU = 0.78 ± 0.05; *p*-value < 0.05; Fig 2D) to WT levels. These results support the existence and biological significance of LRI-IV in MMTV replication. Furthermore, they highlight the importance of complementarity among nucleotides forming LRI-IV, rather than the sequence itself, for RNA packaging.

#### Mutations in LRIs do not affect RNA dimerization

**Fig 3:**
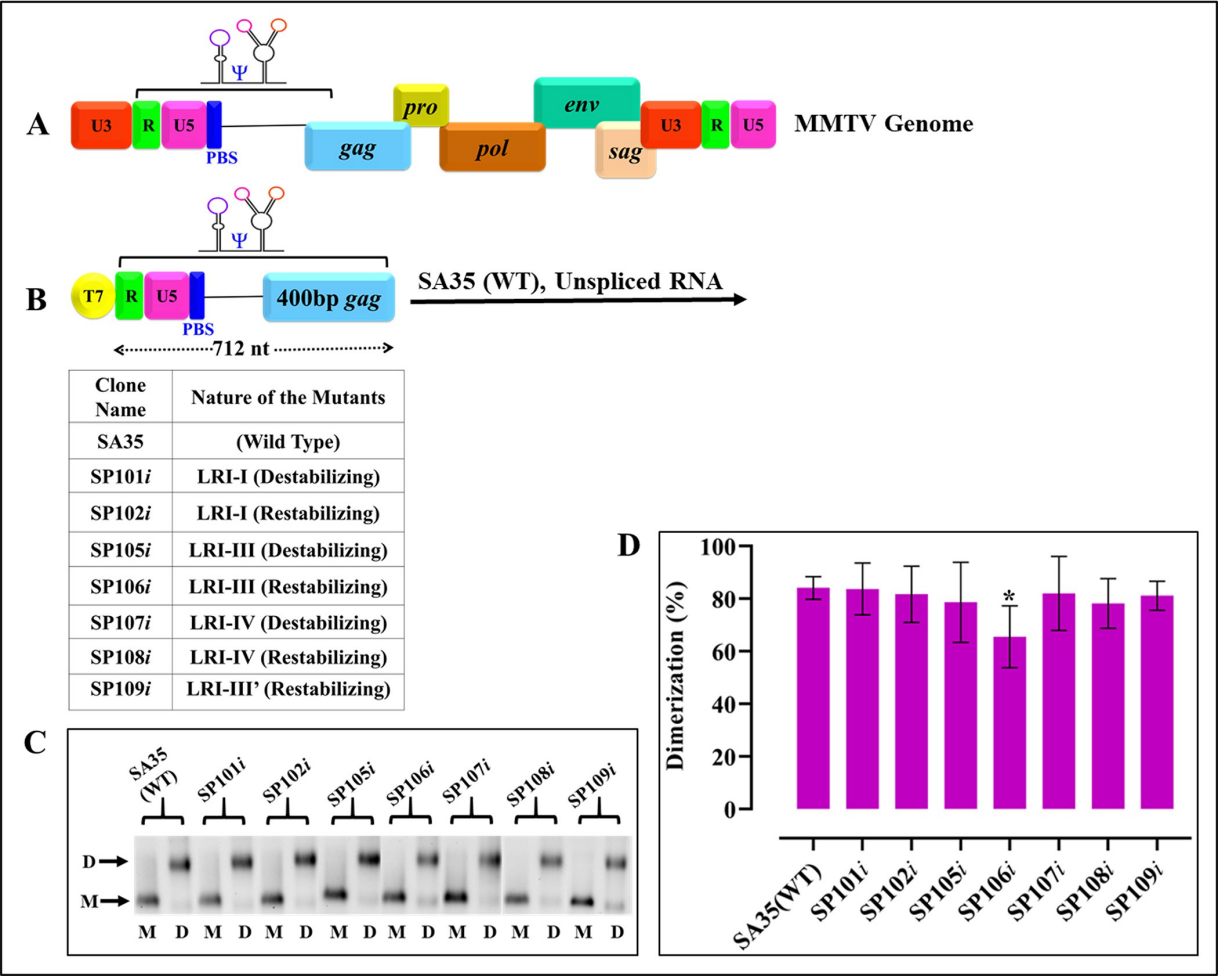
Dimerization of LRI mutants remains unaffected by the introduced mutations. **(A)** Schematic representation of the MMTV genome indicating the location of MMTV gRNA packaging determinants. **(B)** Illustration of the packageable vector RNA from R to 712 nucleotides expressed from a T7 expression plasmid, with the table detailing the names of the clones and the nature of the mutations. **(C)** Representative gel images displaying in vitro dimerization of WT (SA35) and LRI mutant RNAs in TBM buffer. M and D labels below the lanes indicate monomer and dimer buffers used for dimerization experiments. Monomeric and dimeric RNA species are denoted by letters M and D, respectively, on the gel’s horizontal margin. Gels have been cropped as indicated by vertical white spaces to show the relevant areas only. **(D)** Histograms illustrating the dimerization efficiencies of mutant RNAs compared to WT (SA35) calculated through densitometric analysis of bands from 3 independent experiments. Dimerization efficiency was determined by dividing the intensity of the dimeric RNA band by the intensity of the band from the total RNA (i.e., sum of dimer and monomer bands). No statistically significant differences (*p*-values > 0.05) were observed in the ability of the mutant clones to dimerize when compared to the WT (SA35) according to the nonparametric Mann–Whitney U test, except for mutant SP106*i* (*p* < 0.05). The data underlying this Fig 3D can be found in S1 Data. gRNA, genomic RNA; LRI, long-range interaction; MMTV, mouse mammary tumor virus; WT, wild type.

Packaging of retroviral gRNA is closely linked to its dimerization [3,4,12,14,17,24,36,38,70,71]. Therefore, to test if the mutations were introduced in the LRIs affect RNA dimerization, 712 nucleotides from the 5′ end of the MMTV genome, encompassing wild type as well as mutant packaging sequences were cloned into a T7 promoter-containing plasmid (Fig 3A and 3B) and dimerization assays were performed using in vitro transcribed RNAs. Interestingly, both LRI packaging and non-packaging RNA mutants were found to dimerize at WT levels and showed no statistically significant differences, except for mutant SP106*i* (Fig 3C and 3D). Note that the RNA monomer species migrates slightly faster in the dimer condition than in the monomer condition, as the high ionic strength of the dimer buffer compacts the RNA. These results reveal that while the LRI destabilizing and restabilizing mutants affected RNA packaging, they did not adversely affect RNA dimerization, further validating that the effects being observed on RNA packaging were bona fide and not because of any effects on RNA dimerization.

#### Structure–function analyses of LRI mutants

Next, we investigated the RNA secondary structure of the 5′ end of the WT and selected LRI mutant MMTV genomes using hSHAPE [72–75]. To that goal, we treated in vitro transcribed RNAs corresponding to the 712 nucleotides at the 5′ end of the WT MMTV genome (SA35; Fig 3B) and LRI mutants (SP101*i*, 102*i*, SP105*i*-SP109*i* RNAs; Fig 3B) with BzCN. The resulting modifications of the flexible riboses were identified as stops during the extension of fluorescently labeled primers by reverse transcriptase and cDNA analysis by capillary electrophoresis. The SHAPE reactivity of each nucleotide obtained from 3 experiments using QuShape [76] (S1 Table) were utilized as pseudoenergy constraints to derive RNA secondary structure models for MMTV *Psi* of the WT and LRI mutants using the RNAStructure version 6.1 program [77].

**Fig 4:**
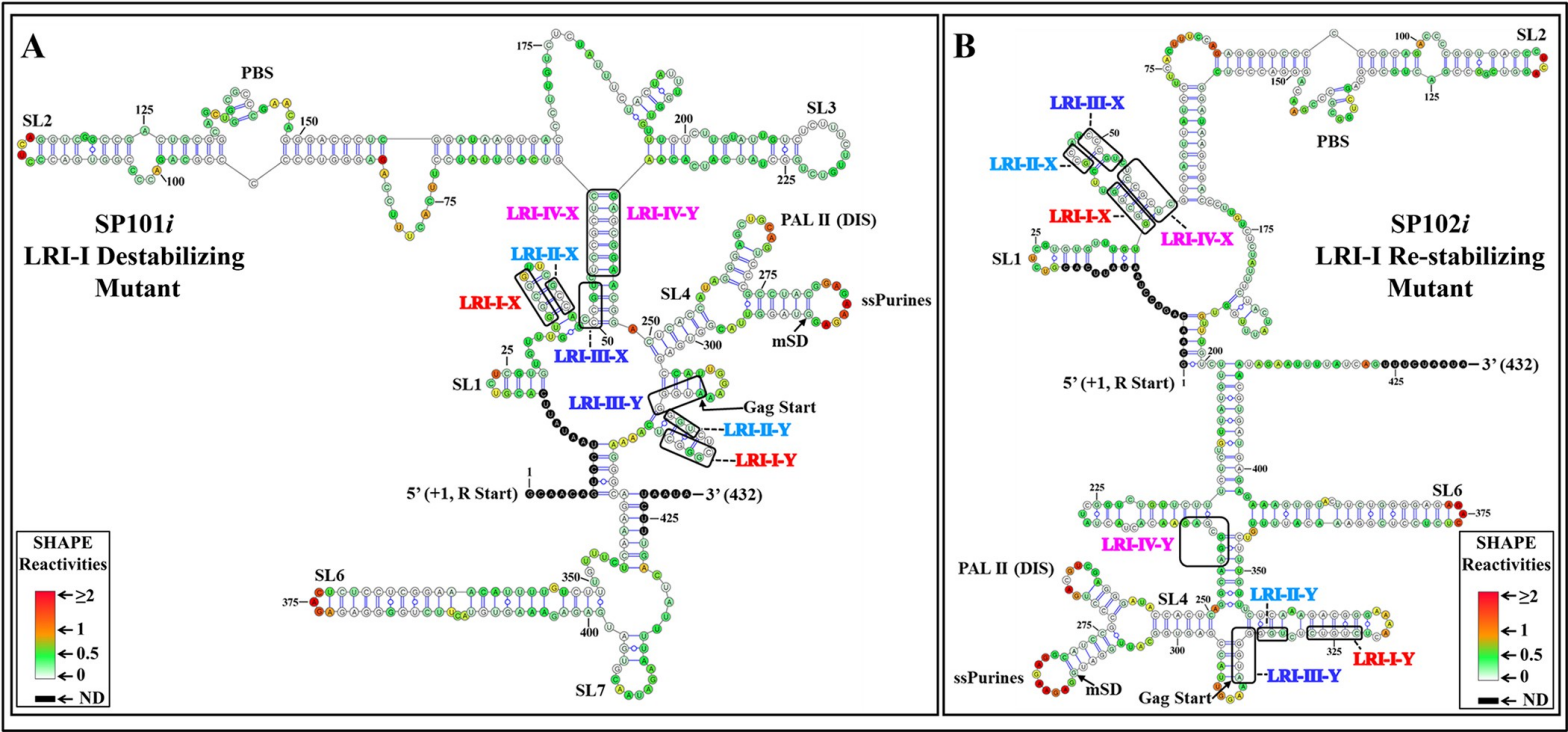
hSHAPE-validated RNA secondary structure model of the LRI-I mutants. The first 432 nucleotides of the 712 nt long RNA are shown. **(A)** Mutant SP101*i* was designed to destabilize LRI-I. **(B)** Mutant SP102*i* was designed to re-stabilize LRI-I. Structural elements that are also present in the WT structure (SA35) such as stem-loops (SL1-7), PBS, DIS, single-stranded purines (ssPurines), and mSD are marked as in Fig 1. The X and Y-strands of different LRIs in both destabilizing and re-stabilizing mutants are boxed and labeled in different colors for clarity. Nucleotides are color-coded according to SHAPE reactivity derived from a minimum of 3 independent experiments, with data provided in S1 Table. DIS, dimerization initiation site; LRI, long-range interaction; mSD, major splice donor; PBS, primer binding site; WT, wild type.

As SP101, designed to disrupt LRI-I, exhibited minimal or no packaging defect, while SP102, designed to restore LRI-I, was severely compromised (Fig 2), we performed hSHAPE on these mutants. Stem loops (SLs) 1–4 and LRI-IV, which were present in the secondary structure of the WT MMTV gRNA proposed earlier [53], are maintained in the resulting RNA secondary structure model of SP101*i*, while LRIs-I-III are lost (Fig 4A). Unexpectedly, LRI-I was not restored in SP102*i*; indeed, this mutant also lost the other 3 LRIs, namely LRI-II, III, and IV (Fig 4B), suggesting that the packaging defect of SP102 (Fig 2) might be due to the disruption of LRI-IV.

**Fig 5:**
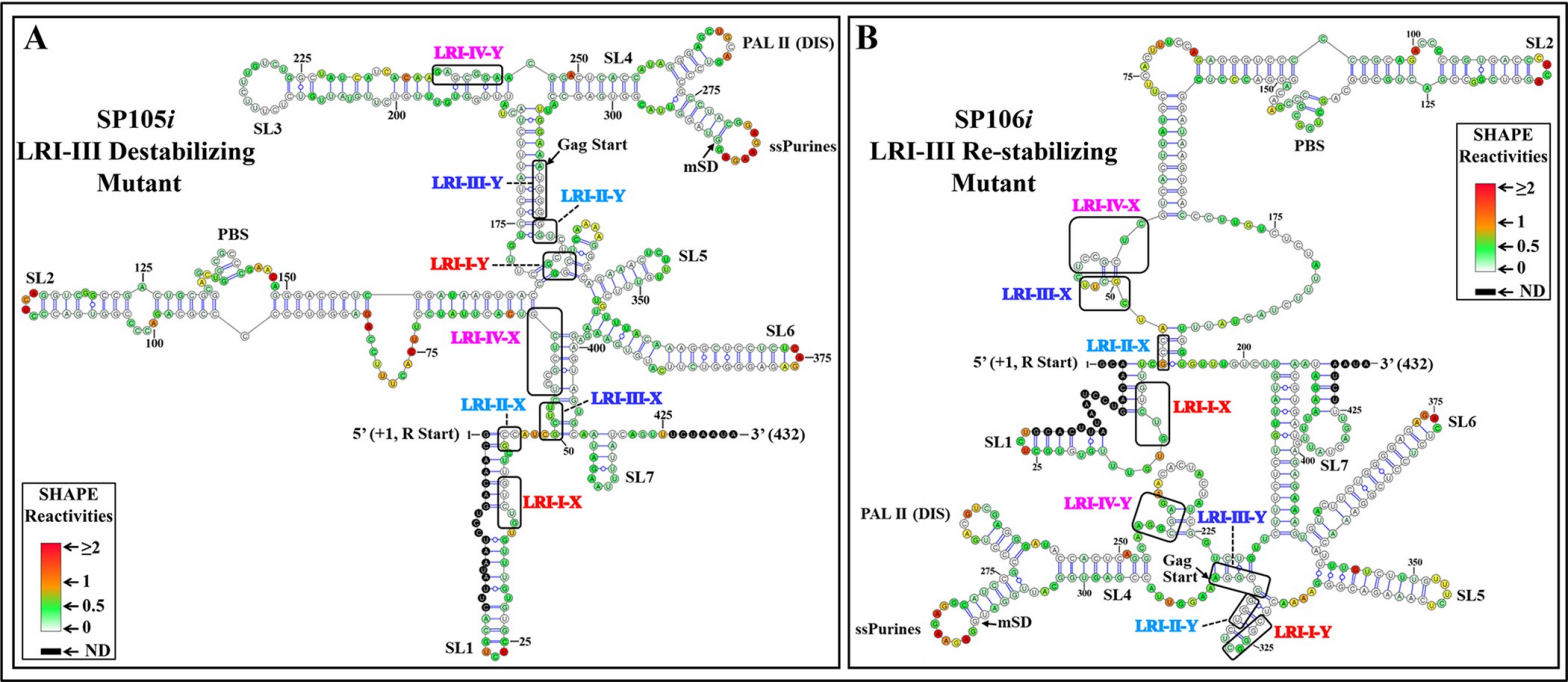
hSHAPE-validated RNA secondary structure model of the LRI-III mutants. The first 432 nucleotides of the 712 nt long RNA are shown. **(A)** Mutant SP105*i* was designed to destabilize LRI-III. **(B)** Mutant SP106*i* was designed to re-stabilize LRI-III. Structural elements that are also present in the WT structure (SA35) such as stem-loops (SL1-7), PBS, DIS, single-stranded purines (ssPurines), and mSD are marked as in Fig 1. The X and Y-strands of different LRIs in both destabilizing and re-stabilizing mutants are boxed and labeled in different colors for clarity. Nucleotides are color-coded according to SHAPE reactivity derived from a minimum of 3 independent experiments, with data provided in S1 Table. DIS, dimerization initiation site; LRI, long-range interaction; mSD, major splice donor; PBS, primer binding site; WT, wild type.

Next, we tested the structure of LRI-III mutant RNAs SP105*i* and SP106*i*, as these 2 mutants exhibited severe defects in RNA packaging and propagation (Fig 2). According to their hSHAPE structures (Fig 5A and 5B), all 4 LRIs were lost in these mutants, consistent with their loss of function (Fig 2).

**Fig 6:**
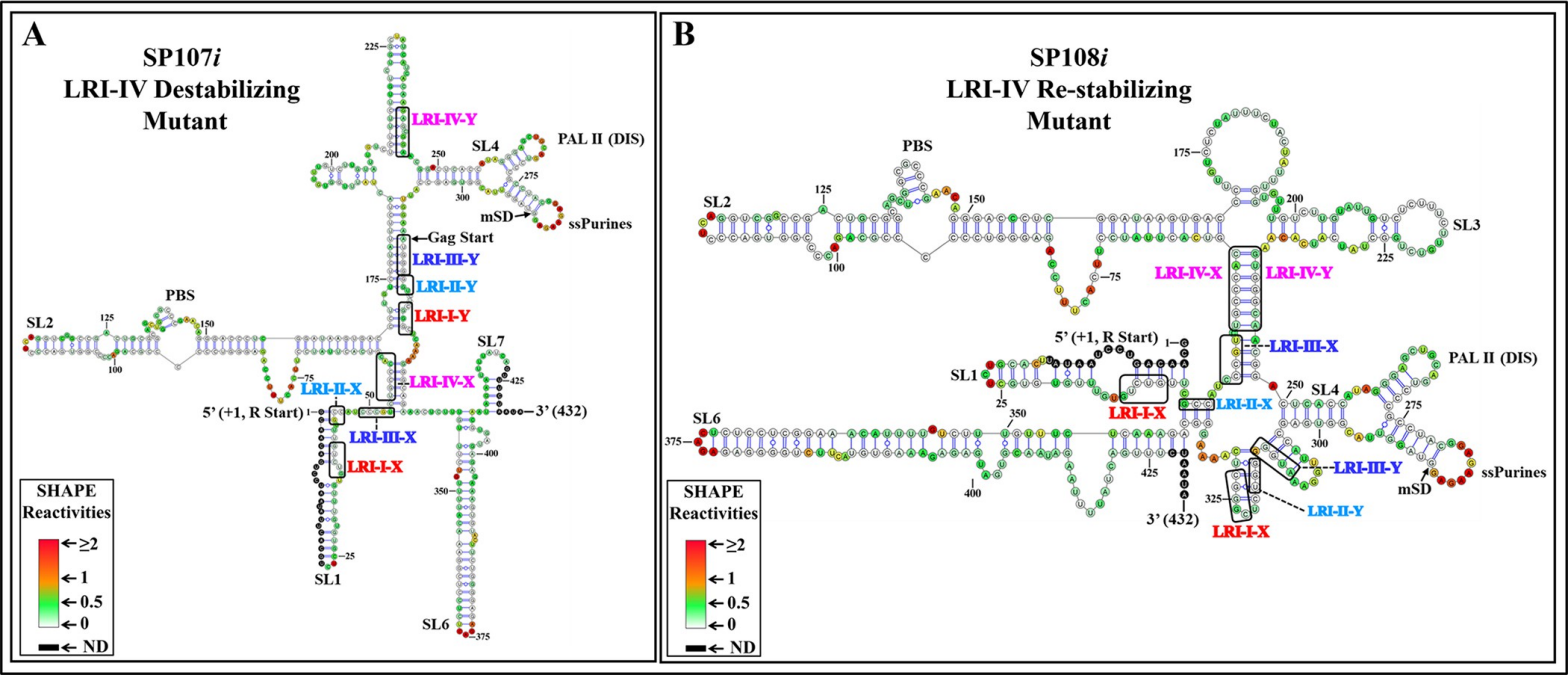
hSHAPE-validated RNA secondary structure model of the LRI-IV mutants. The first 432 nucleotides of the 712 nt long RNA are shown. **(A)** Mutant SP107*i* was designed to destabilize LRI-IV. **(B)** Mutant SP108*i* was designed to re-stabilize LRI-IV. Structural elements that are also present in the WT structure (SA35) such as stem-loops (SL1-7), PBS, DIS, single-stranded purines (ssPurines), and mSD are marked as in Fig 1. The X and Y-strands of different LRIs in both destabilizing and re-stabilizing mutants are boxed and labeled in different colors for clarity. Nucleotides are color-coded according to SHAPE reactivity derived from a minimum of 3 independent experiments, with data provided in S1 Table. DIS, dimerization initiation site; LRI, long-range interaction; mSD, major splice donor; PBS, primer binding site; WT, wild type.

The SP107 mutant, designed to disrupt LRI-IV, lost its ability to package and propagate RNA, while mutant SP108, designed to restore LRI-IV, restored RNA packaging and propagation to WT levels (Fig 2). Accordingly, the hSHAPE-validated structure of SP107*i* revealed not only the loss of LRI-IV but also the loss of the other 3 LRIs (Fig 6A), whereas in the case of SP108*i*, LRI-IV was restored, but not the other LRIs, while adopting a structure globally similar, though not identical, to the wild type (Fig 6B). Altogether, these results indicated that the structure of LRI-IV, but not its sequence is essential for function.

An intriguing observation from the hSHAPE structures is the consistent retention of 2 critical structural patterns, SL2 and the branched SL4, in their original locations across all mutants (Figs 4–6). These motifs encompass nucleotides recognized as primary Gag-binding sites crucial for MMTV RNA packaging [53]. Despite this, certain mutants exhibited defects in RNA packaging (Fig 2), suggesting that Pr77^Gag^ was unable to bind to these sites in those specific mutants.

#### A new secondary structure model supported by structural and functional analysis

Overall, the combined biological and structural data suggest that the previously proposed LRI-I and LRI-II [36,53] may not necessarily exist. LRI-III mutants SP105 and SP106 exhibited pronounced defects in RNA packaging, which suggests that while these sequences are important for function, they may not be involved in complementary base-pairing, as initially thought [36,53]. In contrast, biological and structural data obtained with mutants SP107 and SP108 strongly support the existence of LRI-IV, as initially proposed [53].

**Fig 7:**
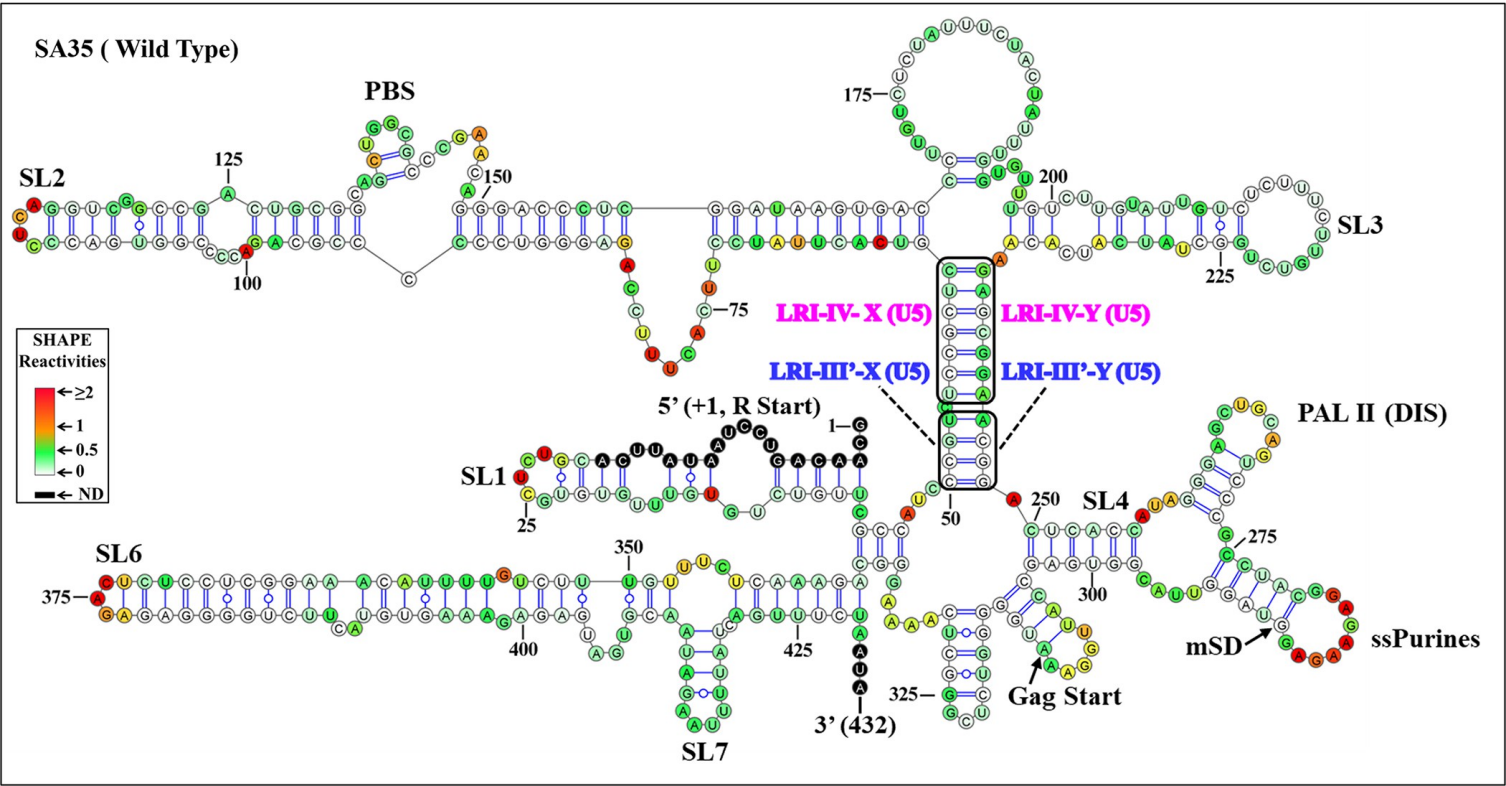
The new hSHAPE-validated RNA secondary structure model of the first 432 nucleotides, obtained through biochemical re-probing of the WT (SA35) 5′ end of MMTV. The updated structure model closely resembles the previously proposed structure but exhibits notable differences in the LRIs and minor differences in SLs 5, 6, and 7. In the new structure model, LRIs I and II are absent, while LRI-IV remains unchanged. Additionally, the X-strand of the initially proposed LRI-III now base pairs with complementary sequences 195 nucleotides downstream within U5, instead of Gag. Structural elements consistent with the earlier proposed model, such as SLs1-7, PBS, DIS, single-stranded purines (ssPurines), and mSD, are present in their native positions and labeled as in Fig 1. The X and Y-strands of LRIs III’ and IV are shown in boxes and labeled with different colors for clarity. Nucleotides are color-coded according to SHAPE reactivity derived from a minimum of 3 independent experiments, with data provided in S1 Table. DIS, dimerization initiation site; LRI, long-range interaction; MMTV, mouse mammary tumor virus; mSD, major splice donor; PBS, primer binding site; SL, stem loop; WT, wild type.

To confirm these results, we re-probed the same region of WT (SA35) MMTV gRNA (712 nucleotides from R to 400 nucleotides of Gag). The new hSHAPE-validated structure (Fig 7) closely resembles the earlier proposed structure, but differs notably in the LRIs, with minor differences in SLs 5–7. Specifically, LRI-I is not observed, and LRI-II’s U5 sequences (5′ GCC 3′; nts 44–46) are base paired with a different Gag sequence (3′ CGG 5′; nts 337–335) (Fig 7). However, the existence and biological significance of this alternative LRI remains uncertain because when we mutated the U5 sequences (which forms a LRI in both the old and the new structure models), it did not affect RNA packaging. In the new model, LRI-III is present in a modified form that we named LRI-III’: its 5′ sequence (5′ CCGU 3′; nts 50–53) is base paired with sequences within U5 (5′ ACGG 3′; 244–248) instead of Gag. Consistently, no changes are observed in LRI-IV in the new hSHAPE-validated structure, which corroborates the structure–function data of LRI-IV mutants presented above (Fig 7).

The same software and input sequences (432 nucleotides) were used to predict the secondary structure of the MMTV 5′ region in this work and our previous publication [53]. Both studies also utilized the same RNA and experimental probing conditions. However, new primers for cDNA synthesis were introduced in this study (refer to the Material and methods section). The 3′ primer was redesigned because the one used in our previous study was unsuitable for analyzing the mutants used here. More importantly, a new 5′ primer was designed to improve the signal-to-noise ratio at the 5′ end of the RNA. The enhanced quality of the SHAPE data at the 5′ end of RNA (SA35) resulted in our new modeling, providing a slightly different secondary structure. Notably, our new modeling identified the previously published secondary structure model as the third most stable structure. For large RNAs, it is common for small differences in experimental data to result in different “most stable” structures, as multiple structures often have very similar minimal free energies [78].

**Fig 8:**
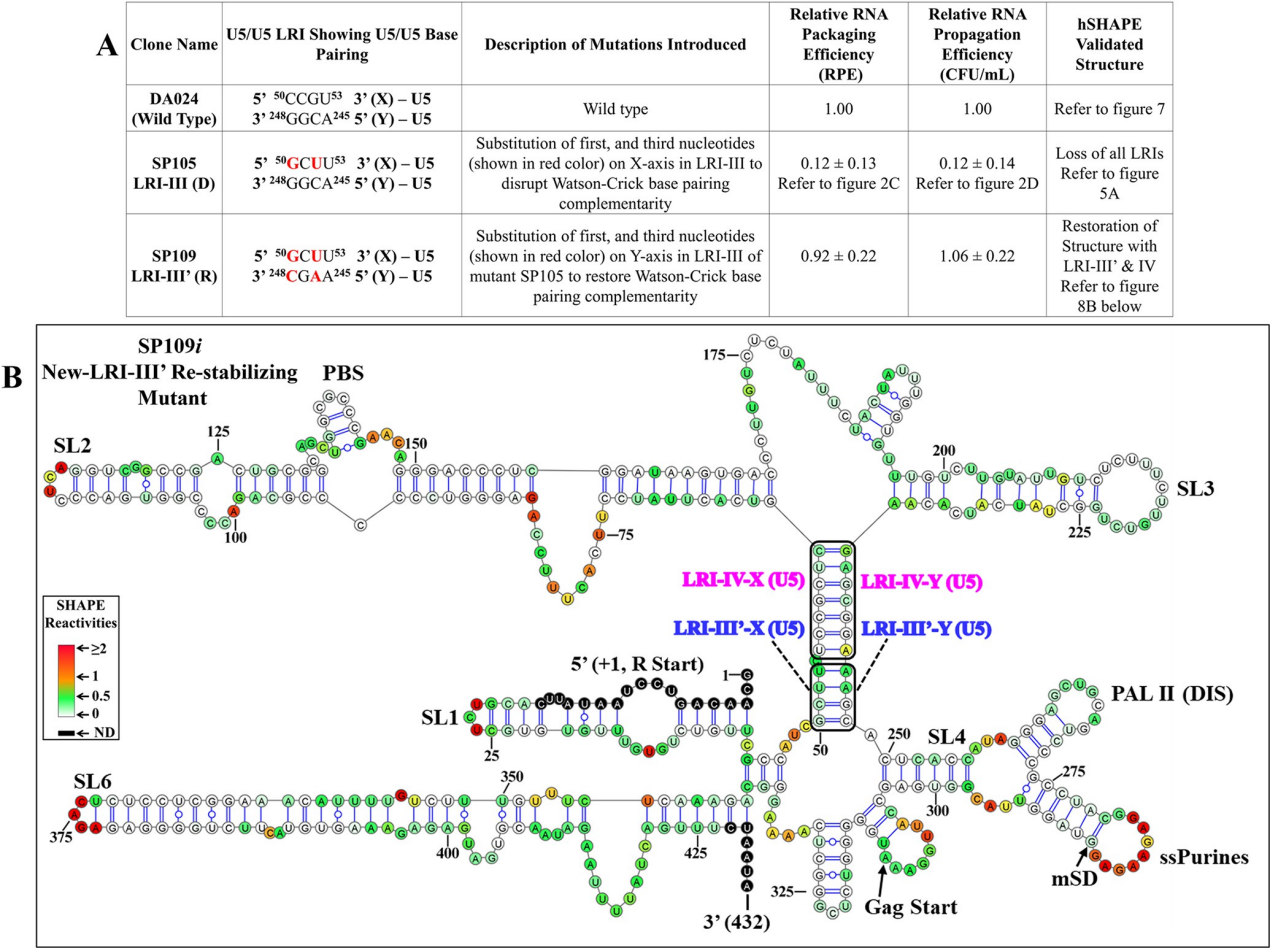
LRI-III’ with complementary heterologous sequences restores RNA packaging and structure. **(A)** Description of the substitution mutants in the newly proposed LRI-III with red nucleotides indicating introduced mutations aimed at destabilizing or re-stabilizing complementarity with heterologous sequences. Columns 4, 5, and 6 show results of the effect of mutations on RNA packaging, propagation, and structure, respectively. The RNA packaging data shown here is from a minimum of 3 independent experiments performed in triplicates (±SD). The RNA propagation data shown here is from a minimum of 3 independent experiments performed in duplicates (±SD). **(B)** A hSHAPE-validated RNA secondary structure model depicts the restored LRI-III’ mutant SP109*i*, designed to re-stabilize LRI-III’ with heterologous complementary sequences. Structural elements that are also present in the new WT structure (SA35) such as stem-loops (SL1-7), PBS, DIS, single-stranded purines (ssPurines), and mSD are marked as in Fig 7. The X and Y-axes of LRIs III’ and IV are boxed and labeled in different colors for clarity. Nucleotides are color-coded according to SHAPE reactivity derived from a minimum of 3 independent experiments, with data provided in S1 Table. The data underlying this Fig 8A can be found in S1 Data. DIS, dimerization initiation site; LRI, long-range interaction; mSD, major splice donor; PBS, primer binding site; SL, stem loop; WT, wild type.

To examine the role of the new LRI-III’ in MMTV gRNA packaging, we utilized mutant SP105, which disrupts it, and created mutant SP109, containing the same mutations as SP105 and additional ones designed to restore LRI-III’ (Fig 8A). As observed earlier, SP105 mutant exhibited severely compromised RNA packaging and propagation; however, when the new LRI-III’ structure was restored, RNA packaging was restored to almost WT levels (Fig 8A). Accordingly, hSHAPE indicated that the secondary structure of mutant SP109*i* is similar to wild type, as expected (compare Fig 7 with Fig 8B). These results suggest that the secondary structure of the MMTV *Psi* RNA is held together by a long stretch of 11 Watson–Crick–Franklin base pairs (interrupted by a 1 nucleotide bulge), the sequence of which is not important for function.

### Binding of Pr77^Gag^ to the mutant RNAs does not correlate with RNA packaging

**Fig 9:**
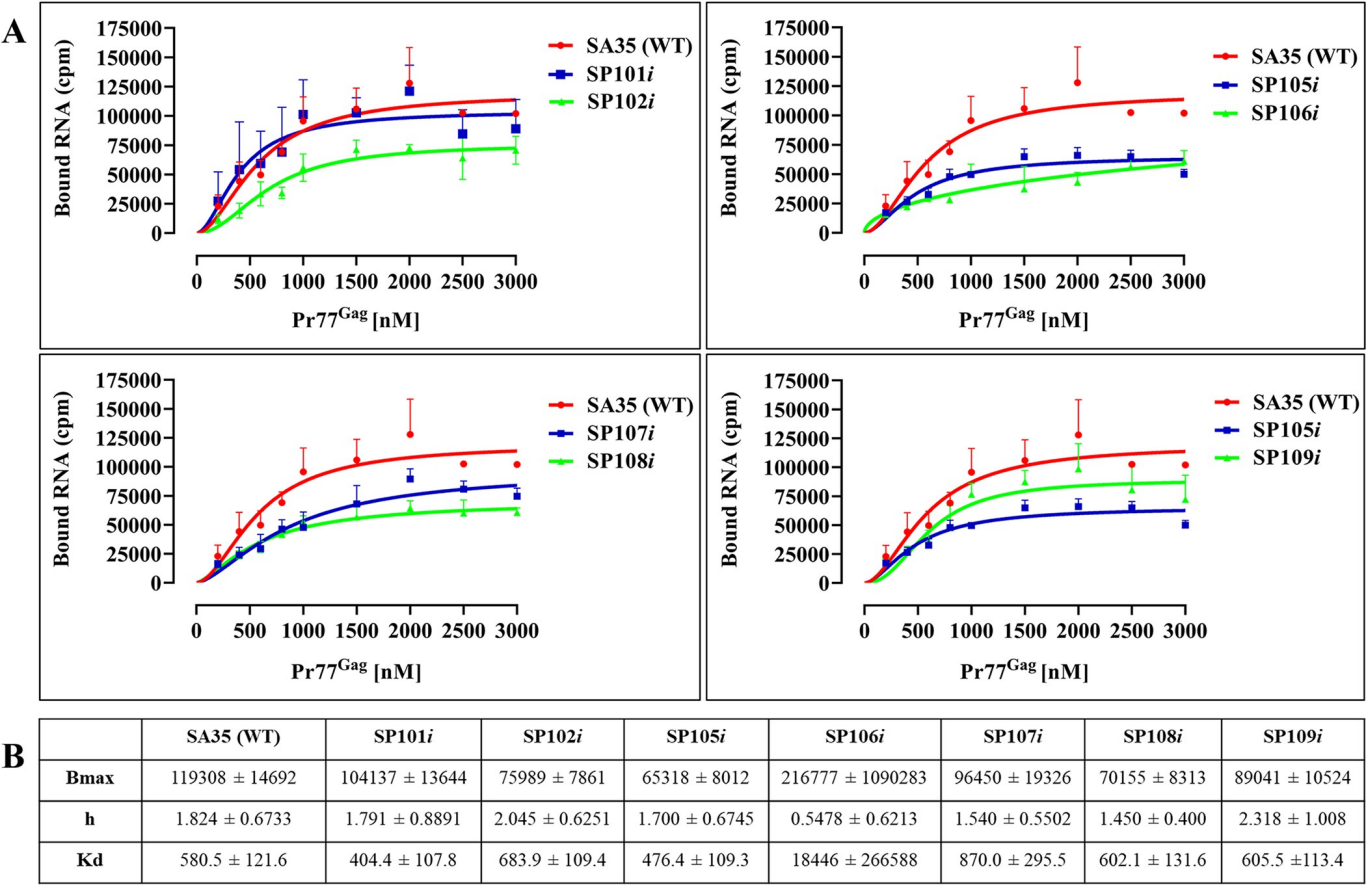
MMTV Pr77^Gag^ binding to the WT and LRI mutant RNAs using filter-binding assays. **(A)** The membrane-bound radioactivity of the wild type WT (SA35) unspliced and mutant RNAs was quantified at increasing concentrations of MMTV Pr77^Gag^. Data points were fitted with the Hill’s equation, with error bars denoting standard deviation from the mean of 3 independent experiments. **(B)** Pr77^Gag^ binding parameters to the WT and LRI mutant MMTV *Psi* region as derived using Hill’s equation. Cumulative data is derived from 3 independent experiments. Bmax: represents the maximum-specific binding; h: represents the Hill slope; Kd: represents the Pr77^Gag^ concentration needed to achieve a half-maximum binding followed by their standard deviation. The data underlying this Fig 9A and 9B can be found in S1 Data. LRI, long-range interaction; MMTV, mouse mammary tumor virus; WT, wild type.

Next, we performed filter-binding assays to determine whether the gRNA packaging results obtained with the LRI mutants could be correlated to their ability to bind to Pr77^Gag^. The Pr77^Gag^ protein we used in these assays is able to form virus-like particles (VLPs) in vitro as well as in vivo [53,79]. Furthermore, the Gag VLPs formed by Pr77^Gag^-His_6_ fusion protein in human embryonic kidney (HEK293T) cells efficiently package RNA containing the MMTV *Psi* [53,79]. Based on the ratio of UV absorbance at 260 and 280 nm, the purified protein was observed to be devoid of nucleic acids. Finally, dynamic light scattering (DLS) revealed that the average hydrodynamic radius (Rh) determined based on volume (percent) and number (percent) distribution was around 6.00 nm, corresponding to Pr77^Gag^ trimers ([53]; S3 Fig). Thus, this purified protein was used in filter-binding assays along with radiolabeled in vitro transcribed RNA from WT (SA35) and LRI mutants (SP101*i*, 102*i*, SP105*i*-SP109*i* RNAs; Fig 3B). As is evident from Fig 9A, almost all mutant RNAs (whether packaging or non-packaging) were able to bind to Pr77^Gag^ efficiently; however, some of the non-packaging mutants exhibited a lower binding plateau, which could reflect a different stoichiometry. Interestingly, as shown in Fig 9B, all mutants except SP106*i* bind Pr77^Gag^ with a Kd and a cooperativity (h) similar to the WT. These results suggest that Pr77^Gag^ efficiently binds to the MMTV *Psi* region regardless of its ability to promote RNA packaging.

### Binding of Pr77^Gag^ to specific sites is critical for efficient MMTV gRNA packaging

Since the filter-binding assays revealed that Pr77^Gag^ binding affinity does not correlate with RNA packaging efficiency, we asked whether Pr77^Gag^ binds at the same sites within *Psi* of the packaging and non-packaging mutants. To this goal, we identified specific nucleotides that bind to Pr77^Gag^ by conducting RNA modification (via BzCN), both in the presence and absence of Pr77^Gag^. Reduced hSHAPE reactivity in the presence of Pr77^Gag^ revealed the footprints of Pr77^Gag^ on the WT and mutant *Psi*. To prevent nonspecific binding, hSHAPE was conducted with excess spliced *env* RNA (AK29; Fig 3C) at a high (4-fold) molar concentration. Pr77^Gag^ was used at a concentration 10-fold higher than the *K*d value, ensuring complete saturation of the high-affinity binding sites. These conditions identified high-affinity binding sites in other retroviral *Psi* footprinting experiments [46,53,80].

**Fig 10:**
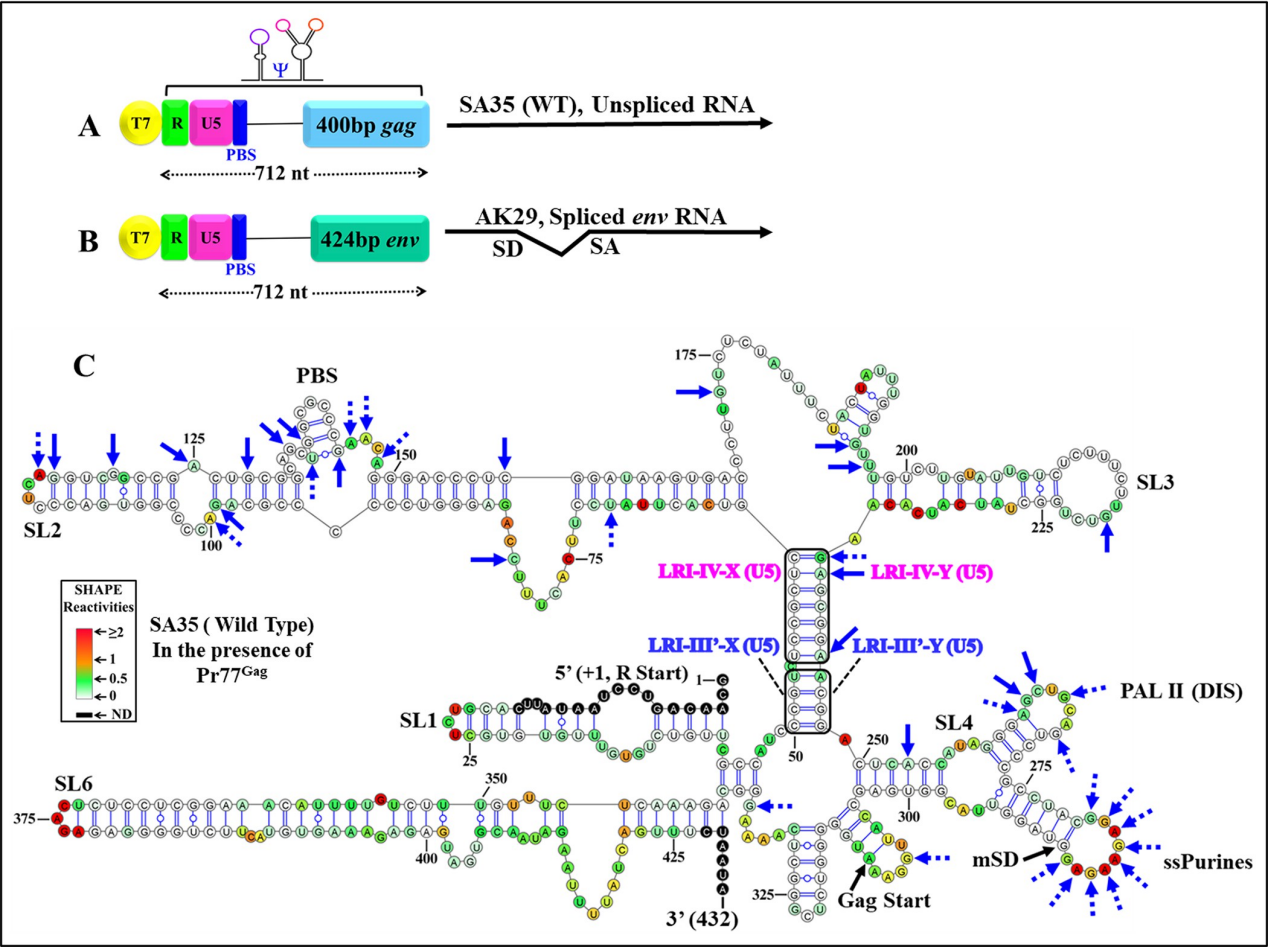
Footprints of Pr77^Gag^ on the WT structure model. **(A)** Schematic illustration of the MMTV WT packageable vector (SA35) RNA from R to 712 nucleotides expressed from a T7 expression plasmid. **(B)** Illustration of the *envelope* (*env*) spliced RNA (AK29) from R to 712 nucleotides expressed from a T7 expression plasmid. **(C)** hSHAPE analysis was carried out both with and without Pr77^Gag^. The mean triplicate SHAPE reactivity obtained without Pr77^Gag^ was used to predict the RNA secondary structure model of WT (SA35) RNA. Subsequently, the mean hSHAPE reactivities obtained with Pr77^Gag^ were overlaid onto the RNA secondary structure model predicted in the absence of Pr77^Gag^. Notably, nucleotides within the previously identified primary Gag-binding sites, such as single-stranded purines (ssPurines) and the PBS, exhibited significant reductions in hSHAPE reactivities. Nucleotides in ssPurines, PBS, and all other nucleotides marked by arrows show significant reduction in SHAPE reactivities. The hSHAPE reactivity key was developed based on the mean of hSHAPE reactivities for each nucleotide, as shown in S3 Table. The data shown is from a minimum of 3 independent experiments conducted both in the absence and presence of Pr77^Gag^. All nucleotides that show a reactivity decrease >40% upon Gag addition, also show statistically significant difference according to the Mann–Whitney non parametrical U test (*p* < 0.05). MMTV, mouse mammary tumor virus; PBS, primer binding site; WT, wild type.

**Fig 11:**
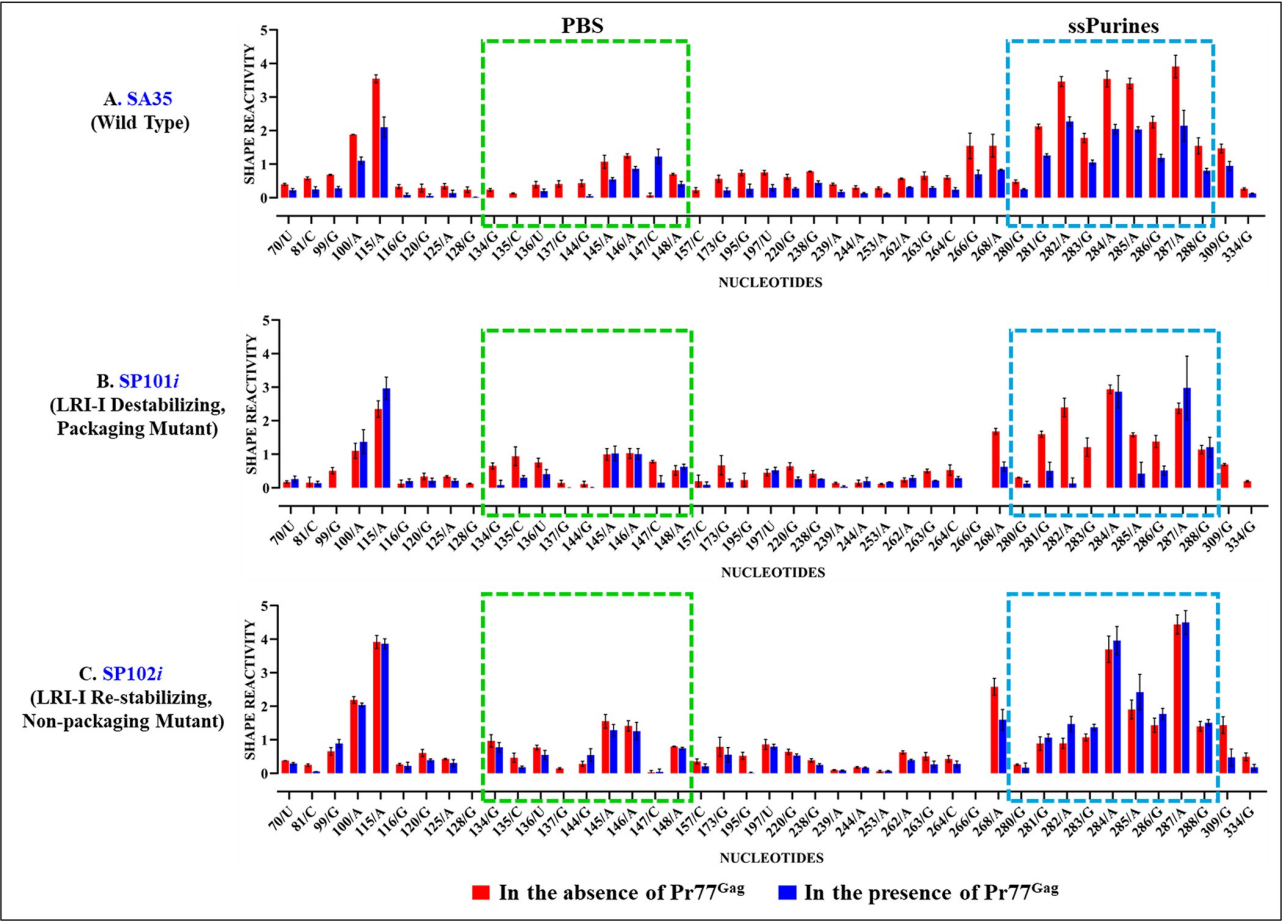
Footprints of Pr77^Gag^ on the WT and LRI-I mutant packaging signal RNAs. Histograms showing the SHAPE reactivities of nucleotides in the absence (red bars) and presence (blue bars) of Gag for the packaging signal RNAs of: **(A)** wild type (SA35), **(B)** mutant SP101*i* designed to destabilize LRI-I, and **(C)** mutant SP102*i* designed to re-stabilize LRI-I. The dashed boxes mark the already identified primary Gag-binding sites, such as single stranded purines (ssPurines) and the PBS. For full details of nucleotides attenuation of SHAPE reactivities in the absence and presence of Pr77^Gag^ from a minimum of 3 independent experiments, see S3 Table and S4 and S5 Figs. All nucleotides that show a reactivity decrease >40% upon Gag addition, also show statistically significant difference according to the Mann–Whitney non parametrical U test (*p* < 0.05). The data underlying this figure can be found in S1 Data. LRI, long-range interaction; PBS, primer binding site; WT, wild type.

When hSHAPE was conducted on WT (SA35) RNA in the absence of Pr77^Gag^, the reactivity pattern was consistent with the new structure model presented in Fig 7. To identify nucleotides interacting with Gag, hSHAPE was conducted on wild type RNA (SA35; Fig 10A) in the presence of Pr77^Gag^ and spliced *env* RNA (AK29; Fig 10B) as a competitor. The differences in hSHAPE reactivity obtained in the absence and in the presence of Pr77^Gag^ were quantified and mapped onto this secondary structure model. Differences were deemed significant when hSHAPE reactivities showed a variance of ≥0.20 and a relative difference exceeding 40% to 50% [81]. hSHAPE reactivities in the presence of Pr77^Gag^ consistently showed reduced reactivity in nucleotides within single-stranded purines (ssPurines) and the primer binding site (PBS), confirming Pr77^Gag^ binding to these sites, as reported earlier (Figs 10C and 11A; [53]).

Footprinting experiments conducted on SP101*i* revealed protections of most nucleotides within ssPurines and the PBS that have been shown to be the primary Gag-binding sites in the WT RNA (Figs 11B and S4). This is consistent with the mutant’s ability to package RNA (Fig 2). In contrast, minimal or no protections were observed in ssPurines and PBS regions in the RNA SP102*i* (only 1 nucleotide (135C) in the PBS was protected). Surprisingly, in this mutant, other nucleotides (26G, 30G, 32U, 35U, 44G, 52G) were protected by Pr77^Gag^ (Figs 11C and S5). The lack of protection in ssPurines and the PBS, which are primary Gag-binding sites [53], aligns with the non-packaging nature of SP102 (Fig 2). However, given the packaging defect of this mutant, the binding of Pr77^Gag^ to other nucleotides was rather puzzling.

**Fig 12:**
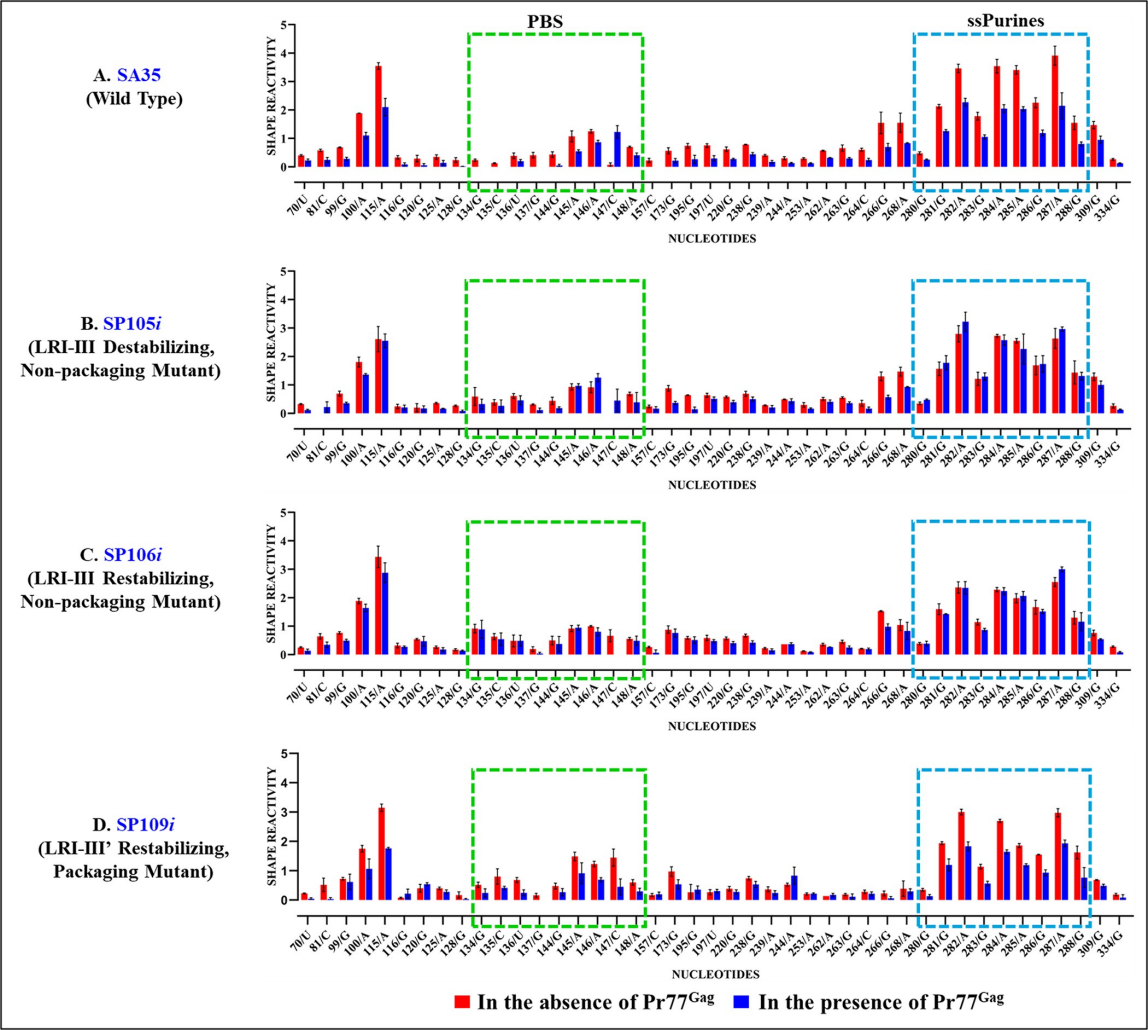
Footprints of Pr77^Gag^ on the WT and LRI-III mutant packaging signal RNAs. Histograms showing the SHAPE reactivities of nucleotides in the absence (red bars) and presence (blue bars) of Gag for the packaging signal RNAs of: **(A)** wild type (SA35), **(B)** mutant SP105*i* designed to destabilize LRI-III, **(C)** mutant SP106*i* designed to re-stabilize LRI-III, and **(D)** mutant SP109*i* designed to restore the LRI-III’. The dashed boxes depict the already identified primary Gag-binding sites, such as single stranded purines (ssPurines) and the PBS. For full details of nucleotides attenuation of SHAPE reactivities in the absence and presence of Pr77^Gag^ from a minimum of 3 independent experiments, see S3 Table and S6, S7 and S8 Figs. All nucleotides that show a reactivity decrease >40% upon Gag addition, also show statistically significant difference according to the Mann–Whitney non parametrical U test (*p* < 0.05). The data underlying this figure can be found in S1 Data. LRI, long-range interaction; PBS, primer binding site; WT, wild type.

In the case of LRI-III, neither SP105*i* (destabilizing mutant) nor SP106*i* (restoring mutant) showed any Pr77^Gag^ footprint within ssPurines (S6 and S7 Figs and Fig 12B and 12C). In the PBS, only 1 (137G) or 2 nucleotides (137G and 147C) were protected by Pr77^Gag^ in SP105*i* and SP106*i*, respectively (S6 and S7 Figs and Fig 12B and 12C). The absence of footprints in the ssPurines and PBS of mutants SP105*i* and SP106*i* corroborates with their inability to package gRNA and the loss of LRIs I-IV in their secondary structure (Figs 2 and 5).

By contrast, mutant SP109, designed to restore LRI-III’ based on the new RNA secondary structure model and which successfully restored both RNA packaging and structure (Fig 8), showed Pr77^Gag^ footprints primarily within ssPurines and the PBS, similar to the wild type (Figs 12D and S8). These results further confirm the new hSHAPE-validated RNA secondary structure model for MMTV *Psi* and stress the importance of Pr77^Gag^ binding to the ssPurines and the PBS for efficient RNA packaging.

**Fig 13:**
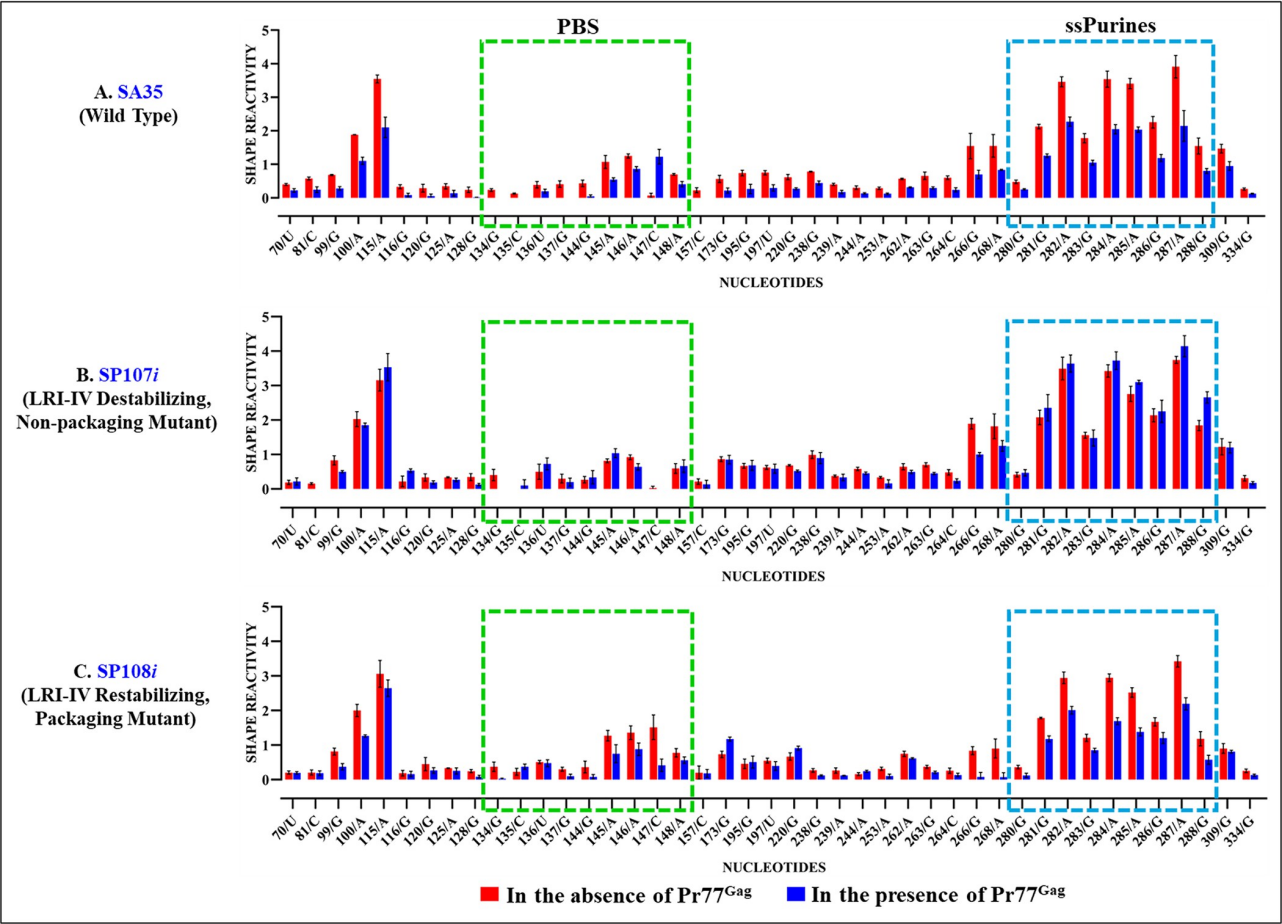
Footprints of Pr77^Gag^ on WT and LRI-IV mutant packaging signal RNAs. Histograms showing SHAPE reactivities of nucleotides in the absence (red bars) and presence (blue bars) of Gag for the packaging signal RNAs of: **(A)** wild type (SA35), **(B)** mutant SP107*i* designed to destabilize LRI-IV, and **(C)** mutant SP108*i* designed to restore LRI-IV. The dashed boxes depict the already identified primary Gag-binding sites such as single stranded purines (ssPurines) and the PBS. For full details of nucleotides attenuation of SHAPE reactivities in the absence and presence of Pr77^Gag^ from a minimum of 3 independent experiments, see S3 Table and S9 and S10 Figs. All nucleotides that show a reactivity decrease >40% upon Gag addition, also show statistically significant difference according to the Mann–Whitney non parametrical U test (*p* < 0.05). The data underlying this figure can be found in S1 Data. LRI, long-range interaction; PBS, primer binding site; WT, wild type.

This result was confirmed by footprinting experiments on mutants SP107*i* and SP108*i*. In brief, the destabilizing mutant (SP107*i*), in which LRI-IV is disrupted, showed no footprint within ssPurines, and only 1 nucleotide was protected by Pr77^Gag^ in the PBS (134G; Figs 13B and S9). Conversely, mutant SP108*i*, which restores LRI-IV, exhibited protections for a majority of the nucleotides in the ssPurines (6/9) and in the PBS (4/7) (Figs 13C and S10). These results indicate that when LRI-IV is restored, Pr77^Gag^ can bind to the ssPurines and the PBS and promote efficient RNA packaging (Fig 2).

Overall, footprinting experiments conducted on all mutants revealed that efficient RNA packaging correlates with strong Pr77^Gag^ binding to nucleotides in ssPurines and the PBS, known as primary Gag-binding sites during WT MMTV gRNA packaging [53]. In stark contrast, mutants that failed to package RNA efficiently revealed Pr77^Gag^ binding to nucleotides scattered in regions other than ssPurines and the PBS. Hence, while Gag binding during retroviral RNA packaging is essential, successful RNA packaging relies on binding to specific nucleotides that can only take place within the appropriate structural context.

## Discussion

RNA viruses rely on conserved structural information found in noncoding and occasionally coding sequences to execute crucial events during their life cycle. The 5′ end of the genome in numerous RNA viruses, including crucial pathogens for humans, animals, and plants, harbors a wealth of *cis*-acting information conveyed through diverse higher-order structures, often maintained by LRIs [82,83].

Notably, LRIs have been identified as conserved structural motifs in various retroviruses [9,10,36,43,45,47–54,84]. Despite significant sequence and structural heterogeneities, the persistence of LRIs in isolates of HIV-1, HIV-2, MPMV, MMTV, and FIV provides evidence for their functional importance in the retroviral life cycle [9,10,36,43,47,48,52–54]. Indeed, mutations destabilizing the complementarity of these LRIs adversely affect crucial steps in the retroviral life cycle, including RNA packaging and dimerization [10,47,48,52,54].

The propensity of retroviral *Psi* to fold into complex secondary structures underscores their crucial role during retroviral gRNA packaging. In most retroviruses, including lentiviruses, the efficiency and selectivity of gRNA packaging in part is governed by the multimerization of the Gag precursor at the plasma membrane [32,85–88], complicating the analysis of this process. MMTV constitutes an attractive system to study membrane-independent retroviral RNA packaging, as it assembles in the cytoplasm of infected cells [59,60]. Within the 5′ end of the MMTV genome, various sequence and structural motifs have been identified as pivotal for gRNA packaging and dimerization ([36,41,53,61]; Fig 1). A distinctive feature of the previously published RNA secondary structure of the MMTV gRNA *Psi* is the presence of several LRIs predicted to anchor the overall secondary RNA structure ([36,41,53]; Fig 1).

The initial goal of this study was to test the role of the 4 proposed LRIs (LRI-I-IV) in MMTV gRNA packaging by combining structural and functional approaches. Among the LRIs that involve only complementary U5 sequences, we were unable to confirm the presence of LRI-II. However, our findings indicate that the proposed LRI-I and LRI-III do not manifest as originally proposed (see Fig 2). On the other hand, our functional data support the existence of LRI-IV, formed by complementary base-pairing of U5/U5 sequences (Fig 2). Our structural data (Figs 4–7) supported these conclusions and demonstrated the existence of an alternative LRI involving U5/U5 complementary sequences (approximately 198 nucleotides apart) instead of U5/Gag as initially proposed ([36,53], compare Fig 1 with Fig 7) that we named as LRI-III’. Interestingly, LRI-III’ and LRI-IV are both important for gRNA packaging (Figs 2 and 8), and our results suggest these LRIs function mechanistically in a similar manner, as their sequences can be substituted with heterologous sequences without having any adverse effects on RNA packaging as long as base pairing is maintained (Figs 2, 6B and 8). Interestingly, the 7 nucleotide LRI-IV in MMTV exhibit functional similarities to the R/U5-Gag heptanucleotide LRI observed in FIV, a lentivirus, since base-pairing, but not sequence, of the FIV LRI is crucial for RNA packaging [10,54].

Of note, the MMTV LRI-III’ and LRI-IV are contiguous in the new secondary structure model of the MMTV *Psi*, being only separated by a one nucleotide bulge (Fig 7). Accordingly, when 2 nucleotides (out of 4) in LRI-III’ or 3 nucleotides (out of 7) in LRI-IV were substituted independently to destabilize the respective LRIs, both LRIs were lost, as well as function (Figs 2, 5A and 6A). Furthermore, in a compensatory approach, when we designed mutants aimed at restoring LRI-III’ and LRI-IV individually (mutants SP109 and SP108, respectively), both function and structure were restored (Figs 2, 6B and 8). The data presented here thus suggest that any perturbation designed to destroy complementarity in this 11 nucleotide long extended LRI severely compromise MMTV gRNA packaging. In light of these observations, we reviewed the structure function analysis of all LRI mutants and observed that the LRI-I destabilizing mutant (SP 101), which did not affect RNA packaging maintained both LRI-III’ and IV as a long continuous stretch as discussed above (Figs 2 and 4A). Taken together, these observations strongly argue that LRIs III’ and IV must be regarded as 1 extended LRI rather than 2 separate LRIs. In this respect, MMTV resembles HIV and FIV, which possess a single identified long-range RNA-RNA interaction [9,10,43,47,48,52,54]. However, unlike other retroviruses such as HIV-1, HIV-2, MPMV, and FIV, where U5/Gag sequences are involved in forming LRIs, [9,10,43,47,48,52,54], the extended MMTV LRI III’-IV only involves U5/U5 sequences (198 nucleotides apart). MPMV, another *betaretrovirus* harbors 2 LRIs that are important for RNA packaging, and in contrast with MMTV, both the structure as well as the sequence of one of these LRIs are important for MPMV gRNA packaging [35,52].

Results from filter-binding experiments demonstrated that both WT and LRI mutants, irrespective of their RNA packaging phenotype, could efficiently bind to Pr77^Gag^ (Fig 9). This observation is in strong contrast with a number of previous studies on several retroviruses, including HIV [44,46], FIV [54], and MMTV [53], in which a clear correlation was observed between binding of the Gag precursor to *Psi* and gRNA packaging efficiency. Our present results thus indicate that while Gag binding to the packaging signal is necessary, it is not sufficient to ensure efficient RNA packaging.

Several previous studies have proposed that high affinity binding of HIV-1 Gag to *Psi*-containing RNAs cannot explain selective packaging of HIV-1 genomic RNA [89–92]. However, a Gag precursor lacking the p6 domain (GagΔp6) was used in all these studies. When a full-length Gag precursor was used, specific binding of Gag to *Psi*-containing RNA was observed [28,44,46] that is consistent with enrichment of HIV-1 genomic RNA in viral particles [93]. In HIV-1 GagΔp6, nonspecific electrostatic interactions overweigh specific interactions [89,92]. It is likely that the p6 domain, which is negatively charged, contributes to the specific binding of full-length Gag to *Psi* by neutralizing the positive charges of the NC domain [28]. Besides, Mutational Interference Mapping Experiment (MIME), an unbiased exhaustive approach, revealed a good correlation between mutations that decreased the binding affinity of full-length HIV-1 Gag for *Psi*-containing RNA [45] with those that decreased packaging of the genomic RNA [26] indicating that, in HIV-1, specific binding of Gag to *Psi* does contribute to packaging. However, some mutations affected RNA packaging without affecting Gag binding [26]. While some of these mutations were found to affect RNA metabolism, thus reducing packaging indirectly, the effect of mutations in the PBS domain remained unexplained. Therefore, it is possible that specific Gag binding to *Psi* is not the only mechanism ensuring selective packaging of the genomic HIV-1 RNA, and kinetics could also affect selectivity of the packaging process [91]. Hence, in the case of MMTV, selection of the gRNA may indeed primarily rely on the kinetic advantage provided by *Psi*.

Indeed, our footprinting experiments (Figs 10–13 and S4–S10) showed that efficient gRNA packaging correlates with Pr77^Gag^ binding in the PBS and ssPurines regions of *Psi*. These results are in line with our previous study that showed that mutations in the PBS and ssPurine regions of MMTV *Psi* dramatically reduced Pr77^Gag^ binding and gRNA packaging [53]. It is also noteworthy that in the case of HIV-1 and many other retroviruses, such as HIV-2, SIV, and MPMV, multiple unpaired purines (single-stranded purines) play a key role in the selective encapsidation of viral RNA [80,94–98]. This points towards a highly conserved role of unpaired purines in selective packaging of retroviral RNAs irrespective of their assembly mode in different parts of the cell. With our non-packaging mutants, we observed scattered footprints even though the local structure of the PBS and ssPurines regions was not altered (Figs 4–6). This suggests that alteration of the overall 3D structure of the *Psi* of the non-packaging mutants, caused by disruption of the extended LRI III’-IV, prevents Pr77^Gag^ binding at these specific sites and promotes binding to nonspecific nucleotides. This indicates that for MMTV gRNA to be selectively packaged, Pr77^Gag^ must bind to specific nucleotides in the correct structural context rather than binding to any nucleotide(s) when the proper structural context is lost, as has been proposed for HIV-1 [99].

Why does efficient Pr77^Gag^ binding to nonspecific nucleotides not promote RNA packaging? RNA is known to be a crucial structural element of retroviral particles [100] and it induces Gag multimerization and Gag assembly [91,101]. Previous experiments suggested that HIV-1 *Psi* more efficiently promotes in vitro Gag/RNA assemblies than heterologous RNAs [91]. Our present study supports a model in which Gag must bind to *Psi* in the correct 3D context to promote efficient assembly of viral particles. Indeed, computer simulations showed that the RNA folding geometry of the packaging signal affects the assembly activation energy barrier, allowing kinetic selectivity of the genomic RNA [102].

## Conclusions

Here, we investigated the relationship between the structure of MMTV gRNA and its ability to interact with Pr77^Gag^. Our study demonstrates that combining genetic, biological, biochemical, and structure–function approaches can provide detailed insights into RNA–RNA and RNA–protein interactions that govern fundamental biological processes during retroviral replication. Specifically, we show that Pr77^Gag^ must bind to specific nucleotides in the correct structural context, which leads to the downstream events of Gag assembly, facilitating selective MMTV gRNA packaging. Furthermore, our work also suggests that Pr77^Gag^ possesses the ability to engage with various RNA conformations with different consequences, suggesting that these mechanistic distinctions could have significant implications for MMTV gRNA packaging. This highlights the importance of a native fold towards identifying well-defined protein-binding sites that promotes function. Thus far, to the best of our knowledge, this is the first demonstration of the intimate connection between retroviral RNA structure, proper Gag binding to specific sites in *Psi*, and efficient encapsidation of the viral RNA in the nascently forming virions.

## Material and methods

### MMTV strain and nucleotide numbers

The MMTV nucleotide numbering system is based on the sequence of HYBMTV, a molecular clone created earlier [103]. This clone was employed for constructing wild-type and mutant transfer vectors used in this study.

### Expression plasmids and transfer vector

JA10 is an MMTV packaging construct that expresses the MMTV *gag-pro-pol* genes ([67]; S1 Fig); the MD.G plasmid serves as an expression vector for the vesicular stomatitis virus glycoprotein G (VSV-G) ([68]; S1 Fig), and the DA024 plasmid ([67]; S1 Fig) is the MMTV sub-genomic transfer vector which functions as a source of packageable RNA. DA024 also contains the *hygromycin B phosphotransferase* gene, which is used to monitor the propagation of the packaged RNA in the transduced cells [67].

### Cloning and mutagenesis

Mutations designed to disrupt or re-establish LRIs were introduced through splice overlap extension (SOE) PCR, utilizing DA024 as the template, using outer sense (S; OTR 249) and antisense (AS; OTR 552) primers (S2 Table). Custom mutation-specific inner primers (S2 Table) were employed along with outer primers in initial 2 separate amplification reactions. Subsequently, the 2 amplified products were combined in a final PCR using outer primers. The final amplified products, flanked by *Spe*I sites, were cleaved and cloned into DA024, previously cleaved with the same restriction enzyme. Mutant clones were confirmed by sequencing.

Mutations in MMTV packaging sequences that were employed for in vivo biological studies were cloned into DA024 [67], which is referred to as the WT and the resultant mutant clones are named as SP101-SP109. For biochemical studies, nucleotides 1–712 of the MMTV gRNA (with nucleotide +1 denoting the start of the R (repeat) region) were PCR-amplified from DA024 or its mutant counterparts, employing already established procedures [53,54]. This amplification utilized primers OTR 984 (S) harboring T7 promoter sequences and a *Hind*III restriction site, and OTR 985 (AS) with a *Xma*I restriction site for T7 promoter insertion at its 5′ end and for the ease of cloning (S2 Table). The resulting amplified products were cloned into pIC19R [104], creating SP101*i*, 102*i*, SP105*i*-SP109*i*. Mutants containing the T7 promoter were also confirmed through sequencing.

### Genetic complementation assay to study single round of MMTV replication

For monitoring the effects of the mutations in the LRIs on gRNA packaging and RNA transduction efficiencies in a biologically relevant assay, we employed a previously established in vivo three-plasmid genetic complementation system ([61,67]; S1 Fig). The MMTV sub-genomic transfer vector (DA024; [67]), harboring the essential *cis*-acting sequences needed for RNA packaging, reverse transcription, and integration, served as the source of RNA that is competent for packaging in virus particles produced by JA10 and pseudotyped by vesicular stomatitis virus envelope glycoprotein (VSV-G) expressed by MD.G [61,67,68]. An additional plasmid DNA (pSEAP2-control vector, Clontech, USA) expressing the *secreted alkaline phosphatase* (*SEAP*) gene was utilized to assess transfection efficiency of each culture. The 4 plasmids were co-transfected into human embryonic kidney (HEK293T) using calcium phosphate transfection kit (Thermo Fisher Scientific, USA). *SEAP* expression was measured employing the Great EscAPe SEAP Chemiluminescence Kit 2.0, (Clontech, USA), as previously described [79,105,106].

Since the replication of the packaged RNA is confined to a single round (S1 Fig), the inclusion of the *hygromycin B phosphotransferase* gene in DA024 allows the quantitative monitoring of propagation of the packaged RNA by counting hygromycin-resistant colonies. Therefore, a portion of the supernatant from the transfected cultures was used to infect human cervical cancer cells (Hela CD4+) in the presence of 1 μg/ml diethylaminoethyl (DEAE)-dextran. This involved selecting the infected cells with cell culture medium containing 200 μg/ml hygromycin B (Hyclone). Cells transduced by the packageable transfer vector RNA expressing the *hygromycin B phosphotransferase* gene were then counted and expressed as colony-forming units (CFUs/ml). The CFU/ml values were normalized to the transfection efficiency of each transfected culture, determined by the relative SEAP values, and divided by the values obtained from the WT DA024 vector to obtain the transduction of mutant RNAs relative to the WT (relative CFU/ml). The number of hygromycin-resistant (Hyg^R^) colonies obtained is expected to be directly proportional to the amount of RNA packaged into the virus particles, providing a good estimate of RNA packaging efficiency.

### Nucleocytoplasmic fractionation of the transfected cells, isolation of cellular and viral RNAs and cDNA synthesis

Transfected cells were fractionated into nuclear and cytoplasmic fractions, following previously established protocols [107]. In summary, virus particles produced from transfected cultures were purified and pelleted through ultracentrifugation, and viral RNA was extracted, treated with TURBO DNase (Invitrogen, Thermo Fisher Scientific) and amplified for 30 cycles employing conventional PCR with established conditions using vector-specific primers OTR 671(S) and OTR 672 (AS) (S2 Table) to test for any residual contaminating plasmid DNA in RNA preparations [36]. In parallel, DNase-treated RNAs were reverse transcribed into cDNAs [53,96,97] and the amplifiability of cDNA preparations was tested through multiplex PCR as described earlier [8,61]. Integrity of the nuclear membrane during fractionation was assessed by multiplex PCR, amplifying cDNAs from the cytoplasmic fraction with oligos OTR 582 (S) and OTR 581 (AS) (S2 Table), specifically targeting unspliced β-actin mRNA. Additionally, as a control for the presence of amplifiable cDNA in multiplex PCRs, cDNAs were amplified using primers/competimer for 18S ribosomal RNA (18S Quantum competimer control, Ambion). Finally, the stability and proper nuclear export of mutant transfer vector RNAs were confirmed by amplifying cytoplasmic cDNA preparations using OTR 671/OTR 672 MMTV-specific primer pair (S2 Table).

### Quantitation of relative RNA packaging efficiency (RPE) using RT-qPCR

To assess the relative levels of transfer vector RNAs in the cytoplasm and in virions, we developed a real-time PCR custom expression assay in a region that was common to all the RNAs and away from the mutation sites, employing minor groove binding (MGB)/FAM chemistry (Applied Biosystems, USA). The Applied Biosystems RT-qPCR assays utilize carboxy-X-rhodamine (ROX) as an internal fluorescence reference dye which is used for normalizing the target reporter dye signal during data analysis. Primers for the PCR were designed to allow annealing to the 101-nucleotide (nt) long region at the beginning of MMTV U5 region shared among all mutant and WT transfer vectors tested. This design enabled their simultaneous relative quantitative assessment using the same probe/primer combination. Detailed information about the probe and primers is provided in S2 Table. In silico validation using the “pcrEfficiency” tool [108] and ABI design pipeline bioinformatic tools confirmed the assay’s specificity, reproducibility, and amplification efficiency. An optimized β-actin MGB FAM labeled assay from ABI (TaqMan 4351368; Applied Biosystems, USA) was employed as an endogenous control.

To standardize the comparative (ΔΔCt) method for relative quantification (RQ) analysis, DNA from plasmids (DA024 and one expressing β-actin) were serially diluted and subjected to non-multiplex RT-qPCRs in triplicates (S2A Fig; panels I and II). The resulting Ct values were then plotted on a scatter plot against the μg amount of plasmid DNA in each dilution (in triplicates) to generate standard curves for both assays (S2B Fig; panels I and II). Subsequently, ΔCt values were calculated (Ct values from the target MMTV assay minus Ct value of the β-actin endogenous control) and plotted against the input plasmid DNA to determine the slope of the curve (S2C Fig).

To quantify gRNA packaged in virus particles, cDNAs derived from viral and cytoplasmic RNAs were tested in triplicates using the Taqman Universal Master Mix (Applied Biosystems) and the 7500 Real Time PCR System (Applied Biosystems, USA). Prior to cDNA preparation, extracted RNA samples were DNase treated and subjected to PCR using vector-specific primers. No detectable amplification could be observed in these DNase-treated RNA samples after 30 cycles of PCR, ensuring lack of any measurable plasmid DNA contamination in these samples, under these conditions. Next, cDNAs were prepared and assessed for vector expression in cytoplasmic fractions and their ability to package into viral particles using the real-time PCR assay under the following cycling conditions: an initial denaturation step of 10 min at 94°C, followed by 40 cycles of denaturation and annealing/extension steps at 95°C for 15 s and 60°C for 1 min. The cytoplasmic RNA expression was normalized to control for transfection efficiency. The determination of the RPE for each mutant transfer vector RNA was achieved by dividing the viral RQ with that of the normalized cytoplasmic RQ and reported relative to that of the WT.

### In vitro transcription and purification of in vitro transcribed RNA

Plasmids carrying the WT (SA35) and mutant MMTV packaging sequences under the control of the T7 promoter were linearized with *Sma*I and utilized for in vitro transcription using MEGAscript T7 Transcription Kit (Thermo Fisher Scientific). The quality of produced RNA was assessed by testing 2 μl of the in vitro transcription reaction on 8% denaturing (8 M urea) polyacrylamide gels. The remaining in vitro transcribed product was precipitated overnight at −20°C using ethanol and the pellets were resuspended in 500 μl of Milli-Q water. Next, these RNAs were purified through gel filtration chromatography using a TSK Gel G4000SW column (Tosoh Bioscience) in 0.2 M sodium acetate (pH 6.5) containing 1% (v/v) methanol, following previously established protocols [109–111]. Subsequently, the appropriate fractions were pooled, ethanol precipitated, and examined for purity and integrity as described above.

Internally labeled RNAs were prepared by conducting in vitro transcription in the presence of (α-^32^P)-ATP, following previously described methods [46,110,111]. The DNase-treated labeled RNAs were purified using Micro Bio-Spin chromatography columns (BioRad), as per manufacturer’s instructions.

### Expression and purification of full-length recombinant MMTV Pr77^Gag^ and western blot analysis

Recombinant full-length MMTV Gag containing hexa-histidine tag at the C-terminus (Pr77^Gag^) was expressed and purified as described previously [79,105,106,112]. The purified Pr77^Gag^ was characterized by western blotting using anti-His_6_ as well as MMTV anti-p27 monoclonal antibodies to confirm purity [79].

### Characterization of Pr77^Gag^ by dynamic light scattering (DLS)

MMTV Pr77^Gag^ was further characterized in the storage buffer (50 mM Tris-HCl (pH 8.0) and 1 M NaCl) by DLS as described earlier [46]. By assimilating the proteins in solution to spheres, their diffusion coefficient was correlated to their hydrodynamic radius (*R*_h_) using the Stokes–Einstein equation:

$$D=\frac{kT}{6\pi \mu Rh}.$$

In this equation, *k* represents the Boltzmann constant, *T* is the absolute temperature, and μ is the viscosity of the solvent. Before acquiring reading, the buffer underwent filtration through 0.2-micron filters (Millex) and buffer solvent offset was measured for subsequent data analysis.

### Filter-binding assay

For filter-binding experiments, 25,000 cpm of internally ^32^P-labeled RNAs were denatured in the presence of 5 nM of the cognate unlabeled RNA and 0.4 μg of yeast tRNA at 90°C for 2 min, following which the samples were chilled on ice for 2 min. The denatured RNAs were incubated at 37°C for 30 min in 1× RNA folding buffer (30 mM Tris-HCl (pH 8), 300 mM NaCl, 5 mM MgCl_2_), along with 5U of Rnasin and 0.01% Triton X-100. Reaction mixtures underwent an additional 30-min incubation with increasing concentrations of protein in 1× protein buffer (30 mM Tris-HCl (pH 8), 300 mM NaCl, 5 mM MgCl_2_, 10 mM DTT, 0.02 mg/ml BSA) to form protein-RNA complexes, which were stabilized by incubating on ice for 30 min.

Filter-binding assays were performed utilizing 0.45 μm nitrocellulose membranes (BioRad) that were pre-wetted with 1× TBS (Tris buffered saline: 20 mM Tris-HCl (pH 7.5) and 500 mM NaCl) for 10 min at room temperature. After drying the membrane on a filter paper, it was placed onto a Bio-Dot Microfiltration unit (BioRad) attached to a vacuum suction drainage. Each well was washed with 100 μl of 1× buffer before applying the protein-RNA complex. Following the addition of 40 μl of cold 1× Gag-binding buffer (30 mM Tris-HCl (pH 8), 300 mM NaCl, 5 mM MgCl_2_), 20 μl of the reaction mixture was applied to each well and incubated at room temperature for 10 min. Following sample filtration, wells were washed 3 times with 100 μl cold 1× Gag-binding buffer to remove any unbound RNA, the nitrocellulose membrane was removed from filtration unit and air-dried. Finally, the membranes were exposed with an imaging plate (Fujifilm), scanned using a FLA 5000 (Fuji) scanner, and quantification was performed using ImageQuant (Cytiva) software, as described earlier [46]. The GraphPad Prism version 8 (v8) software was employed to fit the experimental data with the Hill equation:

$$Y=\frac{B_{max}\times X^{h}}{\left({K_{d}}^{h}+X^{h}\right)}.$$

In this equation, *B_max_* represents the maximum specific binding, *K_d_* represents the equilibrium dissociation constant, and *h* is the Hill slope.

### High-throughput selective 2′-hydroxyl acylation analyzed by primer extension (hSHAPE) and Pr77 ^Gag^ RNA footprints on MMTV gRNA

The structure of RNAs was interrogated using the hSHAPE methodology, enabling a detailed examination of each nucleotide through structure-dependent RNA modification [35,36,46,52–54,72–75,80,96,113]. hSHAPE utilizes a single reagent such as benzoyl cyanide (BzCN), to modify all 4 nucleotides simultaneously, which are more reactive when unpaired. In contrast, the base-paired nucleotides show minimal reactivity to the hSHAPE reagent. To get the Pr77^Gag^ footprint on WT and LRI mutant RNAs, BzCN was used to modify WT RNA and LRI mutant RNAs both in the absence and presence of Pr77^Gag^. Nucleotides displaying reduced reactivity in the presence of Pr77^Gag^ indicated protein’s binding to these nucleotides. This approach has proven successful in identifying Gag-binding sites on the respective gRNAs of HIV-1, MMTV, and MPMV [46,53,80]. Footprinting assays were conducted in the presence of *env* mRNA to mimic the physiological conditions, resulting in obtaining only highly specific Gag-binding sites.

Briefly, for hSHAPE the WT or mutant RNAs were modified in the absence or presence of Pr77^Gag^ and reverse transcription was performed as described previously [53] using VIC-labeled primers (MMTV_WT_VIC and MMTV_329_VIC) (S2 Table). Sequencing reactions using ddGTP were prepared in parallel using NED-labeled primers (MMTV_WT_NED and MMTV_329_NED) (S2 Table) as previously described [53,81]. The resulting cDNAs from both reactions were combined, precipitated, and denatured before analysis using an Applied Biosystems 3130xl genetic analyzer. The obtained electropherograms were then analyzed with the QuShape software [76]. Normalized hSHAPE reactivity data from a minimum of 3 to 4 independent experiments was applied as pseudo-energy constraints for folding the secondary structure of the MMTV packaging signal RNA. The RNA folding was achieved using the RNAstructure version 6.1 program [77], while the VARNA v3-93 software [114] was used to redraw the RNA structures. The hSHAPE reactivity data for each nucleotide was incorporated into the structure as described previously [114]. For RNA footprinting experiments, the hSHAPE reactivities obtained in the presence of 4 μm Pr77^Gag^ was applied onto the RNA structure obtained using reactivity in the absence of Pr77^Gag^. The Mann–Whitney non parametrical U test was conducted to assess statistically significant differences in hSHAPE reactivities in the absence and presence of Pr77^Gag^. Variations in hSHAPE reactivity showing a variance of ≥0.20 and a relative difference exceeding 40% to 50% were deemed significant, as reported earlier for HIV-1, MMTV, and MPMV [46,53,80,81].

### In vitro dimerization assays

Dimerization of 300 nM of the purified WT and LRI mutant RNAs in either a dimer buffer (30 mM Tris (pH 7.5), 300 mM NaCl, 5 mM MgCl_2_) or monomer buffer (30 mM Tris (pH 7.5), 300 mM NaCl, 0.1 mM MgCl_2_) was performed using previously established protocols [35,53]. Following electrophoresis of the samples through a native 1% agarose gel in TBM (50 mM Tris base, 45 mM boric acid, 0.1 mM MgCl_2_) at 4°C, gels were scanned using a Gel Doc EZ Imager (BioRad) and monomeric and dimeric bands were quantified using Image Lab (BioRad) software, as described previously [53,54]. The weight/weight percentage (%) of dimerization efficiency was determined by dividing the intensity of the dimeric RNA band by the sum of the intensities of the dimer and monomer bands. These values were plotted as the percent of dimerization relative to the wild type for each LRI mutation introduced.

### Statistical analysis

We used GraphPad Prism version 8 (v8) software to plot individual data set points. Statistical analysis was performed using the Mann–Whitney non parametrical U test. Results were considered significant when the *p*-value was <0.05.

## Supporting information

S1 Fig Schematic representation of the MMTV three-plasmid genetic complementation assay. The MMTV three-plasmid genetic complementation assay was designed with the following rationale: virus particles generated from the MMTV Gag/Pro/Pol expression plasmid (JA10), pseudotyped with vesicular stomatitis virus envelope glycoprotein (VSV-G) expressed by MD.G, facilitate packaging of MMTV subgenomic transfer vector (DA024) RNA, containing a functional RNA packaging sequences (Ψ). HEK293T cells were co-transfected with these 3 plasmids to produce pseudotyped infectious virus particles capable of only 1 round of replication. Transfected cells were fractionated into nuclear and cytoplasmic fractions to isolate cytoplasmic RNA and analyzed for RNA stability and efficient nuclear export. Viral RNA isolated from the pelleted virus particles was used to quantify the packaged RNA using RT-qPCR. Viral supernatants were used to infect target HeLa CD4+ cells in order to assess the ability of propagation of the encapsidated RNA via selection with media supplemented with hygromycin B antibiotic. This allowed selection of cells transduced by the packaged RNA containing the *hygromycin resistance* gene cassette and appearing as hygromycin resistant colonies.
(PDF)

S2 Fig Validation of the custom made MMTV TaqMan assay for relative quantification of WT and mutant transfer vector RNAs. Estimation of the amplification efficiency of **(A: panel I)** the custom-made MMTV TaqMan assay, **(A: panel II)** the commercially available β-actin TaqMan assay. ΔRn = Normalized Reporter (Rn)—baseline). Standard curves were generated for both **(B: panel I)** MMTV, and **(B: panel II)** β-actin TaqMan assays. **(C)** Relative amplification efficiency plot of MMTV and β-actin TaqMan assays. To ensure similar amplification efficiencies, the slope of log input amount vs. ΔCt should be close to zero (ideally ≤0.1). In our experimental conditions, this slope was calculated to be 0.1076, validating the assay for relative quantification analysis. The data underlying this figure can be found in S1 Data.
(PDF)

S3 Fig Characterization of the recombinant MMTV full-length Pr77^Gag^-His_6_-tag fusion precursor protein. **(A)** Coomassie brilliant blue stained SDS-PAGE of the purest form of the recombinant full-length MMTV Pr77^Gag^-His6-tag fusion protein post-size exclusion chromatography. **(B)** Western blot analysis using α-His_6_ and MMTV α-p27 monoclonal antibodies. **(C, D)** Characterization of the full-length MMTV Pr77^Gag^-His_6_-tag fusion protein conducted via dynamic light scattering (DLS) in binding buffer, showing: protein mass vs. size distribution represented as hydrodynamic radius (Rh) distribution and protein number vs. hydrodynamic radius (Rh) distribution, respectively. The data underlying this S3C and S3D Fig can be found in S1 Data.
(PDF)

S4 Fig Footprints of Pr77^Gag^ on LRI-I destabilizing mutant SP101*i* RNA secondary structure model. hSHAPE analysis was carried out both with and without Pr77^Gag^. The mean triplicate SHAPE reactivity obtained without Pr77^Gag^ was used to predict the RNA secondary structure model. Subsequently, the mean hSHAPE reactivities obtained with Pr77^Gag^ were overlaid onto the RNA secondary structure model predicted in the absence of Pr77^Gag^. Nucleotides marked by arrows show significant reduction in hSHAPE reactivities according to the Mann–Whitney non parametrical U test (*p* < 0.05). The hSHAPE reactivity key was developed based on the mean of hSHAPE reactivities for each nucleotide, as shown in S3 Table. The data shown is from a minimum of 3 independent experiments conducted both in the absence and presence of Pr77^Gag^.
(PDF)

S5 Fig Footprints of Pr77^Gag^ on LRI-I re-stabilizing mutant SP102*i* RNA secondary structure model. hSHAPE analysis was carried out both with and without Pr77^Gag^. The mean triplicate SHAPE reactivity obtained without Pr77^Gag^ was used to predict the RNA secondary structure model. Subsequently, the mean hSHAPE reactivities obtained with Pr77^Gag^ were overlaid onto the RNA secondary structure model predicted in the absence of Pr77^Gag^. Nucleotides marked by arrows show significant reduction in hSHAPE reactivities according to the Mann–Whitney non parametrical U test (*p* < 0.05). The hSHAPE reactivity key was developed based on the mean of hSHAPE reactivities for each nucleotide, as shown in S3 Table. The data shown is from a minimum of 3 independent experiments conducted both in the absence and presence of Pr77^Gag^.
(PDF)

S6 Fig Footprints of Pr77^Gag^ on LRI-III destabilizing mutant SP105*i* RNA secondary structure model. hSHAPE analysis was carried out both with and without Pr77^Gag^. The mean triplicate SHAPE reactivity obtained without Pr77^Gag^ was used to predict the RNA secondary structure model. Subsequently, the mean hSHAPE reactivities obtained with Pr77^Gag^ were overlaid onto the RNA secondary structure model predicted in the absence of Pr77^Gag^. Nucleotides marked by arrows show significant reduction in hSHAPE reactivities according to the Mann–Whitney non parametrical U test (*p* < 0.05). The hSHAPE reactivity key was developed based on the mean of hSHAPE reactivities for each nucleotide, as shown in S3 Table. The data shown is from a minimum of 3 independent experiments conducted both in the absence and presence of Pr77^Gag^.
(PDF)

S7 Fig Footprints of Pr77^Gag^ on LRI-III restabilizing mutant SP106*i* RNA secondary structure model. hSHAPE analysis was carried out both with and without Pr77^Gag^. The mean triplicate SHAPE reactivity obtained without Pr77^Gag^ was used to predict the RNA secondary structure model. Subsequently, the mean hSHAPE reactivities obtained with Pr77^Gag^ were overlaid onto the RNA secondary structure model predicted in the absence of Pr77^Gag^. Nucleotides marked by arrows show significant reduction in hSHAPE reactivities according to the Mann–Whitney non parametrical U test (*p* < 0.05). The hSHAPE reactivity key was developed based on the mean of hSHAPE reactivities for each nucleotide, as shown in S3 Table. The data shown is from a minimum of 3 independent experiments conducted both in the absence and presence of Pr77^Gag^.
(PDF)

S8 Fig Footprints of Pr77^Gag^ on LRI-III’ re-stabilizing mutant SP109*i* RNA secondary structure model. hSHAPE analysis was carried out both with and without Pr77^Gag^. The mean triplicate SHAPE reactivity obtained without Pr77^Gag^ was used to predict the RNA secondary structure model. Subsequently, the mean hSHAPE reactivities obtained with Pr77^Gag^ were overlaid onto the RNA secondary structure model predicted in the absence of Pr77^Gag^. Nucleotides marked by arrows show significant reduction in hSHAPE reactivities according to the Mann–Whitney non parametrical U test (*p* < 0.05). The hSHAPE reactivity key was developed based on the mean of hSHAPE reactivities for each nucleotide, as shown in S3 Table. The data shown is from a minimum of 3 independent experiments conducted both in the absence and presence of Pr77^Gag^.
(PDF)

S9 Fig Footprints of Pr77^Gag^ on LRI-IV destabilizing mutant SP107*i* RNA secondary structure model. hSHAPE analysis was carried out both with and without Pr77^Gag^. The mean triplicate SHAPE reactivity obtained without Pr77^Gag^ was used to predict the RNA secondary structure model. Subsequently, the mean hSHAPE reactivities obtained with Pr77^Gag^ were overlaid onto the RNA secondary structure model predicted in the absence of Pr77^Gag^. Nucleotides marked by arrows show significant reduction in hSHAPE reactivities according to the Mann–Whitney non parametrical U test (*p* < 0.05). The hSHAPE reactivity key was developed based on the mean of hSHAPE reactivities for each nucleotide, as shown in S3 Table. The data shown is from a minimum of 3 independent experiments conducted both in the absence and presence of Pr77^Gag^.
(PDF)

S10 Fig Footprints of Pr77^Gag^ on LRI-IV re-stabilizing mutant SP108*i* RNA secondary structure model. hSHAPE analysis was carried out both with and without Pr77^Gag^. The mean triplicate SHAPE reactivity obtained without Pr77^Gag^ was used to predict the RNA secondary structure model. Subsequently, the mean hSHAPE reactivities obtained with Pr77^Gag^ were overlaid onto the RNA secondary structure model predicted in the absence of Pr77^Gag^. Nucleotides marked by arrows show significant reduction in hSHAPE reactivities according to the Mann–Whitney non parametrical U test (*p* < 0.05). The hSHAPE reactivity key was developed based on the mean of hSHAPE reactivities for each nucleotide, as shown in S3 Table. The data shown is from a minimum of 3 independent experiments conducted both in the absence and presence of Pr77^Gag^.
(PDF)

S1 Table Mean hSHAPE reactivities with standard deviations (SD) of first 432 nucleotides of WT unspliced RNA (SA35) and LRI mutant clones (SP101*i*, SP 102*i*, SP 105*i*-SP 109*i*) from 3 independent experiments. The yellow highlighted region shows mutations introduced in LRI mutants.
(PDF)

S2 Table Primer details used for introduction of mutations, construction of clones, hSHAPE, RT-qPCR, and other amplifications. (PDF)

S3 Table Mean hSHAPE reactivities from 3 experiments in the absence and presence of Pr77^Gag^. The yellow highlighted nucleotides showed ≥1.5-fold reduction of hSHAPE reactivities in the presence of Pr77^Gag^.
(PDF)

S1 Data Excel spreadsheet containing, in separate sheets for each figure, the underlying and individual numerical data used for Figs 2C, 2D, 3D, 8A, 9A, 9B, 11A, 11B, 11C, 12A, 12B, 12C, 12D, 13A, 13B, 13C, S2A–S2C, S3C and S3D. (XLSX)

S1 Raw Images Uncropped gel images used to make panel I of Fig 2B, panels II and III of Figs 2B, 3C, S3A and S3B. (PDF)

## Abbreviations

BzCN: benzoyl cyanide
cDNA: complementary DNA
CFU: colony-forming unit
DIS: dimerization initiation site
DLS: dynamic light scattering
gRNA: genomic RNA
HEK: human embryonic kidney
HIV: human immunodeficiency virus
LRI: long-range interaction
MGB: minor groove binding
MIME: Mutational Interference Mapping Experiment
MMTV: mouse mammary tumor virus
mSD: major splice donor
NC: nucleocapsid
ORF: open reading frame
PBS: primer binding site
RPE: relative packaging efficiency
RQ: relative quantification
RT-qPCR: real-time quantitative PCR
SL: stem loop
SOE: splice overlap extension
TBS: Tris buffered saline
VLP: virus-like particle
VSV-G: vesicular stomatitis virus glycoprotein G
WT: wild type
