# Supplementary material for: MMTV RNA packaging requires an extended long-range interaction for productive Gag binding to packaging signals
Source: PLoS Biol. 2024 Oct 3;22(10):e3002827. doi: 10.1371/journal.pbio.3002827 (PMC11449360; doi:10.1371/journal.pbio.3002827)
Supplement: S1 Fig — The MMTV three-plasmid genetic complementation assay was designed with the following rationale: virus particles generated from the MMTV Gag/Pro/Pol expression plasmid (JA10), pseudotyped with vesicular stomatitis virus envelope glycoprotein (VSV-G) expressed by MD.G, facilitate packaging of MMTV subgenomic transfer vector (DA024) RNA, containing a functional RNA packaging sequences (Ψ). HEK293T cells were co-transfected with these 3 plasmids to produce pseudotyped infectious virus particles capable of only 1 round of replication. Transfected cells were fractionated into nuclear and cytoplasmic fractions to isolate cytoplasmic RNA and analyzed for RNA stability and efficient nuclear export. Viral RNA isolated from the pelleted virus particles was used to quantify the packaged RNA using RT-qPCR. Viral supernatants were used to infect target HeLa CD4+ cells in order to assess the ability of propagation of the encapsidated RNA via selection with media supplemented with hygromycin B antibiotic. This allowed selection of cells transduced by the packaged RNA containing the hygromycin resistance gene cassette and appearing as hygromycin resistant colonies. (PDF) [file pbio.3002827.s001.pdf]

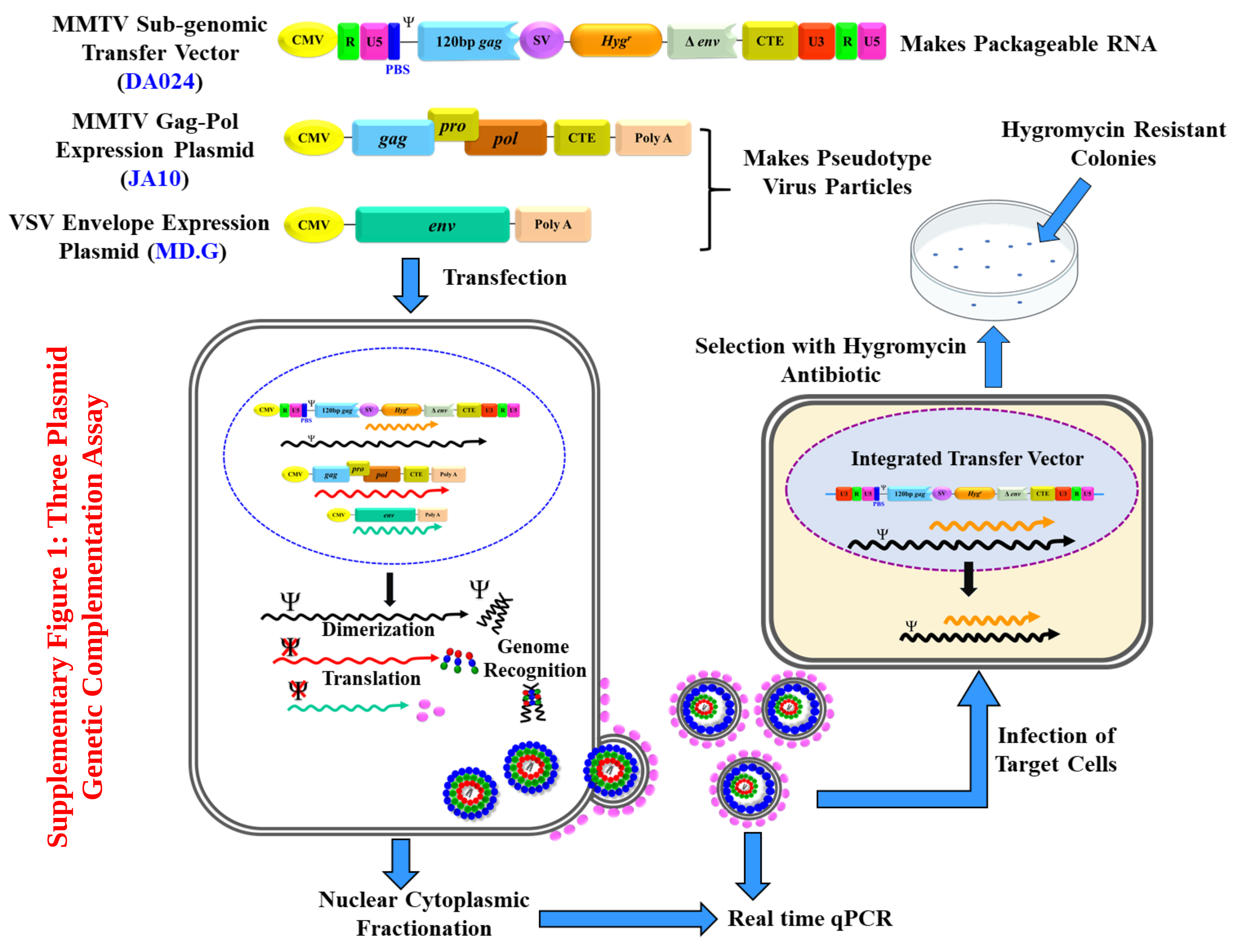


Supplementary Figure 1: Three Plasmid Genetic Complementation Assay
