# Supplementary figures and images for: MMTV RNA packaging requires an extended long-range interaction for productive Gag binding to packaging signals
Source: PLoS Biol. 2024 Oct 3;22(10):e3002827. doi: 10.1371/journal.pbio.3002827 (PMC11449360; doi:10.1371/journal.pbio.3002827)

Supplementary Figure 2: Real Time PCR Related Figures

**A**

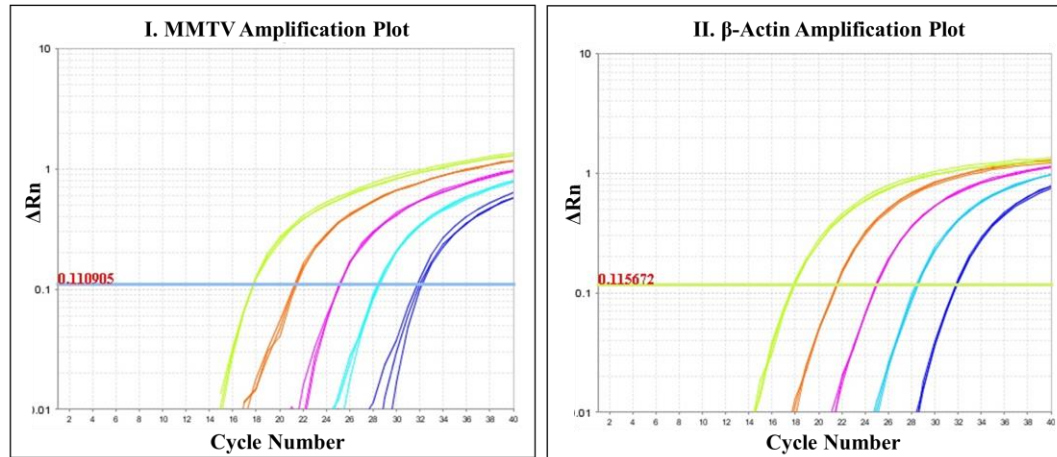

**B**

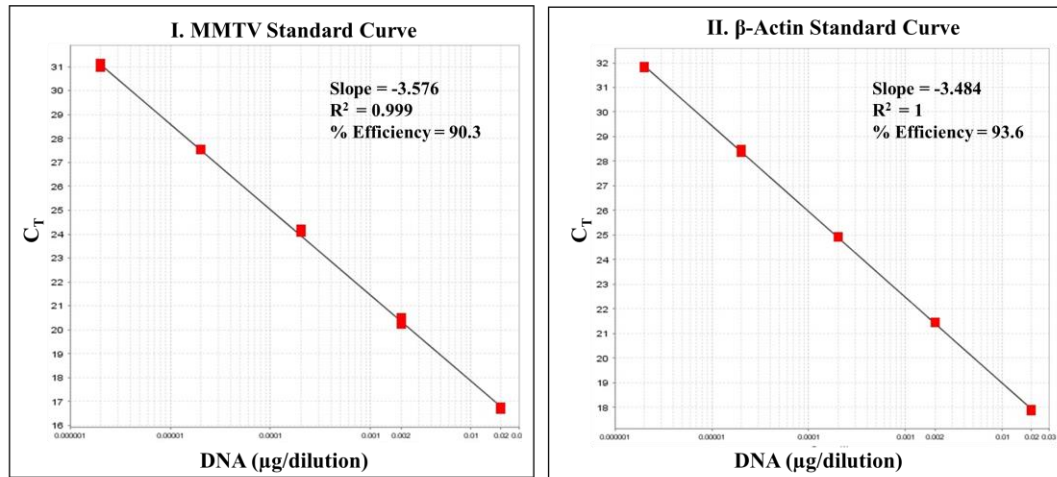

**C**

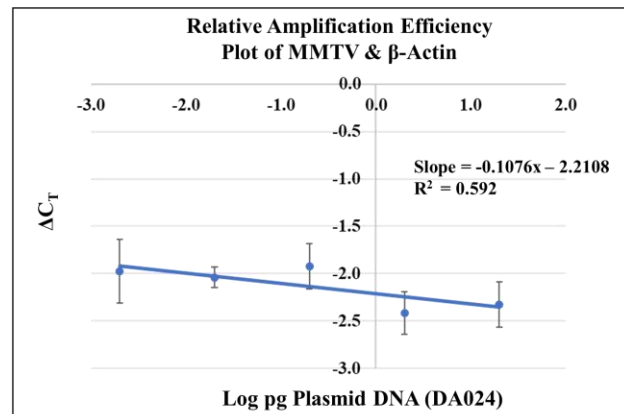

Supplement: S2 Fig — Estimation of the amplification efficiency of (A: panel I) the custom-made MMTV TaqMan assay, (A: panel II) the commercially available β-actin TaqMan assay. ΔRn = Normalized Reporter (Rn)—baseline). Standard curves were generated for both (B: panel I) MMTV, and (B: panel II) β-actin TaqMan assays. (C) Relative amplification efficiency plot of MMTV and β-actin TaqMan assays. To ensure similar amplification efficiencies, the slope of log input amount vs. ΔCt should be close to zero (ideally ≤0.1). In our experimental conditions, this slope was calculated to be 0.1076, validating the assay for relative quantification analysis. The data underlying this figure can be found in S1 Data. (PDF) [file pbio.3002827.s002.pdf]

### Supplementary Figure 3: Coomassie, Western Blots, DLS

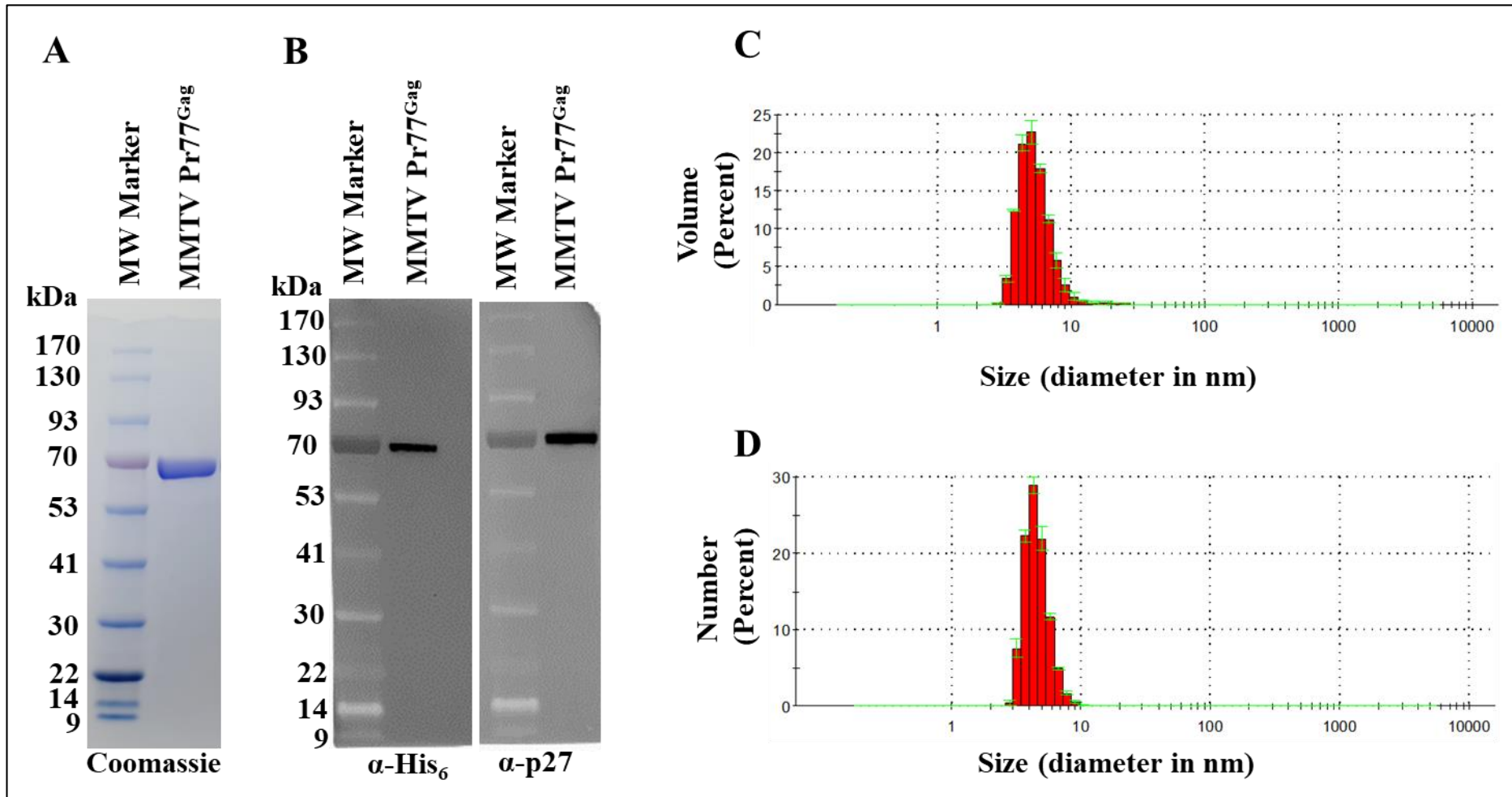

Supplement: S3 Fig — (A) Coomassie brilliant blue stained SDS-PAGE of the purest form of the recombinant full-length MMTV Pr77Gag-His6-tag fusion protein post-size exclusion chromatography. (B) Western blot analysis using α-His6 and MMTV α-p27 monoclonal antibodies. (C, D) Characterization of the full-length MMTV Pr77Gag-His6-tag fusion protein conducted via dynamic light scattering (DLS) in binding buffer, showing: protein mass vs. size distribution represented as hydrodynamic radius (Rh) distribution and protein number vs. hydrodynamic radius (Rh) distribution, respectively. The data underlying this S3C and S3D Fig can be found in S1 Data. (PDF) [file pbio.3002827.s003.pdf]

**Supplementary Figure 4: SP101i Footprinting**

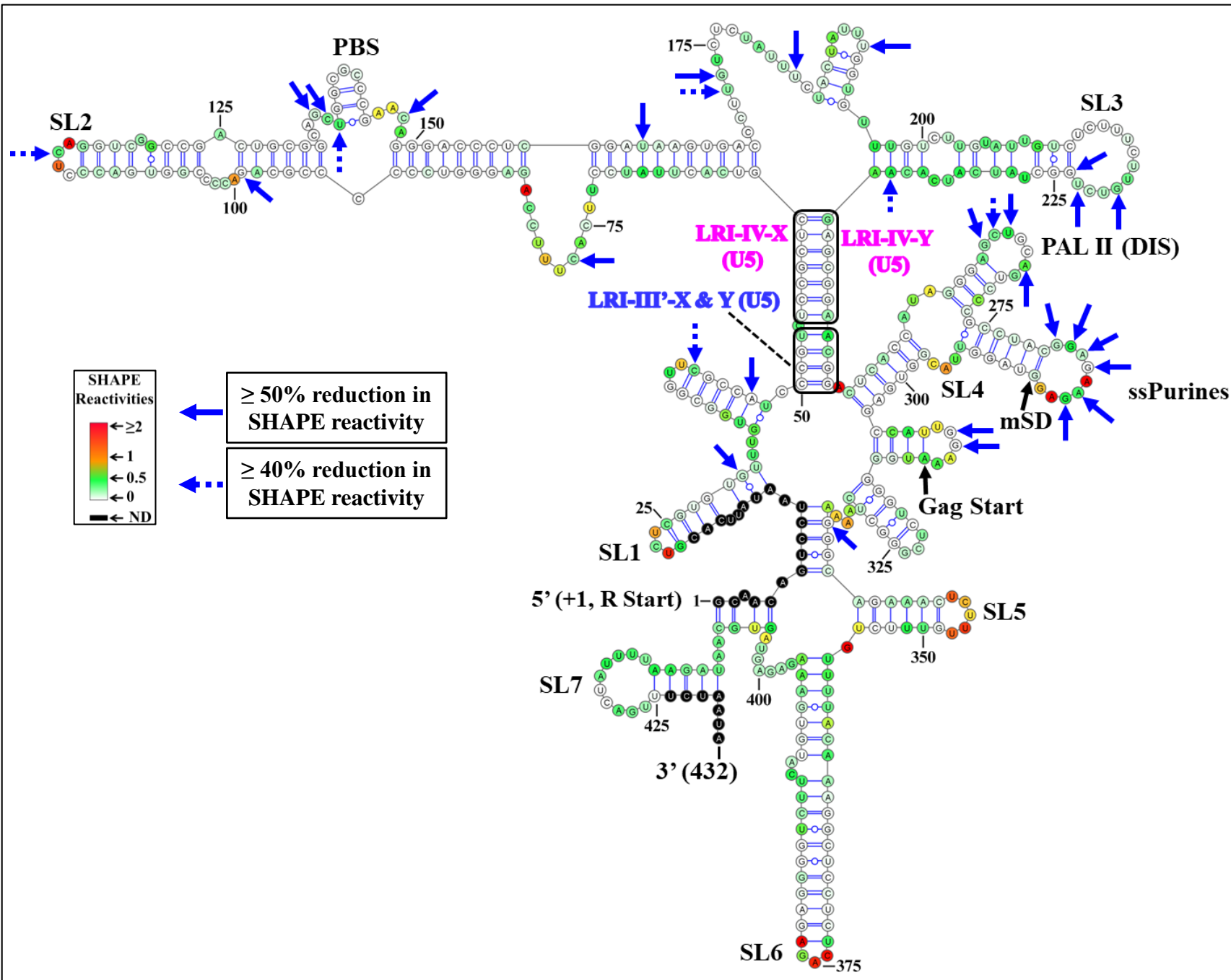

Supplement: S4 Fig — hSHAPE analysis was carried out both with and without Pr77Gag. The mean triplicate SHAPE reactivity obtained without Pr77Gag was used to predict the RNA secondary structure model. Subsequently, the mean hSHAPE reactivities obtained with Pr77Gag were overlaid onto the RNA secondary structure model predicted in the absence of Pr77Gag. Nucleotides marked by arrows show significant reduction in hSHAPE reactivities according to the Mann–Whitney non parametrical U test (p < 0.05). The hSHAPE reactivity key was developed based on the mean of hSHAPE reactivities for each nucleotide, as shown in S3 Table. The data shown is from a minimum of 3 independent experiments conducted both in the absence and presence of Pr77Gag. (PDF) [file pbio.3002827.s004.pdf]

## Supplementary Figure 5: SP102i Footprinting

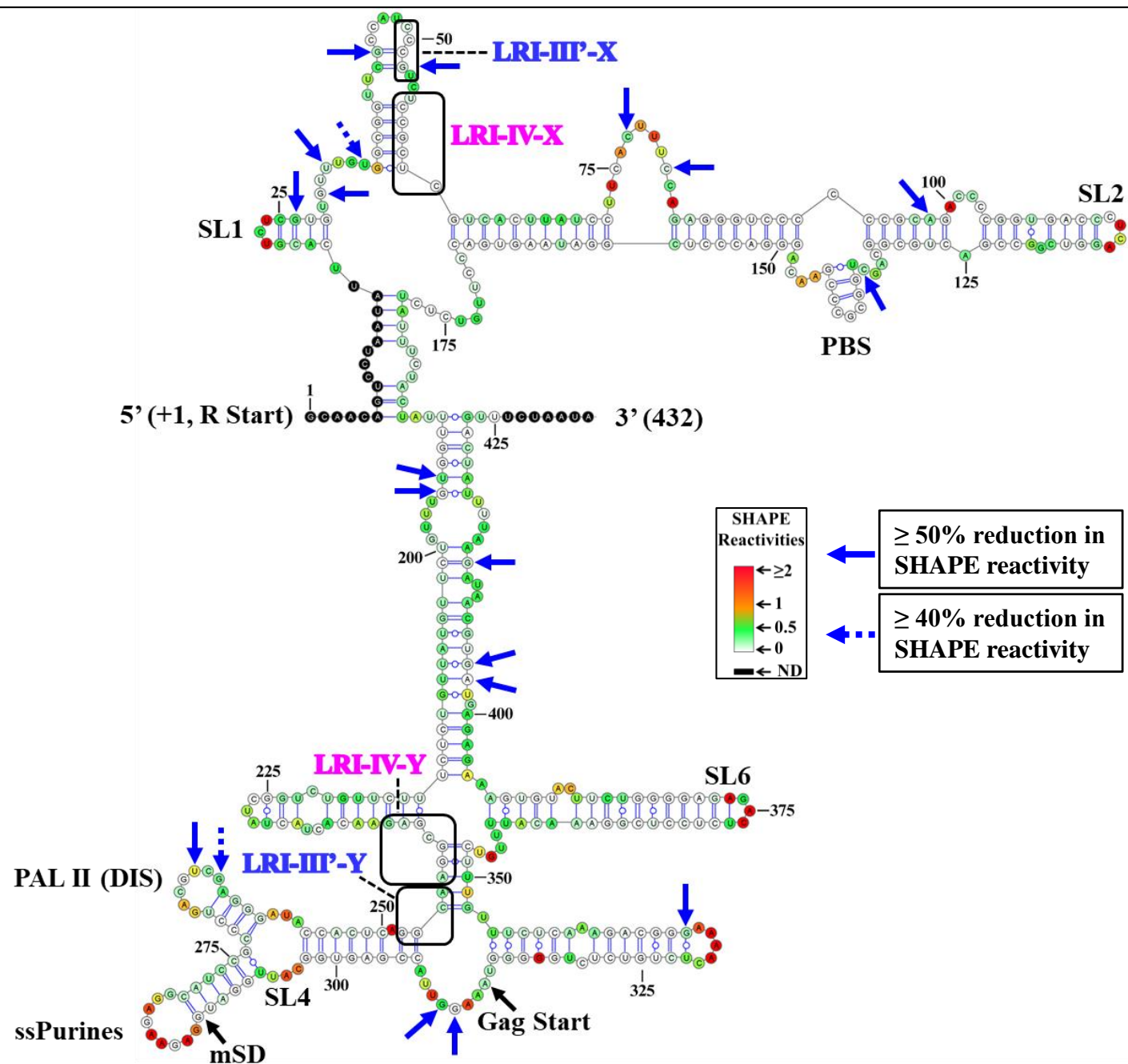

Supplement: S5 Fig — hSHAPE analysis was carried out both with and without Pr77Gag. The mean triplicate SHAPE reactivity obtained without Pr77Gag was used to predict the RNA secondary structure model. Subsequently, the mean hSHAPE reactivities obtained with Pr77Gag were overlaid onto the RNA secondary structure model predicted in the absence of Pr77Gag. Nucleotides marked by arrows show significant reduction in hSHAPE reactivities according to the Mann–Whitney non parametrical U test (p < 0.05). The hSHAPE reactivity key was developed based on the mean of hSHAPE reactivities for each nucleotide, as shown in S3 Table. The data shown is from a minimum of 3 independent experiments conducted both in the absence and presence of Pr77Gag. (PDF) [file pbio.3002827.s005.pdf]

**Supplementary Figure 6: SP105i Footprinting**

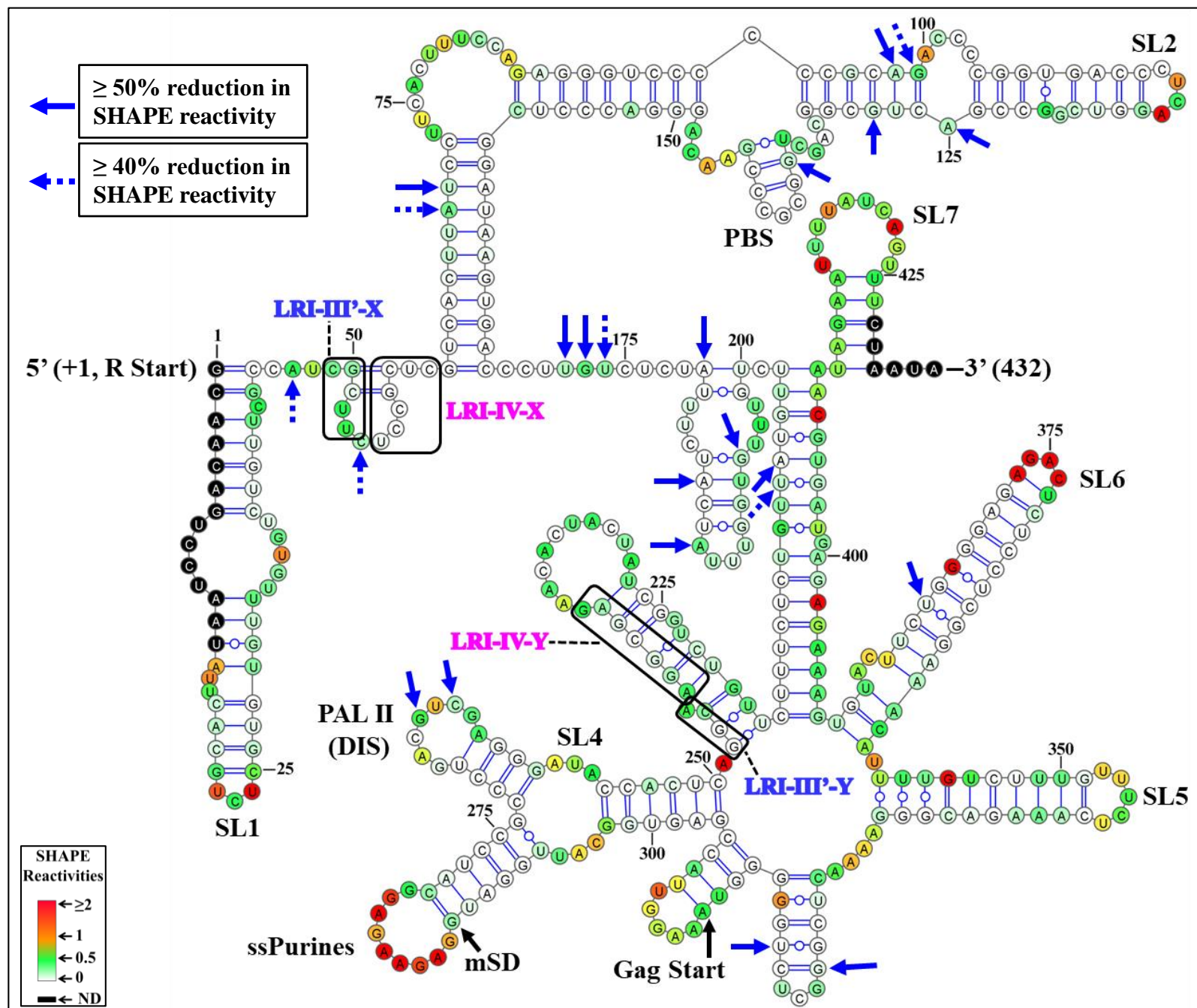

Supplement: S6 Fig — hSHAPE analysis was carried out both with and without Pr77Gag. The mean triplicate SHAPE reactivity obtained without Pr77Gag was used to predict the RNA secondary structure model. Subsequently, the mean hSHAPE reactivities obtained with Pr77Gag were overlaid onto the RNA secondary structure model predicted in the absence of Pr77Gag. Nucleotides marked by arrows show significant reduction in hSHAPE reactivities according to the Mann–Whitney non parametrical U test (p < 0.05). The hSHAPE reactivity key was developed based on the mean of hSHAPE reactivities for each nucleotide, as shown in S3 Table. The data shown is from a minimum of 3 independent experiments conducted both in the absence and presence of Pr77Gag. (PDF) [file pbio.3002827.s006.pdf]

**Supplementary Figure 7: SP106i Footprinting**

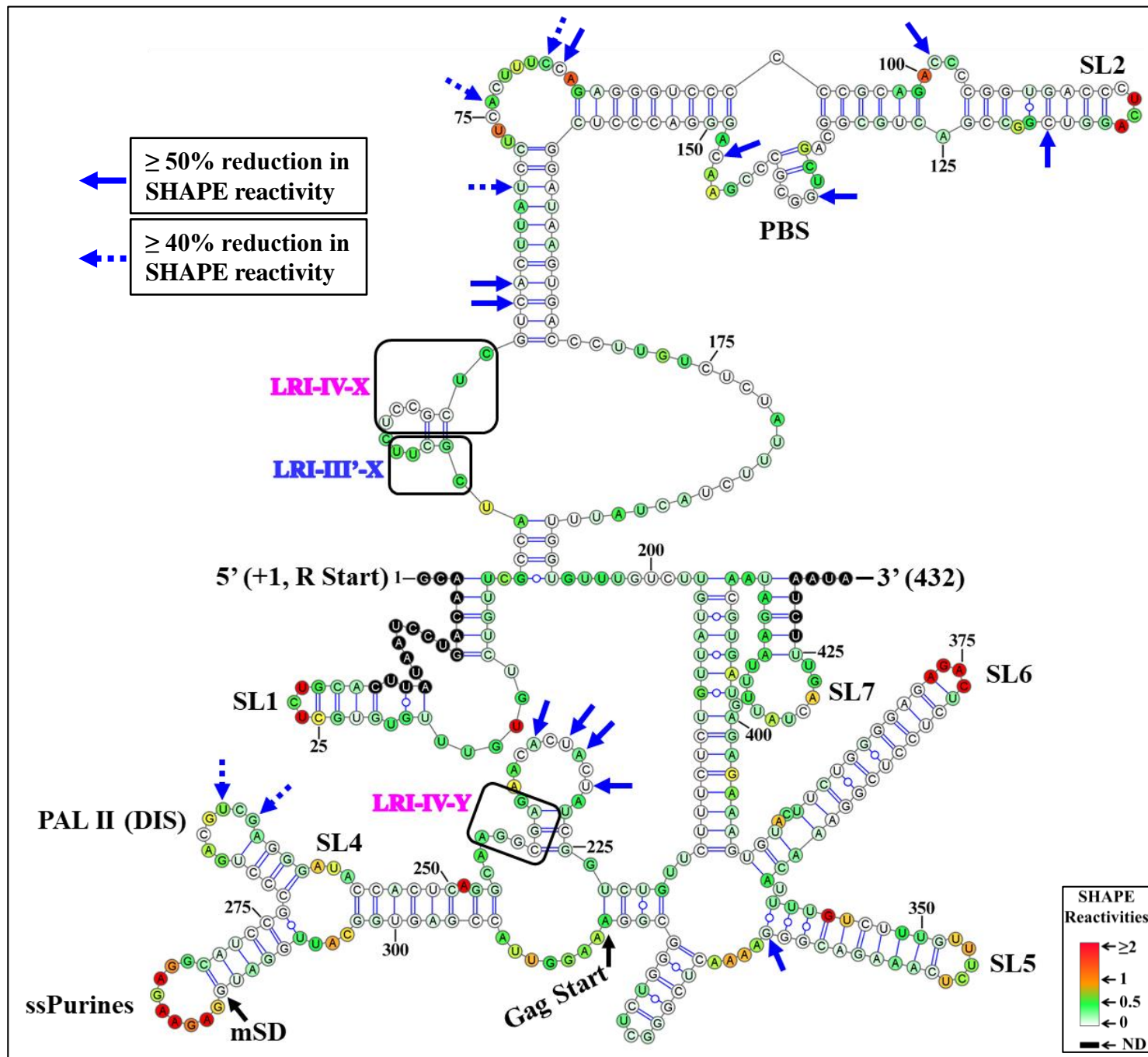

Supplement: S7 Fig — hSHAPE analysis was carried out both with and without Pr77Gag. The mean triplicate SHAPE reactivity obtained without Pr77Gag was used to predict the RNA secondary structure model. Subsequently, the mean hSHAPE reactivities obtained with Pr77Gag were overlaid onto the RNA secondary structure model predicted in the absence of Pr77Gag. Nucleotides marked by arrows show significant reduction in hSHAPE reactivities according to the Mann–Whitney non parametrical U test (p < 0.05). The hSHAPE reactivity key was developed based on the mean of hSHAPE reactivities for each nucleotide, as shown in S3 Table. The data shown is from a minimum of 3 independent experiments conducted both in the absence and presence of Pr77Gag. (PDF) [file pbio.3002827.s007.pdf]

**Supplementary Figure 8: SP109i Footprinting**

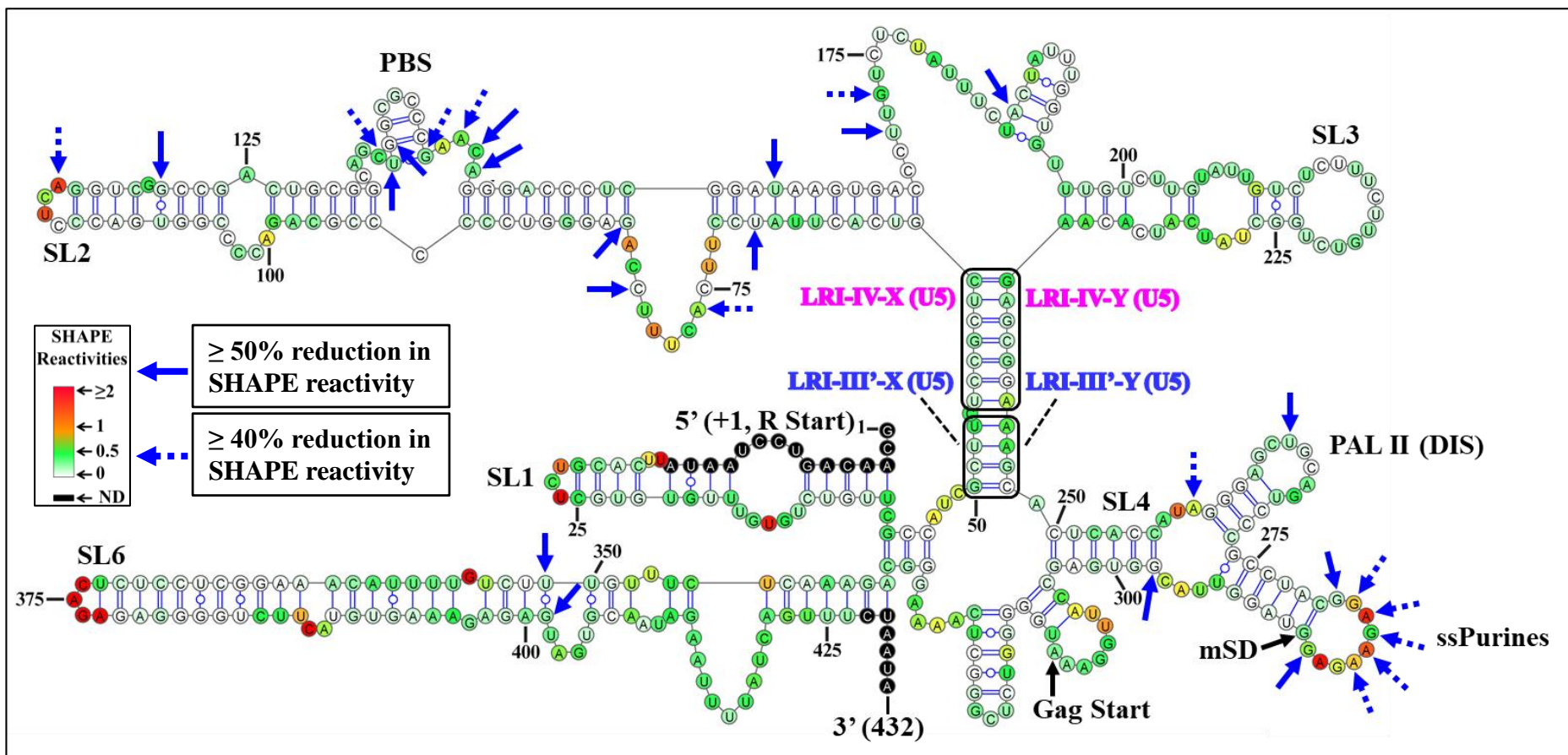

Supplement: S8 Fig — hSHAPE analysis was carried out both with and without Pr77Gag. The mean triplicate SHAPE reactivity obtained without Pr77Gag was used to predict the RNA secondary structure model. Subsequently, the mean hSHAPE reactivities obtained with Pr77Gag were overlaid onto the RNA secondary structure model predicted in the absence of Pr77Gag. Nucleotides marked by arrows show significant reduction in hSHAPE reactivities according to the Mann–Whitney non parametrical U test (p < 0.05). The hSHAPE reactivity key was developed based on the mean of hSHAPE reactivities for each nucleotide, as shown in S3 Table. The data shown is from a minimum of 3 independent experiments conducted both in the absence and presence of Pr77Gag. (PDF) [file pbio.3002827.s008.pdf]

Supplementary Figure 9: SP107i Footprinting

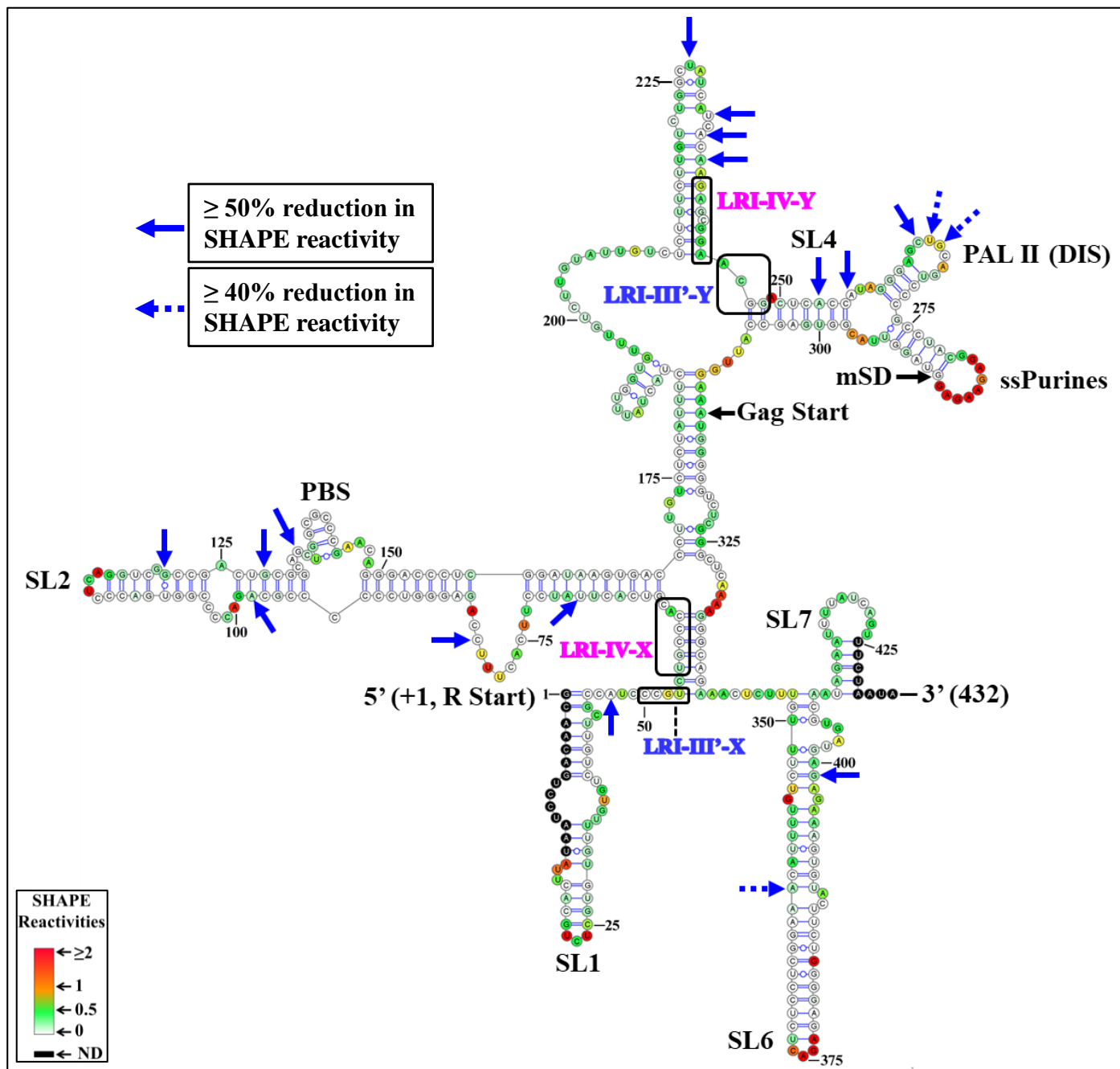

Supplement: S9 Fig — hSHAPE analysis was carried out both with and without Pr77Gag. The mean triplicate SHAPE reactivity obtained without Pr77Gag was used to predict the RNA secondary structure model. Subsequently, the mean hSHAPE reactivities obtained with Pr77Gag were overlaid onto the RNA secondary structure model predicted in the absence of Pr77Gag. Nucleotides marked by arrows show significant reduction in hSHAPE reactivities according to the Mann–Whitney non parametrical U test (p < 0.05). The hSHAPE reactivity key was developed based on the mean of hSHAPE reactivities for each nucleotide, as shown in S3 Table. The data shown is from a minimum of 3 independent experiments conducted both in the absence and presence of Pr77Gag. (PDF) [file pbio.3002827.s009.pdf]

**Supplementary Figure 10: SP108i Footprinting**

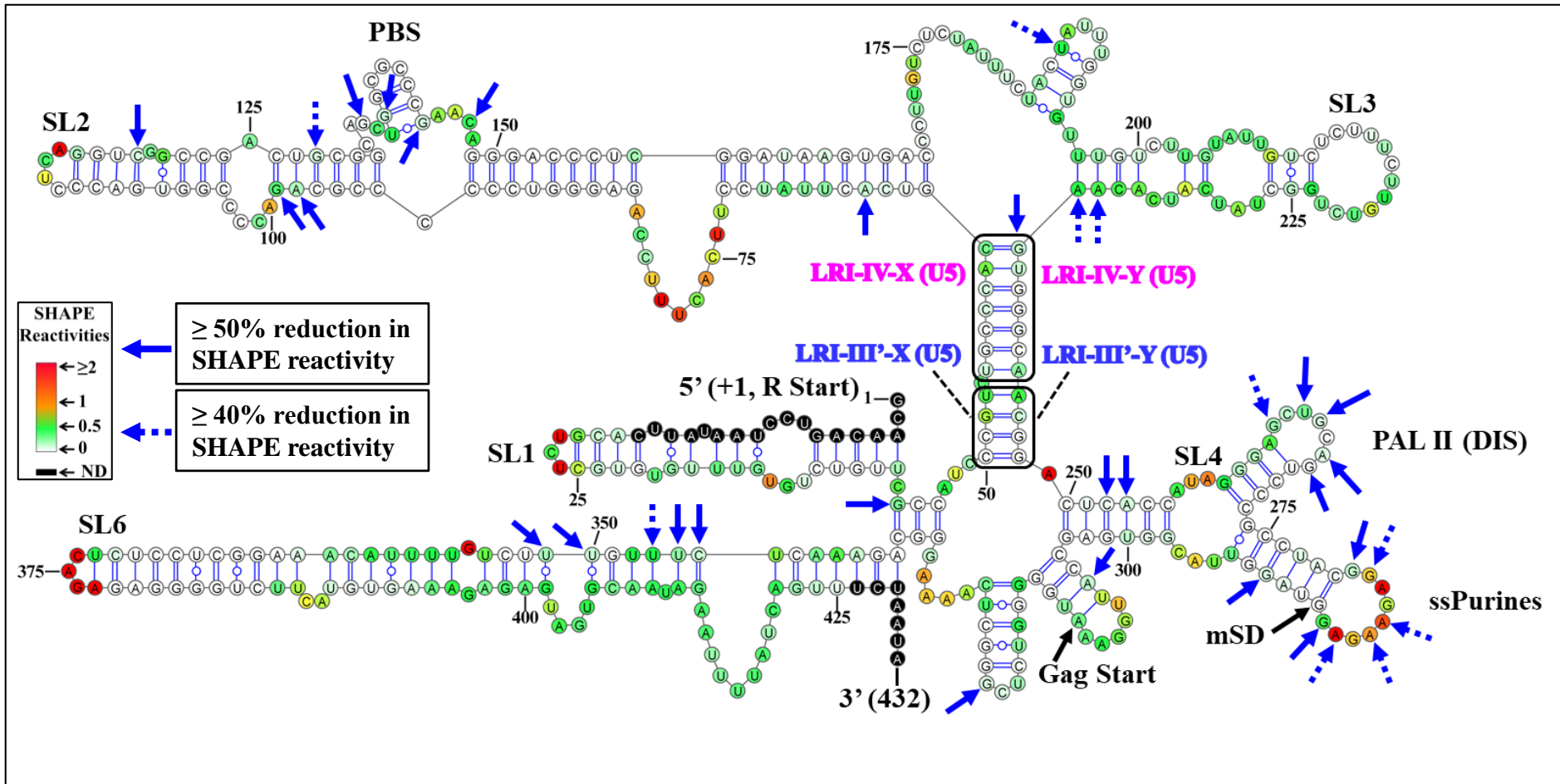

Supplement: S10 Fig — hSHAPE analysis was carried out both with and without Pr77Gag. The mean triplicate SHAPE reactivity obtained without Pr77Gag was used to predict the RNA secondary structure model. Subsequently, the mean hSHAPE reactivities obtained with Pr77Gag were overlaid onto the RNA secondary structure model predicted in the absence of Pr77Gag. Nucleotides marked by arrows show significant reduction in hSHAPE reactivities according to the Mann–Whitney non parametrical U test (p < 0.05). The hSHAPE reactivity key was developed based on the mean of hSHAPE reactivities for each nucleotide, as shown in S3 Table. The data shown is from a minimum of 3 independent experiments conducted both in the absence and presence of Pr77Gag. (PDF) [file pbio.3002827.s010.pdf]
