# Supplementary material for: MMTV RNA packaging requires an extended long-range interaction for productive Gag binding to packaging signals
Source: PLoS Biol. 2024 Oct 3;22(10):e3002827. doi: 10.1371/journal.pbio.3002827 (PMC11449360; doi:10.1371/journal.pbio.3002827)
Supplement: S1 Table — The yellow highlighted region shows mutations introduced in LRI mutants. (PDF) [file pbio.3002827.s011.pdf]

Supplementary Table 1: Mean hSHAPE reactivities with standard deviations (SD) of first 432 nucleotides of wild type unspliced RNA (SA35) and LRI mutant clones (SP101*i* , SP 102*i* , SP 105*i* - SP 109*i*) from three independent experiments. The yellow highlighted region shows mutations introduced in LRI mutants.

hSHAPE Reactivities from 3 independent experiments (SA35-Wild Type)						
Nucleotides		Experiment 1	Experiment 2	Experiment 3	Mean	SD
Number	Sequence					
1	G	-999	-999	-999	-999	0.000
2	C	-999	-999	-999	-999	0.000
3	A	-999	-999	-999	-999	0.000
4	A	-999	-999	-999	-999	0.000
5	C	-999	-999	-999	-999	0.000
6	A	-999	-999	-999	-999	0.000
7	G	-999	-999	-999	-999	0.000
8	U	-999	-999	-999	-999	0.000
9	C	-999	-999	-999	-999	0.000
10	C	-999	-999	-999	-999	0.000
11	U	-999	-999	-999	-999	0.000
12	A	-999	-999	-999	-999	0.000
13	A	-999	-999	-999	-999	0.000
14	U	-999	-999	-999	-999	0.000
15	A	-999	-999	-999	-999	0.000
16	U	-999	-999	-999	-999	0.000
17	U	-999	-999	-999	-999	0.000
18	C	-999	-999	-999	-999	0.000
19	A	-999	-999	-999	-999	0.000
20	C	0.130	0.170	0.160	0.153	0.021
21	G	0.880	1.080	0.690	0.883	0.195
22	U	2.850	3.200	3.330	3.127	0.248
23	C	0.620	0.760	0.810	0.730	0.098
24	U	3.560	4.160	3.890	3.870	0.300
25	C	0.800	0.840	1.290	0.977	0.272
26	G	0.270	0.100	0.380	0.250	0.141
27	U	0.090	0.060	0.220	0.123	0.085
28	G	0.000	0.000	0.000	0.000	0.000
29	U	0.180	0.020	0.100	0.100	0.080
30	G	0.140	0.160	0.390	0.230	0.139
31	U	0.080	0.125	0.100	0.102	0.023
32	U	0.460	0.400	0.310	0.390	0.075
33	U	0.285	0.280	0.390	0.318	0.062
34	G	0.225	0.160	0.255	0.213	0.049
35	U	2.005	1.740	1.915	1.887	0.135
36	G	0.350	0.275	0.355	0.327	0.045
37	U	0.090	0.075	0.135	0.100	0.031
38	C	0.090	0.120	0.170	0.127	0.040
39	U	0.030	0.000	0.240	0.090	0.131
40	G	0.000	0.000	0.000	0.000	0.000
41	U	0.000	0.000	0.000	0.000	0.000
42	U	0.430	0.395	0.230	0.352	0.107
43	C	0.515	0.430	0.425	0.457	0.051
44	G	0.185	0.000	0.070	0.085	0.093
45	C	0.000	0.000	0.000	0.000	0.000
46	C	0.000	0.000	0.000	0.000	0.000
47	A	1.800	1.450	1.980	1.743	0.270
48	U	1.040	1.000	0.950	0.997	0.045

hSHAPE Reactivities from 3 independent experiments (SA35-Wild Type)						
Nucleotides		Experiment 1	Experiment 2	Experiment 3	Mean	SD
Number	Sequence					
49	C	0.220	0.370	0.000	0.197	0.186
50	C	0.000	0.000	0.000	0.000	0.000
51	C	0.000	0.000	0.000	0.000	0.000
52	G	0.210	0.310	0.000	0.173	0.158
53	U	0.480	0.390	0.390	0.420	0.052
54	C	0.530	0.425	0.345	0.433	0.093
55	U	0.210	0.050	0.200	0.153	0.090
56	C	0.000	0.070	0.000	0.023	0.040
57	C	0.000	0.000	0.000	0.000	0.000
58	G	0.000	0.000	0.000	0.000	0.000
59	C	0.020	0.000	0.050	0.023	0.025
60	U	0.250	0.110	0.250	0.203	0.081
61	C	0.400	0.250	0.250	0.300	0.087
62	G	0.100	0.020	0.000	0.040	0.053
63	U	0.350	0.365	0.300	0.338	0.034
64	C	1.610	3.750	1.120	2.160	1.399
65	A	0.485	0.610	0.440	0.512	0.088
66	C	0.035	0.075	0.240	0.117	0.109
67	U	0.540	0.520	0.510	0.523	0.015
68	U	1.345	1.720	0.870	1.312	0.426
69	A	1.000	1.110	1.060	1.057	0.055
70	U	0.670	0.460	0.405	0.512	0.140
71	C	0.115	0.605	0.050	0.257	0.303
72	C	0.150	0.240	0.000	0.130	0.121
73	U	0.940	0.655	0.880	0.825	0.150
74	U	2.480	1.150	1.190	1.607	0.757
75	C	0.230	0.070	0.060	0.120	0.095
76	A	1.775	1.460	2.095	1.777	0.318
77	C	0.660	0.360	0.695	0.572	0.184
78	U	1.715	1.440	2.010	1.722	0.285
79	U	1.565	1.760	2.345	1.890	0.406
80	U	0.980	0.795	1.000	0.925	0.113
81	C	0.085	0.170	0.080	0.112	0.051
82	C	0.655	0.995	0.140	0.597	0.430
83	A	1.980	2.370	2.670	2.340	0.346
84	G	1.530	1.910	0.100	1.180	0.954
85	A	0.020	0.070	0.155	0.082	0.068
86	G	0.000	0.000	0.000	0.000	0.000
87	G	0.000	0.000	0.000	0.000	0.000
88	G	0.000	0.000	0.000	0.000	0.000
89	U	0.000	0.000	0.000	0.000	0.000
90	C	0.000	0.030	0.000	0.010	0.017
91	C	0.000	0.000	0.105	0.035	0.061
92	C	0.220	0.110	0.535	0.288	0.221
93	C	0.000	0.000	0.000	0.000	0.000
94	C	0.000	0.000	0.000	0.000	0.000
95	C	0.000	0.050	0.160	0.070	0.082
96	G	0.000	0.085	0.035	0.040	0.043

hSHAPE Reactivities from 3 independent experiments (SA35-Wild Type)						
Nucleotides		Experiment 1	Experiment 2	Experiment 3	Mean	SD
Number	Sequence					
97	C	0.000	0.000	0.000	0.000	0.000
98	A	0.300	0.430	0.650	0.460	0.177
99	G	0.710	0.675	0.910	0.765	0.127
100	A	2.060	1.790	1.990	1.947	0.140
101	C	0.030	0.150	0.030	0.070	0.069
102	C	0.185	0.280	0.000	0.155	0.142
103	C	0.000	0.000	0.000	0.000	0.000
104	C	0.000	0.000	0.000	0.000	0.000
105	G	0.005	0.040	0.005	0.017	0.020
106	G	0.035	0.060	0.040	0.045	0.013
107	U	0.240	0.160	0.160	0.187	0.046
108	G	0.010	0.020	0.005	0.012	0.008
109	A	0.000	0.015	0.000	0.005	0.009
110	C	0.000	0.000	0.000	0.000	0.000
111	C	0.265	0.185	0.210	0.220	0.041
112	C	1.070	0.475	0.810	0.785	0.298
113	U	3.415	3.075	3.750	3.413	0.338
114	C	1.460	1.380	1.100	1.313	0.189
115	A	3.110	4.020	4.650	3.927	0.774
116	G	0.215	0.270	0.350	0.278	0.068
117	G	0.060	0.050	0.100	0.070	0.026
118	U	0.010	0.080	0.010	0.033	0.040
119	C	0.525	0.250	0.235	0.337	0.163
120	G	0.335	0.395	0.485	0.405	0.075
121	G	0.805	0.645	0.675	0.708	0.085
122	C	0.000	0.000	0.000	0.000	0.000
123	C	0.000	0.000	0.000	0.000	0.000
124	G	0.220	0.180	0.235	0.212	0.028
125	A	0.290	0.345	0.410	0.348	0.060
126	C	0.060	0.065	0.095	0.073	0.019
127	U	0.175	0.085	0.175	0.145	0.052
128	G	0.225	0.235	0.250	0.237	0.013
129	C	0.090	0.090	0.070	0.083	0.012
130	G	0.000	0.000	0.000	0.000	0.000
131	G	0.000	0.000	0.000	0.000	0.000
132	C	0.000	0.075	0.000	0.025	0.043
133	A	0.210	0.750	0.240	0.400	0.303
134	G	0.260	0.250	0.220	0.243	0.021
135	C	1.335	1.155	1.425	1.305	0.137
136	U	0.790	0.690	0.965	0.815	0.139
137	G	0.310	0.500	0.485	0.432	0.106
138	G	0.535	0.670	0.805	0.670	0.135
139	C	0.180	0.235	0.465	0.293	0.151
140	G	0.160	0.185	0.220	0.188	0.030
141	C	0.000	0.000	0.000	0.000	0.000
142	C	0.000	0.000	0.000	0.000	0.000
143	C	0.375	0.390	0.040	0.268	0.198
144	G	0.690	1.000	0.920	0.870	0.161

hSHAPE Reactivities from 3 independent experiments (SA35-Wild Type)						
Nucleotides		Experiment 1	Experiment 2	Experiment 3	Mean	SD
Number	Sequence					
145	A	1.330	1.500	1.460	1.430	0.089
146	A	1.115	1.425	0.990	1.177	0.224
147	C	0.000	0.000	0.000	0.000	0.000
148	A	0.585	0.705	0.530	0.607	0.089
149	G	0.015	0.000	0.000	0.005	0.009
150	G	0.000	0.000	0.000	0.000	0.000
151	G	0.000	0.000	0.000	0.000	0.000
152	A	0.000	0.000	0.000	0.000	0.000
153	C	0.000	0.000	0.000	0.000	0.000
154	C	0.000	0.000	0.000	0.000	0.000
155	C	0.390	0.390	0.230	0.337	0.092
156	U	0.135	0.125	0.070	0.110	0.035
157	C	0.340	0.320	0.145	0.268	0.107
158	G	0.000	0.000	0.000	0.000	0.000
159	G	0.000	0.000	0.055	0.018	0.032
160	A	0.035	0.000	0.100	0.045	0.051
161	U	0.585	0.465	0.880	0.643	0.214
162	A	0.050	0.000	0.000	0.017	0.029
163	A	0.030	0.000	0.060	0.030	0.030
164	G	0.000	0.000	0.000	0.000	0.000
165	U	0.000	0.000	0.000	0.000	0.000
166	G	0.000	0.000	0.000	0.000	0.000
167	A	0.000	0.000	0.000	0.000	0.000
168	C	0.000	0.000	0.000	0.000	0.000
169	C	0.140	0.275	0.030	0.148	0.123
170	C	0.000	0.150	0.000	0.050	0.087
171	U	0.030	0.145	0.530	0.235	0.262
172	U	0.340	0.330	0.530	0.400	0.113
173	G	0.570	0.555	0.730	0.618	0.097
174	U	0.385	0.370	0.620	0.458	0.140
175	C	0.060	0.070	0.120	0.083	0.032
176	U	0.000	0.000	0.000	0.000	0.000
177	C	0.000	0.000	0.000	0.000	0.000
178	U	0.200	0.210	0.000	0.137	0.118
179	A	0.395	0.285	0.355	0.345	0.056
180	U	0.075	0.045	0.110	0.077	0.033
181	U	0.090	0.000	0.040	0.043	0.045
182	U	0.110	0.070	0.060	0.080	0.026
183	C	0.000	0.000	0.240	0.080	0.139
184	U	0.150	0.055	0.580	0.262	0.280
185	A	0.070	0.110	0.110	0.097	0.023
186	C	0.060	0.040	0.080	0.060	0.020
187	U	0.830	0.780	0.170	0.593	0.367
188	A	0.590	0.545	0.460	0.532	0.066
189	U	0.110	0.030	0.260	0.133	0.117
190	U	0.150	0.060	0.170	0.127	0.059
191	U	0.250	0.000	0.510	0.253	0.255
192	G	0.110	0.040	0.440	0.197	0.214

hSHAPE Reactivities from 3 independent experiments (SA35-Wild Type)						
Nucleotides		Experiment 1	Experiment 2	Experiment 3	Mean	SD
Number	Sequence					
193	G	0.320	0.360	0.530	0.403	0.112
194	U	0.555	0.430	0.535	0.507	0.067
195	G	0.590	0.420	0.840	0.617	0.211
196	U	0.465	0.340	0.625	0.477	0.143
197	U	0.720	0.830	0.690	0.747	0.074
198	U	0.310	0.290	0.350	0.317	0.031
199	G	0.000	0.000	0.000	0.000	0.000
200	U	0.000	0.000	0.000	0.000	0.000
201	C	0.060	0.000	0.130	0.063	0.065
202	U	0.090	0.020	0.140	0.083	0.060
203	U	0.175	0.060	0.010	0.082	0.085
204	G	0.160	0.050	0.010	0.073	0.078
205	U	0.535	0.575	0.010	0.373	0.315
206	A	0.180	0.175	0.140	0.165	0.022
207	U	0.130	0.060	0.090	0.093	0.035
208	U	0.480	0.175	0.190	0.282	0.172
209	G	0.655	0.455	0.590	0.567	0.102
210	U	0.080	0.140	0.165	0.128	0.044
211	C	0.000	0.000	0.000	0.000	0.000
212	U	0.000	0.000	0.000	0.000	0.000
213	C	0.000	0.000	0.000	0.000	0.000
214	U	0.000	0.000	0.000	0.000	0.000
215	U	0.050	0.035	0.105	0.063	0.037
216	U	0.110	0.050	0.000	0.053	0.055
217	C	0.005	0.000	0.000	0.002	0.003
218	U	0.170	0.130	0.260	0.187	0.067
219	U	0.370	0.275	0.210	0.285	0.080
220	G	0.435	0.385	0.330	0.383	0.053
221	U	0.195	0.230	0.090	0.172	0.073
222	C	0.075	0.205	0.130	0.137	0.065
223	U	0.315	0.500	0.210	0.342	0.147
224	G	0.120	0.520	0.340	0.327	0.200
225	G	0.000	0.000	0.000	0.000	0.000
226	C	0.000	0.000	0.000	0.000	0.000
227	U	1.070	1.240	0.590	0.967	0.337
228	A	0.615	0.505	0.495	0.538	0.067
229	U	0.355	0.300	0.140	0.265	0.112
230	C	0.170	0.885	0.000	0.352	0.470
231	A	1.210	0.770	1.125	1.035	0.233
232	U	0.000	0.000	0.000	0.000	0.000
233	C	0.000	0.945	0.000	0.315	0.546
234	A	1.060	1.000	0.780	0.947	0.147
235	C	0.000	0.175	0.000	0.058	0.101
236	A	0.840	1.150	0.940	0.977	0.158
237	A	1.510	1.580	1.430	1.507	0.075
238	G	0.625	0.785	0.625	0.678	0.092
239	A	0.345	0.400	0.485	0.410	0.071
240	G	0.055	0.055	0.005	0.038	0.029

hSHAPE Reactivities from 3 independent experiments (SA35-Wild Type)						
Nucleotides		Experiment 1	Experiment 2	Experiment 3	Mean	SD
Number	Sequence					
241	C	0.195	0.155	0.060	0.137	0.069
242	G	0.460	0.355	0.435	0.417	0.055
243	G	0.455	0.370	0.510	0.445	0.071
244	A	0.750	0.530	0.700	0.660	0.115
245	A	0.355	0.335	0.715	0.468	0.214
246	C	0.000	0.000	0.000	0.000	0.000
247	G	0.000	0.000	0.000	0.000	0.000
248	G	0.000	0.000	0.000	0.000	0.000
249	A	2.170	2.110	2.260	2.180	0.075
250	C	0.320	0.170	0.130	0.207	0.100
251	U	0.000	0.040	0.130	0.057	0.067
252	C	0.070	0.115	0.115	0.100	0.026
253	A	0.030	0.260	0.110	0.133	0.117
254	C	0.000	0.000	0.000	0.000	0.000
255	C	0.300	0.340	0.060	0.233	0.151
256	A	3.360	2.190	2.280	2.610	0.651
257	U	1.305	1.130	1.230	1.222	0.088
258	A	1.155	1.475	0.990	1.207	0.247
259	G	0.095	0.110	0.060	0.088	0.026
260	G	0.060	0.215	0.020	0.098	0.103
261	G	0.120	0.205	0.135	0.153	0.045
262	A	0.515	0.635	0.570	0.573	0.060
263	G	0.415	0.410	0.385	0.403	0.016
264	C	0.600	0.185	0.260	0.348	0.221
265	U	1.245	1.055	1.210	1.170	0.101
266	G	1.005	1.105	0.910	1.007	0.098
267	C	0.295	0.200	0.000	0.165	0.151
268	A	0.755	1.270	1.880	1.302	0.563
269	G	1.010	1.100	0.865	0.992	0.119
270	U	0.180	0.180	0.185	0.182	0.003
271	C	0.010	0.000	0.000	0.003	0.006
272	C	0.000	0.000	0.000	0.000	0.000
273	C	0.000	0.000	0.000	0.000	0.000
274	G	0.820	0.420	0.560	0.600	0.203
275	C	0.245	0.560	0.580	0.462	0.188
276	C	0.410	0.245	0.230	0.295	0.100
277	U	0.130	0.260	0.000	0.130	0.130
278	A	0.080	0.245	0.070	0.132	0.098
279	C	0.290	0.360	0.155	0.268	0.104
280	G	0.465	0.240	0.390	0.365	0.115
281	G	1.710	1.905	1.740	1.785	0.105
282	A	2.435	1.970	2.615	2.340	0.333
283	G	0.895	0.570	0.740	0.735	0.163
284	A	2.805	2.410	2.885	2.700	0.254
285	A	1.930	1.600	1.790	1.773	0.166
286	G	1.455	2.150	1.140	1.582	0.517
287	A	2.860	1.970	2.540	2.457	0.451
288	G	0.640	0.530	0.600	0.590	0.056

hSHAPE Reactivities from 3 independent experiments (SA35-Wild Type)						
Nucleotides		Experiment 1	Experiment 2	Experiment 3	Mean	SD
Number	Sequence					
289	G	0.000	0.000	0.040	0.013	0.023
290	U	0.000	0.110	0.325	0.145	0.165
291	A	0.000	0.000	0.000	0.000	0.000
292	G	0.010	0.000	0.000	0.003	0.006
293	G	0.180	0.010	0.000	0.063	0.101
294	U	0.680	0.410	1.385	0.825	0.503
295	U	0.450	0.705	0.550	0.568	0.128
296	A	0.665	0.815	0.640	0.707	0.095
297	C	0.615	0.615	0.630	0.620	0.009
298	G	0.020	0.000	0.060	0.027	0.031
299	G	0.070	0.010	0.000	0.027	0.038
300	U	0.120	0.140	0.000	0.087	0.076
301	G	0.010	0.150	0.000	0.053	0.084
302	A	0.070	0.040	0.000	0.037	0.035
303	G	0.000	0.000	0.000	0.000	0.000
304	C	0.030	0.280	0.000	0.103	0.154
305	C	0.140	0.510	0.000	0.217	0.264
306	A	1.290	0.750	0.420	0.820	0.439
307	U	0.980	0.750	0.790	0.840	0.123
308	U	1.440	1.120	1.310	1.290	0.161
309	G	0.950	1.000	1.070	1.007	0.060
310	G	0.790	1.010	1.150	0.983	0.181
311	A	0.820	1.000	1.100	0.973	0.142
312	A	0.350	0.470	0.460	0.427	0.067
313	A	0.330	0.460	0.230	0.340	0.115
314	U	0.400	0.000	0.000	0.133	0.231
315	G	0.020	0.000	0.000	0.007	0.012
316	G	0.000	0.000	0.000	0.000	0.000
317	G	0.000	0.000	0.000	0.000	0.000
318	G	0.000	0.000	0.000	0.000	0.000
319	G	0.440	0.030	0.150	0.207	0.211
320	U	0.250	0.150	0.220	0.207	0.051
321	C	0.030	0.000	0.010	0.013	0.015
322	U	0.200	0.140	0.130	0.157	0.038
323	C	0.050	0.090	0.010	0.050	0.040
324	G	0.380	0.260	0.310	0.317	0.060
325	G	0.410	0.490	0.450	0.450	0.040
326	G	0.000	0.000	0.000	0.000	0.000
327	C	0.000	0.020	0.000	0.007	0.012
328	U	0.260	0.620	0.040	0.307	0.293
329	C	0.000	1.070	0.000	0.357	0.618
330	A	1.320	0.970	0.540	0.943	0.391
331	A	0.990	0.740	0.950	0.893	0.134
332	A	0.940	0.710	0.980	0.877	0.146
333	A	0.860	0.780	1.100	0.913	0.167
334	G	0.230	0.220	0.290	0.247	0.038
335	G	0.030	0.060	0.050	0.047	0.015
336	G	0.080	0.040	0.020	0.047	0.031

hSHAPE Reactivities from 3 independent experiments (SA35-Wild Type)						
Nucleotides		Experiment 1	Experiment 2	Experiment 3	Mean	SD
Number	Sequence					
337	C	0.180	0.130	0.000	0.103	0.093
338	A	0.120	0.190	0.000	0.103	0.096
339	G	0.000	0.020	0.000	0.007	0.012
340	A	0.170	0.180	0.070	0.140	0.061
341	A	0.400	0.250	0.160	0.270	0.121
342	A	0.040	0.150	0.040	0.077	0.064
343	C	0.000	0.000	0.000	0.000	0.000
344	U	1.150	1.000	0.980	1.043	0.093
345	C	0.490	0.690	0.630	0.603	0.103
346	U	0.820	1.170	1.340	1.110	0.265
347	U	0.860	1.290	1.410	1.187	0.289
348	U	0.860	1.080	1.200	1.047	0.172
349	G	0.080	0.180	0.080	0.113	0.058
350	U	0.540	0.630	0.480	0.550	0.075
351	U	0.250	0.260	0.280	0.263	0.015
352	U	0.000	0.060	0.040	0.033	0.031
353	C	0.000	0.000	0.000	0.000	0.000
354	U	1.260	0.520	0.370	0.717	0.476
355	G	2.050	1.200	1.480	1.577	0.433
356	U	0.400	0.570	0.510	0.493	0.086
357	U	0.300	0.590	0.690	0.527	0.203
358	U	0.250	0.510	0.480	0.413	0.142
359	U	0.230	0.500	0.400	0.377	0.137
360	A	0.560	0.990	0.810	0.787	0.216
361	C	0.000	1.250	0.000	0.417	0.722
362	A	0.250	0.460	0.380	0.363	0.106
363	A	0.140	0.220	0.180	0.180	0.040
364	A	0.030	0.100	0.100	0.077	0.040
365	G	0.000	0.000	0.000	0.000	0.000
366	G	0.000	0.000	0.000	0.000	0.000
367	C	0.030	0.000	0.050	0.027	0.025
368	U	0.000	0.000	0.000	0.000	0.000
369	C	0.000	0.000	0.000	0.000	0.000
370	C	0.110	0.040	0.000	0.050	0.056
371	U	0.620	0.510	0.290	0.473	0.168
372	C	0.160	0.260	0.140	0.187	0.064
373	U	1.470	1.100	1.030	1.200	0.236
374	C	2.500	1.430	1.960	1.963	0.535
375	A	3.480	2.680	2.500	2.887	0.522
376	G	1.510	1.020	1.600	1.377	0.312
377	A	1.120	1.080	1.260	1.153	0.095
378	G	0.020	0.080	0.000	0.033	0.042
379	A	0.000	0.000	0.000	0.000	0.000
380	G	0.000	0.000	0.000	0.000	0.000
381	G	0.000	0.000	0.000	0.000	0.000
382	G	0.000	0.000	0.000	0.000	0.000
383	G	0.000	0.000	0.000	0.000	0.000
384	U	0.000	0.060	0.000	0.020	0.035

hSHAPE Reactivities from 3 independent experiments (SA35-Wild Type)						
Nucleotides		Experiment 1	Experiment 2	Experiment 3	Mean	SD
Number	Sequence					
385	C	0.000	0.000	0.000	0.000	0.000
386	U	0.050	0.340	0.120	0.170	0.151
387	U	0.490	0.900	0.230	0.540	0.338
388	C	0.000	1.170	0.000	0.390	0.675
389	A	0.530	1.040	0.540	0.703	0.292
390	U	0.000	0.130	0.080	0.070	0.066
391	G	0.000	0.020	0.020	0.013	0.012
392	U	0.000	0.010	0.000	0.003	0.006
393	G	0.050	0.010	0.020	0.027	0.021
394	A	0.190	0.160	0.250	0.200	0.046
395	A	0.170	0.150	0.250	0.190	0.053
396	A	0.360	0.410	0.600	0.457	0.127
397	G	0.480	0.210	0.310	0.333	0.137
398	A	0.260	0.070	0.080	0.137	0.107
399	G	0.010	0.050	0.020	0.027	0.021
400	A	0.000	0.100	0.110	0.070	0.061
401	G	0.000	0.000	0.000	0.000	0.000
402	U	0.050	0.090	0.000	0.047	0.045
403	A	0.190	0.210	0.170	0.190	0.020
404	G	0.160	0.210	0.290	0.220	0.066
405	U	0.270	0.190	0.570	0.343	0.200
406	G	0.640	0.000	0.000	0.213	0.370
407	C	0.000	0.000	0.000	0.000	0.000
408	A	0.800	0.150	0.000	0.317	0.425
409	A	0.450	0.180	0.370	0.333	0.139
410	U	0.480	0.240	0.000	0.240	0.240
411	A	0.560	0.490	0.370	0.473	0.096
412	G	0.370	0.220	0.250	0.280	0.079
413	A	0.520	0.430	0.330	0.427	0.095
414	A	0.500	0.390	0.420	0.437	0.057
415	U	0.370	0.280	0.320	0.323	0.045
416	U	0.350	0.220	0.360	0.310	0.078
417	U	0.520	0.320	0.360	0.400	0.106
418	U	0.360	0.180	0.140	0.227	0.117
419	A	0.400	0.260	0.130	0.263	0.135
420	U	0.100	0.090	0.000	0.063	0.055
421	C	0.000	0.250	0.000	0.083	0.144
422	A	0.530	0.720	0.000	0.417	0.373
423	G	0.180	0.180	0.190	0.183	0.006
424	U	0.322	0.574	0.079	0.325	0.248
425	U	0.278	0.037	0.284	0.200	0.141
426	U	0.000	0.000	0.000	0.000	0.000
427	C	0.000	0.000	0.000	0.000	0.000
428	U	0.377	0.029	0.778	0.395	0.375
429	A	0.415	0.283	0.607	0.435	0.163
430	A	-999	-999	-999	-999	0.000
431	U	-999	-999	-999	-999	0.000
432	A	-999	-999	-999	-999	0.000

hSHAPE Reactivities from 3 independent experiments (SP101*i*)						
Nucleotides		Experiment 1	Experiment 2	Experiment 3	Mean	SD
Number	Sequence					
1	G	-999	-999	-999	-999	0.000
2	C	-999	-999	-999	-999	0.000
3	A	-999	-999	-999	-999	0.000
4	A	-999	-999	-999	-999	0.000
5	C	-999	-999	-999	-999	0.000
6	A	-999	-999	-999	-999	0.000
7	G	-999	-999	-999	-999	0.000
8	U	-999	-999	-999	-999	0.000
9	C	-999	-999	-999	-999	0.000
10	C	-999	-999	-999	-999	0.000
11	U	-999	-999	-999	-999	0.000
12	A	-999	-999	-999	-999	0.000
13	A	-999	-999	-999	-999	0.000
14	U	-999	-999	-999	-999	0.000
15	A	-999	-999	-999	-999	0.000
16	U	-999	-999	-999	-999	0.000
17	U	-999	-999	-999	-999	0.000
18	C	-999	-999	-999	-999	0.000
19	A	0.640	0.651	0.659	0.650	0.010
20	C	0.380	0.400	0.391	0.390	0.010
21	G	0.620	0.890	0.756	0.755	0.135
22	U	0.820	1.150	1.150	1.040	0.191
23	C	0.150	0.260	0.490	0.300	0.173
24	U	1.455	1.650	1.740	1.615	0.146
25	C	0.175	0.270	0.020	0.155	0.126
26	G	0.615	0.580	0.340	0.512	0.150
27	U	0.075	0.100	0.710	0.295	0.360
28	G	0.170	0.000	0.270	0.147	0.137
29	U	0.395	0.860	0.170	0.475	0.352
30	G	0.480	0.430	0.300	0.403	0.093
31	U	0.270	0.740	0.530	0.513	0.235
32	U	0.860	0.840	0.130	0.610	0.416
33	U	0.940	1.070	0.680	0.897	0.199
34	G	0.415	0.660	0.635	0.570	0.135
35	U	1.090	0.655	0.490	0.745	0.310
36	G	0.190	1.060	0.810	0.687	0.448
37	U → G	0.030	0.180	0.090	0.100	0.075
38	C	0.205	0.170	0.050	0.142	0.081
39	U → G	0.180	0.050	0.050	0.093	0.075
40	G	1.155	1.260	0.940	1.118	0.163
41	U	0.615	0.420	0.490	0.508	0.099
42	U	1.050	1.130	0.990	1.057	0.070
43	C	0.250	0.180	0.170	0.200	0.044
44	G	0.195	0.340	0.315	0.283	0.078
45	C	0.000	0.000	0.000	0.000	0.000
46	C	0.000	0.000	0.000	0.000	0.000
47	A	0.570	0.780	0.550	0.633	0.127
48	U	0.375	0.460	0.400	0.412	0.044

hSHAPE Reactivities from 3 independent experiments (SP101*i*)						
Nucleotides		Experiment 1	Experiment 2	Experiment 3	Mean	SD
Number	Sequence					
49	C	0.145	0.120	0.140	0.135	0.013
50	C	0.000	0.000	0.000	0.000	0.000
51	C	0.070	0.004	0.001	0.025	0.039
52	G	0.495	0.430	0.340	0.422	0.078
53	U	0.460	0.440	0.320	0.407	0.076
54	C	0.210	0.215	0.285	0.237	0.042
55	U	0.090	0.035	0.050	0.058	0.028
56	C	0.090	0.220	0.120	0.143	0.068
57	C	0.000	0.000	0.000	0.000	0.000
58	G	0.000	0.000	0.000	0.000	0.000
59	C	0.000	0.000	0.000	0.000	0.000
60	U	0.075	0.190	0.050	0.105	0.075
61	C	0.110	0.215	0.220	0.182	0.062
62	G	0.000	0.000	0.000	0.000	0.000
63	U	0.110	0.190	0.220	0.173	0.057
64	C	0.570	0.510	1.010	0.697	0.273
65	A	0.305	0.460	0.420	0.395	0.080
66	C	0.100	0.065	0.020	0.062	0.040
67	U	0.285	0.520	0.340	0.382	0.123
68	U	0.495	0.860	0.680	0.678	0.183
69	A	0.595	0.760	0.635	0.663	0.086
70	U	0.330	0.545	0.385	0.420	0.112
71	C	0.070	0.020	0.080	0.057	0.032
72	C	0.000	0.000	0.000	0.000	0.000
73	U	0.595	0.685	0.500	0.593	0.093
74	U	1.450	1.645	0.990	1.362	0.336
75	C	0.370	0.320	0.450	0.380	0.066
76	A	1.280	1.585	1.050	1.305	0.268
77	C	0.225	0.530	0.250	0.335	0.169
78	U	0.870	1.005	1.015	0.963	0.081
79	U	1.195	1.210	1.400	1.268	0.114
80	U	0.560	0.410	0.660	0.543	0.126
81	C	0.010	0.215	0.200	0.142	0.114
82	C	0.200	0.280	-0.010	0.157	0.150
83	A	1.205	1.115	1.250	1.190	0.069
84	G	1.930	2.450	2.170	2.183	0.260
85	A	0.120	0.230	0.330	0.227	0.105
86	G	0.000	0.000	0.000	0.000	0.000
87	G	0.000	0.000	0.000	0.000	0.000
88	G	0.000	0.000	0.000	0.000	0.000
89	U	0.000	0.000	0.000	0.000	0.000
90	C	0.000	0.000	0.000	0.000	0.000
91	C	0.000	0.000	0.000	0.000	0.000
92	C	0.000	0.000	0.000	0.000	0.000
93	C	0.000	0.000	0.000	0.000	0.000
94	C	0.000	0.000	0.000	0.000	0.000
95	C	0.000	0.000	0.000	0.000	0.000
96	G	0.000	0.000	0.000	0.000	0.000

hSHAPE Reactivities from 3 independent experiments (SP101*i*)						
Nucleotides		Experiment 1	Experiment 2	Experiment 3	Mean	SD
Number	Sequence					
97	C	0.228	0.650	0.000	0.293	0.330
98	A	0.230	0.400	0.170	0.267	0.119
99	G	0.490	0.570	0.555	0.538	0.043
100	A	1.315	1.150	1.220	1.228	0.083
101	C	0.060	0.195	0.070	0.108	0.075
102	C	0.055	0.275	0.160	0.163	0.110
103	C	0.660	0.300	0.130	0.363	0.271
104	C	0.008	0.012	0.001	0.007	0.006
105	G	0.100	0.055	0.010	0.055	0.045
106	G	0.170	0.070	0.010	0.083	0.081
107	U	0.140	0.105	0.055	0.100	0.043
108	G	0.110	0.000	0.050	0.053	0.055
109	A	0.000	0.070	0.050	0.040	0.036
110	C	0.030	0.000	0.010	0.013	0.015
111	C	0.210	0.260	0.455	0.308	0.129
112	C	0.600	0.825	0.640	0.688	0.120
113	U	2.155	2.385	2.425	2.322	0.146
114	C	0.855	1.355	1.000	1.070	0.257
115	A	2.810	2.850	2.955	2.872	0.075
116	G	0.225	0.225	0.190	0.213	0.020
117	G	0.090	0.025	0.040	0.052	0.034
118	U	0.080	0.135	0.010	0.075	0.063
119	C	0.050	0.290	0.160	0.167	0.120
120	G	0.790	0.535	0.320	0.548	0.235
121	G	0.475	0.325	0.550	0.450	0.115
122	C	0.110	0.120	0.020	0.083	0.055
123	C	0.041	0.037	0.050	0.043	0.007
124	G	0.185	0.225	0.095	0.168	0.067
125	A	0.230	0.215	0.255	0.233	0.020
126	C	0.035	0.050	0.050	0.045	0.009
127	U	0.055	0.170	0.210	0.145	0.080
128	G	0.175	0.140	0.265	0.193	0.064
129	C	0.090	0.070	0.020	0.060	0.036
130	G	0.000	0.000	0.000	0.000	0.000
131	G	0.000	0.000	0.000	0.000	0.000
132	C	0.240	0.170	0.070	0.160	0.085
133	A	0.190	0.360	0.400	0.317	0.112
134	G	0.710	0.330	0.230	0.423	0.253
135	C	1.200	1.370	0.990	1.187	0.190
136	U	0.765	0.685	0.810	0.753	0.063
137	G	0.250	0.290	0.505	0.348	0.137
138	G	0.390	0.440	0.520	0.450	0.066
139	C	0.135	0.225	0.190	0.183	0.045
140	G	0.125	0.155	0.175	0.152	0.025
141	C	0.000	0.000	0.000	0.000	0.000
142	C	0.000	0.000	0.000	0.000	0.000
143	C	0.325	0.215	0.240	0.260	0.058
144	G	0.495	0.460	0.535	0.497	0.038

**hSHAPE Reactivities from 3 independent experiments (SP101*i*)**						
**Nucleotides**		**Experiment 1**	**Experiment 2**	**Experiment 3**	**Mean**	**SD**
**Number**	**Sequence**					
145	A	1.035	1.025	1.210	1.090	0.104
146	A	0.970	1.315	1.100	1.128	0.174
147	C	0.990	0.460	1.110	0.853	0.346
148	A	0.305	0.415	0.480	0.400	0.088
149	G	0.000	0.000	0.000	0.000	0.000
150	G	0.000	0.000	0.000	0.000	0.000
151	G	0.000	0.000	0.000	0.000	0.000
152	A	0.000	0.000	0.000	0.000	0.000
153	C	0.000	0.000	0.000	0.000	0.000
154	C	0.000	0.000	0.000	0.000	0.000
155	C	0.000	0.000	0.000	0.000	0.000
156	U	0.325	0.150	0.030	0.168	0.148
157	C	0.465	0.265	0.305	0.345	0.106
158	G	0.065	0.025	0.020	0.037	0.025
159	G	0.010	0.025	-0.020	0.005	0.023
160	A	0.220	0.220	0.190	0.210	0.017
161	U	0.685	0.575	0.525	0.595	0.082
162	A	0.110	0.060	0.150	0.107	0.045
163	A	0.120	0.015	0.105	0.080	0.057
164	G	0.000	0.000	0.000	0.000	0.000
165	U	0.000	0.000	0.000	0.000	0.000
166	G	0.000	0.000	0.000	0.000	0.000
167	A	0.210	0.100	0.350	0.220	0.125
168	C	0.055	0.085	-0.045	0.032	0.068
169	C	0.060	0.195	-0.060	0.065	0.128
170	C	0.140	0.170	-0.010	0.100	0.096
171	U	0.340	0.055	0.340	0.245	0.165
172	U	0.515	0.550	0.495	0.520	0.028
173	G	0.460	0.420	0.640	0.507	0.117
174	U	0.140	0.335	0.470	0.315	0.166
175	C	0.030	0.150	0.040	0.073	0.067
176	U	0.000	0.000	0.000	0.000	0.000
177	C	0.000	0.000	0.000	0.000	0.000
178	U	0.550	0.555	0.390	0.498	0.094
179	A	0.590	0.690	0.780	0.687	0.095
180	U	0.090	0.070	0.245	0.135	0.096
181	U	0.190	0.210	0.240	0.213	0.025
182	U	0.025	0.015	0.045	0.028	0.015
183	C	0.000	0.000	0.000	0.000	0.000
184	U	0.225	0.320	0.040	0.195	0.142
185	A	0.000	0.045	0.135	0.060	0.069
186	C	0.010	0.021	0.035	0.022	0.013
187	U	0.530	0.650	0.670	0.617	0.076
188	A	0.445	0.810	0.710	0.655	0.189
189	U	0.010	0.065	0.060	0.045	0.030
190	U	0.060	0.160	0.130	0.117	0.051
191	U	0.605	0.540	0.530	0.558	0.041
192	G	0.050	0.185	0.070	0.102	0.073

hSHAPE Reactivities from 3 independent experiments (SP101*i*)						
Nucleotides		Experiment 1	Experiment 2	Experiment 3	Mean	SD
Number	Sequence					
193	G	0.315	0.350	0.775	0.480	0.256
194	U	0.555	0.445	0.855	0.618	0.212
195	G	0.605	0.475	1.140	0.740	0.352
196	U	0.320	0.320	0.745	0.462	0.245
197	U	0.580	0.810	0.960	0.783	0.191
198	U	0.270	0.350	0.530	0.383	0.133
199	G	0.000	0.000	0.000	0.000	0.000
200	U	0.000	0.000	0.000	0.000	0.000
201	C	0.030	0.100	0.027	0.052	0.041
202	U	0.135	0.205	0.150	0.163	0.037
203	U	0.295	0.350	0.290	0.312	0.033
204	G	0.255	0.135	0.205	0.198	0.060
205	U	0.445	0.675	0.180	0.433	0.248
206	A	0.170	0.140	0.150	0.153	0.015
207	U	0.025	0.225	0.210	0.153	0.111
208	U	0.535	0.775	0.400	0.570	0.190
209	G	0.465	0.525	0.675	0.555	0.108
210	U	0.040	0.085	0.095	0.073	0.029
211	C	0.000	0.000	0.000	0.000	0.000
212	U	0.000	0.000	0.000	0.000	0.000
213	C	0.000	0.000	0.000	0.000	0.000
214	U	0.000	0.000	0.000	0.000	0.000
215	U	0.090	0.030	0.105	0.075	0.040
216	U	0.090	0.020	0.055	0.055	0.035
217	C	0.080	0.065	0.010	0.052	0.037
218	U	0.175	0.395	0.340	0.303	0.114
219	U	0.440	0.380	0.600	0.473	0.114
220	G	0.445	0.385	0.665	0.498	0.147
221	U	0.200	0.330	0.100	0.210	0.115
222	C	0.200	0.255	0.150	0.202	0.053
223	U	0.270	0.225	0.300	0.265	0.038
224	G	0.070	0.150	0.310	0.177	0.122
225	G	0.000	0.000	0.000	0.000	0.000
226	C	0.000	0.000	0.000	0.000	0.000
227	U	0.525	0.450	0.450	0.475	0.043
228	A	0.105	0.360	0.470	0.312	0.187
229	U	0.095	0.085	0.240	0.140	0.087
230	C	0.400	0.090	0.520	0.337	0.222
231	A	0.360	0.755	0.540	0.552	0.198
232	U	0.130	0.235	0.190	0.185	0.053
233	C	0.360	0.100	0.261	0.240	0.131
234	A	0.430	0.400	0.530	0.453	0.068
235	C	0.450	0.000	0.720	0.390	0.364
236	A	0.595	0.780	0.510	0.628	0.138
237	A	0.915	0.780	0.875	0.857	0.069
238	G	0.465	0.345	0.545	0.452	0.101
239	A	0.175	0.245	0.235	0.218	0.038
240	G	0.005	0.010	0.007	0.007	0.003

hSHAPE Reactivities from 3 independent experiments (SP101*i*)						
Nucleotides		Experiment 1	Experiment 2	Experiment 3	Mean	SD
Number	Sequence					
241	C	0.085	0.095	0.050	0.077	0.024
242	G	0.325	0.230	0.360	0.305	0.067
243	G	0.310	0.450	0.545	0.435	0.118
244	A	0.210	0.805	0.405	0.473	0.303
245	A	0.270	0.490	0.480	0.413	0.124
246	C	0.115	0.215	0.110	0.147	0.059
247	G	0.060	0.155	0.020	0.078	0.069
248	G	0.070	0.250	0.040	0.120	0.114
249	A	1.525	1.910	1.570	1.668	0.210
250	C	0.200	0.085	0.010	0.098	0.096
251	U	0.000	0.000	0.000	0.000	0.000
252	C	0.020	0.195	0.080	0.098	0.089
253	A	0.010	0.049	0.120	0.060	0.056
254	C	0.050	0.147	0.005	0.067	0.073
255	C	0.047	0.328	0.060	0.145	0.159
256	A	0.380	1.020	0.730	0.710	0.320
257	U	1.120	1.730	0.810	1.220	0.468
258	A	0.670	0.680	0.950	0.767	0.159
259	G	0.090	0.105	0.205	0.133	0.063
260	G	0.280	0.040	0.045	0.122	0.137
261	G	0.100	0.120	0.080	0.100	0.020
262	A	0.350	0.355	0.530	0.412	0.103
263	G	0.290	0.265	0.360	0.305	0.049
264	C	0.170	0.300	0.100	0.190	0.101
265	U	0.865	0.915	1.350	1.043	0.267
266	G	0.840	0.760	0.905	0.835	0.073
267	C	1.140	2.485	1.120	1.582	0.782
268	A	1.335	1.740	1.690	1.588	0.221
269	G	0.735	0.860	0.720	0.772	0.077
270	U	0.270	0.200	0.020	0.163	0.129
271	C	0.000	0.000	0.000	0.000	0.000
272	C	0.000	0.000	0.000	0.000	0.000
273	C	0.000	0.000	0.000	0.000	0.000
274	G	0.275	0.385	0.080	0.247	0.154
275	C	0.365	0.190	0.070	0.208	0.148
276	C	0.270	0.320	0.200	0.263	0.060
277	U	0.110	0.199	0.200	0.170	0.052
278	A	0.175	0.065	0.150	0.130	0.058
279	C	0.315	0.085	0.275	0.225	0.123
280	G	0.390	0.220	0.395	0.335	0.100
281	G	1.150	1.350	1.845	1.448	0.358
282	A	1.645	1.635	1.920	1.733	0.162
283	G	0.375	0.480	0.620	0.492	0.123
284	A	2.075	2.550	2.370	2.332	0.240
285	A	1.625	1.710	1.800	1.712	0.088
286	G	0.885	0.925	1.595	1.135	0.399
287	A	2.040	2.440	2.730	2.403	0.346
288	G	0.400	0.530	0.845	0.592	0.229

hSHAPE Reactivities from 3 independent experiments (SP101*i*)						
Nucleotides		Experiment 1	Experiment 2	Experiment 3	Mean	SD
Number	Sequence					
289	G	0.090	0.105	0.300	0.165	0.117
290	U	0.125	0.080	0.090	0.098	0.024
291	A	0.053	0.020	0.001	0.025	0.026
292	G	0.000	0.000	0.000	0.000	0.000
293	G	0.240	0.329	0.145	0.238	0.092
294	U	0.755	0.805	0.540	0.700	0.141
295	U	0.995	0.690	0.720	0.802	0.168
296	A	0.770	0.920	0.910	0.867	0.084
297	C	0.570	0.550	0.590	0.570	0.020
298	G	0.150	0.015	0.010	0.058	0.079
299	G	0.000	0.000	0.000	0.000	0.000
300	U	0.050	0.029	0.001	0.027	0.025
301	G	0.000	0.000	0.000	0.000	0.000
302	A	0.000	0.000	0.000	0.000	0.000
303	G	0.000	0.000	0.000	0.000	0.000
304	C	0.000	0.000	0.000	0.000	0.000
305	C	0.590	0.620	0.320	0.510	0.165
306	A	0.440	0.770	0.660	0.623	0.168
307	U	0.470	0.630	0.690	0.597	0.114
308	U	1.110	1.250	1.150	1.170	0.072
309	G	0.720	0.850	1.000	0.857	0.140
310	G	0.870	0.850	0.920	0.880	0.036
311	A	0.810	0.780	0.810	0.800	0.017
312	A	0.530	0.350	0.350	0.410	0.104
313	A	0.470	0.380	0.380	0.410	0.052
314	U	0.035	0.024	0.001	0.020	0.017
315	G	0.000	0.000	0.000	0.000	0.000
316	G	0.000	0.000	0.000	0.000	0.000
317	G	0.000	0.000	0.000	0.000	0.000
318	G	0.000	0.000	0.000	0.000	0.000
319	G	0.320	0.220	0.110	0.217	0.105
320	U	0.040	0.160	0.160	0.120	0.069
321	C	0.000	0.000	0.000	0.000	0.000
322	U	0.000	0.000	0.000	0.000	0.000
323	C	0.000	0.000	0.000	0.000	0.000
324	G	0.140	0.300	0.290	0.243	0.090
325	G	0.190	0.520	0.530	0.413	0.193
326	G	0.000	0.000	0.000	0.000	0.000
327	C	0.050	0.020	0.010	0.027	0.021
328	U	0.230	0.040	0.220	0.163	0.107
329	C	1.000	0.000	0.670	0.557	0.510
330	A	0.880	0.740	0.750	0.790	0.078
331	A	1.090	1.130	1.110	1.110	0.020
332	A	0.880	1.000	1.040	0.973	0.083
333	A	0.760	0.960	0.980	0.900	0.122
334	G	0.110	0.180	0.190	0.160	0.044
335	G	0.000	0.000	0.000	0.000	0.000
336	G	0.000	0.000	0.000	0.000	0.000

hSHAPE Reactivities from 3 independent experiments (SP101*i*)						
Nucleotides		Experiment 1	Experiment 2	Experiment 3	Mean	SD
Number	Sequence					
337	C	0.050	0.010	0.010	0.023	0.023
338	A	0.004	0.070	0.056	0.043	0.035
339	G	0.000	0.000	0.000	0.000	0.000
340	A	0.040	0.045	0.036	0.040	0.005
341	A	0.010	0.220	0.190	0.140	0.114
342	A	0.000	0.000	0.000	0.000	0.000
343	C	0.109	0.075	0.065	0.083	0.023
344	U	0.420	0.800	0.710	0.643	0.199
345	C	0.660	0.350	0.460	0.490	0.157
346	U	0.670	0.710	0.780	0.720	0.056
347	U	0.770	0.880	0.810	0.820	0.056
348	U	0.660	0.780	0.810	0.750	0.079
349	G	0.029	0.012	0.028	0.023	0.010
350	U	0.150	0.180	0.230	0.187	0.040
351	U	0.010	0.060	0.080	0.050	0.036
352	U	0.000	0.000	0.000	0.000	0.000
353	C	0.000	0.000	0.000	0.000	0.000
354	U	0.620	0.700	0.380	0.567	0.167
355	G	0.960	1.030	1.210	1.067	0.129
356	U	0.240	0.410	0.400	0.350	0.095
357	U	0.220	0.470	0.430	0.373	0.134
358	U	0.040	0.290	0.330	0.220	0.157
359	U	0.140	0.260	0.390	0.263	0.125
360	A	0.430	0.600	0.680	0.570	0.128
361	C	0.770	0.058	0.461	0.430	0.357
362	A	0.220	0.320	0.320	0.287	0.058
363	A	0.010	0.110	0.110	0.077	0.058
364	A	0.000	0.000	0.000	0.000	0.000
365	G	0.000	0.000	0.000	0.000	0.000
366	G	0.000	0.000	0.000	0.000	0.000
367	C	0.074	0.012	0.025	0.037	0.033
368	U	0.000	0.000	0.000	0.000	0.000
369	C	0.015	0.001	0.005	0.007	0.007
370	C	0.009	0.020	0.001	0.010	0.010
371	U	0.230	0.230	0.240	0.233	0.006
372	C	0.098	0.057	0.075	0.077	0.021
373	U	0.540	0.620	0.750	0.637	0.106
374	C	1.710	1.710	1.690	1.703	0.012
375	A	2.200	2.440	2.290	2.310	0.121
376	G	1.380	1.850	1.820	1.683	0.263
377	A	1.240	1.460	1.380	1.360	0.111
378	G	0.000	0.000	0.000	0.000	0.000
379	A	0.000	0.000	0.000	0.000	0.000
380	G	0.000	0.000	0.000	0.000	0.000
381	G	0.000	0.000	0.000	0.000	0.000
382	G	0.890	0.000	0.170	0.353	0.472
383	G	0.000	0.000	0.000	0.000	0.000
384	U	0.000	0.000	0.000	0.000	0.000

hSHAPE Reactivities from 3 independent experiments (SP101*i*)						
Nucleotides		Experiment 1	Experiment 2	Experiment 3	Mean	SD
Number	Sequence					
385	C	0.189	0.258	0.145	0.197	0.057
386	U	0.330	0.180	0.290	0.267	0.078
387	U	1.040	0.750	1.270	1.020	0.261
388	C	1.050	0.020	1.150	0.740	0.626
389	A	0.820	0.900	0.930	0.883	0.057
390	U	0.080	0.140	0.120	0.113	0.031
391	G	0.000	0.000	0.000	0.000	0.000
392	U	0.220	0.110	0.070	0.133	0.078
393	G	0.180	0.030	0.040	0.083	0.084
394	A	0.310	0.180	0.150	0.213	0.085
395	A	0.520	0.370	0.370	0.420	0.087
396	A	0.480	0.470	0.470	0.473	0.006
397	G	0.300	0.250	0.250	0.267	0.029
398	A	0.190	0.100	0.130	0.140	0.046
399	G	0.250	0.040	0.070	0.120	0.114
400	A	0.210	0.180	0.060	0.150	0.079
401	G	0.000	0.080	0.380	0.153	0.200
402	U	0.058	0.000	0.143	0.067	0.072
403	A	0.001	0.050	0.018	0.023	0.025
404	G	0.000	0.070	0.110	0.060	0.056
405	U	0.110	0.170	0.090	0.123	0.042
406	G	0.000	0.000	0.000	0.000	0.000
407	C	0.680	0.536	0.125	0.447	0.288
408	A	1.240	1.090	0.410	0.913	0.442
409	A	0.870	0.480	0.260	0.537	0.309
410	U	0.330	0.360	0.360	0.350	0.017
411	A	0.570	0.740	0.580	0.630	0.095
412	G	0.820	0.560	0.590	0.657	0.142
413	A	0.660	0.660	0.690	0.670	0.017
414	A	0.440	0.490	0.620	0.517	0.093
415	U	0.320	0.330	0.530	0.393	0.118
416	U	0.140	0.240	0.540	0.307	0.208
417	U	0.500	0.450	0.630	0.527	0.093
418	U	0.430	0.180	0.480	0.363	0.161
419	A	0.400	0.240	0.030	0.223	0.186
420	U	0.154	0.015	0.010	0.060	0.082
421	C	1.830	0.000	0.400	0.743	0.962
422	A	1.430	1.010	1.220	1.220	0.210
423	G	0.290	0.360	0.325	0.325	0.035
424	U	0.110	0.230	0.171	0.170	0.060
425	U	-999	-999	-999	-999	0.000
426	U	-999	-999	-999	-999	0.000
427	C	-999	-999	-999	-999	0.000
428	U	-999	-999	-999	-999	0.000
429	A	-999	-999	-999	-999	0.000
430	A	-999	-999	-999	-999	0.000
431	U	-999	-999	-999	-999	0.000
432	A	-999	-999	-999	-999	0.000

hSHAPE Reactivities from 3 independent experiments (SP102*i*)						
Nucleotides		Experiment 1	Experiment 2	Experiment 3	Mean	SD
Number	Sequence					
1	G	-999	-999	-999	-999	0.000
2	C	-999	-999	-999	-999	0.000
3	A	-999	-999	-999	-999	0.000
4	A	-999	-999	-999	-999	0.000
5	C	-999	-999	-999	-999	0.000
6	A	-999	-999	-999	-999	0.000
7	G	-999	-999	-999	-999	0.000
8	U	-999	-999	-999	-999	0.000
9	C	-999	-999	-999	-999	0.000
10	C	-999	-999	-999	-999	0.000
11	U	-999	-999	-999	-999	0.000
12	A	-999	-999	-999	-999	0.000
13	A	-999	-999	-999	-999	0.000
14	U	-999	-999	-999	-999	0.000
15	A	-999	-999	-999	-999	0.000
16	U	-999	-999	-999	-999	0.000
17	U	-999	-999	-999	-999	0.000
18	C	-999	-999	-999	-999	0.000
19	A	-999	-999	-999	-999	0.000
20	C	-999	-999	-999	-999	0.000
21	G	0.930	0.805	0.680	0.805	0.125
22	U	1.290	1.251	1.210	1.250	0.040
23	C	0.375	0.384	0.390	0.383	0.008
24	U	1.440	1.300	1.400	1.380	0.072
25	C	0.360	0.240	0.450	0.350	0.105
26	G	0.530	0.360	0.590	0.493	0.119
27	U	0.240	0.120	0.160	0.173	0.061
28	G	0.260	0.000	0.100	0.120	0.131
29	U	0.350	0.120	0.270	0.247	0.117
30	G	0.315	0.295	0.250	0.287	0.033
31	U	0.060	0.300	0.360	0.240	0.159
32	U	0.670	0.540	0.320	0.510	0.177
33	U	0.120	0.630	0.610	0.453	0.289
34	G	0.625	0.635	0.560	0.607	0.041
35	U	0.620	0.660	0.555	0.612	0.053
36	G	0.905	0.575	0.690	0.723	0.168
37	U → G	0.060	0.110	0.125	0.098	0.034
38	C	0.135	0.170	0.050	0.118	0.062
39	U → G	0.035	0.060	0.070	0.055	0.018
40	G	0.325	0.600	0.110	0.345	0.246
41	U	0.205	0.390	0.435	0.343	0.122
42	U	0.745	0.830	1.095	0.890	0.183
43	C	0.320	0.440	0.760	0.507	0.227
44	G	0.870	0.750	0.680	0.767	0.096
45	C	0.000	0.000	0.000	0.000	0.000
46	C	0.000	0.000	0.000	0.000	0.000
47	A	0.355	0.375	0.075	0.268	0.168
48	U	0.410	0.375	0.375	0.387	0.020

hSHAPE Reactivities from 3 independent experiments (SP102*i*)						
Nucleotides		Experiment 1	Experiment 2	Experiment 3	Mean	SD
Number	Sequence					
49	C	0.060	0.004	0.018	0.027	0.029
50	C	0.000	0.000	0.000	0.000	0.000
51	C	0.000	0.000	0.000	0.000	0.000
52	G	0.515	0.490	0.090	0.365	0.238
53	U	0.450	0.280	0.320	0.350	0.089
54	C	0.335	0.350	0.315	0.333	0.018
55	U	0.075	0.010	0.055	0.047	0.033
56	C	0.020	0.000	0.185	0.068	0.102
57	C	0.040	0.010	0.032	0.027	0.016
58	G	0.000	0.000	0.000	0.000	0.000
59	C	0.000	0.000	0.000	0.000	0.000
60	U	0.190	0.115	0.085	0.130	0.054
61	C	0.175	0.150	0.100	0.142	0.038
62	G	0.000	0.000	0.000	0.000	0.000
63	U	0.000	0.000	0.000	0.000	0.000
64	C	0.000	0.000	0.000	0.000	0.000
65	A	0.210	0.380	0.210	0.267	0.098
66	C	0.110	0.020	0.165	0.098	0.073
67	U	0.170	0.155	0.295	0.207	0.077
68	U	0.000	0.000	0.000	0.000	0.000
69	A	0.525	0.625	0.610	0.587	0.054
70	U	0.270	0.275	0.185	0.243	0.051
71	C	0.000	0.000	0.000	0.000	0.000
72	C	0.000	0.000	0.000	0.000	0.000
73	U	0.760	0.640	0.680	0.693	0.061
74	U	0.980	0.700	0.870	0.850	0.141
75	C	0.000	0.000	0.000	0.000	0.000
76	A	1.340	0.850	0.915	1.035	0.266
77	C	0.550	0.350	0.620	0.507	0.140
78	U	1.960	1.520	1.190	1.557	0.386
79	U	1.650	1.675	1.195	1.507	0.270
80	U	1.205	1.395	0.820	1.140	0.293
81	C	0.520	0.680	0.020	0.407	0.344
82	C	0.010	0.000	0.070	0.027	0.038
83	A	0.490	2.390	2.050	1.643	1.013
84	G	1.955	1.640	1.260	1.618	0.348
85	A	0.155	0.070	0.000	0.075	0.078
86	G	0.000	0.000	0.000	0.000	0.000
87	G	0.010	0.030	0.130	0.057	0.064
88	G	0.000	0.000	0.000	0.000	0.000
89	U	0.000	0.000	0.000	0.000	0.000
90	C	0.020	0.058	0.124	0.067	0.053
91	C	0.070	0.030	0.060	0.053	0.021
92	C	0.000	0.000	0.000	0.000	0.000
93	C	0.000	0.140	0.000	0.047	0.081
94	C	0.000	0.000	0.000	0.000	0.000
95	C	0.000	0.000	0.000	0.000	0.000
96	G	0.010	0.090	0.170	0.090	0.080

hSHAPE Reactivities from 3 independent experiments (SP102*i*)						
Nucleotides		Experiment 1	Experiment 2	Experiment 3	Mean	SD
Number	Sequence					
97	C	0.000	0.000	0.000	0.000	0.000
98	A	0.330	0.215	0.370	0.305	0.080
99	G	0.670	0.700	0.690	0.687	0.015
100	A	1.570	1.350	1.085	1.335	0.243
101	C	0.210	0.100	0.240	0.183	0.074
102	C	0.000	0.160	0.160	0.107	0.092
103	C	0.020	0.360	0.000	0.127	0.202
104	C	0.090	0.120	0.000	0.070	0.062
105	G	0.100	0.090	0.000	0.063	0.055
106	G	0.025	0.040	0.090	0.052	0.034
107	U	0.070	0.130	0.105	0.102	0.030
108	G	0.065	0.015	0.090	0.057	0.038
109	A	0.000	0.000	0.000	0.000	0.000
110	C	0.085	0.000	0.195	0.093	0.098
111	C	0.320	0.350	0.420	0.363	0.051
112	C	0.455	0.340	0.240	0.345	0.108
113	U	3.300	2.565	2.150	2.672	0.582
114	C	1.455	0.880	0.720	1.018	0.387
115	A	3.880	3.370	2.970	3.407	0.456
116	G	0.440	0.370	0.335	0.382	0.053
117	G	0.095	0.050	0.165	0.103	0.058
118	U	0.065	0.010	0.060	0.045	0.030
119	C	0.430	0.800	0.535	0.588	0.191
120	G	0.530	0.345	0.300	0.392	0.122
121	G	0.745	0.660	0.675	0.693	0.045
122	C	0.000	0.000	0.000	0.000	0.000
123	C	0.000	0.000	0.000	0.000	0.000
124	G	0.300	0.245	0.200	0.248	0.050
125	A	0.350	0.340	0.255	0.315	0.052
126	C	0.050	0.180	0.105	0.112	0.065
127	U	0.380	0.470	0.480	0.443	0.055
128	G	0.340	0.360	0.330	0.343	0.015
129	C	0.020	0.190	0.000	0.070	0.104
130	G	0.000	0.000	0.000	0.000	0.000
131	G	0.000	0.000	0.000	0.000	0.000
132	C	0.000	0.000	0.000	0.000	0.000
133	A	0.000	0.000	0.000	0.000	0.000
134	G	0.660	0.430	0.490	0.527	0.119
135	C	0.975	1.190	0.750	0.972	0.220
136	U	1.020	1.230	0.535	0.928	0.356
137	G	0.455	0.655	0.440	0.517	0.120
138	G	0.720	0.690	0.660	0.690	0.030
139	C	0.265	0.355	0.320	0.313	0.045
140	G	0.175	0.190	0.180	0.182	0.008
141	C	0.030	0.005	0.031	0.022	0.015
142	C	0.000	0.000	0.000	0.000	0.000
143	C	0.270	0.590	0.000	0.287	0.295
144	G	0.825	0.820	0.530	0.725	0.169

hSHAPE Reactivities from 3 independent experiments (SP102*i*)						
Nucleotides		Experiment 1	Experiment 2	Experiment 3	Mean	SD
Number	Sequence					
145	A	1.500	1.415	1.225	1.380	0.141
146	A	0.675	0.820	0.380	0.625	0.224
147	C	0.000	0.000	0.000	0.000	0.000
148	A	0.275	0.280	0.200	0.252	0.045
149	G	0.000	0.030	0.060	0.030	0.030
150	G	0.000	0.000	0.000	0.000	0.000
151	G	0.000	0.000	0.000	0.000	0.000
152	A	0.000	0.000	0.000	0.000	0.000
153	C	0.000	0.000	0.000	0.000	0.000
154	C	0.000	0.000	0.000	0.000	0.000
155	C	0.000	0.000	0.000	0.000	0.000
156	U	0.190	0.120	0.000	0.103	0.096
157	C	0.675	0.655	0.340	0.557	0.188
158	G	0.090	0.090	0.075	0.085	0.009
159	G	0.060	0.050	0.070	0.060	0.010
160	A	0.090	0.165	0.055	0.103	0.056
161	U	0.575	0.455	0.370	0.467	0.103
162	A	0.070	0.130	0.010	0.070	0.060
163	A	0.010	0.010	0.150	0.057	0.081
164	G	0.000	0.000	0.000	0.000	0.000
165	U	0.000	0.000	0.000	0.000	0.000
166	G	0.000	0.022	0.001	0.008	0.012
167	A	0.120	0.125	0.140	0.128	0.010
168	C	0.087	0.001	0.010	0.033	0.047
169	C	0.008	0.000	0.000	0.003	0.005
170	C	0.050	0.060	0.010	0.040	0.026
171	U	0.210	0.255	0.010	0.158	0.130
172	U	0.625	0.565	0.530	0.573	0.048
173	G	1.085	1.100	1.010	1.065	0.048
174	U	0.450	0.495	0.385	0.443	0.055
175	C	0.001	0.045	0.019	0.022	0.022
176	U	0.000	0.000	0.000	0.000	0.000
177	C	0.250	0.050	0.000	0.100	0.132
178	U	0.285	0.420	0.000	0.235	0.214
179	A	0.795	1.000	0.520	0.772	0.241
180	U	0.170	0.250	0.155	0.192	0.051
181	U	0.090	0.205	0.070	0.122	0.073
182	U	0.005	0.009	0.000	0.005	0.005
183	C	0.100	0.040	0.080	0.073	0.031
184	U	0.000	0.000	0.000	0.000	0.000
185	A	0.090	0.175	0.105	0.123	0.045
186	C	0.115	0.160	0.090	0.122	0.035
187	U	0.375	0.310	0.260	0.315	0.058
188	A	0.910	0.910	0.730	0.850	0.104
189	U	0.160	0.170	0.210	0.180	0.026
190	U	0.150	0.250	0.240	0.213	0.055
191	U	0.650	0.940	0.770	0.787	0.146
192	G	0.000	0.000	0.000	0.000	0.000

hSHAPE Reactivities from 3 independent experiments (SP102*i*)						
Nucleotides		Experiment 1	Experiment 2	Experiment 3	Mean	SD
Number	Sequence					
193	G	0.565	0.715	0.350	0.543	0.183
194	U	0.905	0.910	0.740	0.852	0.097
195	G	1.225	1.255	1.070	1.183	0.099
196	U	0.840	0.845	0.740	0.808	0.059
197	U	1.110	1.100	1.075	1.095	0.018
198	U	0.600	0.650	0.640	0.630	0.026
199	G	0.060	0.140	0.075	0.092	0.043
200	U	0.070	0.020	0.055	0.048	0.026
201	C	0.085	0.095	0.200	0.127	0.064
202	U	0.280	0.290	0.170	0.247	0.067
203	U	0.275	0.240	0.150	0.222	0.064
204	G	0.160	0.225	0.325	0.237	0.083
205	U	0.075	0.150	0.140	0.122	0.041
206	A	0.190	0.225	0.390	0.268	0.107
207	U	0.180	0.290	0.170	0.213	0.067
208	U	0.595	0.690	0.475	0.587	0.108
209	G	1.045	1.085	1.050	1.060	0.022
210	U	0.165	0.120	0.190	0.158	0.035
211	C	0.040	0.240	0.010	0.097	0.125
212	U	0.020	0.450	0.000	0.157	0.254
213	C	0.000	0.000	0.000	0.000	0.000
214	U	0.280	0.181	0.125	0.195	0.078
215	U	0.170	0.205	0.150	0.175	0.028
216	U	0.090	0.070	0.220	0.127	0.081
217	C	0.150	0.005	0.070	0.075	0.073
218	U	0.225	0.280	0.170	0.225	0.055
219	U	0.615	0.685	0.575	0.625	0.056
220	G	0.780	0.745	0.680	0.735	0.051
221	U	0.165	0.140	0.225	0.177	0.044
222	C	0.190	0.140	0.280	0.203	0.071
223	U	0.430	0.430	0.495	0.452	0.038
224	G	0.420	0.445	0.450	0.438	0.016
225	G	0.115	0.170	0.260	0.182	0.073
226	C	0.000	0.000	0.000	0.000	0.000
227	U	0.405	0.240	0.560	0.402	0.160
228	A	0.400	0.470	0.400	0.423	0.040
229	U	0.250	0.100	0.060	0.137	0.100
230	C	0.000	0.000	0.000	0.000	0.000
231	A	0.620	0.680	0.510	0.603	0.086
232	U	0.000	0.000	0.000	0.000	0.000
233	C	0.000	0.000	0.000	0.000	0.000
234	A	0.310	0.340	0.190	0.280	0.079
235	C	0.000	0.000	0.000	0.000	0.000
236	A	0.625	0.730	0.580	0.645	0.077
237	A	0.910	0.900	0.960	0.923	0.032
238	G	0.625	0.680	0.630	0.645	0.030
239	A	0.300	0.325	0.400	0.342	0.052
240	G	0.075	0.010	0.060	0.048	0.034

**hSHAPE Reactivities from 3 independent experiments (SP102*i*)**						
**Nucleotides**		**Experiment 1**	**Experiment 2**	**Experiment 3**	**Mean**	**SD**
**Number**	**Sequence**					
241	C	0.020	0.100	0.035	0.052	0.043
242	G	0.475	0.570	0.335	0.460	0.118
243	G	0.525	0.590	0.330	0.482	0.135
244	A	0.245	0.300	0.205	0.250	0.048
245	A	0.395	0.410	0.395	0.400	0.009
246	C	0.260	0.250	0.340	0.283	0.049
247	G	0.425	0.605	0.140	0.390	0.234
248	G	0.210	0.440	0.180	0.277	0.142
249	A	1.765	1.420	1.350	1.512	0.222
250	C	0.350	0.175	0.080	0.202	0.137
251	U	0.000	0.000	0.000	0.000	0.000
252	C	0.000	0.000	0.000	0.000	0.000
253	A	0.000	0.000	0.000	0.000	0.000
254	C	0.000	0.000	0.000	0.000	0.000
255	C	0.000	0.000	0.000	0.000	0.000
256	A	0.870	1.010	1.020	0.967	0.084
257	U	0.725	0.620	0.680	0.675	0.053
258	A	1.030	0.995	0.990	1.005	0.022
259	G	0.065	0.155	0.065	0.095	0.052
260	G	0.015	0.075	-0.015	0.025	0.046
261	G	0.025	0.065	0.030	0.040	0.022
262	A	0.595	0.565	0.690	0.617	0.065
263	G	0.505	0.500	0.455	0.487	0.028
264	C	0.565	0.395	0.380	0.447	0.103
265	U	1.765	1.810	1.865	1.813	0.050
266	G	1.515	1.290	1.385	1.397	0.113
267	C	0.000	0.000	0.000	0.000	0.000
268	A	2.025	2.040	2.010	2.025	0.015
269	G	1.520	1.225	1.330	1.358	0.150
270	U	0.230	0.210	0.200	0.213	0.015
271	C	0.100	0.065	0.000	0.055	0.051
272	C	0.000	0.000	0.000	0.000	0.000
273	C	0.000	0.000	0.000	0.000	0.000
274	G	0.000	0.000	0.000	0.000	0.000
275	C	0.055	0.210	0.000	0.088	0.109
276	C	0.230	0.190	0.180	0.200	0.026
277	U	0.015	0.120	0.025	0.053	0.058
278	A	0.075	0.040	0.105	0.073	0.033
279	C	0.340	0.365	0.395	0.367	0.028
280	G	0.405	0.430	0.520	0.452	0.060
281	G	1.815	1.750	1.875	1.813	0.063
282	A	2.860	2.865	2.755	2.827	0.062
283	G	0.915	0.875	1.055	0.948	0.095
284	A	3.345	3.240	3.340	3.308	0.059
285	A	2.740	1.855	2.325	2.307	0.443
286	G	1.445	1.720	1.470	1.545	0.152
287	A	3.650	2.205	3.595	3.150	0.819
288	G	0.885	0.390	0.855	0.710	0.278

hSHAPE Reactivities from 3 independent experiments (SP102*i*)						
Nucleotides		Experiment 1	Experiment 2	Experiment 3	Mean	SD
Number	Sequence					
289	G	0.030	0.140	0.230	0.133	0.100
290	U	0.000	0.000	0.000	0.000	0.000
291	A	0.000	0.000	0.000	0.000	0.000
292	G	0.030	0.030	0.000	0.020	0.017
293	G	0.000	0.160	0.240	0.133	0.122
294	U	0.440	0.650	0.620	0.570	0.114
295	U	0.465	0.400	0.810	0.558	0.220
296	A	0.990	0.990	0.700	0.893	0.167
297	C	0.950	0.750	0.735	0.812	0.120
298	G	0.050	0.030	0.070	0.050	0.020
299	G	0.000	0.000	0.000	0.000	0.000
300	U	0.060	0.070	0.000	0.043	0.038
301	G	0.060	0.020	0.020	0.033	0.023
302	A	0.010	0.055	0.010	0.025	0.026
303	G	0.000	0.000	0.000	0.000	0.000
304	C	0.000	0.000	0.000	0.000	0.000
305	C	0.000	0.000	0.480	0.160	0.277
306	A	0.650	0.820	0.600	0.690	0.115
307	U	0.680	0.780	0.620	0.693	0.081
308	U	1.420	1.630	1.470	1.507	0.110
309	G	1.470	1.560	1.180	1.403	0.199
310	G	0.950	0.940	0.980	0.957	0.021
311	A	0.970	1.240	1.160	1.123	0.139
312	A	0.370	0.530	0.310	0.403	0.114
313	A	0.100	0.180	0.130	0.137	0.040
314	U	0.000	0.000	0.000	0.000	0.000
315	G	0.000	0.000	0.000	0.000	0.000
316	G	0.000	0.000	0.000	0.000	0.000
317	G	0.000	0.000	0.000	0.000	0.000
318	G	0.000	0.000	0.000	0.000	0.000
319	G	0.170	0.149	0.110	0.143	0.030
320	U	0.280	0.260	0.210	0.250	0.036
321	C	0.230	0.100	0.030	0.120	0.101
322	U	0.000	0.000	0.030	0.010	0.017
323	C	0.270	0.020	0.000	0.097	0.150
324	G → U	0.000	0.000	0.000	0.000	0.000
325	G	0.000	0.000	0.000	0.000	0.000
326	G → U	0.000	0.000	0.000	0.000	0.000
327	C	0.230	0.147	0.005	0.127	0.114
328	U	0.290	0.360	0.210	0.287	0.075
329	C	0.000	0.000	0.000	0.000	0.000
330	A	1.190	1.490	1.480	1.387	0.170
331	A	0.890	0.960	1.020	0.957	0.065
332	A	1.090	1.170	1.340	1.200	0.128
333	A	0.860	0.910	1.110	0.960	0.132
334	G	0.280	0.310	0.340	0.310	0.030
335	G	0.080	0.120	0.160	0.120	0.040
336	G	0.005	0.010	0.025	0.013	0.010

hSHAPE Reactivities from 3 independent experiments (SP102*i*)						
Nucleotides		Experiment 1	Experiment 2	Experiment 3	Mean	SD
Number	Sequence					
337	C	0.048	0.003	0.001	0.017	0.027
338	A	0.010	0.010	0.010	0.010	0.000
339	G	0.000	0.000	0.000	0.000	0.000
340	A	0.170	0.240	0.150	0.187	0.047
341	A	0.530	0.600	0.780	0.637	0.129
342	A	0.090	0.060	0.220	0.123	0.085
343	C	0.000	0.000	0.000	0.000	0.000
344	U	0.000	0.000	0.000	0.000	0.000
345	C	0.360	0.000	0.000	0.120	0.208
346	U	0.100	0.190	0.170	0.153	0.047
347	U	0.280	0.390	0.390	0.353	0.064
348	U	0.400	0.430	0.500	0.443	0.051
349	G	0.200	0.280	0.310	0.263	0.057
350	U	0.550	0.630	0.700	0.627	0.075
351	U	0.230	0.210	0.350	0.263	0.076
352	U	0.000	0.000	0.000	0.000	0.000
353	C	0.000	0.000	0.000	0.000	0.000
354	U	0.880	1.100	1.050	1.010	0.115
355	G	1.250	1.280	1.250	1.260	0.017
356	U	0.390	0.450	0.470	0.437	0.042
357	U	0.330	0.400	0.550	0.427	0.112
358	U	0.160	0.170	0.380	0.237	0.124
359	U	0.000	0.000	0.000	0.000	0.000
360	A	0.380	0.480	0.460	0.440	0.053
361	C	0.000	0.000	0.000	0.000	0.000
362	A	0.310	0.350	0.430	0.363	0.061
363	A	0.130	0.130	0.180	0.147	0.029
364	A	0.000	0.000	0.000	0.000	0.000
365	G	0.000	0.000	0.000	0.000	0.000
366	G	0.000	0.000	0.000	0.000	0.000
367	C	0.430	0.050	0.040	0.173	0.222
368	U	0.000	0.000	0.000	0.000	0.000
369	C	0.000	0.000	0.000	0.000	0.000
370	C	0.210	0.075	0.015	0.100	0.100
371	U	0.400	0.450	0.380	0.410	0.036
372	C	0.300	0.240	0.000	0.180	0.159
373	U	0.730	0.860	0.730	0.773	0.075
374	C	1.840	1.720	1.630	1.730	0.105
375	A	2.850	2.870	3.230	2.983	0.214
376	G	1.960	2.170	2.080	2.070	0.105
377	A	1.720	1.730	1.820	1.757	0.055
378	G	0.000	0.000	0.000	0.000	0.000
379	A	0.000	0.000	0.000	0.000	0.000
380	G	0.052	0.058	0.011	0.040	0.026
381	G	0.000	0.000	0.000	0.000	0.000
382	G	0.000	0.040	0.000	0.013	0.023
383	G	0.030	0.000	0.250	0.093	0.137
384	U	0.004	0.020	0.007	0.010	0.009

hSHAPE Reactivities from 3 independent experiments (SP102*i*)						
Nucleotides		Experiment 1	Experiment 2	Experiment 3	Mean	SD
Number	Sequence					
385	C	0.166	0.005	0.060	0.077	0.082
386	U	0.000	0.000	0.000	0.000	0.000
387	U	0.089	0.105	0.098	0.097	0.008
388	C	0.000	0.000	0.000	0.000	0.000
389	A	0.990	1.090	1.000	1.027	0.055
390	U	0.120	0.210	0.240	0.190	0.062
391	G	0.080	0.050	0.130	0.087	0.040
392	U	0.000	0.190	0.230	0.140	0.123
393	G	0.070	0.090	0.140	0.100	0.036
394	A	0.380	0.300	0.320	0.333	0.042
395	A	0.410	0.690	0.640	0.580	0.149
396	A	0.180	0.730	0.880	0.597	0.369
397	G	0.320	0.430	0.400	0.383	0.057
398	A	0.250	0.390	0.510	0.383	0.130
399	G	0.100	0.300	0.470	0.290	0.185
400	A	0.000	0.000	0.000	0.000	0.000
401	G	0.036	0.025	0.000	0.020	0.018
402	U	0.120	0.000	0.430	0.183	0.222
403	A	0.030	0.000	0.070	0.033	0.035
404	G	0.050	0.058	0.033	0.047	0.013
405	U	0.280	0.110	0.160	0.183	0.087
406	G	0.000	0.000	0.000	0.000	0.000
407	C	0.000	0.000	0.000	0.000	0.000
408	A	1.060	0.000	0.450	0.503	0.532
409	A	0.430	0.170	0.490	0.363	0.170
410	U	0.000	0.000	0.000	0.000	0.000
411	A	0.740	0.500	1.070	0.770	0.286
412	G	0.450	0.610	0.780	0.613	0.165
413	A	0.620	0.860	1.130	0.870	0.255
414	A	0.400	0.510	0.840	0.583	0.229
415	U	0.280	0.470	0.630	0.460	0.175
416	U	0.110	0.190	0.430	0.243	0.167
417	U	0.330	0.420	0.690	0.480	0.187
418	U	0.000	0.000	0.220	0.073	0.127
419	A	0.440	0.410	0.560	0.470	0.079
420	U	0.050	0.000	0.080	0.043	0.040
421	C	0.000	0.000	0.000	0.000	0.000
422	A	1.690	0.940	2.040	1.557	0.562
423	G	0.210	0.160	0.540	0.303	0.206
424	U	-999	-999	-999	-999	0.000
425	U	-999	-999	-999	-999	0.000
426	U	-999	-999	-999	-999	0.000
427	C	-999	-999	-999	-999	0.000
428	U	-999	-999	-999	-999	0.000
429	A	-999	-999	-999	-999	0.000
430	A	-999	-999	-999	-999	0.000
431	U	-999	-999	-999	-999	0.000
432	A	-999	-999	-999	-999	0.000

hSHAPE Reactivities from 3 independent experiments (SP105*i*)						
Nucleotides		Experiment 1	Experiment 2	Experiment 3	Mean	SD
Number	Sequence					
1	G	-999	-999	-999	-999	0.000
2	C	-999	-999	-999	-999	0.000
3	A	-999	-999	-999	-999	0.000
4	A	-999	-999	-999	-999	0.000
5	C	-999	-999	-999	-999	0.000
6	A	-999	-999	-999	-999	0.000
7	G	-999	-999	-999	-999	0.000
8	U	-999	-999	-999	-999	0.000
9	C	-999	-999	-999	-999	0.000
10	C	-999	-999	-999	-999	0.000
11	U	-999	-999	-999	-999	0.000
12	A	-999	-999	-999	-999	0.000
13	A	-999	-999	-999	-999	0.000
14	U	-999	-999	-999	-999	0.000
15	A	-999	-999	-999	-999	0.000
16	U	-999	-999	-999	-999	0.000
17	U	-999	-999	-999	-999	0.000
18	C	-999	-999	-999	-999	0.000
19	A	0.254	0.24	0.332	0.275	0.050
20	C	0.140	0.220	0.280	0.213	0.070
21	G	0.390	0.460	0.500	0.450	0.056
22	U	1.300	1.850	1.290	1.480	0.320
23	C	0.590	0.280	0.460	0.443	0.156
24	U	1.850	2.540	1.920	2.103	0.380
25	C	0.490	0.645	0.510	0.548	0.084
26	G	0.260	0.265	0.230	0.252	0.019
27	U	0.000	0.000	0.000	0.000	0.000
28	G	0.280	0.205	0.200	0.228	0.045
29	U	0.545	0.475	0.220	0.413	0.171
30	G	0.295	0.510	0.280	0.362	0.129
31	U	0.330	0.100	0.015	0.148	0.163
32	U	0.415	0.410	0.300	0.375	0.065
33	U	0.395	0.265	0.285	0.315	0.070
34	G	0.470	0.385	0.400	0.418	0.045
35	U	1.150	1.370	1.065	1.195	0.157
36	G	0.420	0.300	0.310	0.343	0.067
37	U	0.320	0.040	0.040	0.133	0.162
38	C	0.220	-0.050	-0.015	0.052	0.147
39	U	0.000	0.000	0.000	0.000	0.000
40	G	0.050	0.050	0.000	0.033	0.029
41	U	0.000	0.000	0.000	0.000	0.000
42	U	0.325	0.295	0.395	0.338	0.051
43	C	0.360	0.590	0.440	0.463	0.117
44	G	0.630	1.020	0.855	0.835	0.196
45	C	0.000	0.000	0.000	0.000	0.000
46	C	0.000	0.000	0.000	0.000	0.000
47	A	1.200	1.300	0.990	1.163	0.158
48	U	1.595	1.390	1.135	1.373	0.230

hSHAPE Reactivities from 3 independent experiments (SP105*i*)						
Nucleotides		Experiment 1	Experiment 2	Experiment 3	Mean	SD
Number	Sequence					
49	C	1.180	1.390	1.110	1.227	0.146
50	C → G	0.555	0.865	0.835	0.752	0.171
51	C	0.310	0.425	0.305	0.347	0.068
52	G → U	0.520	0.930	0.835	0.762	0.215
53	U	0.680	0.680	0.610	0.657	0.040
54	C	0.210	0.265	0.230	0.235	0.028
55	U	0.065	0.070	0.100	0.078	0.019
56	C	0.080	0.060	0.010	0.050	0.036
57	C	0.000	0.000	0.000	0.000	0.000
58	G	0.080	0.190	0.050	0.107	0.074
59	C	0.000	0.000	0.000	0.000	0.000
60	U	0.170	0.410	0.205	0.262	0.130
61	C	0.060	0.285	0.110	0.152	0.118
62	G	0.000	0.000	0.000	0.000	0.000
63	U	0.240	0.195	0.120	0.185	0.061
64	C	1.730	1.570	1.150	1.483	0.300
65	A	0.150	0.530	0.350	0.343	0.190
66	C	0.000	0.035	0.000	0.012	0.020
67	U	0.350	0.335	0.330	0.338	0.010
68	U	0.410	0.790	0.580	0.593	0.190
69	A	0.785	0.645	0.615	0.682	0.091
70	U	0.555	0.375	0.350	0.427	0.112
71	C	0.305	0.305	0.210	0.273	0.055
72	C	0.100	0.020	0.020	0.047	0.046
73	U	0.660	0.650	0.585	0.632	0.041
74	U	2.360	1.300	1.015	1.558	0.709
75	C	1.675	2.740	1.740	2.052	0.597
76	A	1.025	1.345	1.120	1.163	0.164
77	C	0.340	0.420	0.365	0.375	0.041
78	U	1.220	1.120	1.200	1.180	0.053
79	U	1.055	1.500	1.380	1.312	0.230
80	U	0.730	1.005	0.940	0.892	0.144
81	C	0.475	0.385	0.325	0.395	0.075
82	C	0.340	0.340	0.330	0.337	0.006
83	A	3.710	1.960	1.090	2.253	1.334
84	G	1.470	1.820	1.765	1.685	0.188
85	A	0.100	0.190	0.150	0.147	0.045
86	G	0.000	0.000	0.000	0.000	0.000
87	G	0.000	0.000	0.000	0.000	0.000
88	G	0.000	0.000	0.000	0.000	0.000
89	U	0.000	0.000	0.000	0.000	0.000
90	C	0.000	0.000	0.000	0.000	0.000
91	C	0.000	0.000	0.000	0.000	0.000
92	C	0.000	0.000	0.000	0.000	0.000
93	C	0.000	0.000	0.000	0.000	0.000
94	C	0.000	0.000	0.000	0.000	0.000
95	C	0.100	0.060	0.010	0.057	0.045
96	G	0.065	0.010	0.020	0.032	0.029

hSHAPE Reactivities from 3 independent experiments (SP105*i*)						
**Nucleotides**		**Experiment 1**	**Experiment 2**	**Experiment 3**	**Mean**	**SD**
**Number**	**Sequence**					
97	C	0.000	0.000	0.000	0.000	0.000
98	A	0.270	0.310	0.215	0.265	0.048
99	G	0.580	0.740	0.650	0.657	0.080
100	A	1.285	1.540	1.440	1.422	0.128
101	C	0.215	0.135	0.180	0.177	0.040
102	C	0.120	0.185	0.195	0.167	0.041
103	C	0.180	0.320	0.500	0.333	0.160
104	C	0.000	0.000	0.000	0.000	0.000
105	G	0.000	0.000	0.000	0.000	0.000
106	G	0.000	0.060	0.090	0.050	0.046
107	U	0.170	0.115	0.025	0.103	0.073
108	G	0.075	0.090	0.025	0.063	0.034
109	A	0.075	0.130	0.010	0.072	0.060
110	C	0.000	0.000	0.000	0.000	0.000
111	C	0.240	0.230	0.250	0.240	0.010
112	C	0.330	0.430	0.295	0.352	0.070
113	U	2.180	2.545	2.425	2.383	0.186
114	C	1.535	1.405	1.205	1.382	0.166
115	A	2.810	3.225	3.070	3.035	0.210
116	G	0.235	0.215	0.200	0.217	0.018
117	G	0.035	0.090	0.025	0.050	0.035
118	U	0.208	0.001	0.009	0.073	0.117
119	C	0.215	0.465	0.020	0.233	0.223
120	G	0.645	0.730	0.745	0.707	0.054
121	G	0.555	0.755	0.715	0.675	0.106
122	C	0.000	0.000	0.000	0.000	0.000
123	C	0.000	0.000	0.000	0.000	0.000
124	G	0.140	0.145	0.190	0.158	0.028
125	A	0.225	0.305	0.320	0.283	0.051
126	C	0.055	0.090	0.030	0.058	0.030
127	U	0.145	0.160	0.150	0.152	0.008
128	G	0.265	0.255	0.245	0.255	0.010
129	C	0.115	0.000	0.080	0.065	0.059
130	G	0.000	0.000	0.000	0.000	0.000
131	G	0.000	0.000	0.000	0.000	0.000
132	C	0.140	0.270	0.180	0.197	0.067
133	A	1.160	1.135	0.860	1.052	0.166
134	G	0.315	0.180	0.180	0.225	0.078
135	C	1.005	1.185	0.765	0.985	0.211
136	U	0.675	0.590	0.775	0.680	0.093
137	G	0.365	0.370	0.450	0.395	0.048
138	G	0.180	0.275	-0.010	0.148	0.145
139	C	0.110	0.140	0.100	0.117	0.021
140	G	0.135	0.190	0.205	0.177	0.037
141	C	0.090	0.010	0.000	0.033	0.049
142	C	0.000	0.000	0.000	0.000	0.000
143	C	0.250	0.265	0.440	0.318	0.106
144	G	0.640	0.595	0.575	0.603	0.033

hSHAPE Reactivities from 3 independent experiments (SP105*i*)						
Nucleotides		Experiment 1	Experiment 2	Experiment 3	Mean	SD
Number	Sequence					
145	A	1.215	1.305	1.255	1.258	0.045
146	A	0.870	1.255	1.025	1.050	0.194
147	C	2.750	2.930	2.160	2.613	0.403
148	A	0.535	0.515	0.560	0.537	0.023
149	G	0.020	0.070	0.060	0.050	0.026
150	G	0.000	0.000	0.000	0.000	0.000
151	G	0.000	0.000	0.000	0.000	0.000
152	A	0.000	0.000	0.000	0.000	0.000
153	C	0.000	0.000	0.000	0.000	0.000
154	C	0.000	0.000	0.000	0.000	0.000
155	C	0.000	0.000	0.000	0.000	0.000
156	U	0.080	0.040	0.070	0.063	0.021
157	C	0.350	0.320	0.405	0.358	0.043
158	G	0.070	0.020	0.020	0.037	0.029
159	G	0.000	0.000	0.000	0.000	0.000
160	A	0.150	0.150	0.180	0.160	0.017
161	U	0.600	0.480	0.470	0.517	0.072
162	A	0.040	0.190	0.040	0.090	0.087
163	A	0.015	0.055	0.060	0.043	0.025
164	G	0.000	0.000	0.000	0.000	0.000
165	U	0.000	0.000	0.000	0.000	0.000
166	G	0.000	0.000	0.000	0.000	0.000
167	A	0.080	0.040	0.030	0.050	0.026
168	C	0.000	0.000	0.000	0.000	0.000
169	C	0.080	0.090	0.030	0.067	0.032
170	C	0.000	0.000	0.000	0.000	0.000
171	U	0.000	0.000	0.000	0.000	0.000
172	U	0.135	0.225	0.320	0.227	0.093
173	G	0.480	0.575	0.725	0.593	0.124
174	U	0.350	0.335	0.385	0.357	0.026
175	C	0.050	0.040	0.040	0.043	0.006
176	U	0.000	0.000	0.000	0.000	0.000
177	C	0.000	0.000	0.000	0.000	0.000
178	U	0.000	0.000	0.000	0.000	0.000
179	A	0.165	0.140	0.195	0.167	0.028
180	U	0.025	0.050	0.025	0.033	0.014
181	U	0.000	0.000	0.000	0.000	0.000
182	U	0.010	0.030	0.110	0.050	0.053
183	C	0.000	0.000	0.000	0.000	0.000
184	U	0.375	0.075	0.120	0.190	0.162
185	A	0.000	0.115	0.165	0.093	0.085
186	C	0.150	0.055	0.110	0.105	0.048
187	U	1.255	0.640	0.810	0.902	0.318
188	A	0.520	0.510	0.665	0.565	0.087
189	U	0.000	0.000	0.000	0.000	0.000
190	U	0.000	0.000	0.000	0.000	0.000
191	U	0.005	0.000	0.181	0.062	0.103
192	G	0.020	0.020	0.020	0.020	0.000

**hSHAPE Reactivities from 3 independent experiments (SP105*i*)**						
**Nucleotides**		**Experiment 1**	**Experiment 2**	**Experiment 3**	**Mean**	**SD**
**Number**	**Sequence**					
193	G	0.165	0.170	0.190	0.175	0.013
194	U	0.315	0.360	0.405	0.360	0.045
195	G	0.580	0.500	0.560	0.547	0.042
196	U	0.420	0.360	0.365	0.382	0.033
197	U	0.285	0.430	0.510	0.408	0.114
198	U	0.090	0.190	0.240	0.173	0.076
199	G	0.000	0.000	0.000	0.000	0.000
200	U	0.000	0.000	0.000	0.000	0.000
201	C	0.130	0.010	0.020	0.053	0.067
202	U	0.030	0.070	0.190	0.097	0.083
203	U	0.120	0.080	0.185	0.128	0.053
204	G	0.310	0.045	0.095	0.150	0.141
205	U	0.190	0.250	0.290	0.243	0.050
206	A	0.120	0.150	0.190	0.153	0.035
207	U	0.130	0.145	0.190	0.155	0.031
208	U	0.260	0.215	0.420	0.298	0.108
209	G	0.620	0.440	0.650	0.570	0.114
210	U	0.110	0.050	0.090	0.083	0.031
211	C	0.000	0.000	0.000	0.000	0.000
212	U	0.000	0.000	0.000	0.000	0.000
213	C	0.000	0.000	0.000	0.000	0.000
214	U	0.000	0.000	0.000	0.000	0.000
215	U	0.040	0.015	0.100	0.052	0.044
216	U	0.100	0.040	0.085	0.075	0.031
217	C	0.050	0.030	0.050	0.043	0.012
218	U	0.080	0.120	0.150	0.117	0.035
219	U	0.225	0.280	0.525	0.343	0.160
220	G	0.395	0.390	0.490	0.425	0.056
221	U	0.070	0.035	0.140	0.082	0.053
222	C	0.130	0.155	0.165	0.150	0.018
223	U	0.290	0.195	0.320	0.268	0.065
224	G	0.215	0.310	0.325	0.283	0.060
225	G	0.060	0.040	0.010	0.037	0.025
226	C	0.000	0.000	0.000	0.000	0.000
227	U	0.965	0.650	0.710	0.775	0.167
228	A	0.420	0.460	0.455	0.445	0.022
229	U	0.690	0.220	0.170	0.360	0.287
230	C	1.940	1.130	-0.140	0.977	1.048
231	A	0.300	0.555	0.315	0.390	0.143
232	U	0.000	0.000	0.000	0.000	0.000
233	C	1.850	1.525	-0.120	1.085	1.056
234	A	0.240	0.490	0.290	0.340	0.132
235	C	3.380	1.310	-0.190	1.500	1.793
236	A	0.620	0.860	0.725	0.735	0.120
237	A	0.840	0.915	0.870	0.875	0.038
238	G	0.555	0.575	0.535	0.555	0.020
239	A	0.160	0.245	0.205	0.203	0.043
240	G	0.045	0.070	0.025	0.047	0.023

hSHAPE Reactivities from 3 independent experiments (SP105*i*)						
Nucleotides		Experiment 1	Experiment 2	Experiment 3	Mean	SD
Number	Sequence					
241	C	0.100	0.105	0.110	0.105	0.005
242	G	0.150	0.225	0.330	0.235	0.090
243	G	0.235	0.315	0.340	0.297	0.055
244	A	0.390	0.390	0.435	0.405	0.026
245	A	0.420	0.440	0.515	0.458	0.050
246	C	0.205	0.130	0.155	0.163	0.038
247	G	0.180	0.165	0.240	0.195	0.040
248	G	0.195	0.200	0.115	0.170	0.048
249	A	1.810	1.525	1.685	1.673	0.143
250	C	0.000	0.000	0.000	0.000	0.000
251	U	0.000	0.000	0.000	0.000	0.000
252	C	0.110	0.135	0.145	0.130	0.018
253	A	0.070	0.250	0.310	0.210	0.125
254	C	0.140	0.020	-0.010	0.050	0.079
255	C	0.335	0.150	0.140	0.208	0.110
256	A	0.675	0.380	0.950	0.668	0.285
257	U	0.560	0.750	0.795	0.702	0.125
258	A	0.640	0.740	0.875	0.752	0.118
259	G	0.020	0.155	0.110	0.095	0.069
260	G	0.000	0.000	0.000	0.000	0.000
261	G	0.030	0.060	0.055	0.048	0.016
262	A	0.445	0.545	0.620	0.537	0.088
263	G	0.320	0.460	0.465	0.415	0.082
264	C	0.375	0.375	0.545	0.432	0.098
265	U	1.525	1.610	1.645	1.593	0.062
266	G	1.210	1.265	1.415	1.297	0.106
267	C	0.000	0.000	0.000	0.000	0.000
268	A	1.285	1.850	1.490	1.542	0.286
269	G	0.210	0.800	0.490	0.500	0.295
270	U	0.080	0.070	0.140	0.097	0.038
271	C	0.000	0.000	0.000	0.000	0.000
272	C	0.000	0.000	0.000	0.000	0.000
273	C	0.000	0.000	0.000	0.000	0.000
274	G	0.000	0.000	0.000	0.000	0.000
275	C	0.305	0.050	0.160	0.172	0.128
276	C	0.230	0.165	0.180	0.192	0.034
277	U	0.060	0.130	0.090	0.093	0.035
278	A	0.080	0.060	0.150	0.097	0.047
279	C	0.255	0.225	0.335	0.272	0.057
280	G	0.300	0.240	0.435	0.325	0.100
281	G	1.195	1.140	1.375	1.237	0.123
282	A	1.920	1.650	2.215	1.928	0.283
283	G	1.420	1.435	0.810	1.222	0.357
284	A	2.260	1.400	2.500	2.053	0.578
285	A	1.805	1.835	1.925	1.855	0.062
286	G	1.250	1.550	1.035	1.278	0.259
287	A	2.180	1.910	2.720	2.270	0.412
288	G	0.620	0.520	0.560	0.567	0.050

hSHAPE Reactivities from 3 independent experiments (SP105*i*)						
Nucleotides		Experiment 1	Experiment 2	Experiment 3	Mean	SD
Number	Sequence					
289	G	0.070	0.600	0.010	0.227	0.325
290	U	0.430	0.030	0.070	0.177	0.220
291	A	0.050	-0.050	0.055	0.018	0.059
292	G	0.000	0.000	0.000	0.000	0.000
293	G	0.260	0.030	0.060	0.117	0.125
294	U	0.955	0.485	0.320	0.587	0.329
295	U	1.105	0.600	0.580	0.762	0.298
296	A	0.640	0.705	0.790	0.712	0.075
297	C	0.380	0.555	0.590	0.508	0.113
298	G	0.000	0.000	0.000	0.000	0.000
299	G	0.000	0.000	0.000	0.000	0.000
300	U	0.000	0.000	0.000	0.000	0.000
301	G	0.100	0.070	0.110	0.093	0.021
302	A	0.000	0.000	0.000	0.000	0.000
303	G	0.000	0.000	0.000	0.000	0.000
304	C	0.520	-0.130	-0.100	0.097	0.367
305	C	0.280	-0.030	0.000	0.083	0.171
306	A	0.620	0.500	0.570	0.563	0.060
307	U	0.330	0.520	0.690	0.513	0.180
308	U	0.600	0.880	1.220	0.900	0.310
309	G	0.480	0.800	0.975	0.752	0.251
310	G	0.530	0.840	0.940	0.770	0.214
311	A	0.580	0.820	0.820	0.740	0.139
312	A	0.260	0.300	0.400	0.320	0.072
313	A	0.230	0.350	0.320	0.300	0.062
314	U	0.000	0.000	0.000	0.000	0.000
315	G	0.000	0.000	0.000	0.000	0.000
316	G	0.000	0.000	0.000	0.000	0.000
317	G	0.000	0.000	0.000	0.000	0.000
318	G	0.000	0.000	0.000	0.000	0.000
319	G	0.290	0.040	0.300	0.210	0.147
320	U	0.170	0.050	0.160	0.127	0.067
321	C	0.130	-0.010	0.030	0.050	0.072
322	U	0.020	0.030	0.110	0.053	0.049
323	C	-0.030	0.040	0.080	0.030	0.056
324	G	0.140	0.440	0.520	0.367	0.200
325	G	0.210	0.590	0.720	0.507	0.265
326	G	0.000	0.000	0.000	0.000	0.000
327	C	0.200	-0.050	0.020	0.057	0.129
328	U	0.560	0.110	0.020	0.230	0.289
329	C	1.240	0.680	-0.370	0.517	0.817
330	A	0.510	0.810	0.840	0.720	0.182
331	A	0.660	1.130	1.260	1.017	0.316
332	A	0.640	1.080	1.130	0.950	0.270
333	A	0.620	1.150	1.130	0.967	0.300
334	G	0.130	0.250	0.230	0.203	0.064
335	G	0.020	0.050	0.070	0.047	0.025
336	G	0.000	0.000	0.000	0.000	0.000

hSHAPE Reactivities from 3 independent experiments (SP105*i*)						
Nucleotides		Experiment 1	Experiment 2	Experiment 3	Mean	SD
Number	Sequence					
337	C	0.080	-0.060	0.000	0.007	0.070
338	A	0.030	0.030	0.090	0.050	0.035
339	G	0.000	0.000	0.000	0.000	0.000
340	A	0.020	0.090	0.160	0.090	0.070
341	A	0.050	0.200	0.300	0.183	0.126
342	A	-0.120	0.050	0.110	0.013	0.119
343	C	0.400	-0.120	-0.080	0.067	0.289
344	U	0.240	0.460	0.570	0.423	0.168
345	C	0.420	0.340	0.440	0.400	0.053
346	U	0.190	0.660	0.690	0.513	0.280
347	U	0.420	0.800	0.990	0.737	0.290
348	U	0.360	0.640	0.750	0.583	0.201
349	G	0.020	-0.070	0.080	0.010	0.075
350	U	0.160	0.330	0.240	0.243	0.085
351	U	0.000	0.090	0.110	0.067	0.059
352	U	0.000	0.000	0.000	0.000	0.000
353	C	0.000	0.000	0.000	0.000	0.000
354	U	0.710	0.540	0.660	0.637	0.087
355	G	0.710	1.000	1.130	0.947	0.215
356	U	0.350	0.290	0.430	0.357	0.070
357	U	0.390	0.360	0.500	0.417	0.074
358	U	0.920	0.270	0.360	0.517	0.352
359	U	0.840	0.260	0.440	0.513	0.297
360	A	0.730	0.530	0.720	0.660	0.113
361	C	0.000	0.570	-0.310	0.087	0.446
362	A	0.220	0.280	0.380	0.293	0.081
363	A	0.040	0.080	0.150	0.090	0.056
364	A	0.000	0.000	0.000	0.000	0.000
365	G	0.000	0.000	0.000	0.000	0.000
366	G	0.000	0.000	0.000	0.000	0.000
367	C	0.400	-0.030	-0.120	0.083	0.278
368	U	0.000	0.000	0.000	0.000	0.000
369	C	0.000	0.000	0.000	0.000	0.000
370	C	0.380	-0.200	0.010	0.063	0.294
371	U	0.060	0.130	0.210	0.133	0.075
372	C	0.338	0.004	0.101	0.148	0.172
373	U	0.840	0.470	0.650	0.653	0.185
374	C	2.520	1.460	1.650	1.877	0.565
375	A	1.030	2.360	2.420	1.937	0.786
376	G	0.680	1.220	1.800	1.233	0.560
377	A	0.480	0.900	1.410	0.930	0.466
378	G	0.020	-0.050	0.090	0.020	0.070
379	A	0.000	0.000	0.000	0.000	0.000
380	G	0.000	0.000	0.000	0.000	0.000
381	G	0.000	0.000	0.000	0.000	0.000
382	G	0.000	0.000	0.000	0.000	0.000
383	G	0.000	0.000	0.000	0.000	0.000
384	U	0.000	0.000	0.000	0.000	0.000

hSHAPE Reactivities from 3 independent experiments (SP105*i*)						
Nucleotides		Experiment 1	Experiment 2	Experiment 3	Mean	SD
Number	Sequence					
385	C	0.370	0.000	0.100	0.157	0.191
386	U	0.000	0.000	0.000	0.000	0.000
387	U	0.000	0.000	0.000	0.000	0.000
388	C	0.730	0.800	0.910	0.813	0.091
389	A	0.050	0.810	0.140	0.333	0.415
390	U	-0.020	0.060	-0.020	0.007	0.046
391	G	0.050	0.000	0.120	0.057	0.060
392	U	0.080	0.020	0.090	0.063	0.038
393	G	0.140	0.020	0.230	0.130	0.105
394	A	0.210	0.170	0.400	0.260	0.123
395	A	0.320	0.240	0.500	0.353	0.133
396	A	0.200	0.420	0.300	0.307	0.110
397	G	0.200	0.250	0.170	0.207	0.040
398	A	0.252	0.001	0.027	0.093	0.138
399	G	0.165	0.045	0.001	0.070	0.085
400	A	0.220	-0.030	-0.020	0.057	0.142
401	G	0.000	0.000	0.000	0.000	0.000
402	U	0.010	0.030	0.080	0.040	0.036
403	A	0.000	0.040	0.110	0.050	0.056
404	G	0.250	0.010	0.120	0.127	0.120
405	U	0.530	0.190	0.170	0.297	0.202
406	G	0.000	0.310	0.400	0.237	0.210
407	C	0.000	0.000	0.000	0.000	0.000
408	A	0.610	0.610	0.740	0.653	0.075
409	A	0.000	0.290	0.330	0.207	0.180
410	U	0.669	0.001	0.073	0.248	0.367
411	A	0.430	0.440	0.580	0.483	0.084
412	G	0.460	0.420	0.490	0.457	0.035
413	A	0.410	0.460	0.590	0.487	0.093
414	A	0.260	0.370	0.380	0.337	0.067
415	U	0.000	0.270	0.280	0.183	0.159
416	U	0.470	0.190	0.160	0.273	0.171
417	U	0.000	0.180	0.340	0.173	0.170
418	U	0.170	-0.100	0.140	0.070	0.148
419	A	0.210	0.140	0.200	0.183	0.038
420	U	0.000	0.070	-0.020	0.017	0.047
421	C	0.000	0.000	0.000	0.000	0.000
422	A	0.270	0.550	0.920	0.580	0.326
423	G	0.122	0.484	0.11	0.239	0.213
424	U	0.095	0.192	0.688	0.325	0.318
425	U	2.390	0.712	0.663	1.255	0.983
426	U	-999	-999	-999	-999	0.000
427	C	-999	-999	-999	-999	0.000
428	U	-999	-999	-999	-999	0.000
429	A	-999	-999	-999	-999	0.000
430	A	-999	-999	-999	-999	0.000
431	U	-999	-999	-999	-999	0.000
432	A	-999	-999	-999	-999	0.000

**hSHAPE Reactivities from 3 independent experiments (SP106*i*)**						
**Nucleotides**		**Experiment 1**	**Experiment 2**	**Experiment 3**	**Mean**	**SD**
**Number**	**Sequence**					
1	G	-999	-999	-999	-999	0.000
2	C	-999	-999	-999	-999	0.000
3	A	-999	-999	-999	-999	0.000
4	A	-999	-999	-999	-999	0.000
5	C	-999	-999	-999	-999	0.000
6	A	-999	-999	-999	-999	0.000
7	G	-999	-999	-999	-999	0.000
8	U	-999	-999	-999	-999	0.000
9	C	-999	-999	-999	-999	0.000
10	C	-999	-999	-999	-999	0.000
11	U	-999	-999	-999	-999	0.000
12	A	-999	-999	-999	-999	0.000
13	A	-999	-999	-999	-999	0.000
14	U	-999	-999	-999	-999	0.000
15	A	-999	-999	-999	-999	0.000
16	U	-999	-999	-999	-999	0.000
17	U	-999	-999	-999	-999	0.000
18	C	-999	-999	-999	-999	0.000
19	A	-999	-999	-999	-999	0.000
20	C	-999	-999	-999	-999	0.000
21	G	-999	-999	-999	-999	0.000
22	U	1.840	1.070	1.310	1.407	0.394
23	C	0.300	0.720	0.420	0.480	0.216
24	U	1.993	1.629	1.522	1.715	0.247
25	C	0.475	0.405	0.430	0.437	0.035
26	G	0.360	0.300	0.650	0.437	0.187
27	U	0.090	0.585	0.485	0.387	0.262
28	G	0.000	0.000	0.000	0.000	0.000
29	U	0.290	0.490	0.190	0.323	0.153
30	G	0.375	0.335	0.470	0.393	0.069
31	U	0.020	0.255	0.355	0.210	0.172
32	U	0.300	0.560	0.200	0.353	0.186
33	U	0.300	0.500	0.440	0.413	0.103
34	G	0.340	0.690	0.885	0.638	0.276
35	U	1.410	1.785	0.890	1.362	0.449
36	G	0.290	0.565	0.330	0.395	0.149
37	U	0.235	0.215	0.140	0.197	0.050
38	C	0.270	0.240	0.310	0.273	0.035
39	U	0.000	0.000	0.000	0.000	0.000
40	G	0.040	0.235	-0.020	0.085	0.133
41	U	0.060	0.265	0.090	0.138	0.111
42	U	0.540	0.830	0.775	0.715	0.154
43	C	0.455	0.865	0.495	0.605	0.226
44	G	0.995	1.850	1.175	1.340	0.451
45	C	0.000	0.000	0.000	0.000	0.000
46	C	0.000	0.000	0.000	0.000	0.000
47	A	1.170	1.515	0.695	1.127	0.412
48	U	1.095	1.085	0.980	1.053	0.064

hSHAPE Reactivities from 3 independent experiments (SP106*i*)						
Nucleotides		Experiment 1	Experiment 2	Experiment 3	Mean	SD
Number	Sequence					
49	C	0.855	0.560	0.510	0.642	0.186
50	C → G	0.940	0.970	1.020	0.977	0.040
51	C	0.110	0.170	0.190	0.157	0.042
52	G → U	0.900	1.395	1.415	1.237	0.292
53	U	0.760	0.895	0.995	0.883	0.118
54	C	0.265	0.320	0.275	0.287	0.029
55	U	0.130	0.140	0.075	0.115	0.035
56	C	0.000	0.000	0.000	0.000	0.000
57	C	0.000	0.000	0.000	0.000	0.000
58	G	0.065	0.145	0.050	0.087	0.051
59	C	0.000	0.000	0.000	0.000	0.000
60	U	0.180	0.480	0.450	0.370	0.165
61	C	0.260	0.340	0.250	0.283	0.049
62	G	0.000	0.000	0.000	0.000	0.000
63	U	0.183	0.000	0.018	0.067	0.101
64	C	0.000	0.000	0.000	0.000	0.000
65	A	0.315	0.210	0.040	0.188	0.139
66	C	0.010	-0.120	0.140	0.010	0.130
67	U	0.205	0.130	0.250	0.195	0.061
68	U	0.230	0.330	0.170	0.243	0.081
69	A	0.575	0.720	0.380	0.558	0.171
70	U	0.285	0.425	0.245	0.318	0.095
71	C	0.000	0.000	0.000	0.000	0.000
72	C	0.000	0.000	0.000	0.000	0.000
73	U	0.870	1.265	0.490	0.875	0.388
74	U	1.040	1.400	0.670	1.037	0.365
75	C	0.000	0.000	0.000	0.000	0.000
76	A	0.900	1.105	0.600	0.868	0.254
77	C	0.340	0.035	-0.255	0.040	0.298
78	U	1.065	1.235	1.155	1.152	0.085
79	U	1.405	1.505	1.440	1.450	0.051
80	U	0.670	0.520	0.535	0.575	0.083
81	C	0.000	0.000	0.000	0.000	0.000
82	C	0.000	0.000	0.000	0.000	0.000
83	A	1.955	1.490	1.990	1.812	0.279
84	G	0.260	1.710	0.605	0.858	0.757
85	A	0.010	0.230	0.180	0.140	0.115
86	G	0.000	0.000	0.000	0.000	0.000
87	G	0.000	0.000	0.000	0.000	0.000
88	G	0.000	0.000	0.000	0.000	0.000
89	U	0.000	0.000	0.000	0.000	0.000
90	C	0.000	0.000	0.000	0.000	0.000
91	C	0.030	-0.090	0.130	0.023	0.110
92	C	0.010	0.340	0.200	0.183	0.166
93	C	0.240	0.210	0.270	0.240	0.030
94	C	0.100	0.110	0.050	0.087	0.032
95	C	0.200	0.070	0.140	0.137	0.065
96	G	0.055	0.040	0.290	0.128	0.140

hSHAPE Reactivities from 3 independent experiments (SP106*i*)						
Nucleotides		Experiment 1	Experiment 2	Experiment 3	Mean	SD
Number	Sequence					
97	C	0.000	0.000	0.000	0.000	0.000
98	A	0.360	0.360	0.530	0.417	0.098
99	G	0.885	0.945	0.905	0.912	0.031
100	A	1.695	1.810	1.915	1.807	0.110
101	C	0.050	0.140	0.145	0.112	0.053
102	C	0.140	0.135	0.180	0.152	0.025
103	C	0.670	0.420	0.470	0.520	0.132
104	C	0.000	0.000	0.000	0.000	0.000
105	G	0.000	0.010	0.240	0.083	0.136
106	G	0.280	0.140	0.240	0.220	0.072
107	U	0.220	0.190	0.020	0.143	0.108
108	G	0.000	0.010	0.240	0.083	0.136
109	A	0.065	0.135	0.100	0.100	0.035
110	C	0.045	-0.060	0.060	0.015	0.065
111	C	0.435	0.150	0.195	0.260	0.153
112	C	1.090	1.060	0.860	1.003	0.125
113	U	2.805	2.330	2.700	2.612	0.250
114	C	0.635	0.510	0.510	0.552	0.072
115	A	3.575	3.360	3.450	3.462	0.108
116	G	0.165	0.175	0.145	0.162	0.015
117	G	0.000	0.010	0.240	0.083	0.136
118	U	0.000	0.010	0.240	0.083	0.136
119	C	0.115	0.500	-0.010	0.202	0.266
120	G	0.745	0.210	0.660	0.538	0.288
121	G	0.955	0.700	0.710	0.788	0.144
122	C	0.000	0.000	0.000	0.000	0.000
123	C	0.000	0.000	0.000	0.000	0.000
124	G	0.135	0.245	0.270	0.217	0.072
125	A	0.340	0.360	0.380	0.360	0.020
126	C	0.060	0.070	0.190	0.107	0.072
127	U	0.150	0.270	0.135	0.185	0.074
128	G	0.270	0.325	0.335	0.310	0.035
129	C	0.080	0.090	0.045	0.072	0.024
130	G	0.000	0.000	0.000	0.000	0.000
131	G	0.000	0.000	0.000	0.000	0.000
132	C	0.000	0.000	0.000	0.000	0.000
133	A	0.000	0.000	0.000	0.000	0.000
134	G	0.830	0.760	0.685	0.758	0.073
135	C	0.330	0.890	0.325	0.515	0.325
136	U	0.610	0.780	0.815	0.735	0.110
137	G	0.340	0.380	0.200	0.307	0.095
138	G	0.820	0.690	0.870	0.793	0.093
139	C	0.280	0.360	0.215	0.285	0.073
140	G	0.260	0.250	0.350	0.287	0.055
141	C	0.020	0.120	0.120	0.087	0.058
142	C	0.080	0.190	-0.010	0.087	0.100
143	C	0.195	0.175	0.200	0.190	0.013
144	G	0.715	0.585	0.760	0.687	0.091

hSHAPE Reactivities from 3 independent experiments (SP106*i*)						
Nucleotides		Experiment 1	Experiment 2	Experiment 3	Mean	SD
Number	Sequence					
145	A	1.175	1.195	1.010	1.127	0.102
146	A	0.000	0.000	0.000	0.000	0.000
147	C	0.000	0.000	0.000	0.000	0.000
148	A	0.400	0.170	0.240	0.270	0.118
149	G	0.000	0.000	0.000	0.000	0.000
150	G	0.000	0.000	0.000	0.000	0.000
151	G	0.000	0.000	0.000	0.000	0.000
152	A	0.000	0.000	0.000	0.000	0.000
153	C	0.130	0.870	0.000	0.333	0.469
154	C	0.040	1.210	1.440	0.897	0.751
155	C	0.120	0.510	0.250	0.293	0.199
156	U	0.210	0.210	-0.030	0.130	0.139
157	C	0.395	0.390	0.570	0.452	0.103
158	G	0.000	0.000	0.000	0.000	0.000
159	G	0.000	0.000	0.000	0.000	0.000
160	A	0.060	0.050	0.000	0.037	0.032
161	U	0.215	0.230	0.205	0.217	0.013
162	A	0.040	0.090	0.120	0.083	0.040
163	A	0.010	0.040	0.060	0.037	0.025
164	G	0.050	0.060	0.080	0.063	0.015
165	U	0.000	0.000	0.000	0.000	0.000
166	G	0.000	0.000	0.000	0.000	0.000
167	A	0.000	0.000	0.000	0.000	0.000
168	C	0.180	0.120	0.210	0.170	0.046
169	C	0.000	0.000	0.000	0.000	0.000
170	C	0.120	0.070	0.110	0.100	0.026
171	U	0.340	0.400	0.240	0.327	0.081
172	U	0.475	0.530	0.580	0.528	0.053
173	G	0.965	0.740	0.935	0.880	0.122
174	U	0.505	0.425	0.425	0.452	0.046
175	C	0.000	0.000	0.000	0.000	0.000
176	U	0.000	0.000	0.000	0.000	0.000
177	C	0.000	0.000	0.000	0.000	0.000
178	U	0.000	0.000	0.000	0.000	0.000
179	A	0.435	0.520	0.545	0.500	0.058
180	U	0.095	0.220	0.190	0.168	0.065
181	U	0.065	0.110	0.250	0.142	0.096
182	U	0.120	0.100	0.145	0.122	0.023
183	C	0.000	0.000	0.000	0.000	0.000
184	U	0.080	0.230	0.100	0.137	0.081
185	A	0.165	0.105	0.305	0.192	0.103
186	C	0.065	0.135	0.155	0.118	0.047
187	U	0.475	0.830	0.505	0.603	0.197
188	A	0.880	0.780	0.920	0.860	0.072
189	U	0.085	0.140	0.050	0.092	0.045
190	U	0.180	0.160	0.040	0.127	0.076
191	U	0.345	0.375	0.475	0.398	0.068
192	G	0.035	-0.010	0.020	0.015	0.023

hSHAPE Reactivities from 3 independent experiments (SP106*i*)						
Nucleotides		Experiment 1	Experiment 2	Experiment 3	Mean	SD
Number	Sequence					
193	G	0.450	0.480	0.450	0.460	0.017
194	U	0.595	0.640	0.810	0.682	0.113
195	G	0.845	0.865	0.980	0.897	0.073
196	U	0.640	0.600	0.655	0.632	0.028
197	U	0.820	0.690	1.005	0.838	0.158
198	U	0.375	0.480	0.585	0.480	0.105
199	G	0.001	0.029	0.152	0.061	0.080
200	U	0.020	0.030	0.110	0.053	0.049
201	C	0.140	0.020	0.010	0.057	0.072
202	U	0.130	0.205	0.150	0.162	0.039
203	U	0.185	0.255	0.480	0.307	0.154
204	G	0.140	0.245	0.270	0.218	0.069
205	U	0.130	0.215	0.145	0.163	0.045
206	A	0.255	0.265	0.315	0.278	0.032
207	U	0.270	0.320	0.340	0.310	0.036
208	U	0.425	0.475	0.650	0.517	0.118
209	G	0.760	0.615	0.805	0.727	0.099
210	U	0.155	0.180	0.160	0.165	0.013
211	C	0.000	0.000	0.000	0.000	0.000
212	U	0.000	0.000	0.000	0.000	0.000
213	C	0.000	0.000	0.000	0.000	0.000
214	U	0.000	0.000	0.000	0.000	0.000
215	U	0.060	0.135	0.110	0.102	0.038
216	U	0.065	0.150	0.150	0.122	0.049
217	C	0.090	0.035	-0.020	0.035	0.055
218	U	0.250	0.275	0.370	0.298	0.063
219	U	0.455	0.445	0.815	0.572	0.211
220	G	0.470	0.445	0.595	0.503	0.080
221	U	0.100	0.085	0.160	0.115	0.040
222	C	0.160	0.000	0.130	0.097	0.085
223	U	0.250	0.220	0.510	0.327	0.159
224	G	0.625	0.560	0.590	0.592	0.033
225	G	0.120	0.085	0.085	0.097	0.020
226	C	0.000	0.000	0.000	0.000	0.000
227	U	0.365	0.390	0.130	0.295	0.143
228	A	0.535	0.555	0.550	0.547	0.010
229	U	0.000	0.000	0.000	0.000	0.000
230	C	0.000	0.000	0.000	0.000	0.000
231	A	0.580	0.710	0.580	0.623	0.075
232	U	0.000	0.000	0.000	0.000	0.000
233	C	0.000	0.000	0.000	0.000	0.000
234	A	0.000	0.000	0.000	0.000	0.000
235	C	0.000	0.000	0.000	0.000	0.000
236	A	1.065	1.030	1.060	1.052	0.019
237	A	1.340	1.045	1.620	1.335	0.288
238	G	0.860	0.620	0.460	0.647	0.201
239	A	0.270	0.250	0.350	0.290	0.053
240	G	0.070	0.035	0.100	0.068	0.033

hSHAPE Reactivities from 3 independent experiments (SP106*i*)						
Nucleotides		Experiment 1	Experiment 2	Experiment 3	Mean	SD
Number	Sequence					
241	C	0.105	0.075	0.115	0.098	0.021
242	G	0.455	0.590	0.555	0.533	0.070
243	G	0.515	0.775	0.660	0.650	0.130
244	A	0.660	0.515	0.515	0.563	0.084
245	A	0.765	0.615	0.635	0.672	0.081
246	C	0.480	0.170	0.230	0.293	0.164
247	G	0.205	0.130	0.110	0.148	0.050
248	G	0.265	0.180	0.180	0.208	0.049
249	A	1.810	1.420	1.575	1.602	0.196
250	C	0.000	0.000	0.000	0.000	0.000
251	U	0.000	0.000	0.000	0.000	0.000
252	C	0.000	0.000	0.000	0.000	0.000
253	A	0.155	0.145	0.155	0.152	0.006
254	C	0.000	0.000	0.000	0.000	0.000
255	C	0.000	0.000	0.000	0.000	0.000
256	A	0.000	0.000	0.000	0.000	0.000
257	U	0.640	0.600	0.170	0.470	0.261
258	A	1.345	1.130	0.990	1.155	0.179
259	G	0.615	0.575	0.460	0.550	0.080
260	G	0.375	0.385	0.215	0.325	0.095
261	G	0.000	0.000	0.000	0.000	0.000
262	A	0.435	0.405	0.425	0.422	0.015
263	G	0.350	0.310	0.375	0.345	0.033
264	C	0.230	0.095	0.100	0.142	0.077
265	U	1.300	1.015	1.210	1.175	0.146
266	G	1.965	1.360	1.380	1.568	0.344
267	C	0.000	0.000	0.000	0.000	0.000
268	A	1.260	1.420	0.620	1.100	0.423
269	G	0.630	0.780	0.720	0.710	0.075
270	U	0.150	0.090	0.090	0.110	0.035
271	C	0.000	0.000	0.000	0.000	0.000
272	C	0.000	0.000	0.000	0.000	0.000
273	C	0.000	0.000	0.000	0.000	0.000
274	G	0.690	0.240	-0.080	0.283	0.387
275	C	0.000	0.000	0.000	0.000	0.000
276	C	0.200	0.165	0.120	0.162	0.040
277	U	0.275	0.215	0.050	0.180	0.117
278	A	0.190	0.080	0.235	0.168	0.080
279	C	0.555	0.440	0.475	0.490	0.059
280	G	0.460	0.380	0.490	0.443	0.057
281	G	1.355	1.070	1.245	1.223	0.144
282	A	1.980	1.560	1.905	1.815	0.224
283	G	0.655	0.475	0.670	0.600	0.109
284	A	1.945	1.765	2.230	1.980	0.234
285	A	1.535	1.245	1.535	1.438	0.167
286	G	1.065	0.855	1.155	1.025	0.154
287	A	2.255	1.945	2.045	2.082	0.158
288	G	0.750	0.540	0.695	0.662	0.109

hSHAPE Reactivities from 3 independent experiments (SP106*i*)						
Nucleotides		Experiment 1	Experiment 2	Experiment 3	Mean	SD
Number	Sequence					
289	G	0.000	0.000	0.000	0.000	0.000
290	U	0.000	0.000	0.000	0.000	0.000
291	A	0.285	0.195	0.385	0.288	0.095
292	G	0.080	0.110	0.230	0.140	0.079
293	G	0.195	0.150	0.010	0.118	0.096
294	U	0.280	-0.110	0.090	0.087	0.195
295	U	0.450	-0.015	-0.245	0.063	0.354
296	A	0.770	0.670	0.855	0.765	0.093
297	C	0.530	0.490	0.305	0.442	0.120
298	G	0.220	0.110	0.205	0.178	0.060
299	G	0.190	0.180	0.110	0.160	0.044
300	U	0.270	0.145	0.240	0.218	0.065
301	G	0.300	0.480	0.300	0.360	0.104
302	A	0.210	0.270	0.220	0.233	0.032
303	G	0.080	0.340	0.280	0.233	0.136
304	C	0.000	0.000	0.000	0.000	0.000
305	C	0.000	0.000	0.000	0.000	0.000
306	A	0.670	0.500	0.010	0.393	0.343
307	U	0.610	0.660	0.630	0.633	0.025
308	U	1.440	1.600	1.400	1.480	0.106
309	G	0.500	0.530	0.520	0.517	0.015
310	G	0.850	0.940	0.760	0.850	0.090
311	A	0.740	0.550	0.210	0.500	0.269
312	A	0.690	0.700	0.590	0.660	0.061
313	A	0.460	0.590	0.540	0.530	0.066
314	U → G	0.040	0.080	0.060	0.060	0.020
315	G	0.140	0.210	0.190	0.180	0.036
316	G → C	0.000	0.000	0.080	0.027	0.046
317	G	0.000	0.070	0.190	0.087	0.096
318	G	0.110	-0.020	0.040	0.043	0.065
319	G	0.000	0.000	0.000	0.000	0.000
320	U	0.000	0.000	0.000	0.000	0.000
321	C	0.040	0.000	0.000	0.013	0.023
322	U	0.200	0.120	0.180	0.167	0.042
323	C	0.180	0.030	0.300	0.170	0.135
324	G	0.620	0.470	0.520	0.537	0.076
325	G	0.380	0.350	0.320	0.350	0.030
326	G	0.000	0.000	0.000	0.000	0.000
327	C	0.000	0.000	0.000	0.000	0.000
328	U	0.000	0.000	0.000	0.000	0.000
329	C	0.000	0.000	0.000	0.000	0.000
330	A	0.750	0.730	0.450	0.643	0.168
331	A	1.180	1.210	1.090	1.160	0.062
332	A	1.330	1.360	1.280	1.323	0.040
333	A	1.060	1.070	1.080	1.070	0.010
334	G	0.410	0.420	0.370	0.400	0.026
335	G	0.040	0.030	0.000	0.023	0.021
336	G	0.000	0.000	0.000	0.000	0.000

hSHAPE Reactivities from 3 independent experiments (SP106*i*)						
Nucleotides		Experiment 1	Experiment 2	Experiment 3	Mean	SD
Number	Sequence					
337	C	0.000	0.000	0.000	0.000	0.000
338	A	0.030	0.030	-0.040	0.007	0.040
339	G	0.000	0.000	0.000	0.000	0.000
340	A	0.000	0.000	0.000	0.000	0.000
341	A	0.130	0.150	0.010	0.097	0.076
342	A	0.010	0.040	0.070	0.040	0.030
343	C	0.000	0.000	0.000	0.000	0.000
344	U	1.050	0.980	0.920	0.983	0.065
345	C	0.420	0.290	0.070	0.260	0.177
346	U	1.050	0.830	0.940	0.940	0.110
347	U	1.210	1.090	0.990	1.097	0.110
348	U	1.030	0.970	0.890	0.963	0.070
349	G	0.140	0.060	-0.100	0.033	0.122
350	U	0.280	0.120	-0.080	0.107	0.180
351	U	0.310	0.310	0.390	0.337	0.046
352	U	0.000	0.000	0.000	0.000	0.000
353	C	0.000	0.000	0.000	0.000	0.000
354	U	1.000	0.990	0.460	0.817	0.309
355	G	1.900	1.980	1.600	1.827	0.200
356	U	0.350	0.540	0.190	0.360	0.175
357	U	0.560	0.480	0.390	0.477	0.085
358	U	0.000	0.000	0.000	0.000	0.000
359	U	0.000	0.000	0.000	0.000	0.000
360	A	0.000	0.000	0.000	0.000	0.000
361	C	0.000	0.000	0.000	0.000	0.000
362	A	0.200	0.230	0.150	0.193	0.040
363	A	0.090	0.100	0.020	0.070	0.044
364	A	-0.060	0.050	0.040	0.010	0.061
365	G	0.000	0.000	0.000	0.000	0.000
366	G	0.000	0.000	0.000	0.000	0.000
367	C	0.000	0.000	0.000	0.000	0.000
368	U	0.000	-0.030	0.070	0.013	0.051
369	C	0.000	0.000	0.000	0.000	0.000
370	C	0.000	0.000	0.000	0.000	0.000
371	U	0.190	0.200	0.030	0.140	0.095
372	C	0.000	0.000	0.000	0.000	0.000
373	U	0.000	0.000	0.000	0.000	0.000
374	C	0.640	1.090	-0.020	0.570	0.558
375	A	2.550	2.670	2.580	2.600	0.062
376	G	1.390	1.630	1.560	1.527	0.123
377	A	1.130	1.310	1.280	1.240	0.096
378	G	0.000	0.000	0.000	0.000	0.000
379	A	0.000	0.000	0.000	0.000	0.000
380	G	0.000	0.000	0.000	0.000	0.000
381	G	0.000	0.000	0.000	0.000	0.000
382	G	0.000	0.000	0.000	0.000	0.000
383	G	0.000	0.000	0.000	0.000	0.000
384	U	0.000	0.000	0.000	0.000	0.000

hSHAPE Reactivities from 3 independent experiments (SP106*i*)						
Nucleotides		Experiment 1	Experiment 2	Experiment 3	Mean	SD
Number	Sequence					
385	C	0.000	0.000	0.000	0.000	0.000
386	U	0.000	0.000	0.000	0.000	0.000
387	U	0.000	0.000	0.000	0.000	0.000
388	C	0.000	0.000	0.000	0.000	0.000
389	A	0.580	0.850	0.260	0.563	0.295
390	U	0.110	0.220	-0.040	0.097	0.131
391	G	0.000	0.000	0.000	0.000	0.000
392	U	0.070	0.140	0.310	0.173	0.123
393	G	0.070	0.150	0.010	0.077	0.070
394	A	0.190	0.180	0.200	0.190	0.010
395	A	0.310	0.450	0.580	0.447	0.135
396	A	0.490	0.520	0.190	0.400	0.182
397	G	0.110	0.230	0.340	0.227	0.115
398	A	0.080	-0.130	0.390	0.113	0.262
399	G	-0.100	0.250	0.270	0.140	0.208
400	A	-0.110	0.110	0.180	0.060	0.151
401	G	0.000	0.000	0.000	0.000	0.000
402	U	0.000	0.000	0.000	0.000	0.000
403	A	0.000	0.000	0.000	0.000	0.000
404	G	0.000	0.000	0.000	0.000	0.000
405	U	0.000	0.000	0.000	0.000	0.000
406	G	0.000	0.000	0.000	0.000	0.000
407	C	0.000	0.000	0.000	0.000	0.000
408	A	0.370	0.250	0.000	0.207	0.189
409	A	0.300	0.430	0.000	0.243	0.221
410	U	0.000	0.000	0.000	0.000	0.000
411	A	0.620	0.650	0.180	0.483	0.263
412	G	0.410	0.540	0.480	0.477	0.065
413	A	0.610	0.720	0.390	0.573	0.168
414	A	0.400	0.530	0.390	0.440	0.078
415	U	0.340	0.420	0.370	0.377	0.040
416	U	0.240	0.330	0.450	0.340	0.105
417	U	0.140	0.510	0.000	0.217	0.264
418	U	0.000	0.360	0.500	0.287	0.258
419	A	0.240	0.240	0.000	0.160	0.139
420	U	0.000	0.000	0.000	0.000	0.000
421	C	0.000	0.000	0.000	0.000	0.000
422	A	0.310	0.190	0.280	0.260	0.062
423	G	0.560	-0.020	0.290	0.277	0.290
424	U	0.553	0.072	0.075	0.233	0.277
425	U	-999	-999	-999	-999	0.000
426	U	-999	-999	-999	-999	0.000
427	C	-999	-999	-999	-999	0.000
428	U	-999	-999	-999	-999	0.000
429	A	-999	-999	-999	-999	0.000
430	A	-999	-999	-999	-999	0.000
431	U	-999	-999	-999	-999	0.000
432	A	-999	-999	-999	-999	0.000

hSHAPE Reactivities from 3 independent experiments (SP107*i*)						
**Nucleotides**		**Experiment 1**	**Experiment 2**	**Experiment 3**	**Mean**	**SD**
**Number**	**Sequence**					
1	G	-999	-999	-999	-999	0.000
2	C	-999	-999	-999	-999	0.000
3	A	-999	-999	-999	-999	0.000
4	A	-999	-999	-999	-999	0.000
5	C	-999	-999	-999	-999	0.000
6	A	-999	-999	-999	-999	0.000
7	G	-999	-999	-999	-999	0.000
8	U	-999	-999	-999	-999	0.000
9	C	-999	-999	-999	-999	0.000
10	C	-999	-999	-999	-999	0.000
11	U	-999	-999	-999	-999	0.000
12	A	-999	-999	-999	-999	0.000
13	A	-999	-999	-999	-999	0.000
14	U	-999	-999	-999	-999	0.000
15	A	-999	-999	-999	-999	0.000
16	U	-999	-999	-999	-999	0.000
17	U	-999	-999	-999	-999	0.000
18	C	-999	-999	-999	-999	0.000
19	A	-999	-999	-999	-999	0.000
20	C	-999	-999	-999	-999	0.000
21	G	-999	-999	-999	-999	0.000
22	U	-999	-999	-999	-999	0.000
23	C	0.510	0.250	0.410	0.390	0.131
24	U	2.470	3.450	1.720	2.547	0.868
25	C	0.740	0.540	0.490	0.590	0.132
26	G	0.420	0.390	0.210	0.340	0.114
27	U	0.260	0.130	0.080	0.157	0.093
28	G	0.155	0.175	0.000	0.110	0.096
29	U	0.330	0.470	0.220	0.340	0.125
30	G	0.180	0.370	0.250	0.267	0.096
31	U	0.145	0.050	0.060	0.085	0.052
32	U	0.235	0.380	0.240	0.285	0.082
33	U	0.155	0.320	0.120	0.198	0.107
34	G	0.495	0.340	0.190	0.342	0.153
35	U	0.560	1.485	0.750	0.932	0.489
36	G	0.210	0.415	0.285	0.303	0.104
37	U	0.030	0.055	0.070	0.052	0.020
38	C	0.010	0.115	0.050	0.058	0.053
39	U	0.000	0.000	0.000	0.000	0.000
40	G	0.000	0.195	0.015	0.070	0.109
41	U	-0.025	0.150	-0.020	0.035	0.100
42	U	0.340	0.350	0.200	0.297	0.084
43	C	0.555	0.850	0.530	0.645	0.178
44	G	0.765	1.090	0.970	0.942	0.164
45	C	0.000	0.000	0.000	0.000	0.000
46	C	0.000	0.000	0.000	0.000	0.000
47	A	0.520	0.720	0.865	0.702	0.173
48	U	0.695	0.770	0.870	0.778	0.088

hSHAPE Reactivities from 3 independent experiments (SP107*i*)						
Nucleotides		Experiment 1	Experiment 2	Experiment 3	Mean	SD
Number	Sequence					
49	C	0.090	0.105	0.190	0.128	0.054
50	C	0.000	0.000	0.000	0.000	0.000
51	C	0.020	0.200	0.100	0.107	0.090
52	G	0.975	1.170	0.910	1.018	0.135
53	U	0.620	0.640	0.560	0.607	0.042
54	C	0.030	0.110	0.060	0.067	0.040
55	U	0.070	0.095	0.070	0.078	0.014
56	C → G	0.040	0.015	0.020	0.025	0.013
57	C	0.000	0.000	0.000	0.000	0.000
58	G → C	0.000	0.000	0.000	0.000	0.000
59	C	0.410	0.180	0.470	0.353	0.153
60	U→ A	0.865	0.960	0.785	0.870	0.088
61	C	0.295	0.335	0.305	0.312	0.021
62	G	0.000	0.000	0.000	0.000	0.000
63	U	-0.060	0.065	0.005	0.003	0.063
64	C	0.010	0.230	0.280	0.173	0.144
65	A	0.030	0.115	0.095	0.080	0.044
66	C	0.000	0.035	0.020	0.018	0.018
67	U	0.150	0.170	0.140	0.153	0.015
68	U	0.440	0.265	0.410	0.372	0.094
69	A	0.215	0.220	0.280	0.238	0.036
70	U	0.120	0.070	0.160	0.117	0.045
71	C	0.000	0.040	0.080	0.040	0.040
72	C	0.000	0.000	0.000	0.000	0.000
73	U	0.640	0.850	0.920	0.803	0.146
74	U	1.595	1.845	1.375	1.605	0.235
75	C	0.620	0.550	0.910	0.693	0.191
76	A	1.910	1.510	1.605	1.675	0.209
77	C	0.695	0.440	0.425	0.520	0.152
78	U	0.980	1.075	1.480	1.178	0.266
79	U	2.305	1.810	2.065	2.060	0.248
80	U	2.080	1.700	1.045	1.608	0.524
81	C	0.745	0.635	0.130	0.503	0.328
82	C	0.230	0.485	0.770	0.495	0.270
83	A	0.620	0.730	1.190	0.847	0.302
84	G	1.290	1.130	0.045	0.822	0.677
85	A	0.030	0.035	0.010	0.025	0.013
86	G	0.000	0.000	0.000	0.000	0.000
87	G	0.000	0.000	0.000	0.000	0.000
88	G	0.000	0.000	0.000	0.000	0.000
89	U	0.000	0.000	0.000	0.000	0.000
90	C	-0.025	0.045	0.010	0.010	0.035
91	C	0.000	0.000	0.000	0.000	0.000
92	C	0.000	0.000	0.000	0.000	0.000
93	C	0.000	0.000	0.000	0.000	0.000
94	C	0.000	0.000	0.000	0.000	0.000
95	C	-0.010	0.010	0.040	0.013	0.025
96	G	0.055	0.105	0.060	0.073	0.028

hSHAPE Reactivities from 3 independent experiments (SP107*i*)						
Nucleotides		Experiment 1	Experiment 2	Experiment 3	Mean	SD
Number	Sequence					
97	C	0.000	0.000	0.000	0.000	0.000
98	A	0.250	0.245	0.320	0.272	0.042
99	G	0.600	0.600	0.635	0.612	0.020
100	A	1.490	1.495	1.535	1.507	0.025
101	C	0.230	0.165	0.220	0.205	0.035
102	C	-0.060	0.220	0.150	0.103	0.146
103	C	0.000	0.000	0.000	0.000	0.000
104	C	0.000	0.000	0.000	0.000	0.000
105	G	0.000	0.000	0.000	0.000	0.000
106	G	0.000	0.000	0.000	0.000	0.000
107	U	0.045	0.085	0.085	0.072	0.023
108	G	0.000	0.000	0.000	0.000	0.000
109	A	-0.010	0.005	0.025	0.007	0.018
110	C	0.000	0.000	0.000	0.000	0.000
111	C	0.060	0.140	0.200	0.133	0.070
112	C	0.000	0.000	0.000	0.000	0.000
113	U	2.805	2.525	2.860	2.730	0.180
114	C	0.870	1.010	1.375	1.085	0.261
115	A	3.750	3.420	3.550	3.573	0.166
116	G	0.255	0.225	0.295	0.258	0.035
117	G	0.070	0.000	0.030	0.033	0.035
118	U	-0.020	0.010	0.020	0.003	0.021
119	C	0.490	0.590	0.670	0.583	0.090
120	G	0.355	0.460	0.300	0.372	0.081
121	G	0.715	0.345	0.710	0.590	0.212
122	C	0.020	0.000	0.020	0.013	0.012
123	C	0.000	0.000	0.000	0.000	0.000
124	G	0.075	0.105	0.135	0.105	0.030
125	A	0.350	0.295	0.285	0.310	0.035
126	C	0.045	0.060	0.090	0.065	0.023
127	U	0.090	0.065	0.135	0.097	0.035
128	G	0.360	0.215	0.265	0.280	0.074
129	C	0.080	0.060	0.030	0.057	0.025
130	G	0.000	0.000	0.000	0.000	0.000
131	G	0.000	0.000	0.000	0.000	0.000
132	C	0.000	0.000	0.000	0.000	0.000
133	A	0.370	0.695	0.545	0.537	0.163
134	G	0.685	0.610	0.340	0.545	0.181
135	C	0.850	1.250	1.055	1.052	0.200
136	U	0.980	0.945	0.860	0.928	0.062
137	G	0.615	0.540	0.500	0.552	0.058
138	G	0.660	0.800	0.650	0.703	0.084
139	C	0.340	0.195	0.335	0.290	0.082
140	G	0.260	0.140	0.185	0.195	0.061
141	C	0.000	0.030	0.000	0.010	0.017
142	C	0.000	0.000	0.000	0.000	0.000
143	C	0.115	-0.215	0.110	0.003	0.189
144	G	0.895	0.315	0.450	0.553	0.303

hSHAPE Reactivities from 3 independent experiments (SP107*i*)						
Nucleotides		Experiment 1	Experiment 2	Experiment 3	Mean	SD
Number	Sequence					
145	A	1.580	1.075	1.075	1.243	0.292
146	A	0.810	1.070	0.920	0.933	0.131
147	C	1.410	0.590	0.740	0.913	0.437
148	A	0.930	0.680	0.535	0.715	0.200
149	G	0.065	0.020	0.020	0.035	0.026
150	G	0.000	0.000	0.000	0.000	0.000
151	G	0.000	0.000	0.000	0.000	0.000
152	A	0.000	0.000	0.000	0.000	0.000
153	C	0.000	0.000	0.000	0.000	0.000
154	C	0.000	0.000	0.000	0.000	0.000
155	C	0.000	0.000	0.000	0.000	0.000
156	U	0.120	0.070	0.130	0.107	0.032
157	C	0.890	0.420	0.425	0.578	0.270
158	G	0.000	0.000	0.000	0.000	0.000
159	G	0.000	0.000	0.000	0.000	0.000
160	A	0.000	0.000	0.000	0.000	0.000
161	U	0.130	0.070	0.075	0.092	0.033
162	A	0.000	0.000	0.000	0.000	0.000
163	A	0.055	0.050	0.020	0.042	0.019
164	G	0.000	0.000	0.000	0.000	0.000
165	U	0.000	0.000	0.000	0.000	0.000
166	G	0.000	0.000	0.000	0.000	0.000
167	A	0.000	0.000	0.000	0.000	0.000
168	C	0.000	0.000	0.000	0.000	0.000
169	C	0.050	0.010	0.015	0.025	0.022
170	C	0.000	0.000	0.000	0.000	0.000
171	U	0.090	0.070	0.080	0.080	0.010
172	U	0.215	0.415	0.230	0.287	0.111
173	G	0.650	0.810	0.735	0.732	0.080
174	U	0.530	0.450	0.460	0.480	0.044
175	C	0.000	0.000	0.000	0.000	0.000
176	U	0.000	0.000	0.000	0.000	0.000
177	C	0.000	0.000	0.000	0.000	0.000
178	U	0.030	0.130	0.090	0.083	0.050
179	A	0.205	0.340	0.250	0.265	0.069
180	U	0.000	0.030	0.060	0.030	0.030
181	U	0.040	0.035	0.080	0.052	0.025
182	U	0.000	0.010	0.035	0.015	0.018
183	C	0.000	0.000	0.000	0.000	0.000
184	U	-0.120	-0.015	0.145	0.003	0.133
185	A	0.175	0.185	0.155	0.172	0.015
186	C	0.030	0.255	0.160	0.148	0.113
187	U	0.710	1.145	1.005	0.953	0.222
188	A	0.995	1.285	0.795	1.025	0.246
189	U	0.190	0.130	0.120	0.147	0.038
190	U	0.260	0.210	0.110	0.193	0.076
191	U	0.510	0.630	0.140	0.427	0.255
192	G	0.110	-0.140	0.060	0.010	0.132

hSHAPE Reactivities from 3 independent experiments (SP107*i*)						
Nucleotides		Experiment 1	Experiment 2	Experiment 3	Mean	SD
Number	Sequence					
193	G	0.245	0.300	0.250	0.265	0.030
194	U	0.480	0.555	0.415	0.483	0.070
195	G	0.845	0.935	0.705	0.828	0.116
196	U	0.575	0.665	0.580	0.607	0.051
197	U	0.800	0.870	0.640	0.770	0.118
198	U	0.420	0.535	0.375	0.443	0.083
199	G	0.010	0.055	0.035	0.033	0.023
200	U	0.035	0.005	0.005	0.015	0.017
201	C	0.000	0.000	0.000	0.000	0.000
202	U	0.180	0.150	0.165	0.165	0.015
203	U	0.485	0.680	0.360	0.508	0.161
204	G	0.260	0.235	0.205	0.233	0.028
205	U	0.255	0.205	0.385	0.282	0.093
206	A	0.330	0.280	0.250	0.287	0.040
207	U	0.350	0.365	0.300	0.338	0.034
208	U	0.560	0.780	0.380	0.573	0.200
209	G	1.060	1.170	0.850	1.027	0.163
210	U	0.280	0.195	0.230	0.235	0.043
211	C	0.000	0.000	0.000	0.000	0.000
212	U	0.000	0.000	0.000	0.000	0.000
213	C	0.000	0.000	0.000	0.000	0.000
214	U	0.000	0.000	0.000	0.000	0.000
215	U	0.115	0.235	0.200	0.183	0.062
216	U	0.185	0.275	0.185	0.215	0.052
217	C	0.000	0.000	0.000	0.000	0.000
218	U	0.025	0.270	0.175	0.157	0.124
219	U	0.460	0.700	0.320	0.493	0.192
220	G	0.385	0.565	0.455	0.468	0.091
221	U	0.080	0.105	0.155	0.113	0.038
222	C	0.190	0.110	0.145	0.148	0.040
223	U	0.310	0.295	0.340	0.315	0.023
224	G	0.355	0.335	0.340	0.343	0.010
225	G	0.080	0.100	0.190	0.123	0.059
226	C	0.060	0.010	0.060	0.043	0.029
227	U	1.010	0.890	1.235	1.045	0.175
228	A	0.740	0.630	0.725	0.698	0.060
229	U	0.265	0.370	0.305	0.313	0.053
230	C	0.700	0.270	0.490	0.487	0.215
231	A	0.770	0.540	0.545	0.618	0.131
232	U	0.260	0.160	0.210	0.210	0.050
233	C	0.470	0.220	0.450	0.380	0.139
234	A	0.740	0.430	0.950	0.707	0.262
235	C	0.480	0.000	0.400	0.293	0.257
236	A	0.420	0.320	0.365	0.368	0.050
237	A	0.750	0.645	0.690	0.695	0.053
238	G	0.770	0.635	0.675	0.693	0.069
239	A	0.320	0.300	0.295	0.305	0.013
240	G	0.065	0.085	0.095	0.082	0.015

hSHAPE Reactivities from 3 independent experiments (SP107*i*)						
**Nucleotides**		**Experiment 1**	**Experiment 2**	**Experiment 3**	**Mean**	**SD**
**Number**	**Sequence**					
241	C	0.210	0.140	0.230	0.193	0.047
242	G	0.640	0.635	0.625	0.633	0.008
243	G	0.690	0.600	0.715	0.668	0.060
244	A	0.495	0.290	0.675	0.487	0.193
245	A	0.505	0.440	0.545	0.497	0.053
246	C	0.145	0.245	0.255	0.215	0.061
247	G	0.250	0.380	0.290	0.307	0.067
248	G	0.070	0.075	0.355	0.167	0.163
249	A	2.160	1.830	2.020	2.003	0.166
250	C	0.000	0.000	0.000	0.000	0.000
251	U	0.000	0.000	0.000	0.000	0.000
252	C	0.130	0.040	0.110	0.093	0.047
253	A	-0.200	0.050	0.180	0.010	0.193
254	C	0.000	0.000	0.000	0.000	0.000
255	C	0.000	0.000	0.000	0.000	0.000
256	A	1.760	0.530	2.990	1.760	1.230
257	U	0.705	0.900	1.060	0.888	0.178
258	A	1.150	0.825	0.875	0.950	0.175
259	G	0.195	0.120	0.165	0.160	0.038
260	G	0.085	0.005	0.050	0.047	0.040
261	G	0.150	0.030	0.140	0.107	0.067
262	A	0.730	0.570	0.685	0.662	0.083
263	G	0.415	0.395	0.475	0.428	0.042
264	C	0.600	0.420	0.505	0.508	0.090
265	U	1.735	1.695	1.570	1.667	0.086
266	G	1.535	1.475	1.380	1.463	0.078
267	C	0.570	0.570	0.950	0.697	0.219
268	A	1.485	1.665	2.200	1.783	0.372
269	G	0.815	1.315	1.290	1.140	0.282
270	U	0.250	0.155	0.165	0.190	0.052
271	C	0.000	0.000	0.000	0.000	0.000
272	C	0.000	0.000	0.000	0.000	0.000
273	C	0.000	0.000	0.000	0.000	0.000
274	G	0.000	0.000	0.000	0.000	0.000
275	C	0.200	0.010	0.275	0.162	0.137
276	C	0.000	0.125	0.305	0.143	0.153
277	U	-0.105	0.025	0.230	0.050	0.169
278	A	0.100	0.180	0.135	0.138	0.040
279	C	0.305	0.285	0.160	0.250	0.079
280	G	0.600	0.360	0.305	0.422	0.157
281	G	1.750	1.555	1.665	1.657	0.098
282	A	2.750	2.585	2.635	2.657	0.085
283	G	1.450	0.925	1.085	1.153	0.269
284	A	3.030	3.015	3.090	3.045	0.040
285	A	2.130	2.415	2.360	2.302	0.151
286	G	1.450	1.160	0.975	1.195	0.239
287	A	2.320	2.055	2.450	2.275	0.201
288	G	0.630	0.750	0.640	0.673	0.067

hSHAPE Reactivities from 3 independent experiments (SP107*i*)						
Nucleotides		Experiment 1	Experiment 2	Experiment 3	Mean	SD
Number	Sequence					
289	G	0.020	0.100	0.020	0.047	0.046
290	U	0.000	0.000	0.000	0.000	0.000
291	A	0.000	0.000	0.000	0.000	0.000
292	G	0.000	0.000	0.000	0.000	0.000
293	G	0.020	0.000	0.045	0.022	0.023
294	U	1.060	0.610	0.830	0.833	0.225
295	U	0.860	0.950	0.790	0.867	0.080
296	A	1.085	1.150	0.910	1.048	0.124
297	C	0.750	0.870	0.640	0.753	0.115
298	G	0.000	0.000	0.000	0.000	0.000
299	G	0.000	0.000	0.000	0.000	0.000
300	U	0.230	0.250	0.220	0.233	0.015
301	G	0.250	0.110	0.160	0.173	0.071
302	A	0.000	0.000	0.000	0.000	0.000
303	G	0.000	0.000	0.000	0.000	0.000
304	C	0.250	-0.220	0.000	0.010	0.235
305	C	0.000	0.000	0.000	0.000	0.000
306	A	1.160	0.490	0.935	0.862	0.341
307	U	0.730	0.710	0.770	0.737	0.031
308	U	1.280	1.330	0.880	1.163	0.247
309	G	0.970	0.720	0.700	0.797	0.150
310	G	0.960	0.760	0.640	0.787	0.162
311	A	0.850	0.520	0.670	0.680	0.165
312	A	0.410	0.200	0.200	0.270	0.121
313	A	0.370	0.100	0.260	0.243	0.136
314	U	0.000	0.000	0.000	0.000	0.000
315	G	0.000	0.000	0.000	0.000	0.000
316	G	0.000	0.000	0.000	0.000	0.000
317	G	0.000	0.000	0.000	0.000	0.000
318	G	0.000	0.000	0.000	0.000	0.000
319	G	0.670	0.020	0.420	0.370	0.328
320	U	0.390	0.400	0.330	0.373	0.038
321	C	0.000	0.030	0.050	0.027	0.025
322	U	0.000	0.090	0.060	0.050	0.046
323	C	0.000	0.000	0.000	0.000	0.000
324	G	0.060	0.200	0.230	0.163	0.091
325	G	0.330	0.410	0.520	0.420	0.095
326	G	0.000	0.000	0.000	0.000	0.000
327	C	0.000	0.000	0.000	0.000	0.000
328	U	0.560	0.600	0.820	0.660	0.140
329	C	0.820	0.280	0.200	0.433	0.337
330	A	1.360	1.310	1.210	1.293	0.076
331	A	1.670	1.630	1.120	1.473	0.307
332	A	1.570	1.560	1.160	1.430	0.234
333	A	1.710	1.880	1.400	1.663	0.243
334	G	0.200	0.220	0.200	0.207	0.012
335	G	0.000	0.000	0.000	0.000	0.000
336	G	0.000	0.000	0.000	0.000	0.000

hSHAPE Reactivities from 3 independent experiments (SP107*i*)						
Nucleotides		Experiment 1	Experiment 2	Experiment 3	Mean	SD
Number	Sequence					
337	C	0.000	0.000	0.000	0.000	0.000
338	A	0.000	0.000	0.000	0.000	0.000
339	G	0.000	0.000	0.000	0.000	0.000
340	A	0.010	0.110	0.230	0.117	0.110
341	A	0.280	0.540	0.490	0.437	0.138
342	A	0.020	0.340	0.270	0.210	0.168
343	C	0.050	0.070	-0.010	0.037	0.042
344	U	0.270	0.540	0.520	0.443	0.150
345	C	0.150	0.290	0.210	0.217	0.070
346	U	0.220	0.480	0.480	0.393	0.150
347	U	0.340	0.720	0.630	0.563	0.199
348	U	0.310	0.650	0.490	0.483	0.170
349	G	-0.040	0.190	0.210	0.120	0.139
350	U	0.380	0.530	0.790	0.567	0.207
351	U	0.050	0.400	0.300	0.250	0.180
352	U	0.000	0.000	0.000	0.000	0.000
353	C	0.320	-0.230	-0.070	0.007	0.283
354	U	1.690	1.620	1.320	1.543	0.197
355	G	2.240	1.850	1.850	1.980	0.225
356	U	0.390	0.380	0.320	0.363	0.038
357	U	0.260	0.280	0.340	0.293	0.042
358	U	0.190	0.230	0.340	0.253	0.078
359	U	0.150	0.250	0.290	0.230	0.072
360	A	0.400	0.520	0.480	0.467	0.061
361	C	0.440	0.090	0.240	0.257	0.176
362	A	0.090	0.240	0.290	0.207	0.104
363	A	0.000	0.030	0.080	0.037	0.040
364	A	0.000	0.000	0.000	0.000	0.000
365	G	0.000	0.000	0.000	0.000	0.000
366	G	0.000	0.000	0.000	0.000	0.000
367	C	0.000	0.000	0.000	0.000	0.000
368	U	0.000	0.000	0.000	0.000	0.000
369	C	0.000	0.000	0.000	0.000	0.000
370	C	0.520	-0.170	0.360	0.237	0.361
371	U	0.350	0.250	0.570	0.390	0.164
372	C	0.250	0.000	0.280	0.177	0.154
373	U	0.810	0.730	1.100	0.880	0.195
374	C	2.560	1.830	2.300	2.230	0.370
375	A	3.480	3.090	2.700	3.090	0.390
376	G	2.210	1.940	1.740	1.963	0.236
377	A	1.670	1.850	1.230	1.583	0.319
378	G	0.000	0.000	0.000	0.000	0.000
379	A	0.000	0.000	0.000	0.000	0.000
380	G	0.000	0.000	0.000	0.000	0.000
381	G	0.040	0.000	0.000	0.013	0.023
382	G	0.410	0.230	0.000	0.213	0.206
383	G	0.000	0.000	0.000	0.000	0.000
384	U	0.000	0.000	0.000	0.000	0.000

hSHAPE Reactivities from 3 independent experiments (SP107*i*)						
Nucleotides		Experiment 1	Experiment 2	Experiment 3	Mean	SD
Number	Sequence					
385	C	0.000	0.000	0.000	0.000	0.000
386	U	0.180	-0.030	0.310	0.153	0.172
387	U	0.870	0.780	0.660	0.770	0.105
388	C	1.430	0.710	1.190	1.110	0.367
389	A	1.360	0.800	1.310	1.157	0.310
390	U	0.050	0.090	0.040	0.060	0.026
391	G	0.000	0.000	0.000	0.000	0.000
392	U	0.130	0.010	-0.010	0.043	0.076
393	G	0.120	0.000	-0.050	0.023	0.087
394	A	0.310	0.150	0.180	0.213	0.085
395	A	0.400	0.410	0.120	0.310	0.165
396	A	0.600	0.540	0.400	0.513	0.103
397	G	0.590	0.360	0.400	0.450	0.123
398	A	0.400	-0.120	0.040	0.107	0.266
399	G	0.410	0.430	0.170	0.337	0.145
400	A	0.070	0.190	0.020	0.093	0.087
401	G	0.760	0.320	0.000	0.360	0.382
402	U	0.180	0.150	0.110	0.147	0.035
403	A	0.020	0.050	0.020	0.030	0.017
404	G	0.230	0.210	0.130	0.190	0.053
405	U	0.460	0.330	0.290	0.360	0.089
406	G	-0.050	-0.210	0.330	0.023	0.277
407	C	1.140	0.480	-0.160	0.487	0.650
408	A	1.220	0.410	0.930	0.853	0.410
409	A	0.510	0.600	0.420	0.510	0.090
410	U	0.790	0.490	0.440	0.573	0.189
411	A	0.720	0.470	0.410	0.533	0.164
412	G	0.690	0.710	0.350	0.583	0.202
413	A	0.720	0.540	0.420	0.560	0.151
414	A	0.620	0.480	0.450	0.517	0.091
415	U	0.490	0.370	0.280	0.380	0.105
416	U	0.350	0.710	0.200	0.420	0.262
417	U	0.620	0.610	0.340	0.523	0.159
418	U	0.660	0.540	0.340	0.513	0.162
419	A	0.250	0.000	0.320	0.190	0.168
420	U	0.000	0.000	0.000	0.000	0.000
421	C	0.470	0.660	0.320	0.483	0.170
422	A	0.210	0.330	0.290	0.277	0.061
423	G	-999	-999	-999	-999	0.000
424	U	-999	-999	-999	-999	0.000
425	U	-999	-999	-999	-999	0.000
426	U	-999	-999	-999	-999	0.000
427	C	-999	-999	-999	-999	0.000
428	U	-999	-999	-999	-999	0.000
429	A	-999	-999	-999	-999	0.000
430	A	-999	-999	-999	-999	0.000
431	U	-999	-999	-999	-999	0.000
432	A	-999	-999	-999	-999	0.000

hSHAPE Reactivities from 3 independent experiments (SP108*i*)						
Nucleotides		Experiment 1	Experiment 2	Experiment 3	Mean	SD
Number	Sequence					
1	G	-999	-999	-999	-999	0.000
2	C	-999	-999	-999	-999	0.000
3	A	-999	-999	-999	-999	0.000
4	A	-999	-999	-999	-999	0.000
5	C	-999	-999	-999	-999	0.000
6	A	-999	-999	-999	-999	0.000
7	G	-999	-999	-999	-999	0.000
8	U	-999	-999	-999	-999	0.000
9	C	-999	-999	-999	-999	0.000
10	C	-999	-999	-999	-999	0.000
11	U	-999	-999	-999	-999	0.000
12	A	-999	-999	-999	-999	0.000
13	A	-999	-999	-999	-999	0.000
14	U	-999	-999	-999	-999	0.000
15	A	-999	-999	-999	-999	0.000
16	U	-999	-999	-999	-999	0.000
17	U	0.790	0.930	1.015	0.912	0.114
18	C	1.690	0.890	1.430	1.337	0.408
19	A	0.210	0.250	0.180	0.213	0.035
20	C	0.120	0.130	0.170	0.140	0.026
21	G	0.480	0.620	0.710	0.603	0.116
22	U	2.390	2.260	2.560	2.403	0.150
23	C	0.425	0.500	0.570	0.498	0.073
24	U	1.770	3.290	3.520	2.860	0.951
25	C	0.615	0.830	0.920	0.788	0.157
26	G	0.260	0.240	0.155	0.218	0.056
27	U	0.110	0.070	0.045	0.075	0.033
28	G	0.000	0.000	0.000	0.000	0.000
29	U	0.400	0.560	0.520	0.493	0.083
30	G	0.185	0.310	0.210	0.235	0.066
31	U	0.100	0.065	0.145	0.103	0.040
32	U	0.280	0.350	0.430	0.353	0.075
33	U	0.155	0.340	0.300	0.265	0.097
34	G	0.130	0.280	0.445	0.285	0.158
35	U	1.460	1.730	1.625	1.605	0.136
36	G	0.370	0.530	0.450	0.450	0.080
37	U	0.000	0.135	0.095	0.077	0.069
38	C	0.000	0.050	0.000	0.017	0.029
39	U	0.000	0.060	0.000	0.020	0.035
40	G	0.000	0.000	0.000	0.000	0.000
41	U	0.000	0.000	0.000	0.000	0.000
42	U	0.220	0.145	0.240	0.202	0.050
43	C	0.590	0.720	0.810	0.707	0.111
44	G	0.640	0.525	0.490	0.552	0.078
45	C	0.000	0.000	0.000	0.000	0.000
46	C	0.000	0.000	0.000	0.000	0.000
47	A	0.660	0.660	0.950	0.757	0.167
48	U	0.740	0.670	1.100	0.837	0.231

hSHAPE Reactivities from 3 independent experiments (SP108*i*)						
Nucleotides		Experiment 1	Experiment 2	Experiment 3	Mean	SD
Number	Sequence					
49	C	0.200	0.215	0.240	0.218	0.020
50	C	0.000	0.000	0.000	0.000	0.000
51	C	0.000	0.020	0.000	0.007	0.012
52	G	0.970	1.190	0.985	1.048	0.123
53	U	0.880	0.910	0.880	0.890	0.017
54	C	0.370	0.360	0.410	0.380	0.026
55	U	0.145	0.200	0.200	0.182	0.032
56	C → G	0.020	0.040	0.010	0.023	0.015
57	C	0.000	0.000	0.000	0.000	0.000
58	G → C	0.000	0.000	0.000	0.000	0.000
59	C	0.000	0.000	0.000	0.000	0.000
60	U→ A	0.810	0.715	0.450	0.658	0.187
61	C	0.120	0.200	0.000	0.107	0.101
62	G	0.000	0.000	0.000	0.000	0.000
63	U	0.170	0.065	0.000	0.078	0.086
64	C	1.495	0.650	1.100	1.082	0.423
65	A	0.330	0.380	0.345	0.352	0.026
66	C	0.000	0.000	0.000	0.000	0.000
67	U	0.290	0.255	0.500	0.348	0.133
68	U	0.570	0.665	0.605	0.613	0.048
69	A	0.570	0.320	0.450	0.447	0.125
70	U	0.200	0.160	0.225	0.195	0.033
71	C	0.100	0.200	0.060	0.120	0.072
72	C	0.000	0.000	0.000	0.000	0.000
73	U	0.550	0.800	0.280	0.543	0.260
74	U	1.720	1.790	1.780	1.763	0.038
75	C	0.000	0.000	0.000	0.000	0.000
76	A	1.830	1.785	1.535	1.717	0.159
77	C	0.450	0.415	0.235	0.367	0.115
78	U	1.115	0.910	1.190	1.072	0.145
79	U	1.790	1.560	1.575	1.642	0.129
80	U	1.080	1.020	1.090	1.063	0.038
81	C	0.240	0.375	0.335	0.317	0.069
82	C	0.200	0.850	0.600	0.550	0.328
83	A	2.170	1.880	1.810	1.953	0.191
84	G	0.070	0.125	0.110	0.102	0.028
85	A	0.015	0.020	0.105	0.047	0.051
86	G	0.000	0.000	0.000	0.000	0.000
87	G	0.000	0.000	0.000	0.000	0.000
88	G	0.000	0.000	0.000	0.000	0.000
89	U	0.000	0.000	0.000	0.000	0.000
90	C	0.000	0.000	0.000	0.000	0.000
91	C	0.000	0.000	0.000	0.000	0.000
92	C	0.000	0.000	0.000	0.000	0.000
93	C	0.000	0.000	0.000	0.000	0.000
94	C	0.000	0.000	0.000	0.000	0.000
95	C	0.000	0.000	0.000	0.000	0.000
96	G	0.130	0.150	0.055	0.112	0.050

hSHAPE Reactivities from 3 independent experiments (SP108*i*)						
Nucleotides		Experiment 1	Experiment 2	Experiment 3	Mean	SD
Number	Sequence					
97	C	0.350	0.290	0.010	0.217	0.181
98	A	0.565	0.435	0.490	0.497	0.065
99	G	0.745	0.890	0.715	0.783	0.094
100	A	2.250	1.920	2.040	2.070	0.167
101	C	0.190	0.200	0.295	0.228	0.058
102	C	0.000	0.005	0.000	0.002	0.003
103	C	0.000	0.000	0.000	0.000	0.000
104	C	0.000	0.000	0.000	0.000	0.000
105	G	0.000	0.000	0.000	0.000	0.000
106	G	0.000	0.000	0.000	0.000	0.000
107	U	0.020	0.200	0.000	0.073	0.110
108	G	0.000	0.090	0.000	0.030	0.052
109	A	0.030	0.000	0.010	0.013	0.015
110	C	0.000	0.000	0.000	0.000	0.000
111	C	0.000	0.000	0.000	0.000	0.000
112	C	0.000	0.000	0.000	0.000	0.000
113	U	1.650	1.970	2.150	1.923	0.253
114	C	1.080	0.880	0.730	0.897	0.176
115	A	2.870	3.130	3.390	3.130	0.260
116	G	0.195	0.210	0.290	0.232	0.051
117	G	0.000	0.000	0.035	0.012	0.020
118	U	0.000	0.000	0.050	0.017	0.029
119	C	0.300	0.380	0.020	0.233	0.189
120	G	0.080	0.260	0.330	0.223	0.129
121	G	0.710	0.790	0.745	0.748	0.040
122	C	0.000	0.000	0.000	0.000	0.000
123	C	0.000	0.000	0.000	0.000	0.000
124	G	0.000	0.000	0.000	0.000	0.000
125	A	0.315	0.360	0.320	0.332	0.025
126	C	0.000	0.000	0.000	0.000	0.000
127	U	0.060	0.080	0.045	0.062	0.018
128	G	0.260	0.220	0.290	0.257	0.035
129	C	0.000	0.000	0.000	0.000	0.000
130	G	0.000	0.000	0.000	0.000	0.000
131	G	0.000	0.000	0.000	0.000	0.000
132	C	0.000	0.000	0.000	0.000	0.000
133	A	0.265	0.580	0.000	0.282	0.290
134	G	0.220	0.490	0.100	0.270	0.200
135	C	0.340	0.480	0.110	0.310	0.187
136	U	0.460	0.340	0.260	0.353	0.101
137	G	0.290	0.170	0.240	0.233	0.060
138	G	0.000	0.000	0.000	0.000	0.000
139	C	0.000	0.000	0.000	0.000	0.000
140	G	0.040	0.050	0.090	0.060	0.026
141	C	0.000	0.000	0.000	0.000	0.000
142	C	0.000	0.000	0.000	0.000	0.000
143	C	0.000	0.000	0.000	0.000	0.000
144	G	0.250	0.280	0.250	0.260	0.017

hSHAPE Reactivities from 3 independent experiments (SP108*i*)						
Nucleotides		Experiment 1	Experiment 2	Experiment 3	Mean	SD
Number	Sequence					
145	A	1.260	0.860	1.180	1.100	0.212
146	A	1.320	1.320	1.560	1.400	0.139
147	C	1.890	2.435	1.450	1.925	0.493
148	A	0.860	0.710	0.920	0.830	0.108
149	G	0.020	0.010	0.000	0.010	0.010
150	G	0.000	0.000	0.000	0.000	0.000
151	G	0.000	0.000	0.000	0.000	0.000
152	A	0.000	0.010	0.000	0.003	0.006
153	C	0.000	0.000	0.000	0.000	0.000
154	C	0.780	0.510	0.000	0.430	0.396
155	C	0.000	0.000	0.000	0.000	0.000
156	U	0.000	0.000	0.000	0.000	0.000
157	C	0.160	0.165	0.160	0.162	0.003
158	G	0.000	0.000	0.000	0.000	0.000
159	G	0.000	0.000	0.000	0.000	0.000
160	A	0.000	0.000	0.000	0.000	0.000
161	U	0.185	0.140	0.120	0.148	0.033
162	A	0.000	0.000	0.000	0.000	0.000
163	A	0.000	0.005	0.005	0.003	0.003
164	G	0.000	0.000	0.000	0.000	0.000
165	U	0.000	0.000	0.000	0.000	0.000
166	G	0.000	0.000	0.000	0.000	0.000
167	A	0.000	0.000	0.000	0.000	0.000
168	C	0.000	0.000	0.000	0.000	0.000
169	C	0.050	0.050	0.000	0.033	0.029
170	C	0.000	0.000	0.000	0.000	0.000
171	U	0.120	0.090	0.000	0.070	0.062
172	U	0.350	0.275	0.275	0.300	0.043
173	G	0.660	0.840	0.750	0.750	0.090
174	U	0.485	0.550	0.510	0.515	0.033
175	C	0.000	0.000	0.000	0.000	0.000
176	U	0.000	0.000	0.000	0.000	0.000
177	C	0.000	0.000	0.000	0.000	0.000
178	U	0.465	0.655	0.290	0.470	0.183
179	A	0.320	0.340	0.380	0.347	0.031
180	U	0.080	0.080	0.170	0.110	0.052
181	U	0.085	0.045	0.065	0.065	0.020
182	U	0.085	0.065	0.070	0.073	0.010
183	C	0.000	0.080	0.030	0.037	0.040
184	U	0.535	0.590	0.600	0.575	0.035
185	A	0.125	0.065	0.035	0.075	0.046
186	C	0.135	0.095	0.000	0.077	0.069
187	U	0.985	0.880	0.680	0.848	0.155
188	A	0.855	0.820	1.080	0.918	0.141
189	U	0.185	0.105	0.235	0.175	0.066
190	U	0.120	0.020	0.120	0.087	0.058
191	U	0.165	0.110	0.155	0.143	0.029
192	G	0.050	0.020	0.000	0.023	0.025

hSHAPE Reactivities from 3 independent experiments (SP108*i*)						
**Nucleotides**		**Experiment 1**	**Experiment 2**	**Experiment 3**	**Mean**	**SD**
**Number**	**Sequence**					
193	G	0.160	0.170	0.000	0.110	0.095
194	U	0.350	0.310	0.190	0.283	0.083
195	G	0.880	0.680	0.500	0.687	0.190
196	U	0.355	0.540	0.500	0.465	0.097
197	U	0.760	0.520	0.355	0.545	0.204
198	U	0.350	0.260	0.170	0.260	0.090
199	G	0.070	0.030	0.000	0.033	0.035
200	U	0.065	0.045	0.030	0.047	0.018
201	C	0.000	0.000	0.000	0.000	0.000
202	U	0.105	0.045	0.085	0.078	0.031
203	U	0.260	0.210	0.340	0.270	0.066
204	G	0.290	0.180	0.150	0.207	0.074
205	U	0.520	0.525	0.385	0.477	0.079
206	A	0.470	0.310	0.240	0.340	0.118
207	U	0.400	0.280	0.150	0.277	0.125
208	U	0.280	0.230	0.200	0.237	0.040
209	G	0.425	0.520	0.380	0.442	0.071
210	U	0.025	0.040	0.035	0.033	0.008
211	C	0.000	0.000	0.000	0.000	0.000
212	U	0.000	0.000	0.000	0.000	0.000
213	C	0.000	0.000	0.000	0.000	0.000
214	U	0.000	0.000	0.000	0.000	0.000
215	U	0.000	0.000	0.000	0.000	0.000
216	U	0.060	0.075	0.000	0.045	0.040
217	C	0.000	0.000	0.000	0.000	0.000
218	U	0.140	0.210	0.100	0.150	0.056
219	U	0.420	0.315	0.390	0.375	0.054
220	G	0.575	0.580	0.655	0.603	0.045
221	U	0.185	0.250	0.210	0.215	0.033
222	C	0.100	0.140	0.075	0.105	0.033
223	U	0.205	0.195	0.160	0.187	0.024
224	G	0.410	0.360	0.350	0.373	0.032
225	G	0.000	0.020	0.000	0.007	0.012
226	C	0.040	0.060	0.000	0.033	0.031
227	U	0.685	0.790	0.720	0.732	0.053
228	A	0.835	0.775	1.055	0.888	0.147
229	U	0.420	0.475	0.535	0.477	0.058
230	C	0.000	0.000	0.000	0.000	0.000
231	A	0.795	0.800	0.995	0.863	0.114
232	U	0.395	0.350	0.335	0.360	0.031
233	C	1.600	0.940	0.935	1.158	0.383
234	A	0.860	1.045	0.890	0.932	0.099
235	C	2.065	1.320	1.280	1.555	0.442
236	A	1.125	1.250	1.050	1.142	0.101
237	A	0.885	0.905	0.995	0.928	0.059
238	G	0.275	0.290	0.285	0.283	0.008
239	A → U	0.330	0.250	0.300	0.293	0.040
240	G	0.035	0.000	0.000	0.012	0.020

hSHAPE Reactivities from 3 independent experiments (SP108*i*)						
Nucleotides		Experiment 1	Experiment 2	Experiment 3	Mean	SD
Number	Sequence					
241	C → G	0.000	0.000	0.000	0.000	0.000
242	G	0.000	0.000	0.000	0.000	0.000
243	G → C	0.230	0.380	0.000	0.203	0.191
244	A	0.200	0.230	0.340	0.257	0.074
245	A	0.490	0.485	0.600	0.525	0.065
246	C	0.035	0.130	0.070	0.078	0.048
247	G	0.000	0.000	0.000	0.000	0.000
248	G	0.000	0.000	0.000	0.000	0.000
249	A	2.635	2.390	2.680	2.568	0.156
250	C	0.000	0.000	0.000	0.000	0.000
251	U	0.110	0.130	0.110	0.117	0.012
252	C	0.270	0.210	0.270	0.250	0.035
253	A	0.280	0.280	0.360	0.307	0.046
254	C	0.000	0.010	0.000	0.003	0.006
255	C	0.265	0.010	0.020	0.098	0.144
256	A	0.540	0.520	0.505	0.522	0.018
257	U	0.995	1.155	0.865	1.005	0.145
258	A	1.780	1.510	1.780	1.690	0.156
259	G	0.580	0.555	0.690	0.608	0.072
260	G	0.190	0.185	0.240	0.205	0.030
261	G	0.195	0.165	0.275	0.212	0.057
262	A	0.660	0.640	0.875	0.725	0.130
263	G	0.290	0.340	0.345	0.325	0.030
264	C	0.190	0.275	0.190	0.218	0.049
265	U	0.770	0.830	0.810	0.803	0.031
266	G	0.535	0.590	0.560	0.562	0.028
267	C	0.000	0.835	0.000	0.278	0.482
268	A	0.735	0.975	0.750	0.820	0.134
269	G	0.000	0.430	0.000	0.143	0.248
270	U	0.070	0.100	0.065	0.078	0.019
271	C	0.000	0.000	0.000	0.000	0.000
272	C	0.000	0.000	0.000	0.000	0.000
273	C	0.000	0.000	0.000	0.000	0.000
274	G	0.000	0.000	0.000	0.000	0.000
275	C	0.000	0.000	0.000	0.000	0.000
276	C	0.000	0.000	0.000	0.000	0.000
277	U	0.020	0.015	0.060	0.032	0.025
278	A	0.045	0.065	0.070	0.060	0.013
279	C	0.150	0.140	0.120	0.137	0.015
280	G	0.390	0.340	0.330	0.353	0.032
281	G	1.730	1.535	1.880	1.715	0.173
282	A	2.760	2.490	3.170	2.807	0.342
283	G	0.950	1.025	1.305	1.093	0.187
284	A	2.810	2.410	3.230	2.817	0.410
285	A	2.410	2.080	2.700	2.397	0.310
286	G	1.620	1.490	1.870	1.660	0.193
287	A	3.510	2.900	3.915	3.442	0.511
288	G	1.860	1.030	1.020	1.303	0.482

hSHAPE Reactivities from 3 independent experiments (SP108*i*)						
Nucleotides		Experiment 1	Experiment 2	Experiment 3	Mean	SD
Number	Sequence					
289	G	0.105	0.000	0.000	0.035	0.061
290	U	0.000	0.000	0.000	0.000	0.000
291	A	0.080	0.060	0.050	0.063	0.015
292	G	0.540	0.460	0.680	0.560	0.111
293	G	0.000	0.230	0.200	0.143	0.125
294	U	0.000	0.340	0.610	0.317	0.306
295	U	0.090	0.470	0.980	0.513	0.447
296	A	1.030	1.040	1.580	1.217	0.315
297	C	0.875	0.815	1.090	0.927	0.145
298	G	0.000	0.010	0.020	0.010	0.010
299	G	0.010	0.050	0.010	0.023	0.023
300	U	0.150	0.150	0.150	0.150	0.000
301	G	0.060	0.070	0.000	0.043	0.038
302	A	0.000	0.000	0.035	0.012	0.020
303	G	0.000	0.000	0.000	0.000	0.000
304	C	0.000	0.000	0.000	0.000	0.000
305	C	0.000	0.000	0.000	0.000	0.000
306	A	0.610	0.210	0.310	0.377	0.208
307	U	0.240	0.610	0.830	0.560	0.298
308	U	1.150	1.030	1.230	1.137	0.101
309	G	0.920	0.750	0.920	0.863	0.098
310	G	0.930	0.720	0.940	0.863	0.124
311	A	0.550	0.580	0.710	0.613	0.085
312	A	0.350	0.390	0.470	0.403	0.061
313	A	0.320	0.300	0.410	0.343	0.059
314	U	0.440	0.290	0.260	0.330	0.096
315	G	0.010	0.000	0.000	0.003	0.006
316	G	0.220	0.000	0.000	0.073	0.127
317	G	3.800	0.000	0.000	1.267	2.194
318	G	0.000	0.000	0.000	0.000	0.000
319	G	0.000	0.000	0.000	0.000	0.000
320	U	0.000	0.000	0.000	0.000	0.000
321	C	0.000	0.000	0.000	0.000	0.000
322	U	0.210	0.200	0.210	0.207	0.006
323	C	0.200	0.130	0.000	0.110	0.101
324	G	0.570	0.310	0.230	0.370	0.178
325	G	0.060	0.110	0.120	0.097	0.032
326	G	0.000	0.000	0.000	0.000	0.000
327	C	0.000	0.000	0.000	0.000	0.000
328	U	0.000	0.300	0.210	0.170	0.154
329	C	0.000	0.980	1.550	0.843	0.784
330	A	0.880	0.820	1.050	0.917	0.119
331	A	1.610	1.310	1.660	1.527	0.189
332	A	1.630	1.290	1.600	1.507	0.188
333	A	1.520	1.250	1.520	1.430	0.156
334	G	0.230	0.220	0.220	0.223	0.006
335	G	0.000	0.000	0.000	0.000	0.000
336	G	0.000	0.000	0.000	0.000	0.000

hSHAPE Reactivities from 3 independent experiments (SP108*i*)						
Nucleotides		Experiment 1	Experiment 2	Experiment 3	Mean	SD
Number	Sequence					
337	C	0.000	0.000	0.000	0.000	0.000
338	A	0.000	0.000	0.000	0.000	0.000
339	G	0.000	0.000	0.000	0.000	0.000
340	A	0.140	0.180	0.240	0.187	0.050
341	A	0.630	0.580	0.710	0.640	0.066
342	A	0.330	0.320	0.320	0.323	0.006
343	C	0.000	0.010	0.000	0.003	0.006
344	U	0.780	0.720	0.680	0.727	0.050
345	C	0.480	0.470	0.330	0.427	0.084
346	U	0.520	0.470	0.580	0.523	0.055
347	U	1.040	0.870	1.020	0.977	0.093
348	U	0.830	0.680	0.710	0.740	0.079
349	G	0.000	0.000	0.000	0.000	0.000
350	U	0.360	0.300	0.250	0.303	0.055
351	U	0.440	0.350	0.170	0.320	0.137
352	U	0.000	0.000	0.000	0.000	0.000
353	C	0.000	0.000	0.000	0.000	0.000
354	U	1.280	1.150	1.210	1.213	0.065
355	G	3.100	2.600	3.160	2.953	0.307
356	U	0.180	0.220	0.250	0.217	0.035
357	U	0.160	0.160	0.270	0.197	0.064
358	U	0.130	0.230	0.310	0.223	0.090
359	U	0.220	0.270	0.260	0.250	0.026
360	A	0.500	0.440	0.450	0.463	0.032
361	C	0.000	0.500	0.630	0.377	0.333
362	A	0.250	0.230	0.280	0.253	0.025
363	A	0.080	0.060	0.080	0.073	0.012
364	A	0.000	0.000	0.000	0.000	0.000
365	G	0.000	0.000	0.000	0.000	0.000
366	G	0.000	0.000	0.000	0.000	0.000
367	C	0.000	0.000	0.000	0.000	0.000
368	U	0.000	0.000	0.000	0.000	0.000
369	C	0.000	0.000	0.000	0.000	0.000
370	C	0.000	0.000	0.000	0.000	0.000
371	U	0.000	0.000	0.000	0.000	0.000
372	C	0.000	0.030	0.080	0.037	0.040
373	U	0.420	0.400	0.490	0.437	0.047
374	C	1.980	2.010	2.300	2.097	0.177
375	A	4.940	3.530	5.090	4.520	0.861
376	G	2.330	1.990	2.590	2.303	0.301
377	A	2.770	2.270	3.040	2.693	0.391
378	G	0.000	0.000	0.000	0.000	0.000
379	A	0.020	0.060	0.000	0.027	0.031
380	G	0.000	0.010	0.000	0.003	0.006
381	G	0.000	0.000	0.000	0.000	0.000
382	G	0.000	0.000	0.000	0.000	0.000
383	G	0.000	0.000	0.000	0.000	0.000
384	U	0.000	0.000	0.000	0.000	0.000

hSHAPE Reactivities from 3 independent experiments (SP108*i*)						
Nucleotides		Experiment 1	Experiment 2	Experiment 3	Mean	SD
Number	Sequence					
385	C	3.640	0.300	0.000	1.313	2.021
386	U	0.000	1.110	0.150	0.420	0.602
387	U	0.070	1.780	0.900	0.917	0.855
388	C	0.000	1.110	1.940	1.017	0.973
389	A	0.920	0.230	1.050	0.733	0.441
390	U	0.090	0.050	0.000	0.047	0.045
391	G	0.020	0.060	0.000	0.027	0.031
392	U	0.040	0.000	0.000	0.013	0.023
393	G	0.050	0.020	0.000	0.023	0.025
394	A	0.080	0.110	0.040	0.077	0.035
395	A	0.190	0.530	0.140	0.287	0.212
396	A	0.590	0.510	0.510	0.537	0.046
397	G	0.600	0.150	0.680	0.477	0.286
398	A	0.170	0.120	0.100	0.130	0.036
399	G	0.130	0.210	0.280	0.207	0.075
400	A	0.220	0.080	0.030	0.110	0.098
401	G	0.030	0.760	0.000	0.263	0.430
402	U	1.040	0.000	0.330	0.457	0.531
403	A	0.590	0.000	0.690	0.427	0.373
404	G	0.260	0.020	0.200	0.160	0.125
405	U	0.360	0.140	0.170	0.223	0.119
406	G	0.000	0.120	0.500	0.207	0.261
407	C	0.000	0.810	0.970	0.593	0.520
408	A	0.000	0.340	0.210	0.183	0.172
409	A	0.220	0.310	0.330	0.287	0.059
410	U	0.230	0.410	0.200	0.280	0.114
411	A	0.290	0.270	0.200	0.253	0.047
412	G	0.160	0.200	0.080	0.147	0.061
413	A	0.250	0.260	0.200	0.237	0.032
414	A	0.270	0.270	0.240	0.260	0.017
415	U	0.200	0.220	0.190	0.203	0.015
416	U	0.080	0.000	0.000	0.027	0.046
417	U	0.020	0.320	0.120	0.153	0.153
418	U	0.260	0.580	0.200	0.347	0.204
419	A	0.000	0.120	0.490	0.203	0.255
420	U	0.000	0.200	0.000	0.067	0.115
421	C	0.000	0.520	0.170	0.230	0.265
422	A	0.060	0.250	0.180	0.163	0.096
423	G	0.180	0.180	0.080	0.147	0.058
424	U	0.040	0.060	0.100	0.067	0.031
425	U	0.050	0.000	0.050	0.033	0.029
426	U	0.000	0.170	0.060	0.077	0.086
427	C	-999	-999	-999	-999	0.000
428	U	-999	-999	-999	-999	0.000
429	A	-999	-999	-999	-999	0.000
430	A	-999	-999	-999	-999	0.000
431	U	-999	-999	-999	-999	0.000
432	A	-999	-999	-999	-999	0.000

**hSHAPE Reactivities from 3 independent experiments (SP109*i*)**						
**Nucleotides**		**Experiment 1**	**Experiment 2**	**Experiment 3**	**Mean**	**SD**
**Number**	**Sequence**					
1	G	-999	-999	-999	-999	0.000
2	C	-999	-999	-999	-999	0.000
3	A	-999	-999	-999	-999	0.000
4	A	-999	-999	-999	-999	0.000
5	C	-999	-999	-999	-999	0.000
6	A	-999	-999	-999	-999	0.000
7	G	-999	-999	-999	-999	0.000
8	U	-999	-999	-999	-999	0.000
9	C	-999	-999	-999	-999	0.000
10	C	-999	-999	-999	-999	0.000
11	U	-999	-999	-999	-999	0.000
12	A	-999	-999	-999	-999	0.000
13	A	-999	-999	-999	-999	0.000
14	U	-999	-999	-999	-999	0.000
15	A	-999	-999	-999	-999	0.000
16	U	-999	-999	-999	-999	0.000
17	U	-999	-999	-999	-999	0.000
18	C	-999	-999	-999	-999	0.000
19	A	0.215	0.105	0.220	0.180	0.065
20	C	0.150	0.260	0.130	0.180	0.070
21	G	0.630	0.625	0.450	0.568	0.103
22	U	1.920	2.045	1.970	1.978	0.063
23	C	0.545	0.640	0.530	0.572	0.060
24	U	2.670	2.880	2.975	2.842	0.156
25	C	0.590	0.685	0.665	0.647	0.050
26	G	0.120	0.260	0.085	0.155	0.093
27	U	0.085	0.000	0.000	0.028	0.049
28	G	0.000	0.000	0.000	0.000	0.000
29	U	0.240	0.375	0.315	0.310	0.068
30	G	0.370	0.340	0.305	0.338	0.033
31	U	0.150	0.120	0.040	0.103	0.057
32	U	0.560	0.425	0.345	0.443	0.109
33	U	0.280	0.260	0.305	0.282	0.023
34	G	0.330	0.420	0.430	0.393	0.055
35	U	1.670	1.780	2.250	1.900	0.308
36	G	0.500	0.430	0.780	0.570	0.185
37	U	0.170	0.070	0.265	0.168	0.098
38	C	0.040	0.000	0.170	0.070	0.089
39	U	0.000	0.000	0.145	0.048	0.084
40	G	0.000	0.000	0.015	0.005	0.009
41	U	0.030	0.230	0.110	0.123	0.101
42	U	0.435	0.470	0.325	0.410	0.076
43	C	1.005	0.815	0.825	0.882	0.107
44	G	0.620	0.360	0.530	0.503	0.132
45	C	0.000	0.000	0.000	0.000	0.000
46	C	0.000	0.000	0.000	0.000	0.000
47	A	1.065	1.065	1.400	1.177	0.193
48	U	1.275	1.605	1.705	1.528	0.225

hSHAPE Reactivities from 3 independent experiments (SP109*i*)						
Nucleotides		Experiment 1	Experiment 2	Experiment 3	Mean	SD
Number	Sequence					
49	C	0.880	1.000	1.240	1.040	0.183
50	C → G	0.090	0.110	0.150	0.117	0.031
51	C	0.130	0.120	0.230	0.160	0.061
52	G → U	0.375	0.350	0.280	0.335	0.049
53	U	0.570	0.395	0.535	0.500	0.093
54	C	0.490	0.405	0.580	0.492	0.088
55	U	0.035	0.045	0.000	0.027	0.024
56	C	0.000	0.000	0.000	0.000	0.000
57	C	0.000	0.000	0.000	0.000	0.000
58	G	0.000	0.000	0.000	0.000	0.000
59	C	0.000	0.000	0.000	0.000	0.000
60	U	0.040	0.120	0.240	0.133	0.101
61	C	0.150	0.180	0.430	0.253	0.154
62	G	0.000	0.000	0.100	0.033	0.058
63	U	0.210	0.230	0.370	0.270	0.087
64	C	0.000	0.000	0.000	0.000	0.000
65	A	0.250	0.255	0.500	0.335	0.143
66	C	0.000	0.000	0.010	0.003	0.006
67	U	0.275	0.350	0.200	0.275	0.075
68	U	0.570	0.440	0.540	0.517	0.068
69	A	0.400	0.550	0.420	0.457	0.081
70	U	0.210	0.240	0.220	0.223	0.015
71	C	0.035	0.035	0.020	0.030	0.009
72	C	0.000	0.035	0.045	0.027	0.024
73	U	0.885	1.355	1.160	1.133	0.236
74	U	1.445	1.740	1.680	1.622	0.156
75	C	0.000	0.000	0.000	0.000	0.000
76	A	1.330	1.275	1.685	1.430	0.223
77	C	0.600	0.560	0.545	0.568	0.028
78	U	1.270	1.020	1.080	1.123	0.131
79	U	1.830	1.350	1.315	1.498	0.288
80	U	0.685	0.475	0.625	0.595	0.108
81	C	0.775	0.425	0.350	0.517	0.227
82	C	0.100	0.010	0.440	0.183	0.227
83	A	1.980	1.610	1.510	1.700	0.248
84	G	1.740	2.070	1.700	1.837	0.203
85	A	0.035	0.030	0.020	0.028	0.008
86	G	0.000	0.000	0.000	0.000	0.000
87	G	0.000	0.000	0.000	0.000	0.000
88	G	0.000	0.000	0.000	0.000	0.000
89	U	0.000	0.000	0.000	0.000	0.000
90	C	0.030	0.090	0.040	0.053	0.032
91	C	0.000	0.000	0.000	0.000	0.000
92	C	0.000	0.000	0.000	0.000	0.000
93	C	0.000	0.000	0.000	0.000	0.000
94	C	0.000	0.000	0.000	0.000	0.000
95	C	0.000	0.000	0.000	0.000	0.000
96	G	0.000	0.000	0.010	0.003	0.006

hSHAPE Reactivities from 3 independent experiments (SP109*i*)						
Nucleotides		Experiment 1	Experiment 2	Experiment 3	Mean	SD
Number	Sequence					
97	C	0.050	0.020	0.295	0.122	0.151
98	A	0.180	0.375	0.285	0.280	0.098
99	G	0.615	0.750	0.670	0.678	0.068
100	A	1.630	1.760	1.860	1.750	0.115
101	C	0.135	0.210	0.200	0.182	0.041
102	C	0.220	0.240	0.315	0.258	0.050
103	C	0.000	0.000	0.000	0.000	0.000
104	C	0.000	0.000	0.000	0.000	0.000
105	G	0.080	0.060	0.000	0.047	0.042
106	G	0.000	0.000	0.000	0.000	0.000
107	U	0.200	0.180	0.110	0.163	0.047
108	G	0.070	0.035	0.000	0.035	0.035
109	A	0.000	0.050	0.010	0.020	0.026
110	C	0.000	0.000	0.000	0.000	0.000
111	C	0.000	0.000	0.000	0.000	0.000
112	C	0.000	0.000	0.000	0.000	0.000
113	U	1.660	2.180	1.780	1.873	0.272
114	C	0.465	1.215	1.110	0.930	0.406
115	A	3.210	3.230	3.000	3.147	0.127
116	G	0.050	0.100	0.070	0.073	0.025
117	G	0.000	0.000	0.000	0.000	0.000
118	U	0.010	0.000	0.080	0.030	0.044
119	C	0.320	0.370	0.540	0.410	0.115
120	G	0.360	0.550	0.290	0.400	0.135
121	G	0.430	0.670	0.995	0.698	0.284
122	C	0.000	0.000	0.105	0.035	0.061
123	C	0.000	0.000	0.000	0.000	0.000
124	G	0.030	0.170	0.070	0.090	0.072
125	A	0.405	0.355	0.435	0.398	0.040
126	C	0.000	0.020	0.030	0.017	0.015
127	U	0.200	0.170	0.070	0.147	0.068
128	G	0.290	0.100	0.110	0.167	0.107
129	C	0.000	0.000	0.000	0.000	0.000
130	G	0.000	0.000	0.000	0.000	0.000
131	G	0.000	0.000	0.000	0.000	0.000
132	C	0.000	0.000	0.105	0.035	0.061
133	A	0.350	0.465	0.470	0.428	0.068
134	G	0.220	0.525	0.600	0.448	0.201
135	C	0.500	0.910	0.990	0.800	0.263
136	U	0.770	0.680	0.600	0.683	0.085
137	G	0.130	0.240	0.110	0.160	0.070
138	G	0.000	0.000	0.000	0.000	0.000
139	C	0.000	0.015	0.000	0.005	0.009
140	G	0.010	0.100	0.015	0.042	0.051
141	C	0.000	0.000	0.000	0.000	0.000
142	C	0.000	0.000	0.000	0.000	0.000
143	C	0.000	0.000	0.000	0.000	0.000
144	G	0.390	0.590	0.440	0.473	0.104

hSHAPE Reactivities from 3 independent experiments (SP109*i*)						
Nucleotides		Experiment 1	Experiment 2	Experiment 3	Mean	SD
Number	Sequence					
145	A	1.510	1.620	1.330	1.487	0.146
146	A	1.150	1.200	1.330	1.227	0.093
147	C	1.590	1.110	1.640	1.447	0.293
148	A	0.510	0.580	0.700	0.597	0.096
149	G	0.000	0.000	0.000	0.000	0.000
150	G	0.000	0.000	0.000	0.000	0.000
151	G	0.000	0.000	0.000	0.000	0.000
152	A	0.000	0.000	0.000	0.000	0.000
153	C	0.000	0.000	0.000	0.000	0.000
154	C	0.000	0.000	0.000	0.000	0.000
155	C	0.000	0.000	0.000	0.000	0.000
156	U	0.000	0.000	0.000	0.000	0.000
157	C	0.150	0.225	0.130	0.168	0.050
158	G	0.000	0.000	0.000	0.000	0.000
159	G	0.000	0.000	0.000	0.000	0.000
160	A	0.000	0.000	0.000	0.000	0.000
161	U	0.400	0.390	0.680	0.490	0.165
162	A	0.000	0.000	0.000	0.000	0.000
163	A	0.000	0.040	0.130	0.057	0.067
164	G	0.000	0.000	0.000	0.000	0.000
165	U	0.000	0.000	0.000	0.000	0.000
166	G	0.000	0.000	0.000	0.000	0.000
167	A	0.000	0.000	0.000	0.000	0.000
168	C	0.000	0.000	0.000	0.000	0.000
169	C	0.000	0.035	0.000	0.012	0.020
170	C	0.000	0.000	0.000	0.000	0.000
171	U	0.250	0.235	0.175	0.220	0.040
172	U	0.310	0.410	0.610	0.443	0.153
173	G	0.840	0.920	0.645	0.802	0.141
174	U	0.230	0.375	0.570	0.392	0.171
175	C	0.000	0.000	0.000	0.000	0.000
176	U	0.000	0.000	0.000	0.000	0.000
177	C	0.000	0.000	0.000	0.000	0.000
178	U	0.000	0.100	0.000	0.033	0.058
179	A	0.540	0.610	0.190	0.447	0.225
180	U	0.250	0.280	0.000	0.177	0.154
181	U	0.255	0.180	0.005	0.147	0.128
182	U	0.165	0.175	0.040	0.127	0.075
183	C	0.000	0.045	0.000	0.015	0.026
184	U	0.150	0.280	0.335	0.255	0.095
185	A	0.250	0.245	0.180	0.225	0.039
186	C	0.100	0.120	0.140	0.120	0.020
187	U	0.390	0.530	0.490	0.470	0.072
188	A	0.430	0.540	0.425	0.465	0.065
189	U	0.110	0.130	0.130	0.123	0.012
190	U	0.100	0.070	0.080	0.083	0.015
191	U	0.030	0.050	0.045	0.042	0.010
192	G	0.000	0.000	0.000	0.000	0.000

hSHAPE Reactivities from 3 independent experiments (SP109*i*)						
Nucleotides		Experiment 1	Experiment 2	Experiment 3	Mean	SD
Number	Sequence					
193	G	0.000	0.000	0.000	0.000	0.000
194	U	0.000	0.000	0.000	0.000	0.000
195	G	0.010	0.220	0.550	0.260	0.272
196	U	0.080	0.190	0.560	0.277	0.251
197	U	0.260	0.300	0.600	0.387	0.186
198	U	0.020	0.080	0.245	0.115	0.117
199	G	0.000	0.000	0.005	0.002	0.003
200	U	0.060	0.110	0.255	0.142	0.101
201	C	0.000	0.000	0.000	0.000	0.000
202	U	0.330	0.300	0.230	0.287	0.051
203	U	0.230	0.320	0.390	0.313	0.080
204	G	0.140	0.300	0.320	0.253	0.099
205	U	0.330	0.550	0.225	0.368	0.166
206	A	0.050	0.105	0.145	0.100	0.048
207	U	0.070	0.080	0.155	0.102	0.046
208	U	0.225	0.365	0.355	0.315	0.078
209	G	0.800	0.885	1.065	0.917	0.135
210	U	0.110	0.165	0.210	0.162	0.050
211	C	0.000	0.000	0.000	0.000	0.000
212	U	0.000	0.000	0.000	0.000	0.000
213	C	0.000	0.000	0.000	0.000	0.000
214	U	0.000	0.000	0.000	0.000	0.000
215	U	0.010	0.160	0.005	0.058	0.088
216	U	0.110	0.150	0.000	0.087	0.078
217	C	0.000	0.000	0.000	0.000	0.000
218	U	0.120	0.105	0.070	0.098	0.026
219	U	0.135	0.250	0.145	0.177	0.064
220	G	0.360	0.470	0.320	0.383	0.078
221	U	0.100	0.180	0.125	0.135	0.041
222	C	0.170	0.180	0.170	0.173	0.006
223	U	0.220	0.285	0.210	0.238	0.041
224	G	0.000	0.280	0.150	0.143	0.140
225	G	0.000	0.000	0.000	0.000	0.000
226	C	0.000	0.000	0.000	0.000	0.000
227	U	1.020	1.200	0.850	1.023	0.175
228	A	1.125	0.890	0.990	1.002	0.118
229	U	0.500	0.430	0.700	0.543	0.140
230	C	0.000	0.000	0.000	0.000	0.000
231	A	0.850	0.950	0.910	0.903	0.050
232	U	0.230	0.220	0.380	0.277	0.090
233	C	0.000	0.000	0.000	0.000	0.000
234	A	0.555	0.510	0.830	0.632	0.173
235	C	0.000	0.000	0.000	0.000	0.000
236	A	0.760	0.740	0.855	0.785	0.061
237	A	0.560	0.570	0.780	0.637	0.124
238	G	0.670	0.770	0.780	0.740	0.061
239	A	0.270	0.275	0.460	0.335	0.108
240	G	0.100	0.075	0.070	0.082	0.016

hSHAPE Reactivities from 3 independent experiments (SP109*i*)						
Nucleotides		Experiment 1	Experiment 2	Experiment 3	Mean	SD
Number	Sequence					
241	C	0.170	0.165	0.110	0.148	0.033
242	G	0.000	0.000	0.000	0.000	0.000
243	G	0.000	0.000	0.000	0.000	0.000
244	A	1.280	1.040	0.550	0.957	0.372
245	A	0.415	0.530	0.380	0.442	0.078
246	C → A	0.335	0.440	0.430	0.402	0.058
247	G	0.000	0.000	0.000	0.000	0.000
248	G → C	0.000	0.000	0.000	0.000	0.000
249	A	0.000	0.000	0.020	0.007	0.012
250	C	0.000	0.000	0.000	0.000	0.000
251	U	0.000	0.000	0.025	0.008	0.014
252	C	0.155	0.290	0.170	0.205	0.074
253	A	0.165	0.220	0.235	0.207	0.037
254	C	0.000	0.000	0.000	0.000	0.000
255	C	0.270	0.345	0.305	0.307	0.038
256	A	0.800	0.745	0.620	0.722	0.092
257	U	1.160	1.350	1.085	1.198	0.137
258	A	1.680	1.600	1.970	1.750	0.195
259	G	0.065	0.060	0.090	0.072	0.016
260	G	0.000	0.000	0.050	0.017	0.029
261	G	0.000	0.000	0.000	0.000	0.000
262	A	0.135	0.190	0.210	0.178	0.039
263	G	0.050	0.150	0.210	0.137	0.081
264	C	0.210	0.320	0.305	0.278	0.060
265	U	0.310	0.380	0.375	0.355	0.039
266	G	0.170	0.320	0.230	0.240	0.075
267	C	0.000	0.000	0.000	0.000	0.000
268	A	0.140	0.660	0.360	0.387	0.261
269	G	0.000	0.000	0.000	0.000	0.000
270	U	0.020	0.130	0.105	0.085	0.058
271	C	0.045	0.040	0.140	0.075	0.056
272	C	0.000	0.000	0.000	0.000	0.000
273	C	0.000	0.000	0.000	0.000	0.000
274	G	0.000	0.000	0.000	0.000	0.000
275	C	0.000	0.000	0.000	0.000	0.000
276	C	0.010	0.020	0.100	0.043	0.049
277	U	0.050	0.110	0.110	0.090	0.035
278	A	0.065	0.035	0.090	0.063	0.028
279	C	0.200	0.280	0.290	0.257	0.049
280	G	0.310	0.330	0.400	0.347	0.047
281	G	1.920	1.900	1.990	1.937	0.047
282	A	2.930	2.960	3.110	3.000	0.096
283	G	1.090	1.090	1.230	1.137	0.081
284	A	2.730	2.730	2.630	2.697	0.058
285	A	1.820	1.810	1.940	1.857	0.072
286	G	1.560	1.540	1.540	1.547	0.012
287	A	3.090	3.010	2.820	2.973	0.139
288	G	1.400	1.830	1.630	1.620	0.215

hSHAPE Reactivities from 3 independent experiments (SP109*i*)						
Nucleotides		Experiment 1	Experiment 2	Experiment 3	Mean	SD
Number	Sequence					
289	G	0.000	0.100	0.250	0.117	0.126
290	U	0.000	0.000	0.000	0.000	0.000
291	A	0.030	0.050	0.100	0.060	0.036
292	G	0.000	0.000	0.000	0.000	0.000
293	G	0.000	0.000	0.000	0.000	0.000
294	U	0.690	0.680	0.590	0.653	0.055
295	U	0.850	1.100	1.220	1.057	0.189
296	A	1.830	1.680	1.790	1.767	0.078
297	C	1.370	1.190	1.450	1.337	0.133
298	G	0.700	0.180	0.670	0.517	0.292
299	G	0.080	0.120	0.130	0.110	0.026
300	U	0.020	0.070	0.090	0.060	0.036
301	G	0.000	0.000	0.000	0.000	0.000
302	A	0.000	0.000	0.000	0.000	0.000
303	G	0.000	0.000	0.000	0.000	0.000
304	C	0.000	0.000	0.000	0.000	0.000
305	C	0.140	0.210	0.510	0.287	0.197
306	A	1.280	1.280	1.190	1.250	0.052
307	U	1.510	1.420	1.460	1.463	0.045
308	U	1.760	1.680	1.780	1.740	0.053
309	G	0.700	0.670	0.680	0.683	0.015
310	G	0.610	0.610	0.620	0.613	0.006
311	A	0.540	0.520	0.490	0.517	0.025
312	A	0.360	0.370	0.440	0.390	0.044
313	A	0.420	0.410	0.490	0.440	0.044
314	U	0.430	0.430	0.610	0.490	0.104
315	G	0.000	0.000	0.030	0.010	0.017
316	G	0.000	0.000	0.000	0.000	0.000
317	G	0.000	0.000	0.000	0.000	0.000
318	G	0.000	0.000	0.000	0.000	0.000
319	G	0.000	0.000	0.000	0.000	0.000
320	U	0.260	0.300	0.510	0.357	0.134
321	C	0.000	0.000	0.010	0.003	0.006
322	U	0.150	0.170	0.260	0.193	0.059
323	C	0.020	0.070	0.110	0.067	0.045
324	G	0.100	0.210	0.170	0.160	0.056
325	G	0.000	0.000	0.000	0.000	0.000
326	G	0.000	0.000	0.000	0.000	0.000
327	C	0.000	0.000	0.000	0.000	0.000
328	U	0.030	0.290	0.050	0.123	0.145
329	C	0.000	0.000	0.000	0.000	0.000
330	A	0.850	0.890	0.890	0.877	0.023
331	A	1.310	1.240	1.430	1.327	0.096
332	A	1.110	1.120	1.140	1.123	0.015
333	A	0.850	0.830	0.910	0.863	0.042
334	G	0.160	0.160	0.230	0.183	0.040
335	G	0.000	0.000	0.110	0.037	0.064
336	G	0.110	0.170	0.200	0.160	0.046

hSHAPE Reactivities from 3 independent experiments (SP109*i*)						
Nucleotides		Experiment 1	Experiment 2	Experiment 3	Mean	SD
Number	Sequence					
337	C	0.350	0.490	0.420	0.420	0.070
338	A	0.090	0.210	0.130	0.143	0.061
339	G	0.000	0.110	0.130	0.080	0.070
340	A	0.110	0.260	0.130	0.167	0.081
341	A	0.340	0.610	0.450	0.467	0.136
342	A	0.000	0.220	0.080	0.100	0.111
343	C	0.000	0.100	0.000	0.033	0.058
344	U	1.440	1.510	1.760	1.570	0.168
345	C	0.490	0.390	0.980	0.620	0.316
346	U	0.760	0.540	0.760	0.687	0.127
347	U	1.230	1.090	1.240	1.187	0.084
348	U	1.180	1.050	1.200	1.143	0.081
349	G	0.000	0.000	0.020	0.007	0.012
350	U	0.320	0.360	0.270	0.317	0.045
351	U	0.400	0.400	0.460	0.420	0.035
352	U	0.000	0.000	0.000	0.000	0.000
353	C	0.000	0.000	0.000	0.000	0.000
354	U	0.980	0.950	0.890	0.940	0.046
355	G	3.660	3.420	3.850	3.643	0.215
356	U	0.290	0.360	0.180	0.277	0.091
357	U	0.410	0.460	0.280	0.383	0.093
358	U	0.420	0.440	0.270	0.377	0.093
359	U	0.370	0.440	0.160	0.323	0.146
360	A	0.430	0.480	0.510	0.473	0.040
361	C	0.000	0.000	0.000	0.000	0.000
362	A	0.110	0.240	0.330	0.227	0.111
363	A	0.000	0.170	0.140	0.103	0.091
364	A	0.000	0.100	0.040	0.047	0.050
365	G	0.000	0.000	0.000	0.000	0.000
366	G	0.000	0.000	0.000	0.000	0.000
367	C	0.000	0.000	0.000	0.000	0.000
368	U	0.000	0.000	0.000	0.000	0.000
369	C	0.000	0.000	0.000	0.000	0.000
370	C	0.000	0.000	0.000	0.000	0.000
371	U	0.000	0.000	0.000	0.000	0.000
372	C	0.070	0.000	0.030	0.033	0.035
373	U	0.660	0.600	0.360	0.540	0.159
374	C	3.110	2.940	1.630	2.560	0.810
375	A	3.630	3.360	4.680	3.890	0.697
376	G	3.100	2.620	3.330	3.017	0.362
377	A	3.770	3.500	4.390	3.887	0.456
378	G	0.000	0.000	0.000	0.000	0.000
379	A	0.000	0.000	0.000	0.000	0.000
380	G	0.000	0.000	0.160	0.053	0.092
381	G	0.000	0.000	0.000	0.000	0.000
382	G	0.000	0.000	6.160	2.053	3.556
383	G	0.120	0.000	0.000	0.040	0.069
384	U	0.000	0.000	0.000	0.000	0.000

hSHAPE Reactivities from 3 independent experiments (SP109*i*)						
Nucleotides		Experiment 1	Experiment 2	Experiment 3	Mean	SD
Number	Sequence					
385	C	0.040	0.060	0.000	0.033	0.031
386	U	0.590	0.410	0.110	0.370	0.242
387	U	0.000	0.000	0.650	0.217	0.375
388	C	1.520	1.310	1.110	1.313	0.205
389	A	0.050	0.010	1.400	0.487	0.791
390	U	0.000	0.000	0.130	0.043	0.075
391	G	0.010	0.000	0.040	0.017	0.021
392	U	0.020	0.000	0.000	0.007	0.012
393	G	0.060	0.000	0.000	0.020	0.035
394	A	0.150	0.110	0.000	0.087	0.078
395	A	0.550	0.400	0.380	0.443	0.093
396	A	0.950	0.830	0.370	0.717	0.306
397	G	0.200	0.170	0.830	0.400	0.373
398	A	0.350	0.190	0.120	0.220	0.118
399	G	0.390	0.230	0.100	0.240	0.145
400	A	0.360	0.140	0.290	0.263	0.112
401	G	1.010	0.710	0.450	0.723	0.280
402	U	0.990	0.560	0.250	0.600	0.372
403	A	0.410	0.260	0.120	0.263	0.145
404	G	0.510	0.050	0.030	0.197	0.272
405	U	0.000	0.000	0.020	0.007	0.012
406	G	0.000	0.000	0.150	0.050	0.087
407	C	0.040	0.220	0.000	0.087	0.117
408	A	0.420	0.540	0.400	0.453	0.076
409	A	0.490	0.490	0.390	0.457	0.058
410	U	0.310	0.640	0.420	0.457	0.168
411	A	0.380	0.250	0.400	0.343	0.081
412	G	0.440	0.500	0.220	0.387	0.147
413	A	0.570	0.510	0.320	0.467	0.131
414	A	0.640	0.550	0.230	0.473	0.215
415	U	0.340	0.220	0.350	0.303	0.072
416	U	0.720	0.740	0.370	0.610	0.208
417	U	0.700	0.650	0.490	0.613	0.110
418	U	1.020	0.500	0.650	0.723	0.268
419	A	0.320	0.200	1.070	0.530	0.471
420	U	0.740	0.620	0.550	0.637	0.096
421	C	0.730	0.810	0.850	0.797	0.061
422	A	0.420	0.400	0.210	0.343	0.116
423	G	0.280	0.280	0.350	0.303	0.040
424	U	0.250	0.040	0.520	0.270	0.241
425	U	0.240	0.410	0.340	0.330	0.085
426	U	0.150	0.260	0.150	0.187	0.064
427	C	-999	-999	-999	-999	0.000
428	U	-999	-999	-999	-999	0.000
429	A	-999	-999	-999	-999	0.000
430	A	-999	-999	-999	-999	0.000
431	U	-999	-999	-999	-999	0.000
432	A	-999	-999	-999	-999	0.000
