# Supplementary material for: MMTV RNA packaging requires an extended long-range interaction for productive Gag binding to packaging signals
Source: PLoS Biol. 2024 Oct 3;22(10):e3002827. doi: 10.1371/journal.pbio.3002827 (PMC11449360; doi:10.1371/journal.pbio.3002827)
Supplement: S2 Table — (PDF) [file pbio.3002827.s012.pdf]

Supplementary Table 2 (pages 1-3)

Primers used for introduction of mutations and construction of clones						
**Mutation Names**	**Primers**	**Region where mutations were introduced/ description**	***S or AS**	**Sequence**	**Nucleotide position**	**Starting plasmid**
SP101	OTR 1601	U5 region	AS	5’ GGA GAC GGG ATG GCG AAG CCC GAC AAA CAC ACG AGA CGT GAA TAT T 3’	HYB-MTV 1184-1229	DA024 (Aktar et al., 2014)
	OTR 1602		S	5’ TTC GCC ATC CCG TCT CCG 3’	HYB-MTV 1213-1224	
SP102	OTR 1603	Gag region	AS	5’ GAG TTT CTG CCC TTT TGA CAG ACA GAC CCC CAT TTC CAA TGG CTC 3’	HYB-MTV 1473-1517	SP101
	OTR 1604		S	5’ TCA AAA GGG CAG AAA CTC TTT G 3’	HYB-MTV 1500-1521	
SP103	OTR 1605	U5 region	AS	5’ ACG AGC GGA GAC GGG ATC CAG AAC AGA CAC AAA CAC ACG AGA C 3’	HYB-MTV 1193-1235	DA024
	OTR 1606		S	5’ ATC CCG TCT CCG CTC GTC 3’	HYB-MTV 1219-1236	
SP104	OTR 1663	Gag region	AS	5’ GCC CTT TTG AGC CCG AGC GGC CCA TTT CCA ATG GCT CAC CG 3’	HYB-MTV 1469-1509	SP103
	OTR 1608		S	5’ CTC GGG CTC AAA AGG GCA G 3’	HYB-MTV 1493-1511	
SP105	OTR 1609	U5 region	AS	5’ GAT AAG TGA CGA GCG GAG TAC CCA TGG CGA ACA GAC ACA AAC ACA C 3’	HYB-MTV 1198-1243	DA024
	OTR 1610		S	5’ CTC CGC TCG TCA CTT ATC C 3’	HYB-MTV 1226-1244	
SP106	OTR 1664	Gag region	AS	5’ CCT TTT GAG CCC GAG ACC GGG CAT TCC AAT GGC TCA CCG TAA CCT 3’	HYB-MTV 1463-1507	SP105
	OTR 1612		S	5’ GGT CTC GGG CTC AAA AGG G 3’	HYB-MTV 1490-1508	
SP107	OTR 1668	U5 region	AS	5’ GAA AGT GAA GGA TAA GTG ACC TCG CCT GAC GGG ATG GCG AAC AGA CAC 3’	HYB-MTV 1206-1253	DA024
	OTR 1669		S	5’ GTC ACT TAT CCT TCA CTT TCC 3’	HYB-MTV 1234-1254	

Supplementary Table 2 (continued)

Primers used for introduction of mutations and construction of clones						
**Mutation Names**	**Primers**	**Region where mutations were introduced/ description**	***S or AS**	**Sequence**	**Nucleotide position****	**Starting plasmid**
SP108	OTR 1670	U5 region	AS	5’ TCC CTA TGG TGA GTC CGT AGG CGA GTT GTG ATG ATA GCC AGA CAA GAA A 3’	HYB-MTV 1386-1434	SP107
	OTR 1671		S	5’ ACG GAC TCA CCA TAG GGA 3’	HYB-MTV 1417-1434	
AK29	OTR 1371	Amplifying from R to mSD	AS	5’ GAT TGG TGT TTC GGC ATC CTC TTC TCC GTA GGC GGG 3’	HYB-MTV 1443-1460 & 6535-6552	DA024
	OTR 1370	Amplifying from *env* splice acceptor to 424 bp of *env*	S	5’ GAT GCC GAA ACA CCA ATC TG 3’	HYB-MTV 6535-6554	
	OTR 1372		AS	5’ aaa **ccc ggg** TAA ACC CGT GAA AGT CAG GC 3’	HYB-MTV 6940-6959	
OTR 249		Outer primers used for construction of subgenomic transfer vectors	S	5’ CC GCT AGC CTT CGC GAT GTA CGG GCC AGA 3’	pCDNA3 204-224	First round amplification products in the SOE PCR
OTR 552			AS	5’ cg **act agt gat atc** GTT CCC CTG GTC CCA T 3’	HYB-MTV 1867-1885	
OTR 984		Primers used for construction of *in vitro* transcribing clones	S	5’ ccc **aag ctt** AAT ACG ACT CAC TAT AGG GCA ACA GTC CTA ATA TTC ACG 3’	HYB-MTV 1173-1193	DA024/its mutants
OTR 985			AS	5’ aaa **ccc ggg** TTC CCC TGG TCC CAT AAG 3’	HYB-MTV 1867-1885	
**Primers used for SHAPE, RT-qPCR and other amplifications**						
OTR 671		Vector specific	S	5’ GTC CTA ATA TTC ACG TCT CGT GTG 3’	HYB-MTV 1179-1202	DA024/its mutants
OTR672		Vector specific	AS	5’ CTG TTC GGG CGC CAG CTG CCG CAG 3’	HYB-MTV 1298-1321	DA024/its mutants

Supplementary Table 2 (continued)

Primers used for SHAPE, RT-qPCR and other amplifications				
**Primer**	**Description**	***S or AS**	**Sequence**	**Nucleotide position or reference**
MMTV_WT_VIC	Labelled primers used for SHAPE analysis of WT (SA35) and mutant RNAs	AS	VIC - 5' CTCCTTCTTCGGGAAACCAAG 3'	HYB-MTV 1615-1635
MMTV_WT_NED		AS	NED - 5' CTCCTTCTTCGGGAAACCAAG 3'	HYB-MTV 1615-1635
MMTV_329_VIC		AS	VIC - 5' CAAAGAGTTTCTGCCCTTTTG 3'	HYB-MTV 1501-1521
MMTV_329_NED		AS	NED - 5' CAAAGAGTTTCTGCCCTTTTG 3'	HYB-MTV 1501-1521
OTR 581	β-actin spliced or unspliced mRNA	AS	5' GGC ATG GGG GAG GGC ATA CC 3'	(Tan et al., 1995)
OTR 582	β-actin unspliced mRNA	S	5' CCA GTG GCT TCC CCA GTG 3'	
MMTV_LRI_2	qPCR probe	Probe	FAM- 5' CTT ATC CTT CAC TTT CCA G 3'	HYB-MTV 1238-1256
MMTV_LRI_2F	qPCR Forward primer	S	5' TCA CGT CTC GTG TGT TTG 3'	HYB-MTV 1189-1207
MMTV_LRI_2R	qPCR Reverse Primer	AS	5' CCT GAG GGT CAC CGG GGT C 3'	HYB-MTV 1271-1288

*S, sense; AS, antisense

** Nucleotide positions refer to the sequence of HYBMTV, a molecular clone created earlier (Shackleford and Varmus, 1988).

Sequences in lower case represent dummy sequences that were introduced into the oligos to facilitate efficient cleaving by restriction enzymes.

Sequences in lower case and bold represent restriction enzyme sites that were introduced into the oligos.

Sequences in upper case and underlined (AAT ACG ACT CAC TAT AGGG) represent T7 Promoter sequences.

References

1) Aktar,S.J., Vivet-Boudou,V., Ali,L.M., Jabeen,A., Kalloush,R.M., Richer,D., Mustafa,F., Marquet,R. and Rizvi,T.A. (2014) Structural basis of genomic RNA (gRNA) dimerization and packaging determinants of mouse mammary tumor virus (MMTV). *Retrovirology*, **11**, 96.
2) Shackleford,G.M. and Varmus,H.E. (1988) Construction of a clonable, infectious, and tumorigenic mouse mammary tumor virus provirus and a derivative genetic vector. *Proc. Natl. Acad. Sci.*, **85**, 9655–9659.
3) Tan, Wei, Barbara K. Felber, Andrei S. Zolotukhin, George N. Pavlakis, and Stefan Schwartz. (1995) Efficient expression of the human papillomavirus type 16 L1 protein in epithelial cells by using Rev and the Rev-responsive element of human immunodeficiency virus or the cis-acting transactivation element of simian retrovirus type 1." *Journal of virology*, **69:**5607–5620.
