# Supplementary material for: MMTV RNA packaging requires an extended long-range interaction for productive Gag binding to packaging signals
Source: PLoS Biol. 2024 Oct 3;22(10):e3002827. doi: 10.1371/journal.pbio.3002827 (PMC11449360; doi:10.1371/journal.pbio.3002827)
Supplement: S3 Table — The yellow highlighted nucleotides showed ≥1.5-fold reduction of hSHAPE reactivities in the presence of Pr77Gag. (PDF) [file pbio.3002827.s013.pdf]

Supplementary Table 3. Mean hSHAPE reactivities from three experiments in the absence and presence of $Pr77^{Gag}$. The yellow highlighted nucleotides showed ≥ 1.5 fold reduction of hSHAPE reactivities in the presence of $Pr77^{Gag}$ (*p* -value < 0.05).

hSHAPE Reactivities from 3 independent experiments (SA35-Wild Type)											
Nucleotides		In the Absence of Pr77^{Gag}					In the Presence of Pr77^{Gag}				
Number	Sequence	Expt 1	Expt 2	Expt 3	Mean	SD	Expt 1	Expt 2	Expt 3	Mean	SD
1	G	-999	-999	-999	-999	0.000	-999	-999	-999	-999	0.000
2	C	-999	-999	-999	-999	0.000	-999	-999	-999	-999	0.000
3	A	-999	-999	-999	-999	0.000	-999	-999	-999	-999	0.000
4	A	-999	-999	-999	-999	0.000	-999	-999	-999	-999	0.000
5	C	-999	-999	-999	-999	0.000	-999	-999	-999	-999	0.000
6	A	-999	-999	-999	-999	0.000	-999	-999	-999	-999	0.000
7	G	-999	-999	-999	-999	0.000	-999	-999	-999	-999	0.000
8	U	-999	-999	-999	-999	0.000	-999	-999	-999	-999	0.000
9	C	-999	-999	-999	-999	0.000	-999	-999	-999	-999	0.000
10	C	-999	-999	-999	-999	0.000	-999	-999	-999	-999	0.000
11	U	-999	-999	-999	-999	0.000	-999	-999	-999	-999	0.000
12	A	-999	-999	-999	-999	0.000	-999	-999	-999	-999	0.000
13	A	-999	-999	-999	-999	0.000	-999	-999	-999	-999	0.000
14	U	-999	-999	-999	-999	0.000	-999	-999	-999	-999	0.000
15	A	-999	-999	-999	-999	0.000	-999	-999	-999	-999	0.000
16	U	-999	-999	-999	-999	0.000	-999	-999	-999	-999	0.000
17	U	1.045	0.585	0.840	0.823	0.230	-999	-999	-999	-999	0.000
18	C	0.025	0.010	0.070	0.035	0.031	-999	-999	-999	-999	0.000
19	A	0.135	0.020	0.175	0.110	0.080	0.250	0.065	0.045	0.120	0.113
20	C	0.025	0.000	0.090	0.038	0.046	0.065	0.140	0.065	0.090	0.043
21	G	0.550	0.420	0.560	0.510	0.078	0.430	0.495	0.415	0.447	0.043
22	U	2.515	1.970	2.355	2.280	0.280	1.905	1.935	1.680	1.840	0.139
23	C	0.755	0.460	0.610	0.608	0.148	0.540	0.605	0.530	0.558	0.041
24	U	3.560	2.745	3.045	3.117	0.412	2.575	2.610	2.370	2.518	0.130
25	C	0.865	0.790	0.815	0.823	0.038	0.750	0.770	0.740	0.753	0.015
26	G	0.195	0.120	0.215	0.177	0.050	0.070	0.160	0.220	0.150	0.075
27	U	0.080	0.080	0.060	0.073	0.012	0.150	0.050	0.080	0.093	0.051
28	G	0.000	0.000	0.000	0.000	0.000	0.000	0.000	0.000	0.000	0.000
29	U	0.200	0.095	0.215	0.170	0.065	0.250	0.110	0.245	0.202	0.079
30	G	0.225	0.180	0.270	0.225	0.045	0.250	0.260	0.155	0.222	0.058
31	U	0.125	0.105	0.115	0.115	0.010	0.055	0.000	0.175	0.077	0.089
32	U	0.380	0.380	0.420	0.393	0.023	0.415	0.235	0.380	0.343	0.095
33	U	0.335	0.295	0.325	0.318	0.021	0.230	0.260	0.305	0.265	0.038
34	G	0.315	0.175	0.260	0.250	0.071	0.180	0.165	0.100	0.148	0.043
35	U	1.900	1.310	1.620	1.610	0.295	1.410	1.420	1.310	1.380	0.061
36	G	0.445	0.270	0.315	0.343	0.091	0.295	0.275	0.310	0.293	0.018
37	U	0.090	0.035	0.060	0.062	0.028	0.120	0.095	0.115	0.110	0.013
38	C	0.000	0.000	0.000	0.000	0.000	0.050	0.060	0.070	0.060	0.010
39	U	0.000	0.000	0.130	0.043	0.075	0.000	0.000	0.000	0.000	0.000
40	G	0.000	0.000	0.000	0.000	0.000	0.000	0.000	0.000	0.000	0.000
41	U	0.000	0.000	0.000	0.000	0.000	0.000	0.000	0.000	0.000	0.000
42	U	0.000	0.000	0.000	0.000	0.000	0.255	0.065	0.000	0.107	0.133
43	C	0.510	0.405	0.415	0.443	0.058	0.550	0.740	0.420	0.570	0.161
44	G	0.000	0.000	0.000	0.000	0.000	0.000	0.000	0.000	0.000	0.000
45	C	0.000	0.000	0.000	0.000	0.000	0.000	0.000	0.000	0.000	0.000
46	C	0.000	0.000	0.000	0.000	0.000	0.000	0.000	0.000	0.000	0.000
47	A	0.700	0.620	0.710	0.677	0.049	0.280	0.700	0.435	0.472	0.212
48	U	0.635	0.580	0.765	0.660	0.095	0.190	0.530	0.485	0.402	0.185

hSHAPE Reactivities from 3 independent experiments (SA35-Wild Type)											
Nucleotides		In the Absence of Pr77^{Gag}					In the Presence of Pr77^{Gag}				
Number	Sequence	Expt 1	Expt 2	Expt 3	Mean	SD	Expt 1	Expt 2	Expt 3	Mean	SD
49	C	0.025	0.000	0.070	0.032	0.035	0.000	0.000	0.040	0.013	0.023
50	C	0.000	0.000	0.000	0.000	0.000	0.000	0.000	0.000	0.000	0.000
51	C	0.000	0.000	0.000	0.000	0.000	0.000	0.000	0.000	0.000	0.000
52	G	0.000	0.000	0.000	0.000	0.000	0.000	0.000	0.000	0.000	0.000
53	U	0.405	0.305	0.360	0.357	0.050	0.315	0.225	0.240	0.260	0.048
54	C	0.555	0.470	0.535	0.520	0.044	0.425	0.315	0.310	0.350	0.065
55	U	0.105	0.060	0.040	0.068	0.033	0.000	0.025	0.025	0.017	0.014
56	C	0.000	0.000	0.000	0.000	0.000	0.000	0.000	0.000	0.000	0.000
57	C	0.000	0.000	0.000	0.000	0.000	0.000	0.000	0.000	0.000	0.000
58	G	0.000	0.000	0.000	0.000	0.000	0.000	0.000	0.000	0.000	0.000
59	C	0.000	0.000	0.000	0.000	0.000	0.000	0.000	0.000	0.000	0.000
60	U	0.000	0.000	0.000	0.000	0.000	0.000	0.000	0.000	0.000	0.000
61	C	0.000	0.000	0.000	0.000	0.000	0.000	0.000	0.000	0.000	0.000
62	G	0.000	0.000	0.000	0.000	0.000	0.000	0.000	0.000	0.000	0.000
63	U	0.065	0.000	0.025	0.030	0.033	0.070	0.000	0.010	0.027	0.038
64	C	0.000	0.115	0.000	0.038	0.066	1.055	1.730	1.660	1.482	0.371
65	A	0.345	0.280	0.300	0.308	0.033	0.125	0.340	0.190	0.218	0.110
66	C	0.000	0.000	0.000	0.000	0.000	0.000	0.000	0.000	0.000	0.000
67	U	0.345	0.320	0.270	0.312	0.038	0.325	0.375	0.400	0.367	0.038
68	U	0.415	0.535	0.385	0.445	0.079	3.740	3.340	2.960	3.347	0.390
69	A	0.785	0.890	0.800	0.825	0.057	0.450	0.595	0.615	0.553	0.090
70	U	0.390	0.435	0.380	0.402	0.029	0.165	0.270	0.235	0.223	0.053
71	C	0.000	0.055	0.000	0.018	0.032	0.120	0.110	0.190	0.140	0.044
72	C	0.000	0.000	0.000	0.000	0.000	0.000	0.000	0.000	0.000	0.000
73	U	0.485	0.410	0.395	0.430	0.048	0.275	0.310	0.240	0.275	0.035
74	U	1.150	0.990	1.010	1.050	0.087	1.155	1.180	1.050	1.128	0.069
75	C	0.000	0.000	0.000	0.000	0.000	5.310	4.280	4.590	4.727	0.528
76	A	1.075	0.810	0.840	0.908	0.145	1.020	1.140	1.180	1.113	0.083
77	C	0.000	0.000	0.070	0.023	0.040	0.225	0.120	0.000	0.115	0.113
78	U	1.100	0.875	1.070	1.015	0.122	0.845	0.540	0.575	0.653	0.167
79	U	1.265	1.345	1.175	1.262	0.085	0.920	0.960	0.840	0.907	0.061
80	U	0.820	0.800	0.820	0.813	0.012	0.645	0.775	0.610	0.677	0.087
81	C	0.550	0.630	0.565	0.582	0.043	0.220	0.340	0.175	0.245	0.085
82	C	0.310	0.315	0.220	0.282	0.053	1.370	1.430	1.260	1.353	0.086
83	A	0.250	0.925	0.685	0.620	0.342	1.630	1.560	1.500	1.563	0.065
84	G	0.855	1.440	1.195	1.163	0.294	0.865	0.170	0.930	0.655	0.421
85	A	0.000	0.115	0.145	0.087	0.077	0.055	0.040	0.150	0.082	0.060
86	G	0.020	0.000	0.000	0.007	0.012	0.000	0.000	0.025	0.008	0.014
87	G	0.000	0.000	0.000	0.000	0.000	0.000	0.000	0.000	0.000	0.000
88	G	0.000	0.000	0.000	0.000	0.000	0.000	0.000	0.000	0.000	0.000
89	U	0.000	0.000	0.000	0.000	0.000	0.000	0.000	0.000	0.000	0.000
90	C	0.000	0.000	0.000	0.000	0.000	0.000	0.000	0.000	0.000	0.000
91	C	0.000	0.000	0.000	0.000	0.000	0.000	0.000	0.000	0.000	0.000
92	C	0.000	0.000	0.000	0.000	0.000	0.000	0.000	0.000	0.000	0.000
93	C	0.000	0.000	0.000	0.000	0.000	0.000	0.000	0.000	0.000	0.000
94	C	0.000	0.000	0.000	0.000	0.000	0.000	0.000	0.000	0.000	0.000
95	C	0.000	0.000	0.000	0.000	0.000	0.000	0.000	0.000	0.000	0.000
96	G	0.080	0.035	0.005	0.040	0.038	0.000	0.000	0.000	0.000	0.000

hSHAPE Reactivities from 3 independent experiments (SA35-Wild Type)											
Nucleotides		In the Absence of Pr77^{Gag}					In the Presence of Pr77^{Gag}				
Number	Sequence	Expt 1	Expt 2	Expt 3	Mean	SD	Expt 1	Expt 2	Expt 3	Mean	SD
97	C	0.000	0.000	0.000	0.000	0.000	0.000	0.000	0.000	0.000	0.000
98	A	0.345	0.310	0.300	0.318	0.024	0.170	0.200	0.140	0.170	0.030
99	G	0.705	0.665	0.680	0.683	0.020	0.230	0.285	0.335	0.283	0.053
100	A	1.885	1.890	1.880	1.885	0.005	1.225	1.070	1.020	1.105	0.107
101	C	0.105	0.145	0.170	0.140	0.033	0.185	0.070	0.035	0.097	0.078
102	C	0.000	0.000	0.000	0.000	0.000	0.000	0.010	0.000	0.003	0.006
103	C	0.000	0.000	0.000	0.000	0.000	0.000	0.000	0.000	0.000	0.000
104	C	0.000	0.000	0.000	0.000	0.000	0.000	0.000	0.000	0.000	0.000
105	G	0.000	0.000	0.000	0.000	0.000	0.000	0.000	0.000	0.000	0.000
106	G	0.000	0.000	0.000	0.000	0.000	0.000	0.000	0.000	0.000	0.000
107	U	0.090	0.010	0.000	0.033	0.049	0.010	0.040	0.120	0.057	0.057
108	G	0.035	0.020	0.080	0.045	0.031	0.095	0.015	0.100	0.070	0.048
109	A	0.060	0.040	0.040	0.047	0.012	0.000	0.015	0.000	0.005	0.009
110	C	0.000	0.000	0.000	0.000	0.000	0.000	0.000	0.090	0.030	0.052
111	C	0.000	0.000	0.000	0.000	0.000	0.000	0.000	0.000	0.000	0.000
112	C	0.000	0.000	0.000	0.000	0.000	0.000	0.000	0.000	0.000	0.000
113	U	2.470	1.710	1.505	1.895	0.508	1.445	1.335	1.460	1.413	0.068
114	C	0.640	0.420	0.440	0.500	0.122	0.645	0.580	0.555	0.593	0.046
115	A	3.600	3.630	3.410	3.547	0.119	2.320	2.230	1.750	2.100	0.306
116	G	0.290	0.310	0.400	0.333	0.059	0.140	0.080	0.040	0.087	0.050
117	G	0.000	0.000	0.000	0.000	0.000	0.000	0.000	0.000	0.000	0.000
118	U	0.010	0.015	0.000	0.008	0.008	0.000	0.000	0.030	0.010	0.017
119	C	0.220	0.165	0.190	0.192	0.028	0.220	0.000	0.150	0.123	0.112
120	G	0.200	0.250	0.420	0.290	0.115	0.009	0.122	0.028	0.053	0.061
121	G	0.625	0.545	0.470	0.547	0.078	0.345	0.385	0.450	0.393	0.053
122	C	0.000	0.000	0.000	0.000	0.000	0.000	0.000	0.000	0.000	0.000
123	C	0.000	0.000	0.000	0.000	0.000	0.000	0.000	0.000	0.000	0.000
124	G	0.000	0.000	0.000	0.000	0.000	0.000	0.000	0.000	0.000	0.000
125	A	0.405	0.380	0.260	0.348	0.078	0.230	0.120	0.065	0.138	0.084
126	C	0.000	0.000	0.000	0.000	0.000	0.000	0.000	0.025	0.008	0.014
127	U	0.190	0.130	0.100	0.140	0.046	0.025	0.050	0.000	0.025	0.025
128	G	0.325	0.245	0.160	0.243	0.083	0.020	0.010	0.015	0.015	0.005
129	C	0.000	0.000	0.000	0.000	0.000	0.000	0.000	0.000	0.000	0.000
130	G	0.000	0.000	0.000	0.000	0.000	0.000	0.000	0.000	0.000	0.000
131	G	0.000	0.000	0.000	0.000	0.000	0.000	0.000	0.000	0.000	0.000
132	C	0.000	0.000	0.000	0.000	0.000	0.000	0.000	0.000	0.000	0.000
133	A	0.000	0.000	0.000	0.000	0.000	0.000	0.000	0.000	0.000	0.000
134	G	0.255	0.260	0.190	0.235	0.039	0.000	0.000	0.000	0.000	0.000
135	C	0.150	0.110	0.130	0.130	0.020	0.000	0.000	0.000	0.000	0.000
136	U	0.325	0.500	0.340	0.388	0.097	0.185	0.265	0.155	0.202	0.057
137	G	0.300	0.475	0.460	0.412	0.097	0.000	0.000	0.000	0.000	0.000
138	G	0.000	0.000	0.000	0.000	0.000	0.000	0.000	0.000	0.000	0.000
139	C	0.000	0.000	0.000	0.000	0.000	0.000	0.000	0.000	0.000	0.000
140	G	0.120	0.070	0.050	0.080	0.036	0.040	0.030	0.015	0.028	0.013
141	C	0.000	0.000	0.000	0.000	0.000	0.000	0.000	0.000	0.000	0.000
142	C	0.000	0.000	0.000	0.000	0.000	0.000	0.000	0.000	0.000	0.000
143	C	0.000	0.000	0.000	0.000	0.000	0.000	0.000	0.000	0.000	0.000
144	G	0.520	0.450	0.330	0.433	0.096	0.000	0.070	0.080	0.050	0.044

hSHAPE Reactivities from 3 independent experiments (SA35-Wild Type)											
Nucleotides		In the Absence of Pr77^{Gag}					In the Presence of Pr77^{Gag}				
Number	Sequence	Expt 1	Expt 2	Expt 3	Mean	SD	Expt 1	Expt 2	Expt 3	Mean	SD
145	A	1.290	0.925	1.000	1.072	0.193	0.596	0.496	0.549	0.547	0.050
146	A	1.310	1.190	1.250	1.250	0.060	0.800	0.930	0.870	0.867	0.065
147	C	0.000	0.130	0.090	0.073	0.067	1.460	1.200	1.030	1.230	0.217
148	A	0.680	0.700	0.730	0.703	0.025	0.435	0.320	0.470	0.408	0.078
149	G	0.000	0.000	0.000	0.000	0.000	0.000	0.000	0.000	0.000	0.000
150	G	0.000	0.000	0.000	0.000	0.000	0.000	0.000	0.000	0.000	0.000
151	G	0.000	0.000	0.000	0.000	0.000	0.000	0.000	0.000	0.000	0.000
152	A	0.000	0.000	0.000	0.000	0.000	0.000	0.000	0.000	0.000	0.000
153	C	0.000	0.000	0.000	0.000	0.000	0.000	0.000	0.000	0.000	0.000
154	C	0.000	0.000	0.000	0.000	0.000	0.000	0.000	0.000	0.000	0.000
155	C	0.000	0.000	0.000	0.000	0.000	0.000	0.000	0.000	0.000	0.000
156	U	0.000	0.000	0.000	0.000	0.000	0.000	0.000	0.000	0.000	0.000
157	C	0.290	0.150	0.250	0.230	0.072	0.000	0.000	0.000	0.000	0.000
158	G	0.000	0.000	0.000	0.000	0.000	0.000	0.000	0.000	0.000	0.000
159	G	0.000	0.000	0.000	0.000	0.000	0.000	0.000	0.000	0.000	0.000
160	A	0.000	0.000	0.000	0.000	0.000	0.000	0.000	0.000	0.000	0.000
161	U	0.260	0.290	0.210	0.253	0.040	0.130	0.245	0.165	0.180	0.059
162	A	0.000	0.000	0.000	0.000	0.000	0.000	0.000	0.000	0.000	0.000
163	A	0.035	0.000	0.000	0.012	0.020	0.000	0.000	0.000	0.000	0.000
164	G	0.000	0.000	0.000	0.000	0.000	0.000	0.000	0.000	0.000	0.000
165	U	0.000	0.000	0.000	0.000	0.000	0.000	0.000	0.000	0.000	0.000
166	G	0.000	0.000	0.000	0.000	0.000	0.000	0.000	0.000	0.000	0.000
167	A	0.000	0.000	0.000	0.000	0.000	0.000	0.000	0.000	0.000	0.000
168	C	0.000	0.000	0.000	0.000	0.000	0.000	0.000	0.000	0.000	0.000
169	C	0.000	0.000	0.000	0.000	0.000	0.000	0.000	0.000	0.000	0.000
170	C	0.000	0.000	0.000	0.000	0.000	0.000	0.000	0.000	0.000	0.000
171	U	0.000	0.000	0.000	0.000	0.000	0.000	0.000	0.000	0.000	0.000
172	U	0.430	0.400	0.340	0.390	0.046	0.510	0.535	0.420	0.488	0.060
173	G	0.530	0.480	0.685	0.565	0.107	0.135	0.275	0.250	0.220	0.075
174	U	0.330	0.320	0.230	0.293	0.055	0.210	0.265	0.165	0.213	0.050
175	C	0.000	0.000	0.000	0.000	0.000	0.000	0.000	0.000	0.000	0.000
176	U	0.000	0.000	0.000	0.000	0.000	0.000	0.000	0.000	0.000	0.000
177	C	0.000	0.000	0.000	0.000	0.000	0.000	0.000	0.000	0.000	0.000
178	U	0.000	0.000	0.000	0.000	0.000	0.000	0.000	0.000	0.000	0.000
179	A	0.150	0.060	0.000	0.070	0.075	0.250	0.475	0.130	0.285	0.175
180	U	0.000	0.000	0.000	0.000	0.000	0.000	0.105	0.000	0.035	0.061
181	U	0.120	0.055	0.000	0.058	0.060	0.075	0.110	0.015	0.067	0.048
182	U	0.095	0.100	0.035	0.077	0.036	0.100	0.050	0.060	0.070	0.026
183	C	0.000	0.000	0.000	0.000	0.000	0.015	0.000	0.005	0.007	0.008
184	U	0.000	0.075	0.000	0.025	0.043	1.410	0.685	1.405	1.167	0.417
185	A	0.150	0.100	0.060	0.103	0.045	0.125	0.330	0.130	0.195	0.117
186	C	0.000	0.000	0.000	0.000	0.000	0.000	0.000	0.000	0.000	0.000
187	U	0.100	0.380	0.210	0.230	0.141	2.245	1.680	2.180	2.035	0.309
188	A	0.795	0.855	0.810	0.820	0.031	0.675	0.695	0.620	0.663	0.039
189	U	0.155	0.190	0.165	0.170	0.018	0.130	0.140	0.125	0.132	0.008
190	U	0.135	0.160	0.110	0.135	0.025	0.180	0.170	0.140	0.163	0.021
191	U	0.230	0.265	0.195	0.230	0.035	0.155	0.125	0.155	0.145	0.017
192	G	0.085	0.080	0.095	0.087	0.008	0.000	0.025	0.000	0.008	0.014

hSHAPE Reactivities from 3 independent experiments (SA35-Wild Type)											
Nucleotides		In the Absence of Pr77^{Gag}					In the Presence of Pr77^{Gag}				
Number	Sequence	Expt 1	Expt 2	Expt 3	Mean	SD	Expt 1	Expt 2	Expt 3	Mean	SD
193	G	0.000	0.000	0.000	0.000	0.000	0.000	0.025	0.000	0.008	0.014
194	U	0.495	0.240	0.280	0.338	0.137	0.150	0.430	0.155	0.245	0.160
195	G	0.830	0.730	0.665	0.742	0.083	0.170	0.425	0.200	0.265	0.139
196	U	0.815	0.575	0.615	0.668	0.129	0.180	0.330	0.270	0.260	0.075
197	U	0.805	0.680	0.770	0.752	0.064	0.340	0.365	0.190	0.298	0.095
198	U	0.555	0.310	0.390	0.418	0.125	0.130	0.390	0.175	0.232	0.139
199	G	0.010	0.000	0.000	0.003	0.006	0.000	0.020	0.000	0.007	0.012
200	U	0.030	0.030	0.010	0.023	0.012	0.000	0.035	0.000	0.012	0.020
201	C	0.000	0.000	0.000	0.000	0.000	0.000	0.000	0.000	0.000	0.000
202	U	0.000	0.000	0.000	0.000	0.000	0.000	0.000	0.000	0.000	0.000
203	U	0.145	0.070	0.110	0.108	0.038	0.175	0.190	0.095	0.153	0.051
204	G	0.050	0.000	0.000	0.017	0.029	0.020	0.080	0.000	0.033	0.042
205	U	0.045	0.145	0.060	0.083	0.054	1.510	1.140	1.365	1.338	0.186
206	A	0.085	0.020	0.010	0.038	0.041	0.160	0.190	0.085	0.145	0.054
207	U	0.155	0.100	0.065	0.107	0.045	0.090	0.165	0.025	0.093	0.070
208	U	0.260	0.220	0.190	0.223	0.035	0.220	0.255	0.140	0.205	0.059
209	G	0.490	0.460	0.335	0.428	0.082	0.190	0.350	0.230	0.257	0.083
210	U	0.110	0.100	0.100	0.103	0.006	0.085	0.110	0.060	0.085	0.025
211	C	0.000	0.000	0.000	0.000	0.000	0.000	0.000	0.000	0.000	0.000
212	U	0.000	0.000	0.000	0.000	0.000	0.000	0.000	0.000	0.000	0.000
213	C	0.000	0.000	0.000	0.000	0.000	0.000	0.000	0.000	0.000	0.000
214	U	0.000	0.000	0.000	0.000	0.000	0.000	0.000	0.000	0.000	0.000
215	U	0.000	0.000	0.000	0.000	0.000	0.000	0.000	0.000	0.000	0.000
216	U	0.000	0.000	0.000	0.000	0.000	0.000	0.000	0.000	0.000	0.000
217	C	0.000	0.000	0.000	0.000	0.000	0.000	0.000	0.000	0.000	0.000
218	U	0.095	0.050	0.000	0.048	0.048	0.000	0.000	0.000	0.000	0.000
219	U	0.405	0.410	0.310	0.375	0.056	0.445	0.320	0.375	0.380	0.063
220	G	0.650	0.675	0.530	0.618	0.078	0.255	0.260	0.305	0.273	0.028
221	U	0.180	0.165	0.120	0.155	0.031	0.125	0.100	0.115	0.113	0.013
222	C	0.105	0.050	0.055	0.070	0.030	0.020	0.050	0.130	0.067	0.057
223	U	0.290	0.265	0.245	0.267	0.023	0.220	0.210	0.180	0.203	0.021
224	G	0.110	0.130	0.015	0.085	0.061	0.020	0.030	0.030	0.027	0.006
225	G	0.000	0.000	0.000	0.000	0.000	0.000	0.000	0.000	0.000	0.000
226	C	0.000	0.000	0.000	0.000	0.000	0.000	0.000	0.000	0.000	0.000
227	U	0.270	0.500	0.220	0.330	0.149	1.495	0.860	1.395	1.250	0.341
228	A	0.595	0.560	0.515	0.557	0.040	0.425	0.380	0.420	0.408	0.025
229	U	0.355	0.365	0.305	0.342	0.032	0.450	0.370	0.330	0.383	0.061
230	C	0.000	0.000	0.000	0.000	0.000	3.050	2.670	1.920	2.547	0.575
231	A	0.755	0.740	0.615	0.703	0.077	0.600	0.630	0.525	0.585	0.054
232	U	0.470	0.480	0.275	0.408	0.116	0.505	0.690	0.490	0.562	0.111
233	C	0.090	0.000	0.000	0.030	0.052	3.940	3.140	2.160	3.080	0.892
234	A	0.655	0.665	0.525	0.615	0.078	0.665	0.920	0.710	0.765	0.136
235	C	0.000	0.000	0.000	0.000	0.000	4.790	4.190	4.700	4.560	0.324
236	A	0.825	0.730	0.665	0.740	0.080	0.815	0.435	0.900	0.717	0.248
237	A	1.280	1.230	1.090	1.200	0.098	0.920	0.920	0.895	0.912	0.014
238	G	0.795	0.765	0.785	0.782	0.015	0.425	0.400	0.510	0.445	0.058
239	A	0.410	0.370	0.430	0.403	0.031	0.195	0.125	0.210	0.177	0.045
240	G	0.150	0.140	0.110	0.133	0.021	0.050	0.070	0.060	0.060	0.010

hSHAPE Reactivities from 3 independent experiments (SA35-Wild Type)											
Nucleotides		In the Absence of Pr77^{Gag}					In the Presence of Pr77^{Gag}				
Number	Sequence	Expt 1	Expt 2	Expt 3	Mean	SD	Expt 1	Expt 2	Expt 3	Mean	SD
241	C	0.050	0.075	0.050	0.058	0.014	0.080	0.090	0.095	0.088	0.008
242	G	0.060	0.000	0.000	0.020	0.035	0.000	0.040	0.000	0.013	0.023
243	G	0.000	0.000	0.000	0.000	0.000	0.000	0.000	0.000	0.000	0.000
244	A	0.345	0.325	0.245	0.305	0.053	0.130	0.160	0.115	0.135	0.023
245	A	0.500	0.430	0.395	0.442	0.053	0.275	0.310	0.270	0.285	0.022
246	C	0.000	0.000	0.000	0.000	0.000	0.000	0.000	0.000	0.000	0.000
247	G	0.000	0.000	0.000	0.000	0.000	0.000	0.000	0.000	0.000	0.000
248	G	0.000	0.000	0.000	0.000	0.000	0.000	0.000	0.000	0.000	0.000
249	A	2.620	2.780	2.590	2.663	0.102	1.850	1.995	1.885	1.910	0.076
250	C	0.000	0.000	0.000	0.000	0.000	0.000	0.000	0.000	0.000	0.000
251	U	0.000	0.000	0.020	0.007	0.012	0.040	0.080	0.015	0.045	0.033
252	C	0.110	0.130	0.080	0.107	0.025	0.190	0.260	0.175	0.208	0.045
253	A	0.295	0.320	0.255	0.290	0.033	0.100	0.150	0.125	0.125	0.025
254	C	0.000	0.000	0.000	0.000	0.000	0.000	0.000	0.000	0.000	0.000
255	C	0.000	0.000	0.000	0.000	0.000	0.350	0.310	0.330	0.330	0.020
256	A	0.330	0.285	0.305	0.307	0.023	0.235	0.165	0.190	0.197	0.035
257	U	0.700	0.810	0.645	0.718	0.084	1.535	1.235	1.345	1.372	0.152
258	A	1.020	1.080	1.080	1.060	0.035	0.895	0.785	0.815	0.832	0.057
259	G	0.120	0.080	0.155	0.118	0.038	0.190	0.110	0.045	0.115	0.073
260	G	0.020	0.000	0.150	0.057	0.081	0.000	0.000	0.000	0.000	0.000
261	G	0.000	0.000	0.000	0.000	0.000	0.000	0.000	0.000	0.000	0.000
262	A	0.590	0.545	0.570	0.568	0.023	0.300	0.335	0.310	0.315	0.018
263	G	0.755	0.680	0.545	0.660	0.106	0.260	0.325	0.305	0.297	0.033
264	C	0.560	0.660	0.585	0.602	0.052	0.195	0.215	0.310	0.240	0.061
265	U	2.080	1.925	1.530	1.845	0.284	1.450	1.160	1.135	1.248	0.175
266	G	1.850	1.670	1.120	1.547	0.380	0.841	0.641	0.608	0.697	0.126
267	C	0.000	0.000	0.000	0.000	0.000	0.550	1.090	1.130	0.923	0.324
268	A	1.725	1.770	1.160	1.552	0.340	0.856	0.824	0.825	0.835	0.018
269	G	0.000	0.000	0.000	0.000	0.000	0.000	0.000	0.000	0.000	0.000
270	U	0.000	0.095	0.000	0.032	0.055	0.000	0.000	0.045	0.015	0.026
271	C	0.000	0.000	0.000	0.000	0.000	0.000	0.000	0.000	0.000	0.000
272	C	0.000	0.000	0.000	0.000	0.000	0.000	0.000	0.000	0.000	0.000
273	C	0.000	0.000	0.000	0.000	0.000	0.000	0.000	0.000	0.000	0.000
274	G	0.000	0.000	0.000	0.000	0.000	0.000	0.000	0.000	0.000	0.000
275	C	0.000	0.000	0.000	0.000	0.000	0.000	0.000	0.000	0.000	0.000
276	C	0.030	0.000	0.020	0.017	0.015	0.130	0.035	0.160	0.108	0.065
277	U	0.080	0.055	0.065	0.067	0.013	0.105	0.075	0.090	0.090	0.015
278	A	0.050	0.055	0.070	0.058	0.010	0.035	0.020	0.035	0.030	0.009
279	C	0.200	0.130	0.030	0.120	0.085	0.115	0.090	0.085	0.097	0.016
280	G	0.505	0.510	0.420	0.478	0.051	0.270	0.235	0.260	0.255	0.018
281	G	2.055	2.190	2.140	2.128	0.068	1.200	1.280	1.300	1.260	0.053
282	A	3.310	3.610	3.470	3.463	0.150	2.410	2.140	2.275	2.275	0.135
283	G	1.630	1.900	1.810	1.780	0.137	1.135	1.020	1.000	1.052	0.073
284	A	3.310	3.790	3.520	3.540	0.241	2.080	2.167	1.903	2.050	0.135
285	A	3.240	3.540	3.440	3.407	0.153	2.000	2.125	1.985	2.037	0.077
286	G	2.350	2.055	2.360	2.255	0.173	1.180	1.300	1.095	1.192	0.103
287	A	3.820	4.285	3.630	3.912	0.337	2.550	2.240	1.650	2.147	0.457
288	G	1.455	1.370	1.820	1.548	0.239	0.875	0.730	0.810	0.805	0.073

hSHAPE Reactivities from 3 independent experiments (SA35-Wild Type)											
Nucleotides		In the Absence of Pr77^{Gag}					In the Presence of Pr77^{Gag}				
Number	Sequence	Expt 1	Expt 2	Expt 3	Mean	SD	Expt 1	Expt 2	Expt 3	Mean	SD
289	G	0.000	0.000	0.000	0.000	0.000	0.000	0.000	0.000	0.000	0.000
290	U	0.000	0.000	0.000	0.000	0.000	0.005	0.000	0.000	0.002	0.003
291	A	0.040	0.000	0.000	0.013	0.023	0.000	0.000	0.025	0.008	0.014
292	G	0.000	0.000	0.000	0.000	0.000	0.000	0.000	0.000	0.000	0.000
293	G	0.000	0.000	0.000	0.000	0.000	0.000	0.000	0.000	0.000	0.000
294	U	0.320	0.080	0.000	0.133	0.167	0.110	0.090	0.070	0.090	0.020
295	U	0.860	0.520	0.390	0.590	0.243	0.660	0.450	0.610	0.573	0.110
296	A	1.580	1.260	1.270	1.370	0.182	1.120	1.280	1.065	1.155	0.112
297	C	1.140	0.800	0.720	0.887	0.223	1.010	0.770	0.930	0.903	0.122
298	G	0.040	0.020	0.000	0.020	0.020	0.010	0.020	0.000	0.010	0.010
299	G	0.000	0.000	0.000	0.000	0.000	0.000	0.000	0.000	0.000	0.000
300	U	0.000	0.000	0.000	0.000	0.000	0.000	0.000	0.000	0.000	0.000
301	G	0.000	0.000	0.000	0.000	0.000	0.000	0.000	0.000	0.000	0.000
302	A	0.000	0.000	0.000	0.000	0.000	0.000	0.000	0.000	0.000	0.000
303	G	0.000	0.000	0.000	0.000	0.000	0.000	0.000	0.000	0.000	0.000
304	C	0.000	0.000	0.000	0.000	0.000	0.000	0.000	0.000	0.000	0.000
305	C	0.000	0.000	0.000	0.000	0.000	0.420	0.430	1.640	0.830	0.701
306	A	0.780	0.430	1.170	0.793	0.370	0.470	0.500	0.430	0.467	0.035
307	U	1.270	0.890	1.090	1.083	0.190	1.020	1.150	1.030	1.067	0.072
308	U	1.710	1.510	1.710	1.643	0.115	1.510	1.135	1.550	1.398	0.229
309	G	1.530	1.320	1.560	1.470	0.131	1.000	0.800	1.050	0.950	0.132
310	G	1.520	1.260	1.570	1.450	0.166	0.930	1.080	0.900	0.970	0.096
311	A	1.280	0.970	0.730	0.993	0.276	0.940	0.990	0.980	0.970	0.026
312	A	1.010	0.780	0.680	0.823	0.169	0.730	0.710	0.710	0.717	0.012
313	A	0.750	0.570	0.580	0.633	0.101	0.580	0.500	0.550	0.543	0.040
314	U	0.690	0.530	0.640	0.620	0.082	0.700	0.550	0.600	0.617	0.076
315	G	0.000	0.010	0.000	0.003	0.006	0.080	0.010	0.030	0.040	0.036
316	G	0.000	0.000	0.000	0.000	0.000	0.000	0.000	0.000	0.000	0.000
317	G	0.000	0.000	0.000	0.000	0.000	0.000	0.000	0.000	0.000	0.000
318	G	0.000	0.000	0.000	0.000	0.000	0.000	0.000	0.000	0.000	0.000
319	G	0.000	0.000	0.000	0.000	0.000	0.000	0.000	0.000	0.000	0.000
320	U	0.000	0.000	0.000	0.000	0.000	0.000	0.000	0.000	0.000	0.000
321	C	0.000	0.000	0.000	0.000	0.000	0.000	0.000	0.000	0.000	0.000
322	U	0.240	0.210	0.160	0.203	0.040	0.190	0.090	0.190	0.157	0.058
323	C	0.060	0.080	0.020	0.053	0.031	0.000	0.000	0.020	0.007	0.012
324	G	0.280	0.330	0.140	0.250	0.098	0.170	0.030	0.220	0.140	0.098
325	G	0.000	0.000	0.000	0.000	0.000	0.000	0.000	0.000	0.000	0.000
326	G	0.000	0.000	0.000	0.000	0.000	0.000	0.000	0.000	0.000	0.000
327	C	0.000	0.000	0.000	0.000	0.000	0.000	0.000	0.000	0.000	0.000
328	U	0.000	0.010	0.000	0.003	0.006	0.150	0.000	0.050	0.067	0.076
329	C	0.000	0.650	0.000	0.217	0.375	0.000	0.000	0.760	0.253	0.439
330	A	0.740	0.770	0.580	0.697	0.102	0.730	0.720	0.830	0.760	0.061
331	A	1.610	1.360	1.410	1.460	0.132	1.340	1.030	1.320	1.230	0.173
332	A	1.550	1.350	1.340	1.413	0.118	1.170	1.160	0.920	1.083	0.142
333	A	1.280	1.290	1.180	1.250	0.061	0.940	0.970	0.780	0.897	0.102
334	G	0.290	0.230	0.270	0.263	0.031	0.110	0.130	0.140	0.127	0.015
335	G	0.000	0.000	0.000	0.000	0.000	0.000	0.000	0.000	0.000	0.000
336	G	0.000	0.000	0.000	0.000	0.000	0.000	0.000	0.000	0.000	0.000

hSHAPE Reactivities from 3 independent experiments (SA35-Wild Type)											
Nucleotides		In the Absence of Pr77^{Gag}					In the Presence of Pr77^{Gag}				
Number	Sequence	Expt 1	Expt 2	Expt 3	Mean	SD	Expt 1	Expt 2	Expt 3	Mean	SD
337	C	0.060	0.070	0.000	0.043	0.038	0.030	0.150	0.100	0.093	0.060
338	A	0.080	0.030	0.000	0.037	0.040	0.000	0.000	0.000	0.000	0.000
339	G	0.000	0.000	0.000	0.000	0.000	0.000	0.000	0.000	0.000	0.000
340	A	0.110	0.090	0.060	0.087	0.025	0.060	0.030	0.050	0.047	0.015
341	A	0.210	0.170	0.160	0.180	0.026	0.170	0.070	0.090	0.110	0.053
342	A	0.110	0.120	0.100	0.110	0.010	0.070	0.070	0.080	0.073	0.006
343	C	0.000	0.010	0.000	0.003	0.006	0.030	0.050	0.010	0.030	0.020
344	U	0.980	0.880	0.860	0.907	0.064	1.390	1.370	1.330	1.363	0.031
345	C	0.680	0.550	0.530	0.587	0.081	0.840	0.710	0.910	0.820	0.101
346	U	0.750	0.670	0.630	0.683	0.061	0.680	0.680	0.720	0.693	0.023
347	U	1.300	1.140	1.140	1.193	0.092	1.360	1.250	1.370	1.327	0.067
348	U	1.110	0.960	0.980	1.017	0.081	1.350	1.260	1.390	1.333	0.067
349	G	0.010	0.000	0.000	0.003	0.006	0.000	0.000	0.030	0.010	0.017
350	U	0.380	0.310	0.280	0.323	0.051	0.360	0.170	0.440	0.323	0.139
351	U	0.390	0.320	0.300	0.337	0.047	0.390	0.440	0.480	0.437	0.045
352	U	0.000	0.000	0.000	0.000	0.000	0.010	0.000	0.000	0.003	0.006
353	C	0.000	0.000	0.000	0.000	0.000	0.000	0.000	0.000	0.000	0.000
354	U	0.520	0.160	0.220	0.300	0.193	0.620	0.640	0.590	0.617	0.025
355	G	2.180	2.070	1.910	2.053	0.136	2.500	2.380	2.220	2.367	0.140
356	U	0.190	0.140	0.090	0.140	0.050	0.390	0.140	0.290	0.273	0.126
357	U	0.400	0.350	0.390	0.380	0.026	0.520	0.460	0.490	0.490	0.030
358	U	0.480	0.420	0.470	0.457	0.032	0.480	0.370	0.470	0.440	0.061
359	U	0.470	0.430	0.460	0.453	0.021	0.450	0.070	0.610	0.377	0.277
360	A	0.820	0.710	0.760	0.763	0.055	0.850	0.710	0.820	0.793	0.074
361	C	0.130	0.460	0.250	0.280	0.167	0.000	0.000	0.000	0.000	0.000
362	A	0.470	0.400	0.400	0.423	0.040	0.360	0.120	0.390	0.290	0.148
363	A	0.220	0.180	0.170	0.190	0.026	0.170	0.110	0.160	0.147	0.032
364	A	0.070	0.060	0.050	0.060	0.010	0.010	0.040	0.050	0.033	0.021
365	G	0.000	0.000	0.000	0.000	0.000	0.000	0.000	0.000	0.000	0.000
366	G	0.000	0.000	0.000	0.000	0.000	0.000	0.000	0.000	0.000	0.000
367	C	0.000	0.000	0.000	0.000	0.000	0.000	0.000	0.000	0.000	0.000
368	U	0.000	0.000	0.000	0.000	0.000	0.000	0.000	0.000	0.000	0.000
369	C	0.000	0.000	0.000	0.000	0.000	0.000	0.000	0.000	0.000	0.000
370	C	0.000	0.000	0.000	0.000	0.000	0.000	0.000	0.000	0.000	0.000
371	U	0.000	0.010	0.000	0.003	0.006	0.000	0.000	0.000	0.000	0.000
372	C	0.050	0.060	0.050	0.053	0.006	0.040	0.030	0.080	0.050	0.026
373	U	0.380	0.460	0.420	0.420	0.040	0.540	0.170	0.540	0.417	0.214
374	C	1.810	1.770	1.600	1.727	0.112	2.450	1.970	2.460	2.293	0.280
375	A	3.620	2.980	3.410	3.337	0.326	3.980	4.140	4.150	4.090	0.095
376	G	2.310	2.230	1.920	2.153	0.206	2.950	2.490	2.470	2.637	0.272
377	A	2.640	2.150	2.440	2.410	0.246	3.120	2.970	2.780	2.957	0.170
378	G	0.000	0.000	0.000	0.000	0.000	0.000	0.020	0.020	0.013	0.012
379	A	0.000	0.000	0.000	0.000	0.000	0.000	0.000	0.000	0.000	0.000
380	G	0.000	0.000	0.000	0.000	0.000	0.000	0.000	0.440	0.147	0.254
381	G	0.000	0.000	0.000	0.000	0.000	0.000	0.000	0.000	0.000	0.000
382	G	0.000	0.000	0.000	0.000	0.000	0.000	0.000	0.000	0.000	0.000
383	G	0.000	0.000	0.000	0.000	0.000	0.000	0.000	0.000	0.000	0.000
384	U	0.000	0.000	0.040	0.013	0.023	0.000	0.020	0.000	0.007	0.012

hSHAPE Reactivities from 3 independent experiments (SA35-Wild Type)											
Nucleotides		In the Absence of Pr77^{Gag}					In the Presence of Pr77^{Gag}				
Number	Sequence	Expt 1	Expt 2	Expt 3	Mean	SD	Expt 1	Expt 2	Expt 3	Mean	SD
385	C	0.170	0.000	0.000	0.057	0.098	0.000	0.000	0.000	0.000	0.000
386	U	0.000	0.000	0.090	0.030	0.052	0.080	0.000	0.000	0.027	0.046
387	U	1.110	0.570	0.600	0.760	0.303	0.890	0.700	1.020	0.870	0.161
388	C	0.480	1.040	1.270	0.930	0.406	1.480	1.150	1.730	1.453	0.291
389	A	1.390	0.930	1.160	1.160	0.230	1.040	1.130	0.970	1.047	0.080
390	U	0.210	0.120	0.100	0.143	0.059	0.200	0.100	0.130	0.143	0.051
391	G	0.040	0.060	0.080	0.060	0.020	0.050	0.030	0.000	0.027	0.025
392	U	0.070	0.120	0.140	0.110	0.036	0.170	0.100	0.150	0.140	0.036
393	G	0.080	0.070	0.200	0.117	0.072	0.030	0.360	0.070	0.153	0.180
394	A	0.160	0.140	0.360	0.220	0.122	0.190	0.070	0.170	0.143	0.064
395	A	0.300	0.250	0.660	0.403	0.224	0.390	0.200	0.420	0.337	0.119
396	A	0.700	0.580	0.680	0.653	0.064	0.830	0.540	0.780	0.717	0.155
397	G	0.730	0.620	0.670	0.673	0.055	0.810	0.480	0.810	0.700	0.191
398	A	0.190	0.160	0.230	0.193	0.035	0.120	0.050	0.230	0.133	0.091
399	G	0.130	0.330	0.620	0.360	0.246	0.200	0.300	0.160	0.220	0.072
400	A	0.200	0.000	0.050	0.083	0.104	0.000	0.000	0.000	0.000	0.000
401	G	0.410	0.710	0.490	0.537	0.155	1.540	1.100	0.990	1.210	0.291
402	U	0.330	0.420	0.190	0.313	0.116	0.430	0.120	0.280	0.277	0.155
403	A	0.100	0.130	0.080	0.103	0.025	0.180	0.140	0.120	0.147	0.031
404	G	0.090	0.190	0.280	0.187	0.095	0.180	0.000	0.050	0.077	0.093
405	U	0.390	0.610	0.680	0.560	0.151	0.130	0.070	0.120	0.107	0.032
406	G	0.000	0.000	0.000	0.000	0.000	0.480	0.590	0.460	0.510	0.070
407	C	0.310	0.400	0.380	0.363	0.047	0.000	0.710	0.370	0.360	0.355
408	A	0.500	0.460	0.500	0.487	0.023	0.440	0.320	0.510	0.423	0.096
409	A	0.410	0.470	0.460	0.447	0.032	0.660	0.610	0.730	0.667	0.060
410	U	0.460	0.340	0.470	0.423	0.072	0.850	0.450	0.890	0.730	0.243
411	A	0.450	0.370	0.450	0.423	0.046	0.470	0.700	0.760	0.643	0.153
412	G	0.460	0.390	0.540	0.463	0.075	0.480	0.710	0.460	0.550	0.139
413	A	0.480	0.430	0.610	0.507	0.093	0.640	0.680	0.720	0.680	0.040
414	A	0.460	0.430	0.560	0.483	0.068	0.670	0.490	0.830	0.663	0.170
415	U	0.340	0.330	0.450	0.373	0.067	0.770	0.920	0.880	0.857	0.078
416	U	0.660	0.540	0.720	0.640	0.092	0.470	0.350	0.550	0.457	0.101
417	U	0.680	0.690	0.640	0.670	0.026	1.120	1.220	1.160	1.167	0.050
418	U	0.810	0.770	0.860	0.813	0.045	1.170	0.460	1.040	0.890	0.378
419	A	0.410	0.330	0.310	0.350	0.053	1.410	0.670	1.260	1.113	0.391
420	U	0.410	0.990	0.650	0.683	0.291	0.550	0.910	0.510	0.657	0.220
421	C	0.850	0.890	0.790	0.843	0.050	0.850	0.990	1.080	0.973	0.116
422	A	0.690	0.430	0.360	0.493	0.174	1.380	1.160	1.460	1.333	0.155
423	G	0.550	0.210	0.430	0.397	0.172	1.080	0.430	1.020	0.843	0.359
424	U	0.350	0.340	0.320	0.337	0.015	0.240	0.070	0.350	0.220	0.141
425	U	0.140	0.210	0.300	0.217	0.080	0.390	0.110	0.610	0.370	0.251
426	U	0.350	0.420	0.280	0.350	0.070	0.050	0.090	0.020	0.053	0.035
427	C	-999	-999	-999	-999	0.000	-999	-999	-999	-999	0.000
428	U	-999	-999	-999	-999	0.000	-999	-999	-999	-999	0.000
429	A	-999	-999	-999	-999	0.000	-999	-999	-999	-999	0.000
430	A	-999	-999	-999	-999	0.000	-999	-999	-999	-999	0.000
431	U	-999	-999	-999	-999	0.000	-999	-999	-999	-999	0.000
432	A	-999	-999	-999	-999	0.000	-999	-999	-999	-999	0.000

hSHAPE Reactivities from 3 independent experiments (SP101*i*)											
Nucleotides		In the Absence of Pr77[Gag]					In the Presence of Pr77[Gag]				
Number	Sequence	Expt 1	Expt 2	Expt 3	Mean	SD	Expt 1	Expt 2	Expt 3	Mean	SD
1	G	-999	-999	-999	-999	0.000	-999	-999	-999	-999	0.000
2	C	-999	-999	-999	-999	0.000	-999	-999	-999	-999	0.000
3	A	-999	-999	-999	-999	0.000	-999	-999	-999	-999	0.000
4	A	-999	-999	-999	-999	0.000	-999	-999	-999	-999	0.000
5	C	-999	-999	-999	-999	0.000	-999	-999	-999	-999	0.000
6	A	-999	-999	-999	-999	0.000	-999	-999	-999	-999	0.000
7	G	-999	-999	-999	-999	0.000	-999	-999	-999	-999	0.000
8	U	-999	-999	-999	-999	0.000	-999	-999	-999	-999	0.000
9	C	-999	-999	-999	-999	0.000	-999	-999	-999	-999	0.000
10	C	-999	-999	-999	-999	0.000	-999	-999	-999	-999	0.000
11	U	-999	-999	-999	-999	0.000	-999	-999	-999	-999	0.000
12	A	-999	-999	-999	-999	0.000	-999	-999	-999	-999	0.000
13	A	-999	-999	-999	-999	0.000	-999	-999	-999	-999	0.000
14	U	-999	-999	-999	-999	0.000	-999	-999	-999	-999	0.000
15	A	-999	-999	-999	-999	0.000	-999	-999	-999	-999	0.000
16	U	-999	-999	-999	-999	0.000	-999	-999	-999	-999	0.000
17	U	-999	-999	-999	-999	0.000	-999	-999	-999	-999	0.000
18	C	-999	-999	-999	-999	0.000	-999	-999	-999	-999	0.000
19	A	-999	-999	-999	-999	0.000	-999	-999	-999	-999	0.000
20	C	0.090	0.270	0.360	0.240	0.137	-999	-999	-999	-999	0.000
21	G	0.795	0.980	0.775	0.850	0.113	0.505	0.625	0.400	0.510	0.113
22	U	1.245	1.525	1.100	1.290	0.216	2.280	1.780	1.510	1.857	0.391
23	C	0.365	0.475	0.405	0.415	0.056	0.390	0.215	0.230	0.278	0.097
24	U	1.030	1.315	0.945	1.097	0.194	1.675	1.210	1.130	1.338	0.294
25	C	0.255	0.410	0.550	0.405	0.148	0.380	0.315	0.205	0.300	0.088
26	G	0.350	0.265	0.320	0.312	0.043	0.040	0.170	0.000	0.070	0.089
27	U	0.160	0.110	0.260	0.177	0.076	0.065	0.055	0.000	0.040	0.035
28	G	0.000	0.000	0.000	0.000	0.000	0.000	0.000	0.000	0.000	0.000
29	U	0.120	0.120	0.185	0.142	0.038	0.090	0.040	0.000	0.043	0.045
30	G	0.450	0.390	0.470	0.437	0.042	0.165	0.150	0.000	0.105	0.091
31	U	0.230	0.430	0.570	0.410	0.171	0.250	0.100	0.170	0.173	0.075
32	U	0.145	0.570	0.715	0.477	0.296	0.645	0.210	0.345	0.400	0.223
33	U	0.910	1.120	1.080	1.037	0.112	0.410	0.875	0.610	0.632	0.233
34	G	0.430	0.820	0.680	0.643	0.198	0.775	0.690	0.470	0.645	0.157
35	U	0.490	0.725	0.900	0.705	0.206	0.575	0.495	0.190	0.420	0.203
36	G	0.455	0.895	0.560	0.637	0.230	0.955	0.850	0.500	0.768	0.238
37	U → G	0.025	0.125	0.230	0.127	0.103	0.080	0.065	0.000	0.048	0.043
38	C	0.000	0.000	0.000	0.000	0.000	0.000	0.040	0.000	0.013	0.023
39	U → G	0.000	0.000	0.000	0.000	0.000	0.000	0.000	0.000	0.000	0.000
40	G	0.000	0.000	0.000	0.000	0.000	0.000	0.000	0.000	0.000	0.000
41	U	0.390	0.395	0.000	0.262	0.227	0.540	0.440	0.490	0.490	0.050
42	U	0.740	0.930	0.850	0.840	0.095	1.315	1.070	1.220	1.202	0.124
43	C	0.630	0.620	0.440	0.563	0.107	0.280	0.405	0.280	0.322	0.072
44	G	0.000	0.135	0.320	0.152	0.161	0.000	0.000	0.000	0.000	0.000
45	C	0.000	0.000	0.000	0.000	0.000	0.000	0.000	0.000	0.000	0.000
46	C	0.000	0.000	0.000	0.000	0.000	0.000	0.000	0.000	0.000	0.000
47	A	0.320	0.255	0.170	0.248	0.075	0.000	0.000	0.000	0.000	0.000
48	U	0.505	0.555	0.430	0.497	0.063	0.380	0.250	0.340	0.323	0.067

hSHAPE Reactivities from 3 independent experiments (SP101*i*)											
Nucleotides		In the Absence of Pr77[Gag]					In the Presence of Pr77[Gag]				
Number	Sequence	Expt 1	Expt 2	Expt 3	Mean	SD	Expt 1	Expt 2	Expt 3	Mean	SD
49	C	0.095	0.110	0.100	0.102	0.008	0.000	0.000	0.000	0.000	0.000
50	C	0.000	0.000	0.000	0.000	0.000	0.000	0.000	0.000	0.000	0.000
51	C	0.000	0.000	0.000	0.000	0.000	0.000	0.000	0.000	0.000	0.000
52	G	0.000	0.000	0.000	0.000	0.000	0.000	0.005	0.000	0.002	0.003
53	U	0.365	0.280	0.275	0.307	0.051	0.270	0.245	0.260	0.258	0.013
54	C	0.345	0.460	0.520	0.442	0.089	0.375	0.380	0.375	0.377	0.003
55	U	0.100	0.000	0.160	0.087	0.081	0.060	0.020	0.040	0.040	0.020
56	C	0.000	0.000	0.155	0.052	0.089	0.000	0.000	0.000	0.000	0.000
57	C	0.000	0.000	0.000	0.000	0.000	0.000	0.000	0.000	0.000	0.000
58	G	0.000	0.000	0.000	0.000	0.000	0.000	0.000	0.000	0.000	0.000
59	C	0.000	0.000	0.000	0.000	0.000	0.000	0.000	0.000	0.000	0.000
60	U	0.000	0.000	0.000	0.000	0.000	0.000	0.000	0.000	0.000	0.000
61	C	0.000	0.000	0.000	0.000	0.000	0.000	0.000	0.000	0.000	0.000
62	G	0.000	0.000	0.000	0.000	0.000	0.000	0.000	0.000	0.000	0.000
63	U	0.000	0.000	0.000	0.000	0.000	0.000	0.000	0.000	0.000	0.000
64	C	0.000	0.000	0.885	0.295	0.511	0.000	0.210	0.260	0.157	0.138
65	A	0.190	0.330	0.240	0.253	0.071	0.160	0.195	0.130	0.162	0.033
66	C	0.000	0.000	0.000	0.000	0.000	0.000	0.010	0.000	0.003	0.006
67	U	0.330	0.270	0.050	0.217	0.147	0.285	0.130	0.110	0.175	0.096
68	U	0.420	0.550	0.355	0.442	0.099	0.530	0.390	0.350	0.423	0.095
69	A	0.500	0.540	0.400	0.480	0.072	0.575	0.545	0.395	0.505	0.096
70	U	0.185	0.205	0.135	0.175	0.036	0.350	0.160	0.270	0.260	0.095
71	C	0.000	0.000	0.135	0.045	0.078	0.030	0.000	0.060	0.030	0.030
72	C	0.000	0.000	0.000	0.000	0.000	0.000	0.000	0.000	0.000	0.000
73	U	0.430	0.600	0.630	0.553	0.108	0.100	0.655	0.500	0.418	0.286
74	U	0.925	1.090	1.110	1.042	0.102	0.945	0.880	1.320	1.048	0.238
75	C	0.830	0.660	0.050	0.513	0.410	0.210	0.000	0.000	0.070	0.121
76	A	0.820	0.850	0.815	0.828	0.019	0.690	0.615	0.710	0.672	0.050
77	C	0.405	0.365	0.480	0.417	0.058	0.160	0.254	0.170	0.195	0.052
78	U	1.195	1.145	0.780	1.040	0.227	0.760	0.990	1.025	0.925	0.144
79	U	1.430	1.440	1.035	1.302	0.231	1.365	1.185	1.085	1.212	0.142
80	U	0.815	0.755	0.575	0.715	0.125	0.940	0.645	0.825	0.803	0.149
81	C	0.110	0.025	0.335	0.157	0.160	0.195	0.080	0.145	0.140	0.058
82	C	0.000	0.145	0.785	0.310	0.418	0.170	0.310	0.420	0.300	0.125
83	A	1.945	2.175	1.775	1.965	0.201	2.230	2.005	2.250	2.162	0.136
84	G	0.105	0.135	0.095	0.112	0.021	0.195	0.085	0.150	0.143	0.055
85	A	0.155	0.000	0.080	0.078	0.078	0.155	0.120	0.170	0.148	0.026
86	G	0.000	0.000	0.000	0.000	0.000	0.000	0.000	0.000	0.000	0.000
87	G	0.000	0.000	0.000	0.000	0.000	0.000	0.000	0.000	0.000	0.000
88	G	0.000	0.000	0.000	0.000	0.000	0.000	0.000	0.000	0.000	0.000
89	U	0.000	0.000	0.000	0.000	0.000	0.000	0.000	0.000	0.000	0.000
90	C	0.000	0.000	0.090	0.030	0.052	0.000	0.000	0.200	0.067	0.115
91	C	0.000	0.000	0.000	0.000	0.000	0.000	0.000	0.040	0.013	0.023
92	C	0.000	0.000	0.000	0.000	0.000	0.000	0.000	0.000	0.000	0.000
93	C	0.000	0.000	0.000	0.000	0.000	0.000	0.000	0.000	0.000	0.000
94	C	0.000	0.000	0.000	0.000	0.000	0.000	0.000	0.000	0.000	0.000
95	C	0.000	0.000	0.000	0.000	0.000	0.000	0.000	0.000	0.000	0.000
96	G	0.035	0.070	0.000	0.035	0.035	0.000	0.000	0.000	0.000	0.000

hSHAPE Reactivities from 3 independent experiments (SP101*i*)											
Nucleotides		In the Absence of Pr77^{Gag}					In the Presence of Pr77^{Gag}				
Number	Sequence	Expt 1	Expt 2	Expt 3	Mean	SD	Expt 1	Expt 2	Expt 3	Mean	SD
97	C	0.065	0.080	0.070	0.072	0.008	0.000	0.000	0.000	0.000	0.000
98	A	0.300	0.280	0.360	0.313	0.042	0.170	0.200	0.260	0.210	0.046
99	G	0.500	0.615	0.420	0.512	0.098	0.000	0.000	0.000	0.000	0.000
100	A	1.180	1.285	0.855	1.107	0.224	1.715	0.995	1.415	1.375	0.362
101	C	0.130	0.185	0.010	0.108	0.089	0.205	0.070	0.120	0.132	0.068
102	C	0.000	0.000	0.000	0.000	0.000	0.000	0.300	0.000	0.100	0.173
103	C	0.000	0.000	0.000	0.000	0.000	0.000	0.000	0.000	0.000	0.000
104	C	0.000	0.000	0.000	0.000	0.000	0.000	0.000	0.000	0.000	0.000
105	G	0.000	0.000	0.000	0.000	0.000	0.000	0.000	0.270	0.090	0.156
106	G	0.000	0.000	0.000	0.000	0.000	0.000	0.000	0.000	0.000	0.000
107	U	0.065	0.075	0.000	0.047	0.041	0.060	0.060	0.115	0.078	0.032
108	G	0.060	0.000	0.000	0.020	0.035	0.080	0.000	0.030	0.037	0.040
109	A	0.025	0.115	0.000	0.047	0.060	0.090	0.060	0.235	0.128	0.094
110	C	0.000	0.000	0.000	0.000	0.000	0.040	0.130	0.195	0.122	0.078
111	C	0.000	0.000	0.000	0.000	0.000	0.000	0.360	0.000	0.120	0.208
112	C	0.000	0.000	0.000	0.000	0.000	0.000	0.000	0.000	0.000	0.000
113	U	1.020	1.250	1.650	1.307	0.319	1.755	1.670	1.330	1.585	0.225
114	C	0.540	0.660	0.515	0.572	0.078	0.383	0.352	0.351	0.362	0.018
115	A	2.440	2.535	2.070	2.348	0.246	3.065	3.235	2.595	2.965	0.332
116	G	0.130	0.230	0.025	0.128	0.103	0.135	0.255	0.220	0.203	0.062
117	G	0.000	0.000	0.000	0.000	0.000	0.000	0.030	0.060	0.030	0.030
118	U	0.000	0.000	0.010	0.003	0.006	0.100	0.000	0.120	0.073	0.064
119	C	0.040	0.020	0.180	0.080	0.087	0.000	0.000	0.230	0.077	0.133
120	G	0.345	0.435	0.245	0.342	0.095	0.170	0.300	0.170	0.213	0.075
121	G	0.615	0.755	0.465	0.612	0.145	0.585	0.790	0.470	0.615	0.162
122	C	0.000	0.000	0.000	0.000	0.000	0.000	0.000	0.000	0.000	0.000
123	C	0.000	0.000	0.000	0.000	0.000	0.000	0.015	0.000	0.005	0.009
124	G	0.050	0.100	0.010	0.053	0.045	0.000	0.020	0.000	0.007	0.012
125	A	0.360	0.340	0.305	0.335	0.028	0.210	0.160	0.265	0.212	0.053
126	C	0.005	0.000	0.000	0.002	0.003	0.000	0.050	0.000	0.017	0.029
127	U	0.065	0.070	0.000	0.045	0.039	0.030	0.000	0.010	0.013	0.015
128	G	0.130	0.140	0.110	0.127	0.015	0.000	0.000	0.000	0.000	0.000
129	C	0.000	0.000	0.000	0.000	0.000	0.000	0.000	0.000	0.000	0.000
130	G	0.000	0.000	0.000	0.000	0.000	0.000	0.000	0.000	0.000	0.000
131	G	0.000	0.000	0.000	0.000	0.000	0.000	0.000	0.000	0.000	0.000
132	C	0.000	0.000	0.000	0.000	0.000	0.000	0.000	0.000	0.000	0.000
133	A	0.000	0.000	0.000	0.000	0.000	0.000	0.000	0.000	0.000	0.000
134	G	0.660	0.740	0.560	0.653	0.090	0.000	0.250	0.000	0.083	0.144
135	C	0.840	0.730	1.260	0.943	0.280	0.374	0.268	0.264	0.302	0.062
136	U	0.640	0.740	0.895	0.758	0.128	0.270	0.540	0.425	0.412	0.135
137	G	0.125	0.235	0.080	0.147	0.080	0.000	0.005	0.000	0.002	0.003
138	G	0.000	0.000	0.000	0.000	0.000	0.000	0.000	0.000	0.000	0.000
139	C	0.000	0.000	0.000	0.000	0.000	0.000	0.000	0.000	0.000	0.000
140	G	0.100	0.055	0.000	0.052	0.050	0.000	0.080	0.000	0.027	0.046
141	C	0.000	0.000	0.000	0.000	0.000	0.000	0.035	0.120	0.052	0.062
142	C	0.000	0.000	0.000	0.000	0.000	0.000	0.000	0.000	0.000	0.000
143	C	0.000	0.000	0.000	0.000	0.000	0.000	0.000	0.000	0.000	0.000
144	G	0.210	0.090	0.050	0.117	0.083	0.000	0.015	0.000	0.005	0.009

hSHAPE Reactivities from 3 independent experiments (SP101*i*)											
Nucleotides		In the Absence of Pr77^{Gag}					In the Presence of Pr77^{Gag}				
Number	Sequence	Expt 1	Expt 2	Expt 3	Mean	SD	Expt 1	Expt 2	Expt 3	Mean	SD
145	A	1.070	1.125	0.800	0.998	0.174	0.850	1.270	0.960	1.027	0.218
146	A	0.990	0.905	1.190	1.028	0.146	0.925	1.195	0.905	1.008	0.162
147	C	0.810	0.740	0.795	0.782	0.037	0.085	0.000	0.390	0.158	0.205
148	A	0.680	0.490	0.405	0.525	0.141	0.630	0.705	0.540	0.625	0.083
149	G	0.000	0.000	0.035	0.012	0.020	0.005	0.020	0.000	0.008	0.010
150	G	0.000	0.000	0.000	0.000	0.000	0.000	0.000	0.000	0.000	0.000
151	G	0.000	0.000	0.000	0.000	0.000	0.000	0.000	0.000	0.000	0.000
152	A	0.000	0.000	0.000	0.000	0.000	0.000	0.000	0.000	0.000	0.000
153	C	0.000	0.000	0.000	0.000	0.000	0.000	0.000	0.000	0.000	0.000
154	C	0.000	0.000	0.000	0.000	0.000	0.000	0.000	0.000	0.000	0.000
155	C	0.000	0.000	0.000	0.000	0.000	0.000	0.000	0.000	0.000	0.000
156	U	0.000	0.000	0.000	0.000	0.000	0.000	0.000	0.000	0.000	0.000
157	C	0.220	0.370	0.000	0.197	0.186	0.000	0.130	0.150	0.093	0.081
158	G	0.000	0.000	0.000	0.000	0.000	0.000	0.000	0.000	0.000	0.000
159	G	0.000	0.000	0.000	0.000	0.000	0.000	0.000	0.000	0.000	0.000
160	A	0.000	0.000	0.000	0.000	0.000	0.000	0.065	0.000	0.022	0.038
161	U	0.590	0.580	0.310	0.493	0.159	0.148	0.199	0.142	0.163	0.031
162	A	0.000	0.000	0.035	0.012	0.020	0.060	0.125	0.040	0.075	0.044
163	A	0.000	0.025	0.040	0.022	0.020	0.015	0.065	0.000	0.027	0.034
164	G	0.000	0.000	0.000	0.000	0.000	0.000	0.000	0.000	0.000	0.000
165	U	0.000	0.000	0.000	0.000	0.000	0.000	0.000	0.000	0.000	0.000
166	G	0.000	0.000	0.000	0.000	0.000	0.000	0.000	0.000	0.000	0.000
167	A	0.000	0.000	0.000	0.000	0.000	0.000	0.000	0.000	0.000	0.000
168	C	0.000	0.000	0.000	0.000	0.000	0.000	0.000	0.000	0.000	0.000
169	C	0.000	0.000	0.000	0.000	0.000	0.000	0.000	0.000	0.000	0.000
170	C	0.000	0.000	0.000	0.000	0.000	0.000	0.115	0.000	0.038	0.066
171	U	0.000	0.000	0.000	0.000	0.000	0.000	0.020	0.000	0.007	0.012
172	U	0.350	0.470	0.340	0.387	0.072	0.160	0.320	0.205	0.228	0.083
173	G	0.790	0.880	0.345	0.672	0.286	0.140	0.270	0.090	0.167	0.093
174	U	0.425	0.460	0.370	0.418	0.045	0.440	0.505	0.275	0.407	0.119
175	C	0.000	0.000	0.000	0.000	0.000	0.000	0.000	0.000	0.000	0.000
176	U	0.000	0.000	0.000	0.000	0.000	0.000	0.000	0.000	0.000	0.000
177	C	0.000	0.000	0.000	0.000	0.000	0.000	0.000	0.000	0.000	0.000
178	U	0.000	0.055	0.000	0.018	0.032	0.170	0.320	0.060	0.183	0.131
179	A	0.490	0.445	0.500	0.478	0.029	0.445	0.410	0.000	0.285	0.247
180	U	0.240	0.240	0.045	0.175	0.113	0.100	0.195	0.020	0.105	0.088
181	U	0.260	0.290	0.175	0.242	0.060	0.105	0.200	0.075	0.127	0.065
182	U	0.185	0.225	0.265	0.225	0.040	0.040	0.070	0.105	0.072	0.033
183	C	0.000	0.000	0.000	0.000	0.000	0.000	0.000	0.000	0.000	0.000
184	U	0.320	0.325	0.505	0.383	0.105	0.070	0.250	0.270	0.197	0.110
185	A	0.135	0.180	0.055	0.123	0.063	0.095	0.090	0.015	0.067	0.045
186	C	0.000	0.000	0.000	0.000	0.000	0.000	0.255	0.000	0.085	0.147
187	U	0.845	0.775	0.895	0.838	0.060	0.415	0.800	1.090	0.768	0.339
188	A	0.760	0.815	0.780	0.785	0.028	0.580	0.765	0.685	0.677	0.093
189	U	0.250	0.240	0.290	0.260	0.026	0.075	0.130	0.165	0.123	0.045
190	U	0.195	0.150	0.260	0.202	0.055	0.040	0.150	0.260	0.150	0.110
191	U	0.305	0.225	0.310	0.280	0.048	0.030	0.050	0.080	0.053	0.025
192	G	0.140	0.040	0.100	0.093	0.050	0.000	0.000	0.000	0.000	0.000

hSHAPE Reactivities from 3 independent experiments (SP101*i*)											
**Nucleotides**		**In the Absence of Pr77^{Gag}**					**In the Presence of Pr77^{Gag}**				
**Number**	**Sequence**	**Expt 1**	**Expt 2**	**Expt 3**	**Mean**	**SD**	**Expt 1**	**Expt 2**	**Expt 3**	**Mean**	**SD**
193	G	0.000	0.000	0.000	0.000	0.000	0.000	0.000	0.000	0.000	0.000
194	U	0.205	0.115	0.100	0.140	0.057	0.290	0.350	0.060	0.233	0.153
195	G	0.390	0.310	0.000	0.233	0.206	0.000	0.000	0.000	0.000	0.000
196	U	0.420	0.365	0.150	0.312	0.143	0.340	0.430	0.155	0.308	0.140
197	U	0.505	0.515	0.340	0.453	0.098	0.460	0.625	0.485	0.523	0.089
198	U	0.320	0.240	0.115	0.225	0.103	0.235	0.535	0.330	0.367	0.153
199	G	0.065	0.000	0.000	0.022	0.038	0.000	0.090	0.000	0.030	0.052
200	U	0.110	0.130	0.015	0.085	0.061	0.055	0.085	0.115	0.085	0.030
201	C	0.000	0.000	0.000	0.000	0.000	0.000	0.000	0.000	0.000	0.000
202	U	0.050	0.025	0.000	0.025	0.025	0.105	0.195	0.060	0.120	0.069
203	U	0.255	0.300	0.185	0.247	0.058	0.225	0.310	0.280	0.272	0.043
204	G	0.080	0.050	0.100	0.077	0.025	0.070	0.095	0.000	0.055	0.049
205	U	0.380	0.320	0.350	0.350	0.030	0.285	0.450	0.420	0.385	0.088
206	A	0.215	0.130	0.010	0.118	0.103	0.260	0.385	0.265	0.303	0.071
207	U	0.235	0.170	0.080	0.162	0.078	0.220	0.355	0.295	0.290	0.068
208	U	0.420	0.355	0.210	0.328	0.108	0.260	0.360	0.325	0.315	0.051
209	G	0.825	0.845	0.460	0.710	0.217	0.465	0.825	0.485	0.592	0.202
210	U	0.210	0.310	0.095	0.205	0.108	0.155	0.150	0.120	0.142	0.019
211	C	0.000	0.000	0.000	0.000	0.000	0.000	0.000	0.000	0.000	0.000
212	U	0.000	0.000	0.000	0.000	0.000	0.000	0.000	0.000	0.000	0.000
213	C	0.000	0.000	0.000	0.000	0.000	0.000	0.000	0.000	0.000	0.000
214	U	0.000	0.000	0.000	0.000	0.000	0.000	0.000	0.000	0.000	0.000
215	U	0.000	0.000	0.000	0.000	0.000	0.025	0.000	0.000	0.008	0.014
216	U	0.045	0.085	0.015	0.048	0.035	0.020	0.080	0.065	0.055	0.031
217	C	0.000	0.000	0.000	0.000	0.000	0.000	0.000	0.000	0.000	0.000
218	U	0.115	0.185	0.050	0.117	0.068	0.040	0.175	0.145	0.120	0.071
219	U	0.400	0.330	0.340	0.357	0.038	0.235	0.345	0.385	0.322	0.078
220	G	0.690	0.720	0.530	0.647	0.102	0.220	0.335	0.225	0.260	0.065
221	U	0.265	0.260	0.235	0.253	0.016	0.130	0.150	0.190	0.157	0.031
222	C	0.290	0.215	0.255	0.253	0.038	0.040	0.130	0.150	0.107	0.059
223	U	0.520	0.465	0.365	0.450	0.079	0.080	0.180	0.225	0.162	0.074
224	G	0.325	0.400	0.295	0.340	0.054	0.000	0.000	0.000	0.000	0.000
225	G	0.000	0.000	0.000	0.000	0.000	0.000	0.000	0.000	0.000	0.000
226	C	0.000	0.065	0.000	0.022	0.038	0.000	0.000	0.000	0.000	0.000
227	U	0.850	0.855	0.815	0.840	0.022	0.385	0.580	0.590	0.518	0.116
228	A	0.745	0.810	0.520	0.692	0.152	0.395	0.480	0.445	0.440	0.043
229	U	0.315	0.425	0.330	0.357	0.060	0.290	0.320	0.290	0.300	0.017
230	C	0.000	0.000	0.000	0.000	0.000	0.130	0.490	0.340	0.320	0.181
231	A	0.785	0.865	0.600	0.750	0.136	0.535	0.580	0.330	0.482	0.133
232	U	0.300	0.360	0.345	0.335	0.031	0.325	0.390	0.370	0.362	0.033
233	C	0.000	0.000	1.570	0.523	0.906	0.410	0.640	0.650	0.567	0.136
234	A	0.465	0.410	0.520	0.465	0.055	0.370	0.560	0.370	0.433	0.110
235	C	0.000	0.000	2.010	0.670	1.160	0.170	0.490	0.230	0.297	0.170
236	A	0.885	0.840	0.870	0.865	0.023	0.570	0.410	0.480	0.487	0.080
237	A	0.830	0.845	0.920	0.865	0.048	0.690	0.760	0.420	0.623	0.180
238	G	0.400	0.335	0.520	0.418	0.094	0.260	0.260	0.270	0.263	0.006
239	A	0.160	0.155	0.115	0.143	0.025	0.050	0.000	0.050	0.033	0.029
240	G	0.080	0.070	0.085	0.078	0.008	0.000	0.030	0.055	0.028	0.028

hSHAPE Reactivities from 3 independent experiments (SP101*i*)											
Nucleotides		In the Absence of Pr77^{Gag}					In the Presence of Pr77^{Gag}				
Number	Sequence	Expt 1	Expt 2	Expt 3	Mean	SD	Expt 1	Expt 2	Expt 3	Mean	SD
241	C	0.005	0.005	0.000	0.003	0.003	0.040	0.040	0.015	0.032	0.014
242	G	0.000	0.000	0.000	0.000	0.000	0.000	0.070	0.000	0.023	0.040
243	G	0.000	0.000	0.000	0.000	0.000	0.000	0.060	0.000	0.020	0.035
244	A	0.220	0.180	0.070	0.157	0.078	0.295	0.220	0.090	0.202	0.104
245	A	0.320	0.300	0.225	0.282	0.050	0.505	0.545	0.440	0.497	0.053
246	C	0.000	0.000	0.000	0.000	0.000	0.285	0.265	0.130	0.227	0.084
247	G	0.000	0.000	0.000	0.000	0.000	0.000	0.000	0.000	0.000	0.000
248	G	0.000	0.000	0.000	0.000	0.000	0.000	0.000	0.000	0.000	0.000
249	A	2.110	2.275	2.695	2.360	0.302	3.545	4.155	3.235	3.645	0.468
250	C	0.000	0.000	0.000	0.000	0.000	0.130	0.170	0.170	0.157	0.023
251	U	0.000	0.000	0.000	0.000	0.000	0.095	0.000	0.000	0.032	0.055
252	C	0.000	0.015	0.180	0.065	0.100	0.100	0.100	0.110	0.103	0.006
253	A	0.100	0.135	0.110	0.115	0.018	0.175	0.180	0.170	0.175	0.005
254	C	0.000	0.030	0.020	0.017	0.015	0.000	0.000	0.000	0.000	0.000
255	C	0.000	0.000	0.055	0.018	0.032	0.000	0.000	0.000	0.000	0.000
256	A	0.315	0.460	0.170	0.315	0.145	0.375	0.250	0.220	0.282	0.082
257	U	0.850	0.930	0.960	0.913	0.057	1.025	1.120	0.100	0.748	0.563
258	A	1.030	1.165	0.790	0.995	0.190	1.095	1.075	0.955	1.042	0.076
259	G	0.040	0.110	0.000	0.050	0.056	0.065	0.090	0.140	0.098	0.038
260	G	0.000	0.000	0.000	0.000	0.000	0.020	0.000	0.000	0.007	0.012
261	G	0.000	0.000	0.000	0.000	0.000	0.010	0.020	0.000	0.010	0.010
262	A	0.300	0.235	0.175	0.237	0.063	0.320	0.350	0.225	0.298	0.065
263	G	0.560	0.455	0.500	0.505	0.053	0.220	0.210	0.230	0.220	0.010
264	C	0.565	0.665	0.360	0.530	0.155	0.291	0.291	0.283	0.288	0.005
265	U	1.620	1.810	1.455	1.628	0.178	0.850	0.430	0.290	0.523	0.291
266	G	0.000	0.000	0.000	0.000	0.000	0.000	0.000	0.000	0.000	0.000
267	C	0.000	0.000	0.000	0.000	0.000	0.000	0.000	0.000	0.000	0.000
268	A	1.600	1.685	1.770	1.685	0.085	0.740	0.685	0.475	0.633	0.140
269	G	0.000	0.000	0.000	0.000	0.000	0.000	0.555	0.180	0.245	0.283
270	U	0.015	0.000	0.000	0.005	0.009	0.000	0.000	0.000	0.000	0.000
271	C	0.000	0.005	0.000	0.002	0.003	0.000	0.175	0.000	0.058	0.101
272	C	0.000	0.000	0.000	0.000	0.000	2.235	0.015	0.000	0.750	1.286
273	C	0.000	0.000	0.000	0.000	0.000	0.000	0.135	0.000	0.045	0.078
274	G	0.000	0.000	0.000	0.000	0.000	0.000	0.000	0.000	0.000	0.000
275	C	0.000	0.000	0.000	0.000	0.000	0.165	0.000	0.000	0.055	0.095
276	C	0.000	0.000	0.055	0.018	0.032	0.200	0.000	0.000	0.067	0.115
277	U	0.000	0.000	0.065	0.022	0.038	0.170	0.015	0.030	0.072	0.085
278	A	0.025	0.020	0.070	0.038	0.028	0.000	0.040	0.045	0.028	0.025
279	C	0.150	0.135	0.130	0.138	0.010	0.020	0.110	0.065	0.065	0.045
280	G	0.300	0.320	0.300	0.307	0.012	0.200	0.065	0.110	0.125	0.069
281	G	1.605	1.690	1.500	1.598	0.095	0.220	0.700	0.610	0.510	0.255
282	A	2.425	2.660	2.110	2.398	0.276	0.080	0.315	0.000	0.132	0.164
283	G	1.390	1.350	0.900	1.213	0.272	0.000	0.000	0.000	0.000	0.000
284	A	2.810	2.940	3.060	2.937	0.125	2.545	3.420	2.640	2.868	0.480
285	A	1.635	1.525	1.590	1.583	0.055	0.610	0.630	0.040	0.427	0.335
286	G	1.590	1.260	1.300	1.383	0.180	0.670	0.430	0.450	0.517	0.133
287	A	2.530	2.365	2.220	2.372	0.155	1.970	3.850	3.120	2.980	0.948
288	G	1.280	1.040	1.110	1.143	0.123	0.875	1.365	1.405	1.215	0.295

hSHAPE Reactivities from 3 independent experiments (SP101*i*)											
Nucleotides		In the Absence of Pr77^{Gag}					In the Presence of Pr77^{Gag}				
**Number**	**Sequence**	**Expt 1**	**Expt 2**	**Expt 3**	**Mean**	**SD**	**Expt 1**	**Expt 2**	**Expt 3**	**Mean**	**SD**
289	G	0.000	0.000	0.000	0.000	0.000	0.010	0.105	0.120	0.078	0.060
290	U	0.000	0.000	0.000	0.000	0.000	0.000	0.000	0.000	0.000	0.000
291	A	0.000	0.025	0.050	0.025	0.025	0.000	0.000	0.000	0.000	0.000
292	G	0.000	0.000	0.000	0.000	0.000	0.000	0.000	0.000	0.000	0.000
293	G	0.000	0.000	0.000	0.000	0.000	0.000	0.000	0.000	0.000	0.000
294	U	0.120	0.240	0.170	0.177	0.060	0.595	0.000	0.225	0.273	0.300
295	U	0.570	0.620	0.725	0.638	0.079	1.140	0.485	0.300	0.642	0.441
296	A	1.270	1.210	1.110	1.197	0.081	1.300	1.520	1.315	1.378	0.123
297	C	0.880	1.130	0.935	0.982	0.131	1.200	1.065	0.965	1.077	0.118
298	G	0.030	0.060	0.070	0.053	0.021	0.060	0.060	0.020	0.047	0.023
299	G	0.000	0.000	0.000	0.000	0.000	0.000	0.000	0.000	0.000	0.000
300	U	0.000	0.000	0.000	0.000	0.000	0.000	0.000	0.000	0.000	0.000
301	G	0.000	0.000	0.000	0.000	0.000	0.000	0.000	0.000	0.000	0.000
302	A	0.000	0.000	0.000	0.000	0.000	0.000	0.000	0.000	0.000	0.000
303	G	0.000	0.000	0.000	0.000	0.000	0.000	0.000	0.000	0.000	0.000
304	C	0.000	0.000	0.250	0.083	0.144	0.000	0.000	0.000	0.000	0.000
305	C	0.350	0.470	0.410	0.410	0.060	0.940	0.460	0.590	0.663	0.248
306	A	0.560	0.610	0.520	0.563	0.045	0.500	0.670	0.400	0.523	0.137
307	U	1.070	1.110	1.040	1.073	0.035	1.000	1.200	0.900	1.033	0.153
308	U	1.260	1.460	1.460	1.393	0.115	1.180	0.920	1.040	1.047	0.130
309	G	0.720	0.710	0.670	0.700	0.026	0.000	0.000	0.000	0.000	0.000
310	G	0.610	0.700	0.660	0.657	0.045	0.000	0.000	0.000	0.000	0.000
311	A	0.510	0.610	0.690	0.603	0.090	1.070	1.050	0.730	0.950	0.191
312	A	0.430	0.440	0.660	0.510	0.130	0.620	0.750	0.420	0.597	0.166
313	A	0.480	0.420	0.520	0.473	0.050	0.550	0.710	0.490	0.583	0.114
314	U	0.390	0.300	0.430	0.373	0.067	0.560	0.830	0.840	0.743	0.159
315	G	0.170	0.000	0.120	0.097	0.087	0.000	0.000	0.140	0.047	0.081
316	G	0.000	0.000	0.000	0.000	0.000	0.000	0.050	0.200	0.083	0.104
317	G	0.000	0.000	0.000	0.000	0.000	0.000	0.000	0.000	0.000	0.000
318	G	0.000	0.000	0.000	0.000	0.000	0.000	0.000	0.000	0.000	0.000
319	G	0.000	0.000	0.000	0.000	0.000	0.000	0.000	0.000	0.000	0.000
320	U	0.000	0.000	0.000	0.000	0.000	0.430	0.240	0.000	0.223	0.215
321	C	0.000	0.000	0.000	0.000	0.000	0.000	0.000	0.000	0.000	0.000
322	U	0.150	0.160	0.150	0.153	0.006	0.270	0.290	0.190	0.250	0.053
323	C	0.180	0.190	0.150	0.173	0.021	0.120	0.090	0.140	0.117	0.025
324	G	0.250	0.290	0.200	0.247	0.045	0.150	0.150	0.070	0.123	0.046
325	G	0.000	0.000	0.000	0.000	0.000	0.000	0.000	0.000	0.000	0.000
326	G	0.000	0.000	0.000	0.000	0.000	0.000	0.000	0.000	0.000	0.000
327	C	0.000	0.000	0.000	0.000	0.000	0.000	0.000	0.000	0.000	0.000
328	U	0.180	0.460	0.270	0.303	0.143	0.200	0.000	0.160	0.120	0.106
329	C	0.000	0.240	0.580	0.273	0.291	0.470	0.000	0.000	0.157	0.271
330	A	0.660	0.750	0.730	0.713	0.047	0.930	0.790	0.690	0.803	0.121
331	A	1.040	1.080	1.000	1.040	0.040	1.460	1.410	1.090	1.320	0.201
332	A	0.940	0.890	0.870	0.900	0.036	1.310	1.270	0.900	1.160	0.226
333	A	0.780	0.780	0.800	0.787	0.012	0.960	0.930	0.550	0.813	0.229
334	G	0.180	0.220	0.190	0.197	0.021	0.000	0.000	0.000	0.000	0.000
335	G	0.000	0.000	0.000	0.000	0.000	0.000	0.000	0.000	0.000	0.000
336	G	0.010	0.040	0.030	0.027	0.015	0.000	0.000	0.000	0.000	0.000

hSHAPE Reactivities from 3 independent experiments (SP101*i*)											
Nucleotides		In the Absence of $Pr77^{Gag}$					In the Presence of $Pr77^{Gag}$				
Number	Sequence	Expt 1	Expt 2	Expt 3	Mean	SD	Expt 1	Expt 2	Expt 3	Mean	SD
337	C	0.210	0.320	0.290	0.273	0.057	0.150	0.000	0.040	0.063	0.078
338	A	0.060	0.220	0.120	0.133	0.081	0.020	0.140	0.170	0.110	0.079
339	G	0.050	0.080	0.000	0.043	0.040	0.000	0.000	0.000	0.000	0.000
340	A	0.100	0.230	0.160	0.163	0.065	0.130	0.140	0.160	0.143	0.015
341	A	0.370	0.480	0.350	0.400	0.070	0.230	0.200	0.270	0.233	0.035
342	A	0.100	0.170	0.120	0.130	0.036	0.150	0.110	0.120	0.127	0.021
343	C	0.000	0.100	0.000	0.033	0.058	0.130	0.000	0.060	0.063	0.065
344	U	1.240	1.350	1.450	1.347	0.105	1.740	1.510	1.610	1.620	0.115
345	C	0.320	0.390	0.330	0.347	0.038	1.360	1.190	1.210	1.253	0.093
346	U	0.410	0.460	0.370	0.413	0.045	1.290	1.030	1.020	1.113	0.153
347	U	0.760	0.810	0.880	0.817	0.060	1.810	1.790	1.750	1.783	0.031
348	U	0.840	0.850	0.860	0.850	0.010	1.680	1.660	1.430	1.590	0.139
349	G	0.000	0.000	0.000	0.000	0.000	0.090	0.000	0.000	0.030	0.052
350	U	0.210	0.190	0.230	0.210	0.020	0.540	0.250	0.320	0.370	0.151
351	U	0.340	0.330	0.420	0.363	0.049	0.500	0.510	0.540	0.517	0.021
352	U	0.000	0.030	0.000	0.010	0.017	0.040	0.010	0.000	0.017	0.021
353	C	0.000	0.000	0.000	0.000	0.000	0.000	0.000	0.000	0.000	0.000
354	U	0.420	0.620	0.210	0.417	0.205	1.040	1.210	0.730	0.993	0.243
355	G	2.640	2.420	2.450	2.503	0.119	2.800	2.880	2.630	2.770	0.128
356	U	0.200	0.220	0.130	0.183	0.047	0.500	0.340	0.470	0.437	0.085
357	U	0.370	0.410	0.310	0.363	0.050	0.620	0.540	0.590	0.583	0.040
358	U	0.380	0.420	0.340	0.380	0.040	0.620	0.120	0.200	0.313	0.269
359	U	0.360	0.440	0.400	0.400	0.040	0.700	0.000	0.230	0.310	0.357
360	A	0.350	0.360	0.330	0.347	0.015	0.980	0.800	0.800	0.860	0.104
361	C	0.000	0.090	0.350	0.147	0.182	0.490	0.000	0.000	0.163	0.283
362	A	0.170	0.090	0.160	0.140	0.044	0.480	0.390	0.360	0.410	0.062
363	A	0.060	0.000	0.100	0.053	0.050	0.210	0.180	0.180	0.190	0.017
364	A	0.010	0.000	0.000	0.003	0.006	0.080	0.040	0.050	0.057	0.021
365	G	0.000	0.000	0.000	0.000	0.000	0.000	0.000	0.030	0.010	0.017
366	G	0.000	0.000	0.010	0.003	0.006	0.010	0.000	0.040	0.017	0.021
367	C	0.000	0.000	0.000	0.000	0.000	0.000	0.000	0.040	0.013	0.023
368	U	0.000	0.000	0.000	0.000	0.000	0.000	0.000	0.060	0.020	0.035
369	C	0.000	0.000	0.000	0.000	0.000	0.000	0.000	0.000	0.000	0.000
370	C	0.000	0.000	0.000	0.000	0.000	0.000	0.000	0.130	0.043	0.075
371	U	0.010	0.000	0.000	0.003	0.006	0.000	0.000	0.020	0.007	0.012
372	C	0.120	0.190	0.100	0.137	0.047	0.080	0.000	0.000	0.027	0.046
373	U	0.470	0.520	0.530	0.507	0.032	0.620	0.300	0.350	0.423	0.172
374	C	2.040	2.350	2.360	2.250	0.182	2.530	1.400	1.880	1.937	0.567
375	A	2.620	2.610	2.740	2.657	0.072	2.840	0.820	1.880	1.847	1.010
376	G	2.120	2.000	2.260	2.127	0.130	1.750	0.000	0.490	0.747	0.903
377	A	2.600	2.490	2.730	2.607	0.120	2.930	2.970	2.300	2.733	0.376
378	G	0.000	0.010	0.040	0.017	0.021	0.020	0.000	0.000	0.007	0.012
379	A	0.000	0.000	0.000	0.000	0.000	0.000	0.000	0.000	0.000	0.000
380	G	0.000	0.000	0.000	0.000	0.000	0.000	0.120	0.000	0.040	0.069
381	G	0.000	0.000	0.000	0.000	0.000	0.430	0.000	0.000	0.143	0.248
382	G	0.000	0.000	0.000	0.000	0.000	0.000	0.000	0.000	0.000	0.000
383	G	1.820	0.000	0.000	0.607	1.051	0.000	0.000	0.000	0.000	0.000
384	U	0.000	0.000	0.000	0.000	0.000	0.540	0.670	0.760	0.657	0.111

hSHAPE Reactivities from 3 independent experiments (SP101*i*)											
Nucleotides		In the Absence of Pr77^{Gag}					In the Presence of Pr77^{Gag}				
Number	Sequence	Expt 1	Expt 2	Expt 3	Mean	SD	Expt 1	Expt 2	Expt 3	Mean	SD
385	C	0.000	0.150	0.000	0.050	0.087	0.300	0.150	0.140	0.197	0.090
386	U	0.110	0.790	0.150	0.350	0.382	0.600	0.040	0.450	0.363	0.290
387	U	0.640	0.940	0.980	0.853	0.186	1.240	0.000	0.000	0.413	0.716
388	C	0.000	1.040	1.520	0.853	0.777	0.840	0.000	0.600	0.480	0.433
389	A	1.050	0.050	1.080	0.727	0.586	0.000	0.400	0.000	0.133	0.231
390	U	0.120	0.000	0.050	0.057	0.060	0.000	0.000	0.040	0.013	0.023
391	G	0.020	0.010	0.000	0.010	0.010	0.030	0.070	0.000	0.033	0.035
392	U	0.030	0.040	0.000	0.023	0.021	0.000	0.030	0.000	0.010	0.017
393	G	0.060	0.080	0.050	0.063	0.015	0.230	0.000	0.110	0.113	0.115
394	A	0.080	0.210	0.140	0.143	0.065	0.410	0.100	0.320	0.277	0.159
395	A	0.260	0.440	0.260	0.320	0.104	0.760	0.370	0.760	0.630	0.225
396	A	0.570	0.730	0.540	0.613	0.102	0.790	0.890	0.390	0.690	0.265
397	G	0.770	0.170	0.820	0.587	0.362	0.190	0.660	0.120	0.323	0.294
398	A	0.210	0.250	0.240	0.233	0.021	0.000	0.350	0.130	0.160	0.177
399	G	0.310	0.420	0.200	0.310	0.110	0.000	0.460	0.000	0.153	0.266
400	A	0.390	0.370	0.330	0.363	0.031	0.000	0.440	0.000	0.147	0.254
401	G	0.440	1.610	0.430	0.827	0.678	0.000	0.370	0.000	0.123	0.214
402	U	1.200	0.750	1.660	1.203	0.455	0.140	0.000	0.460	0.200	0.236
403	A	0.880	0.480	0.820	0.727	0.216	0.220	1.280	1.570	1.023	0.711
404	G	0.550	0.490	0.450	0.497	0.050	0.340	0.150	1.010	0.500	0.452
405	U	0.590	0.600	0.500	0.563	0.055	0.980	0.090	1.730	0.933	0.821
406	G	0.570	0.510	0.600	0.560	0.046	1.030	0.000	0.000	0.343	0.595
407	C	0.000	0.500	0.790	0.430	0.400	0.570	0.000	0.120	0.230	0.300
408	A	0.390	0.650	0.360	0.467	0.159	0.580	0.000	0.400	0.327	0.297
409	A	0.630	1.380	0.550	0.853	0.458	0.680	0.260	0.000	0.313	0.343
410	U	0.900	0.500	1.070	0.823	0.293	0.800	0.000	0.180	0.327	0.420
411	A	0.500	0.310	0.660	0.490	0.175	0.070	0.740	0.000	0.270	0.409
412	G	0.300	0.300	0.480	0.360	0.104	0.550	0.000	0.350	0.300	0.278
413	A	0.390	0.320	0.490	0.400	0.085	0.480	0.500	0.400	0.460	0.053
414	A	0.410	0.460	0.520	0.463	0.055	0.510	0.570	0.410	0.497	0.081
415	U	0.480	0.300	0.630	0.470	0.165	0.280	0.470	0.010	0.253	0.231
416	U	0.250	0.680	0.390	0.440	0.219	0.870	0.000	0.390	0.420	0.436
417	U	0.630	1.080	0.880	0.863	0.225	0.870	0.420	0.000	0.430	0.435
418	U	0.700	0.820	1.320	0.947	0.329	0.880	0.000	0.590	0.490	0.448
419	A	0.860	0.510	0.860	0.743	0.202	0.160	0.620	0.170	0.317	0.263
420	U	0.200	0.690	0.380	0.423	0.248	0.000	0.000	0.000	0.000	0.000
421	C	1.180	0.640	1.170	0.997	0.309	0.000	0.000	0.000	0.000	0.000
422	A	0.620	0.300	0.680	0.533	0.204	0.760	0.000	0.360	0.373	0.380
423	G	0.360	0.140	0.430	0.310	0.151	0.490	0.150	0.250	0.297	0.175
424	U	0.290	0.000	0.310	0.200	0.173	0.450	0.150	0.000	0.200	0.229
425	U	0.140	0.080	0.140	0.120	0.035	0.050	0.010	0.000	0.020	0.026
426	U	0.020	0.770	0.030	0.273	0.430	-999	-999	-999	-999	0.000
427	C	0.310	0.460	0.630	0.467	0.160	-999	-999	-999	-999	0.000
428	U	-999	-999	-999	-999	0.000	-999	-999	-999	-999	0.000
429	A	-999	-999	-999	-999	0.000	-999	-999	-999	-999	0.000
430	A	-999	-999	-999	-999	0.000	-999	-999	-999	-999	0.000
431	U	-999	-999	-999	-999	0.000	-999	-999	-999	-999	0.000
432	A	-999	-999	-999	-999	0.000	-999	-999	-999	-999	0.000

hSHAPE Reactivities from 3 independent experiments (SP102*i*)											
Nucleotides		In the Absence of Pr77^{Gag}					In the Presence of Pr77^{Gag}				
Number	Sequence	Expt 1	Expt 2	Expt 3	Mean	SD	Expt 1	Expt 2	Expt 3	Mean	SD
1	G	-999	-999	-999	-999	0.000	-999	-999	-999	-999	0.000
2	C	-999	-999	-999	-999	0.000	-999	-999	-999	-999	0.000
3	A	-999	-999	-999	-999	0.000	-999	-999	-999	-999	0.000
4	A	-999	-999	-999	-999	0.000	-999	-999	-999	-999	0.000
5	C	-999	-999	-999	-999	0.000	-999	-999	-999	-999	0.000
6	A	-999	-999	-999	-999	0.000	-999	-999	-999	-999	0.000
7	G	-999	-999	-999	-999	0.000	-999	-999	-999	-999	0.000
8	U	-999	-999	-999	-999	0.000	-999	-999	-999	-999	0.000
9	C	-999	-999	-999	-999	0.000	-999	-999	-999	-999	0.000
10	C	-999	-999	-999	-999	0.000	-999	-999	-999	-999	0.000
11	U	-999	-999	-999	-999	0.000	-999	-999	-999	-999	0.000
12	A	-999	-999	-999	-999	0.000	-999	-999	-999	-999	0.000
13	A	-999	-999	-999	-999	0.000	-999	-999	-999	-999	0.000
14	U	-999	-999	-999	-999	0.000	-999	-999	-999	-999	0.000
15	A	-999	-999	-999	-999	0.000	-999	-999	-999	-999	0.000
16	U	-999	-999	-999	-999	0.000	-999	-999	-999	-999	0.000
17	U	0.775	0.355	0.450	0.527	0.220	-999	-999	-999	-999	0.000
18	C	0.000	0.000	0.000	0.000	0.000	-999	-999	-999	-999	0.000
19	A	0.650	0.485	0.400	0.512	0.127	0.500	0.630	0.530	0.553	0.068
20	C	0.110	0.215	0.200	0.175	0.057	0.115	0.320	0.085	0.173	0.128
21	G	0.770	0.630	0.360	0.587	0.208	0.675	0.515	0.630	0.607	0.083
22	U	2.065	1.980	1.670	1.905	0.208	1.840	2.060	2.125	2.008	0.149
23	C	0.460	0.480	0.450	0.463	0.015	0.435	0.740	0.395	0.523	0.189
24	U	2.775	2.350	2.125	2.417	0.330	2.450	2.140	2.480	2.357	0.188
25	C	0.610	0.570	0.220	0.467	0.215	0.325	0.710	0.355	0.463	0.214
26	G	0.314	0.400	0.687	0.467	0.195	0.080	0.080	0.060	0.073	0.012
27	U	0.170	0.130	0.200	0.167	0.035	0.095	0.000	0.040	0.045	0.048
28	G	0.040	0.000	0.000	0.013	0.023	0.010	0.000	0.030	0.013	0.015
29	U	0.110	0.070	0.000	0.060	0.056	0.000	0.000	0.000	0.000	0.000
30	G	0.510	0.550	0.330	0.463	0.117	0.100	0.000	0.100	0.067	0.058
31	U	0.550	0.260	0.190	0.333	0.191	0.080	0.410	0.060	0.183	0.197
32	U	0.310	0.550	0.330	0.397	0.133	0.060	0.195	0.110	0.122	0.068
33	U	1.445	1.190	1.070	1.235	0.192	0.935	0.900	0.685	0.840	0.135
34	G	1.165	0.670	0.620	0.818	0.301	0.700	0.510	0.410	0.540	0.147
35	U	1.230	0.770	0.790	0.930	0.260	0.645	0.415	0.450	0.503	0.124
36	G	1.615	1.065	1.210	1.297	0.285	1.400	1.140	1.060	1.200	0.178
37	U → G	0.170	0.130	0.170	0.157	0.023	0.100	0.140	0.000	0.080	0.072
38	C	0.120	0.220	0.140	0.160	0.053	0.180	0.080	0.000	0.087	0.090
39	U → G	0.000	0.000	0.000	0.000	0.000	0.000	0.000	0.000	0.000	0.000
40	G	0.000	0.000	0.000	0.000	0.000	0.000	0.000	0.000	0.000	0.000
41	U	0.335	0.105	0.040	0.160	0.155	0.270	0.300	0.200	0.257	0.051
42	U	0.855	0.815	0.625	0.765	0.123	0.895	0.710	0.820	0.808	0.093
43	C	0.495	0.500	0.590	0.528	0.053	0.560	0.280	0.430	0.423	0.140
44	G	0.675	0.475	0.380	0.510	0.151	0.110	0.040	0.130	0.093	0.047
45	C	0.000	0.000	0.000	0.000	0.000	0.000	0.000	0.000	0.000	0.000
46	C	0.000	0.000	0.000	0.000	0.000	0.000	0.000	0.000	0.000	0.000
47	A	0.760	0.620	0.335	0.572	0.217	0.735	0.355	0.560	0.550	0.190
48	U	0.620	0.555	0.600	0.592	0.033	0.635	0.530	0.485	0.550	0.077

hSHAPE Reactivities from 3 independent experiments (SP102*i*)											
Nucleotides		In the Absence of $Pr77^{Gag}$					In the Presence of $Pr77^{Gag}$				
Number	Sequence	Expt 1	Expt 2	Expt 3	Mean	SD	Expt 1	Expt 2	Expt 3	Mean	SD
49	C	0.000	0.000	0.010	0.003	0.006	0.065	0.020	0.110	0.065	0.045
50	C	0.000	0.000	0.000	0.000	0.000	0.000	0.000	0.000	0.000	0.000
51	C	0.000	0.000	0.000	0.000	0.000	0.000	0.000	0.000	0.000	0.000
52	G	0.240	0.290	0.360	0.297	0.060	0.000	0.010	0.170	0.060	0.095
53	U	0.430	0.465	0.295	0.397	0.090	0.575	0.385	0.415	0.458	0.102
54	C	0.470	0.525	0.505	0.500	0.028	0.500	0.360	0.525	0.462	0.089
55	U	0.025	0.080	0.050	0.052	0.028	0.135	0.020	0.090	0.082	0.058
56	C	0.000	0.000	0.000	0.000	0.000	0.000	0.000	0.000	0.000	0.000
57	C	0.000	0.000	0.000	0.000	0.000	0.000	0.000	0.000	0.000	0.000
58	G	0.000	0.000	0.000	0.000	0.000	0.000	0.000	0.000	0.000	0.000
59	C	0.000	0.000	0.000	0.000	0.000	0.000	0.000	0.000	0.000	0.000
60	U	0.000	0.000	0.000	0.000	0.000	0.000	0.000	0.000	0.000	0.000
61	C	0.000	0.000	0.000	0.000	0.000	0.000	0.000	0.000	0.000	0.000
62	G	0.000	0.000	0.000	0.000	0.000	0.000	0.000	0.000	0.000	0.000
63	U	0.145	0.005	0.145	0.098	0.081	0.050	0.080	0.295	0.142	0.134
64	C	0.000	0.000	0.140	0.047	0.081	0.180	0.120	0.250	0.183	0.065
65	A	0.335	0.325	0.410	0.357	0.046	0.310	0.200	0.410	0.307	0.105
66	C	0.020	0.010	0.000	0.010	0.010	0.000	0.000	0.000	0.000	0.000
67	U	0.390	0.360	0.300	0.350	0.046	0.220	0.180	0.265	0.222	0.043
68	U	0.530	0.465	0.350	0.448	0.091	0.440	0.295	0.400	0.378	0.075
69	A	0.800	0.810	0.715	0.775	0.052	0.515	0.485	0.470	0.490	0.023
70	U	0.380	0.385	0.380	0.382	0.003	0.265	0.290	0.330	0.295	0.033
71	C	0.190	0.000	0.080	0.090	0.095	0.010	0.010	0.060	0.027	0.029
72	C	0.205	0.000	0.000	0.068	0.118	0.000	0.000	0.000	0.000	0.000
73	U	1.150	1.030	0.960	1.047	0.096	0.945	0.650	0.940	0.845	0.169
74	U	1.810	1.460	1.530	1.600	0.185	1.975	1.790	1.945	1.903	0.099
75	C	0.000	0.000	0.000	0.000	0.000	0.000	0.000	0.000	0.000	0.000
76	A	1.675	1.520	1.360	1.518	0.158	1.325	1.185	1.670	1.393	0.250
77	C	0.485	0.370	0.590	0.482	0.110	0.280	0.145	0.175	0.200	0.071
78	U	1.695	1.670	1.640	1.668	0.028	1.375	1.390	1.295	1.353	0.051
79	U	2.220	2.015	1.680	1.972	0.273	1.540	1.760	1.840	1.713	0.155
80	U	1.290	1.085	1.310	1.228	0.125	0.775	1.020	0.950	0.915	0.126
81	C	0.290	0.240	0.220	0.250	0.036	0.055	0.050	0.050	0.052	0.003
82	C	0.000	0.000	0.365	0.122	0.211	0.470	0.260	0.250	0.327	0.124
83	A	2.620	2.270	2.910	2.600	0.320	2.270	2.405	2.445	2.373	0.092
84	G	0.215	0.130	0.190	0.178	0.044	0.230	0.145	0.110	0.162	0.062
85	A	0.040	0.045	0.070	0.052	0.016	0.120	0.050	0.030	0.067	0.047
86	G	0.000	0.000	0.000	0.000	0.000	0.000	0.000	0.000	0.000	0.000
87	G	0.000	0.000	0.000	0.000	0.000	0.000	0.000	0.000	0.000	0.000
88	G	0.000	0.000	0.000	0.000	0.000	0.000	0.000	0.000	0.000	0.000
89	U	0.000	0.000	0.000	0.000	0.000	0.000	0.000	0.000	0.000	0.000
90	C	0.000	0.000	0.000	0.000	0.000	0.000	0.000	0.000	0.000	0.000
91	C	0.000	0.000	0.000	0.000	0.000	0.000	0.000	0.000	0.000	0.000
92	C	0.000	0.000	0.000	0.000	0.000	0.000	0.000	0.000	0.000	0.000
93	C	0.000	0.000	0.000	0.000	0.000	0.000	0.000	0.000	0.000	0.000
94	C	0.000	0.000	0.000	0.000	0.000	0.000	0.000	0.000	0.000	0.000
95	C	0.000	0.000	0.000	0.000	0.000	0.000	0.000	0.000	0.000	0.000
96	G	0.020	0.000	0.000	0.007	0.012	0.000	0.000	0.000	0.000	0.000

hSHAPE Reactivities from 3 independent experiments (SP102*i*)											
Nucleotides		In the Absence of Pr77^{Gag}					In the Presence of Pr77^{Gag}				
Number	Sequence	Expt 1	Expt 2	Expt 3	Mean	SD	Expt 1	Expt 2	Expt 3	Mean	SD
97	C	0.170	0.110	0.120	0.133	0.032	0.200	0.030	0.210	0.147	0.101
98	A	0.635	0.405	0.655	0.565	0.139	0.187	0.205	0.321	0.238	0.073
99	G	0.520	0.730	0.710	0.653	0.116	1.000	0.910	0.760	0.890	0.121
100	A	2.210	2.080	2.275	2.188	0.099	2.040	2.095	1.985	2.040	0.055
101	C	0.220	0.155	0.120	0.165	0.051	0.125	0.125	0.140	0.130	0.009
102	C	0.225	0.000	0.000	0.075	0.130	0.195	0.100	0.275	0.190	0.088
103	C	0.000	0.000	0.000	0.000	0.000	0.000	0.000	0.000	0.000	0.000
104	C	0.000	0.000	0.000	0.000	0.000	0.000	0.000	0.000	0.000	0.000
105	G	0.000	0.000	0.000	0.000	0.000	0.090	0.000	0.030	0.040	0.046
106	G	0.000	0.000	0.000	0.000	0.000	0.000	0.000	0.000	0.000	0.000
107	U	0.165	0.140	0.110	0.138	0.028	0.265	0.135	0.105	0.168	0.085
108	G	0.075	0.000	0.055	0.043	0.039	0.000	0.000	0.000	0.000	0.000
109	A	0.045	0.040	0.070	0.052	0.016	0.000	0.030	0.030	0.020	0.017
110	C	0.000	0.000	0.000	0.000	0.000	0.000	0.000	0.000	0.000	0.000
111	C	0.000	0.000	0.000	0.000	0.000	0.160	0.000	0.260	0.140	0.131
112	C	0.000	0.000	0.000	0.000	0.000	0.000	0.000	0.000	0.000	0.000
113	U	2.745	2.225	2.370	2.447	0.268	2.545	2.475	2.775	2.598	0.157
114	C	1.095	0.825	0.785	0.902	0.169	0.980	0.855	0.940	0.925	0.064
115	A	4.140	3.820	3.795	3.918	0.192	3.730	4.020	3.850	3.867	0.146
116	G	0.240	0.300	0.260	0.267	0.031	0.185	0.345	0.155	0.228	0.102
117	G	0.000	0.000	0.000	0.000	0.000	0.105	0.000	0.000	0.035	0.061
118	U	0.035	0.000	0.000	0.012	0.020	0.000	0.000	0.015	0.005	0.009
119	C	0.050	0.010	0.280	0.113	0.146	0.180	0.000	0.050	0.077	0.093
120	G	0.700	0.495	0.625	0.607	0.104	0.365	0.390	0.430	0.395	0.033
121	G	1.075	0.940	0.970	0.995	0.071	0.760	0.630	0.895	0.762	0.133
122	C	0.000	0.000	0.000	0.000	0.000	0.000	0.000	0.000	0.000	0.000
123	C	0.000	0.000	0.000	0.000	0.000	0.000	0.000	0.000	0.000	0.000
124	G	0.070	0.000	0.050	0.040	0.036	0.000	0.010	0.045	0.018	0.024
125	A	0.445	0.410	0.435	0.430	0.018	0.190	0.355	0.380	0.308	0.103
126	C	0.115	0.025	0.020	0.053	0.053	0.000	0.000	0.000	0.000	0.000
127	U	0.215	0.120	0.110	0.148	0.058	0.090	0.000	0.030	0.040	0.046
128	G	0.000	0.000	0.000	0.000	0.000	0.000	0.000	0.000	0.000	0.000
129	C	0.020	0.080	0.090	0.063	0.038	0.000	0.000	0.000	0.000	0.000
130	G	0.000	0.000	0.000	0.000	0.000	0.000	0.000	0.000	0.000	0.000
131	G	0.000	0.000	0.000	0.000	0.000	0.000	0.000	0.000	0.000	0.000
132	C	0.000	0.000	0.000	0.000	0.000	0.000	0.000	0.000	0.000	0.000
133	A	0.000	0.000	0.000	0.000	0.000	0.250	0.310	0.150	0.237	0.081
134	G	1.130	0.770	1.010	0.970	0.183	0.910	0.630	0.790	0.777	0.140
135	C	0.490	0.590	0.320	0.467	0.137	0.200	0.208	0.147	0.185	0.033
136	U	0.805	0.690	0.815	0.770	0.069	0.470	0.705	0.475	0.550	0.134
137	G	0.150	0.170	0.120	0.147	0.025	0.000	0.000	0.000	0.000	0.000
138	G	0.000	0.000	0.000	0.000	0.000	0.000	0.000	0.000	0.000	0.000
139	C	0.120	0.000	0.020	0.047	0.064	0.000	0.000	0.000	0.000	0.000
140	G	0.115	0.050	0.020	0.062	0.049	0.000	0.000	0.000	0.000	0.000
141	C	0.000	0.000	0.000	0.000	0.000	0.000	0.000	0.000	0.000	0.000
142	C	0.000	0.000	0.000	0.000	0.000	0.000	0.000	0.000	0.000	0.000
143	C	0.000	0.000	0.000	0.000	0.000	0.000	0.000	0.000	0.000	0.000
144	G	0.200	0.315	0.340	0.285	0.075	0.710	0.590	0.340	0.547	0.189

hSHAPE Reactivities from 3 independent experiments (SP102*i*)											
Nucleotides		In the Absence of Pr77^{Gag}					In the Presence of Pr77^{Gag}				
**Number**	**Sequence**	**Expt 1**	**Expt 2**	**Expt 3**	**Mean**	**SD**	**Expt 1**	**Expt 2**	**Expt 3**	**Mean**	**SD**
145	A	1.765	1.545	1.355	1.555	0.205	1.205	1.485	1.170	1.287	0.173
146	A	1.570	1.260	1.420	1.417	0.155	1.245	1.530	1.010	1.262	0.260
147	C	0.000	0.000	0.090	0.030	0.052	0.140	0.000	0.000	0.047	0.081
148	A	0.815	0.795	0.795	0.802	0.012	0.710	0.770	0.760	0.747	0.032
149	G	0.000	0.035	0.020	0.018	0.018	0.010	0.000	0.010	0.007	0.006
150	G	0.000	0.000	0.000	0.000	0.000	0.000	0.000	0.000	0.000	0.000
151	G	0.000	0.000	0.000	0.000	0.000	0.000	0.000	0.000	0.000	0.000
152	A	0.000	0.000	0.000	0.000	0.000	0.000	0.000	0.000	0.000	0.000
153	C	0.000	0.000	0.000	0.000	0.000	0.000	0.000	0.000	0.000	0.000
154	C	0.000	0.000	0.000	0.000	0.000	0.000	0.000	0.000	0.000	0.000
155	C	0.000	0.000	0.000	0.000	0.000	0.000	0.000	0.000	0.000	0.000
156	U	0.000	0.000	0.000	0.000	0.000	0.000	0.000	0.000	0.000	0.000
157	C	0.335	0.295	0.440	0.357	0.075	0.165	0.170	0.295	0.210	0.074
158	G	0.000	0.000	0.000	0.000	0.000	0.000	0.000	0.000	0.000	0.000
159	G	0.000	0.000	0.000	0.000	0.000	0.000	0.000	0.000	0.000	0.000
160	A	0.000	0.000	0.000	0.000	0.000	0.000	0.000	0.000	0.000	0.000
161	U	0.250	0.280	0.235	0.255	0.023	0.195	0.185	0.195	0.192	0.006
162	A	0.025	0.000	0.005	0.010	0.013	0.000	0.010	0.000	0.003	0.006
163	A	0.010	0.120	0.000	0.043	0.067	0.000	0.000	0.000	0.000	0.000
164	G	0.000	0.000	0.000	0.000	0.000	0.000	0.000	0.000	0.000	0.000
165	U	0.000	0.000	0.000	0.000	0.000	0.000	0.000	0.000	0.000	0.000
166	G	0.000	0.000	0.000	0.000	0.000	0.000	0.000	0.000	0.000	0.000
167	A	0.000	0.000	0.000	0.000	0.000	0.000	0.000	0.000	0.000	0.000
168	C	0.000	0.000	0.000	0.000	0.000	0.000	0.000	0.000	0.000	0.000
169	C	0.000	0.000	0.000	0.000	0.000	0.000	0.030	0.135	0.055	0.071
170	C	0.000	0.000	0.000	0.000	0.000	0.000	0.000	0.000	0.000	0.000
171	U	0.320	0.620	0.280	0.407	0.186	0.480	1.050	0.480	0.670	0.329
172	U	0.420	0.405	0.460	0.428	0.028	0.370	0.490	0.440	0.433	0.060
173	G	1.120	0.605	0.655	0.793	0.284	0.710	0.310	0.650	0.557	0.216
174	U	0.530	0.500	0.455	0.495	0.038	0.420	0.455	0.465	0.447	0.024
175	C	0.000	0.000	0.000	0.000	0.000	0.000	0.000	0.000	0.000	0.000
176	U	0.000	0.000	0.000	0.000	0.000	0.000	0.000	0.000	0.000	0.000
177	C	0.000	0.000	0.000	0.000	0.000	0.000	0.000	0.000	0.000	0.000
178	U	0.000	0.000	0.000	0.000	0.000	0.390	0.300	0.360	0.350	0.046
179	A	0.565	0.610	0.600	0.592	0.024	0.890	0.580	0.520	0.663	0.199
180	U	0.125	0.100	0.090	0.105	0.018	0.175	0.065	0.070	0.103	0.062
181	U	0.140	0.115	0.100	0.118	0.020	0.180	0.175	0.100	0.152	0.045
182	U	0.060	0.100	0.090	0.083	0.021	0.150	0.135	0.100	0.128	0.026
183	C	0.000	0.000	0.000	0.000	0.000	0.090	0.000	0.040	0.043	0.045
184	U	0.140	0.190	0.090	0.140	0.050	0.130	0.180	0.165	0.158	0.026
185	A	0.170	0.165	0.110	0.148	0.033	0.180	0.120	0.250	0.183	0.065
186	C	0.000	0.040	0.000	0.013	0.023	0.205	0.130	0.350	0.228	0.112
187	U	0.150	0.190	0.365	0.235	0.114	0.660	0.705	0.670	0.678	0.024
188	A	0.950	0.810	0.830	0.863	0.076	0.860	0.865	0.820	0.848	0.025
189	U	0.150	0.145	0.185	0.160	0.022	0.130	0.130	0.130	0.130	0.000
190	U	0.220	0.140	0.180	0.180	0.040	0.100	0.090	0.020	0.070	0.044
191	U	0.190	0.170	0.100	0.153	0.047	0.015	0.040	0.000	0.018	0.020
192	G	0.000	0.000	0.000	0.000	0.000	0.000	0.000	0.000	0.000	0.000

hSHAPE Reactivities from 3 independent experiments (SP102*i*)											
**Nucleotides**		**In the Absence of Pr77^{Gag}**					**In the Presence of Pr77^{Gag}**				
**Number**	**Sequence**	**Expt 1**	**Expt 2**	**Expt 3**	**Mean**	**SD**	**Expt 1**	**Expt 2**	**Expt 3**	**Mean**	**SD**
193	G	0.150	0.050	0.000	0.067	0.076	0.000	0.000	0.000	0.000	0.000
194	U	0.585	0.450	0.490	0.508	0.069	0.230	0.200	0.170	0.200	0.030
195	G	0.430	0.640	0.495	0.522	0.108	0.040	0.000	0.000	0.013	0.023
196	U	0.765	0.715	0.630	0.703	0.068	0.570	0.755	0.680	0.668	0.093
197	U	1.000	0.885	0.700	0.862	0.151	0.715	0.865	0.810	0.797	0.076
198	U	0.530	0.560	0.385	0.492	0.094	0.395	0.500	0.505	0.467	0.062
199	G	0.100	0.100	0.060	0.087	0.023	0.125	0.115	0.100	0.113	0.013
200	U	0.125	0.110	0.095	0.110	0.015	0.080	0.100	0.050	0.077	0.025
201	C	0.000	0.000	0.000	0.000	0.000	0.000	0.000	0.000	0.000	0.000
202	U	0.110	0.035	0.130	0.092	0.050	0.125	0.100	0.090	0.105	0.018
203	U	0.275	0.175	0.215	0.222	0.050	0.220	0.210	0.160	0.197	0.032
204	G	0.155	0.230	0.145	0.177	0.046	0.150	0.165	0.135	0.150	0.015
205	U	0.240	0.290	0.170	0.233	0.060	0.180	0.265	0.265	0.237	0.049
206	A	0.310	0.255	0.220	0.262	0.045	0.300	0.315	0.250	0.288	0.034
207	U	0.245	0.230	0.275	0.250	0.023	0.260	0.245	0.195	0.233	0.034
208	U	0.325	0.280	0.335	0.313	0.029	0.330	0.325	0.330	0.328	0.003
209	G	0.875	0.585	0.735	0.732	0.145	0.690	0.640	0.660	0.663	0.025
210	U	0.135	0.105	0.150	0.130	0.023	0.120	0.145	0.140	0.135	0.013
211	C	0.000	0.000	0.000	0.000	0.000	0.000	0.000	0.000	0.000	0.000
212	U	0.000	0.000	0.000	0.000	0.000	0.000	0.000	0.000	0.000	0.000
213	C	0.000	0.000	0.000	0.000	0.000	0.000	0.000	0.000	0.000	0.000
214	U	0.000	0.000	0.000	0.000	0.000	0.000	0.000	0.000	0.000	0.000
215	U	0.000	0.000	0.000	0.000	0.000	0.070	0.060	0.040	0.057	0.015
216	U	0.040	0.055	0.050	0.048	0.008	0.040	0.065	0.125	0.077	0.044
217	C	0.000	0.000	0.000	0.000	0.000	0.030	0.000	0.080	0.037	0.040
218	U	0.150	0.130	0.240	0.173	0.059	0.175	0.055	0.220	0.150	0.085
219	U	0.460	0.445	0.515	0.473	0.037	0.365	0.350	0.430	0.382	0.043
220	G	0.655	0.555	0.710	0.640	0.079	0.580	0.480	0.535	0.532	0.050
221	U	0.135	0.150	0.235	0.173	0.054	0.280	0.120	0.135	0.178	0.088
222	C	0.175	0.165	0.230	0.190	0.035	0.255	0.085	0.125	0.155	0.089
223	U	0.325	0.275	0.385	0.328	0.055	0.400	0.260	0.205	0.288	0.101
224	G	0.370	0.280	0.290	0.313	0.049	0.240	0.240	0.150	0.210	0.052
225	G	0.000	0.000	0.000	0.000	0.000	0.000	0.000	0.000	0.000	0.000
226	C	0.000	0.000	0.000	0.000	0.000	0.025	0.000	0.000	0.008	0.014
227	U	0.440	0.645	0.555	0.547	0.103	0.955	0.665	0.835	0.818	0.146
228	A	0.800	0.740	0.775	0.772	0.030	0.665	0.780	0.710	0.718	0.058
229	U	0.305	0.470	0.415	0.397	0.084	0.400	0.435	0.395	0.410	0.022
230	C	0.000	0.000	0.000	0.000	0.000	0.000	0.000	0.000	0.000	0.000
231	A	0.925	0.940	0.895	0.920	0.023	0.800	0.870	1.015	0.895	0.110
232	U	0.170	0.320	0.275	0.255	0.077	0.315	0.220	0.265	0.267	0.048
233	C	0.000	0.000	0.145	0.048	0.084	0.010	0.000	0.090	0.033	0.049
234	A	0.475	0.560	0.585	0.540	0.058	0.410	0.455	0.665	0.510	0.136
235	C	0.000	0.000	0.000	0.000	0.000	0.000	0.000	0.000	0.000	0.000
236	A	0.820	0.850	0.890	0.853	0.035	0.695	0.860	0.910	0.822	0.113
237	A	0.865	0.970	0.965	0.933	0.059	0.805	0.940	0.795	0.847	0.081
238	G	0.405	0.430	0.335	0.390	0.049	0.290	0.250	0.220	0.253	0.035
239	A	0.095	0.085	0.115	0.098	0.015	0.105	0.075	0.080	0.087	0.016
240	G	0.020	0.040	0.000	0.020	0.020	0.020	0.020	0.020	0.020	0.000

hSHAPE Reactivities from 3 independent experiments (SP102*i*)											
Nucleotides		In the Absence of Pr77^{Gag}					In the Presence of Pr77^{Gag}				
Number	Sequence	Expt 1	Expt 2	Expt 3	Mean	SD	Expt 1	Expt 2	Expt 3	Mean	SD
241	C	0.025	0.030	0.070	0.042	0.025	0.045	0.035	0.060	0.047	0.013
242	G	0.020	0.110	0.000	0.043	0.059	0.060	0.000	0.005	0.022	0.033
243	G	0.000	0.000	0.000	0.000	0.000	0.000	0.000	0.000	0.000	0.000
244	A	0.200	0.190	0.155	0.182	0.024	0.155	0.165	0.195	0.172	0.021
245	A	0.260	0.295	0.260	0.272	0.020	0.230	0.275	0.290	0.265	0.031
246	C	0.090	0.140	0.100	0.110	0.026	0.080	0.140	0.220	0.147	0.070
247	G	0.000	0.000	0.000	0.000	0.000	0.000	0.000	0.000	0.000	0.000
248	G	0.000	0.000	0.000	0.000	0.000	0.000	0.000	0.000	0.000	0.000
249	A	2.120	2.310	2.320	2.250	0.113	2.465	2.545	2.985	2.665	0.280
250	C	0.000	0.000	0.000	0.000	0.000	0.000	0.000	0.130	0.043	0.075
251	U	0.020	0.030	0.040	0.030	0.010	0.000	0.010	0.070	0.027	0.038
252	C	0.050	0.000	0.025	0.025	0.025	0.020	0.060	0.110	0.063	0.045
253	A	0.090	0.035	0.070	0.065	0.028	0.050	0.085	0.075	0.070	0.018
254	C	0.000	0.000	0.000	0.000	0.000	0.000	0.000	0.000	0.000	0.000
255	C	0.000	0.000	0.005	0.002	0.003	0.000	0.000	0.000	0.000	0.000
256	A	0.445	0.570	0.535	0.517	0.064	0.740	0.685	0.540	0.655	0.103
257	U	0.710	0.720	0.955	0.795	0.139	2.055	1.520	1.245	1.607	0.412
258	A	1.005	0.995	1.015	1.005	0.010	1.245	1.380	1.080	1.235	0.150
259	G	0.085	0.090	0.040	0.072	0.028	0.050	0.130	0.030	0.070	0.053
260	G	0.080	0.130	0.000	0.070	0.066	0.090	0.080	0.010	0.060	0.044
261	G	0.080	0.040	0.000	0.040	0.040	0.230	0.120	0.020	0.123	0.105
262	A	0.600	0.600	0.680	0.627	0.046	0.415	0.375	0.405	0.398	0.021
263	G	0.595	0.380	0.555	0.510	0.114	0.375	0.180	0.240	0.265	0.100
264	C	0.540	0.365	0.390	0.432	0.095	0.340	0.180	0.325	0.282	0.088
265	U	2.285	2.100	2.385	2.257	0.145	0.830	1.160	0.955	0.982	0.167
266	G	0.000	0.000	0.000	0.000	0.000	0.000	0.000	0.000	0.000	0.000
267	C	0.000	0.000	0.000	0.000	0.000	0.000	0.000	0.130	0.043	0.075
268	A	2.625	2.310	2.810	2.582	0.253	1.510	1.940	1.340	1.597	0.309
269	G	0.170	0.020	0.000	0.063	0.093	1.520	1.170	0.700	1.130	0.411
270	U	0.110	0.100	0.030	0.080	0.044	0.070	0.160	0.180	0.137	0.059
271	C	0.000	0.060	0.070	0.043	0.038	0.000	0.000	0.000	0.000	0.000
272	C	0.000	0.000	0.000	0.000	0.000	0.000	0.000	0.000	0.000	0.000
273	C	0.000	0.000	0.000	0.000	0.000	0.230	0.000	0.030	0.087	0.125
274	G	0.000	0.000	0.000	0.000	0.000	0.000	0.000	0.070	0.023	0.040
275	C	0.000	0.000	0.000	0.000	0.000	0.170	0.000	0.110	0.093	0.086
276	C	0.010	0.095	0.000	0.035	0.052	0.265	0.070	0.270	0.202	0.114
277	U	0.060	0.160	0.055	0.092	0.059	0.265	0.140	0.335	0.247	0.099
278	A	0.080	0.120	0.070	0.090	0.026	0.165	0.010	0.215	0.130	0.107
279	C	0.060	0.025	0.145	0.077	0.062	0.120	0.170	0.070	0.120	0.050
280	G	0.270	0.270	0.240	0.260	0.017	0.040	0.310	0.160	0.170	0.135
281	G	0.810	0.740	1.115	0.888	0.199	0.950	1.110	1.150	1.070	0.106
282	A	0.955	1.005	0.725	0.895	0.149	1.220	1.650	1.550	1.473	0.225
283	G	1.105	1.160	0.960	1.075	0.103	1.380	1.460	1.280	1.373	0.090
284	A	3.350	3.615	4.125	3.697	0.394	3.670	4.440	3.775	3.962	0.418
285	A	1.580	2.055	2.080	1.905	0.282	1.910	2.965	2.390	2.422	0.528
286	G	1.540	1.570	1.190	1.433	0.211	1.800	1.920	1.590	1.770	0.167
287	A	4.325	4.230	4.760	4.438	0.283	4.090	4.740	4.665	4.498	0.356
288	G	1.375	1.270	1.565	1.403	0.150	1.430	1.610	1.490	1.510	0.092

hSHAPE Reactivities from 3 independent experiments (SP102*i*)											
**Nucleotides**		**In the Absence of Pr77^{Gag}**					**In the Presence of Pr77^{Gag}**				
**Number**	**Sequence**	**Expt 1**	**Expt 2**	**Expt 3**	**Mean**	**SD**	**Expt 1**	**Expt 2**	**Expt 3**	**Mean**	**SD**
289	G	0.000	0.000	0.000	0.000	0.000	0.000	0.000	0.000	0.000	0.000
290	U	0.000	0.000	0.000	0.000	0.000	0.005	0.055	0.020	0.027	0.026
291	A	0.035	0.070	0.000	0.035	0.035	0.060	0.165	0.010	0.078	0.079
292	G	0.000	0.000	0.000	0.000	0.000	0.000	0.000	0.000	0.000	0.000
293	G	0.000	0.000	0.000	0.000	0.000	0.000	0.000	0.000	0.000	0.000
294	U	0.470	0.355	0.335	0.387	0.073	0.660	0.520	0.480	0.553	0.095
295	U	0.980	0.810	1.140	0.977	0.165	0.850	0.785	0.825	0.820	0.033
296	A	2.060	1.850	1.900	1.937	0.110	1.515	1.480	2.055	1.683	0.322
297	C	1.185	1.095	1.325	1.202	0.116	1.460	1.655	1.435	1.517	0.120
298	G	0.000	0.000	0.010	0.003	0.006	0.000	0.000	0.010	0.003	0.006
299	G	0.060	0.110	0.160	0.110	0.050	0.055	0.000	0.000	0.018	0.032
300	U	0.060	0.000	0.040	0.033	0.031	0.000	0.000	0.000	0.000	0.000
301	G	0.000	0.000	0.000	0.000	0.000	0.000	0.000	0.000	0.000	0.000
302	A	0.000	0.000	0.000	0.000	0.000	0.000	0.000	0.000	0.000	0.000
303	G	0.000	0.000	0.000	0.000	0.000	0.000	0.000	0.000	0.000	0.000
304	C	0.000	0.000	0.000	0.000	0.000	0.000	0.000	0.065	0.022	0.038
305	C	0.000	0.000	0.000	0.000	0.000	0.000	0.000	0.000	0.000	0.000
306	A	0.780	0.630	1.070	0.827	0.224	0.460	0.495	0.615	0.523	0.081
307	U	0.810	0.790	1.120	0.907	0.185	0.910	0.835	0.930	0.892	0.050
308	U	2.140	1.690	2.040	1.957	0.236	1.705	1.920	1.810	1.812	0.108
309	G	1.250	1.720	1.350	1.440	0.248	0.560	0.680	0.190	0.477	0.255
310	G	0.610	1.130	1.170	0.970	0.312	0.000	0.000	0.000	0.000	0.000
311	A	1.760	1.070	1.630	1.487	0.367	1.680	1.740	1.780	1.733	0.050
312	A	0.700	0.560	0.700	0.653	0.081	0.680	0.630	0.650	0.653	0.025
313	A	0.230	0.170	0.230	0.210	0.035	0.190	0.130	0.200	0.173	0.038
314	U	0.140	0.290	0.250	0.227	0.078	0.170	0.160	0.190	0.173	0.015
315	G	0.000	0.280	0.000	0.093	0.162	0.010	0.060	0.030	0.033	0.025
316	G	0.000	0.000	0.000	0.000	0.000	0.000	0.100	0.090	0.063	0.055
317	G	0.000	0.000	0.000	0.000	0.000	0.000	0.000	0.000	0.000	0.000
318	G	0.000	0.000	0.000	0.000	0.000	0.570	0.420	0.940	0.643	0.268
319	G	0.000	0.000	0.000	0.000	0.000	0.000	0.000	0.000	0.000	0.000
320	U	0.290	0.000	0.180	0.157	0.146	0.600	0.570	0.540	0.570	0.030
321	C	0.000	0.000	0.000	0.000	0.000	0.000	0.000	0.000	0.000	0.000
322	U	0.000	0.000	0.000	0.000	0.000	0.000	0.010	0.030	0.013	0.015
323	C	0.000	0.000	0.000	0.000	0.000	0.000	0.000	0.000	0.000	0.000
324	G → U	0.000	0.030	0.000	0.010	0.017	0.000	0.000	0.000	0.000	0.000
325	G	0.000	0.000	0.000	0.000	0.000	0.000	0.000	0.000	0.000	0.000
326	G → U	0.000	0.230	0.000	0.077	0.133	0.000	0.020	0.010	0.010	0.010
327	C	0.000	0.000	0.000	0.000	0.000	0.000	0.050	0.150	0.067	0.076
328	U	0.590	0.480	0.630	0.567	0.078	0.580	0.710	0.810	0.700	0.115
329	C	0.050	0.000	0.120	0.057	0.060	0.490	0.800	1.000	0.763	0.257
330	A	2.440	2.270	2.660	2.457	0.196	2.050	2.510	2.710	2.423	0.338
331	A	2.350	2.220	2.580	2.383	0.182	1.920	2.370	2.440	2.243	0.282
332	A	2.930	2.810	3.350	3.030	0.284	2.330	3.000	3.100	2.810	0.419
333	A	1.910	1.660	2.140	1.903	0.240	1.420	1.630	1.940	1.663	0.262
334	G	0.470	0.390	0.620	0.493	0.117	0.240	0.080	0.220	0.180	0.087
335	G	0.070	0.000	0.120	0.063	0.060	0.000	0.000	0.000	0.000	0.000
336	G	0.000	0.000	0.000	0.000	0.000	0.000	0.000	0.000	0.000	0.000

hSHAPE Reactivities from 3 independent experiments (SP102*i*)											
Nucleotides		In the Absence of Pr77^{Gag}					In the Presence of Pr77^{Gag}				
**Number**	**Sequence**	**Expt 1**	**Expt 2**	**Expt 3**	**Mean**	**SD**	**Expt 1**	**Expt 2**	**Expt 3**	**Mean**	**SD**
337	C	0.000	0.000	0.000	0.000	0.000	0.000	0.000	0.000	0.000	0.000
338	A	0.150	0.030	0.060	0.080	0.062	0.000	0.110	0.000	0.037	0.064
339	G	0.000	0.000	0.000	0.000	0.000	0.040	0.000	0.000	0.013	0.023
340	A	0.300	0.230	0.320	0.283	0.047	0.200	0.420	0.170	0.263	0.137
341	A	0.890	0.790	0.910	0.863	0.064	0.640	0.800	0.710	0.717	0.080
342	A	0.280	0.290	0.250	0.273	0.021	0.140	0.260	0.180	0.193	0.061
343	C	0.000	0.000	0.000	0.000	0.000	0.000	0.000	0.000	0.000	0.000
344	U	0.000	0.000	0.080	0.027	0.046	0.060	0.030	0.080	0.057	0.025
345	C	0.000	0.000	0.090	0.030	0.052	0.120	0.000	0.160	0.093	0.083
346	U	0.260	0.200	0.290	0.250	0.046	0.200	0.130	0.300	0.210	0.085
347	U	0.630	0.520	0.730	0.627	0.105	0.610	0.560	0.730	0.633	0.087
348	U	0.730	0.600	0.820	0.717	0.111	0.680	0.700	0.830	0.737	0.081
349	G	0.320	0.350	0.380	0.350	0.030	0.350	0.220	0.300	0.290	0.066
350	U	1.120	1.000	1.210	1.110	0.105	1.070	1.130	1.220	1.140	0.075
351	U	0.490	0.450	0.550	0.497	0.050	0.470	0.510	0.520	0.500	0.026
352	U	0.050	0.090	0.080	0.073	0.021	0.050	0.070	0.060	0.060	0.010
353	C	0.000	0.000	0.000	0.000	0.000	0.000	0.000	0.000	0.000	0.000
354	U	1.250	0.930	1.280	1.153	0.194	1.000	1.070	1.140	1.070	0.070
355	G	2.290	1.800	2.140	2.077	0.251	1.960	1.950	1.960	1.957	0.006
356	U	0.660	0.530	0.630	0.607	0.068	0.510	0.620	0.610	0.580	0.061
357	U	0.560	0.400	0.510	0.490	0.082	0.430	0.500	0.480	0.470	0.036
358	U	0.440	0.350	0.490	0.427	0.071	0.460	0.550	0.530	0.513	0.047
359	U	0.370	0.360	0.400	0.377	0.021	0.500	0.520	0.530	0.517	0.015
360	A	0.790	0.470	0.800	0.687	0.188	0.760	0.820	0.850	0.810	0.046
361	C	0.000	0.000	0.580	0.193	0.335	0.180	0.000	0.140	0.107	0.095
362	A	0.460	0.400	0.500	0.453	0.050	0.420	0.390	0.440	0.417	0.025
363	A	0.190	0.220	0.190	0.200	0.017	0.180	0.140	0.160	0.160	0.020
364	A	0.010	0.060	0.040	0.037	0.025	0.040	0.050	0.040	0.043	0.006
365	G	0.000	0.020	0.000	0.007	0.012	0.000	0.000	0.000	0.000	0.000
366	G	0.000	0.080	0.030	0.037	0.040	0.000	0.080	0.080	0.053	0.046
367	C	0.000	0.000	0.000	0.000	0.000	0.000	0.000	0.000	0.000	0.000
368	U	0.000	0.000	0.000	0.000	0.000	0.000	0.000	0.160	0.053	0.092
369	C	0.000	0.000	0.000	0.000	0.000	0.000	0.000	0.000	0.000	0.000
370	C	0.000	0.000	0.000	0.000	0.000	0.060	0.000	0.050	0.037	0.032
371	U	0.000	0.000	0.000	0.000	0.000	0.000	0.000	0.040	0.013	0.023
372	C	0.040	0.030	0.050	0.040	0.010	0.040	0.020	0.000	0.020	0.020
373	U	0.410	0.350	0.530	0.430	0.092	0.620	0.560	0.370	0.517	0.131
374	C	1.960	1.860	2.310	2.043	0.236	2.480	2.800	2.320	2.533	0.244
375	A	3.030	2.660	3.120	2.937	0.244	3.320	2.900	2.390	2.870	0.466
376	G	2.400	2.290	1.890	2.193	0.268	2.780	2.290	2.270	2.447	0.289
377	A	2.320	2.210	2.640	2.390	0.223	2.450	2.750	2.770	2.657	0.179
378	G	0.040	0.050	0.020	0.037	0.015	0.070	0.080	0.050	0.067	0.015
379	A	0.000	0.000	0.030	0.010	0.017	0.000	0.000	0.000	0.000	0.000
380	G	0.000	0.030	0.000	0.010	0.017	0.000	0.000	0.000	0.000	0.000
381	G	0.000	0.000	0.000	0.000	0.000	0.000	0.000	0.000	0.000	0.000
382	G	0.000	4.460	2.780	2.413	2.252	0.000	0.000	0.000	0.000	0.000
383	G	0.000	0.000	0.000	0.000	0.000	0.000	0.000	0.000	0.000	0.000
384	U	0.000	0.000	0.000	0.000	0.000	0.170	0.310	0.480	0.320	0.155

hSHAPE Reactivities from 3 independent experiments (SP102*i*)											
Nucleotides		In the Absence of Pr77^{Gag}					In the Presence of Pr77^{Gag}				
**Number**	**Sequence**	**Expt 1**	**Expt 2**	**Expt 3**	**Mean**	**SD**	**Expt 1**	**Expt 2**	**Expt 3**	**Mean**	**SD**
385	C	0.000	0.000	0.220	0.073	0.127	0.200	0.030	0.100	0.110	0.085
386	U	0.140	0.060	0.480	0.227	0.223	0.160	0.000	0.000	0.053	0.092
387	U	0.380	0.000	0.720	0.367	0.360	0.780	0.750	0.750	0.760	0.017
388	C	0.000	0.000	1.240	0.413	0.716	1.260	0.800	1.790	1.283	0.495
389	A	1.200	0.980	1.060	1.080	0.111	1.160	1.150	1.150	1.153	0.006
390	U	0.000	0.010	0.000	0.003	0.006	0.000	0.000	0.000	0.000	0.000
391	G	0.050	0.020	0.000	0.023	0.025	0.030	0.000	0.010	0.013	0.015
392	U	0.040	0.020	0.070	0.043	0.025	0.080	0.040	0.050	0.057	0.021
393	G	0.000	0.000	0.040	0.013	0.023	0.000	0.000	0.000	0.000	0.000
394	A	0.160	0.000	0.310	0.157	0.155	0.190	0.220	0.230	0.213	0.021
395	A	0.460	0.290	0.540	0.430	0.128	0.510	0.670	0.500	0.560	0.095
396	A	0.960	0.610	0.960	0.843	0.202	0.940	1.070	0.970	0.993	0.068
397	G	0.480	0.390	0.660	0.510	0.137	0.560	0.580	0.510	0.550	0.036
398	A	0.450	0.520	0.560	0.510	0.056	0.490	0.530	0.500	0.507	0.021
399	G	0.470	0.440	0.510	0.473	0.035	0.410	0.390	0.520	0.440	0.070
400	A	0.630	0.720	0.580	0.643	0.071	0.410	0.480	0.730	0.540	0.168
401	G	0.350	0.280	0.370	0.333	0.047	0.160	0.380	0.100	0.213	0.147
402	U	0.220	0.310	0.480	0.337	0.132	0.690	0.570	0.750	0.670	0.092
403	A	0.980	1.050	1.020	1.017	0.035	0.000	0.000	0.000	0.000	0.000
404	G	0.410	0.430	0.250	0.363	0.099	0.000	0.190	0.000	0.063	0.110
405	U	0.410	0.120	0.120	0.217	0.167	0.010	0.120	0.000	0.043	0.067
406	G	0.570	0.580	0.210	0.453	0.211	0.390	0.050	0.070	0.170	0.191
407	C	0.240	0.000	0.110	0.117	0.120	0.900	0.110	0.320	0.443	0.409
408	A	0.330	0.470	0.410	0.403	0.070	0.550	0.330	0.170	0.350	0.191
409	A	0.390	0.380	0.480	0.417	0.055	0.570	0.570	0.550	0.563	0.012
410	U	0.410	0.500	0.220	0.377	0.143	0.570	0.530	0.550	0.550	0.020
411	A	0.490	0.310	0.650	0.483	0.170	0.400	0.440	0.850	0.563	0.249
412	G	0.400	0.330	0.240	0.323	0.080	0.290	0.002	0.000	0.097	0.167
413	A	0.400	0.440	0.570	0.470	0.089	0.470	0.570	0.590	0.543	0.064
414	A	0.400	0.370	0.490	0.420	0.062	0.420	0.530	0.490	0.480	0.056
415	U	0.440	0.330	0.440	0.403	0.064	0.340	0.540	0.540	0.473	0.115
416	U	0.460	0.000	0.000	0.153	0.266	0.000	0.150	0.000	0.050	0.087
417	U	0.360	0.570	0.790	0.573	0.215	0.810	0.570	1.160	0.847	0.297
418	U	0.570	0.580	0.570	0.573	0.006	0.530	0.730	0.780	0.680	0.132
419	A	0.020	0.180	0.350	0.183	0.165	0.560	0.730	0.590	0.627	0.091
420	U	0.180	0.250	0.270	0.233	0.047	0.140	0.130	0.230	0.167	0.055
421	C	0.000	0.000	0.550	0.183	0.318	0.460	0.000	0.000	0.153	0.266
422	A	0.360	0.280	0.360	0.333	0.046	0.000	0.000	0.000	0.000	0.000
423	G	0.280	0.140	0.090	0.170	0.098	0.340	0.360	0.290	0.330	0.036
424	U	0.040	0.130	0.090	0.087	0.045	0.150	0.210	0.200	0.187	0.032
425	U	0.000	0.000	0.000	0.000	0.000	0.000	0.000	0.000	0.000	0.000
426	U	-999	-999	-999	-999	0.000	-999	-999	-999	-999	0.000
427	C	-999	-999	-999	-999	0.000	-999	-999	-999	-999	0.000
428	U	-999	-999	-999	-999	0.000	-999	-999	-999	-999	0.000
429	A	-999	-999	-999	-999	0.000	-999	-999	-999	-999	0.000
430	A	-999	-999	-999	-999	0.000	-999	-999	-999	-999	0.000
431	U	-999	-999	-999	-999	0.000	-999	-999	-999	-999	0.000
432	A	-999	-999	-999	-999	0.000	-999	-999	-999	-999	0.000

hSHAPE Reactivities from 3 independent experiments (SP105*i*)											
Nucleotides		In the Absence of Pr77^{Gag}					In the Presence of Pr77^{Gag}				
Number	Sequence	Expt 1	Expt 2	Expt 3	Mean	SD	Expt 1	Expt 2	Expt 3	Mean	SD
1	G	-999	-999	-999	-999	0.000	-999	-999	-999	-999	0.000
2	C	-999	-999	-999	-999	0.000	-999	-999	-999	-999	0.000
3	A	-999	-999	-999	-999	0.000	-999	-999	-999	-999	0.000
4	A	-999	-999	-999	-999	0.000	-999	-999	-999	-999	0.000
5	C	-999	-999	-999	-999	0.000	-999	-999	-999	-999	0.000
6	A	-999	-999	-999	-999	0.000	-999	-999	-999	-999	0.000
7	G	-999	-999	-999	-999	0.000	-999	-999	-999	-999	0.000
8	U	-999	-999	-999	-999	0.000	-999	-999	-999	-999	0.000
9	C	-999	-999	-999	-999	0.000	-999	-999	-999	-999	0.000
10	C	-999	-999	-999	-999	0.000	-999	-999	-999	-999	0.000
11	U	-999	-999	-999	-999	0.000	-999	-999	-999	-999	0.000
12	A	-999	-999	-999	-999	0.000	-999	-999	-999	-999	0.000
13	A	-999	-999	-999	-999	0.000	-999	-999	-999	-999	0.000
14	U	-999	-999	-999	-999	0.000	-999	-999	-999	-999	0.000
15	A	-999	-999	-999	-999	0.000	1.230	1.335	1.250	1.272	0.056
16	U	-999	-999	-999	-999	0.000	1.265	1.510	1.595	1.457	0.171
17	U	-999	-999	-999	-999	0.000	0.555	0.520	0.740	0.605	0.118
18	C	-999	-999	-999	-999	0.000	0.100	0.000	0.210	0.103	0.105
19	A	0.235	0.160	0.120	0.172	0.058	0.100	0.000	0.330	0.143	0.169
20	C	0.115	0.095	0.165	0.125	0.036	0.000	0.090	0.140	0.077	0.071
21	G	0.530	0.530	0.465	0.508	0.038	0.215	0.545	0.420	0.393	0.167
22	U	2.275	1.890	2.145	2.103	0.196	1.615	1.730	1.600	1.648	0.071
23	C	0.380	0.450	0.560	0.463	0.091	0.410	0.455	0.485	0.450	0.038
24	U	2.915	2.775	3.090	2.927	0.158	2.470	2.545	2.725	2.580	0.131
25	C	0.730	0.740	0.925	0.798	0.110	0.650	0.615	0.745	0.670	0.067
26	G	0.215	0.220	0.250	0.228	0.019	0.020	0.220	0.125	0.122	0.100
27	U	0.060	0.105	0.145	0.103	0.043	0.090	0.035	0.020	0.048	0.037
28	G	0.000	0.000	0.000	0.000	0.000	0.000	0.000	0.000	0.000	0.000
29	U	0.215	0.305	0.395	0.305	0.090	0.290	0.215	0.245	0.250	0.038
30	G	0.270	0.275	0.320	0.288	0.028	0.140	0.195	0.180	0.172	0.028
31	U	0.085	0.105	0.160	0.117	0.039	0.120	0.100	0.125	0.115	0.013
32	U	0.445	0.515	0.415	0.458	0.051	0.320	0.370	0.360	0.350	0.026
33	U	0.420	0.410	0.385	0.405	0.018	0.315	0.255	0.255	0.275	0.035
34	G	0.285	0.295	0.330	0.303	0.024	0.195	0.235	0.300	0.243	0.053
35	U	1.825	1.695	1.640	1.720	0.095	1.210	1.660	1.470	1.447	0.226
36	G	0.430	0.360	0.370	0.387	0.038	0.280	0.325	0.410	0.338	0.066
37	U	0.090	0.070	0.025	0.062	0.033	0.050	0.050	0.080	0.060	0.017
38	C	0.000	0.055	0.020	0.025	0.028	0.000	0.050	0.090	0.047	0.045
39	U	0.030	0.105	0.035	0.057	0.042	0.000	0.100	0.000	0.033	0.058
40	G	0.000	0.000	0.000	0.000	0.000	0.000	0.045	0.000	0.015	0.026
41	U	0.000	0.000	0.000	0.000	0.000	0.055	0.095	0.000	0.050	0.048
42	U	0.140	0.190	0.455	0.262	0.169	0.190	0.415	0.390	0.332	0.123
43	C	0.480	0.465	0.565	0.503	0.054	0.410	0.460	0.630	0.500	0.115
44	G	0.000	0.290	0.450	0.247	0.228	0.255	0.000	0.640	0.298	0.322
45	C	0.000	0.000	0.000	0.000	0.000	0.000	0.000	0.000	0.000	0.000
46	C	0.000	0.000	0.000	0.000	0.000	0.000	0.000	0.000	0.000	0.000
47	A	0.805	0.890	0.790	0.828	0.054	0.405	0.380	0.575	0.453	0.106
48	U	1.090	1.080	1.145	1.105	0.035	0.690	0.740	1.030	0.820	0.184

hSHAPE Reactivities from 3 independent experiments (SP105*i*)											
Nucleotides		In the Absence of Pr77^{Gag}					In the Presence of Pr77^{Gag}				
Number	Sequence	Expt 1	Expt 2	Expt 3	Mean	SD	Expt 1	Expt 2	Expt 3	Mean	SD
49	C	0.530	0.515	0.565	0.537	0.026	0.285	0.180	0.485	0.317	0.155
50	C → G	0.280	0.325	0.490	0.365	0.111	0.240	0.140	0.320	0.233	0.090
51	C	0.000	0.000	0.040	0.013	0.023	0.000	0.080	0.070	0.050	0.044
52	G → U	0.710	0.635	0.910	0.752	0.142	0.295	0.590	0.505	0.463	0.152
53	U	0.735	0.690	0.885	0.770	0.102	0.530	0.500	0.610	0.547	0.057
54	C	0.310	0.245	0.440	0.332	0.099	0.145	0.115	0.300	0.187	0.099
55	U	0.110	0.090	0.205	0.135	0.061	0.030	0.000	0.055	0.028	0.028
56	C	0.000	0.000	0.000	0.000	0.000	0.000	0.000	0.000	0.000	0.000
57	C	0.000	0.000	0.000	0.000	0.000	0.000	0.000	0.000	0.000	0.000
58	G	0.000	0.000	0.000	0.000	0.000	0.000	0.000	0.000	0.000	0.000
59	C	0.000	0.000	0.000	0.000	0.000	0.000	0.000	0.000	0.000	0.000
60	U	0.005	0.040	0.030	0.025	0.018	0.000	0.000	0.000	0.000	0.000
61	C	0.000	0.000	0.025	0.008	0.014	0.000	0.000	0.000	0.000	0.000
62	G	0.000	0.000	0.000	0.000	0.000	0.000	0.000	0.000	0.000	0.000
63	U	0.000	0.000	0.000	0.000	0.000	0.000	0.000	0.000	0.000	0.000
64	C	0.000	0.000	0.000	0.000	0.000	0.000	0.000	0.000	0.000	0.000
65	A	0.075	0.165	0.015	0.085	0.075	0.000	0.000	0.000	0.000	0.000
66	C	0.000	0.000	0.000	0.000	0.000	0.000	0.080	0.000	0.027	0.046
67	U	0.340	0.315	0.320	0.325	0.013	0.120	0.000	0.235	0.118	0.118
68	U	0.255	0.320	0.260	0.278	0.036	0.130	0.000	0.175	0.102	0.091
69	A	0.660	0.560	0.540	0.587	0.064	0.275	0.310	0.340	0.308	0.033
70	U	0.345	0.330	0.305	0.327	0.020	0.100	0.100	0.150	0.117	0.029
71	C	0.000	0.000	0.000	0.000	0.000	0.000	0.000	0.060	0.020	0.035
72	C	0.000	0.000	0.000	0.000	0.000	0.000	0.000	0.000	0.000	0.000
73	U	0.650	0.895	0.790	0.778	0.123	0.665	0.665	0.400	0.577	0.153
74	U	1.360	1.320	1.215	1.298	0.075	1.000	0.980	1.120	1.033	0.076
75	C	0.000	0.000	0.000	0.000	0.000	0.000	0.000	0.000	0.000	0.000
76	A	0.845	0.680	0.470	0.665	0.188	0.730	0.000	0.960	0.563	0.501
77	C	0.145	0.115	0.175	0.145	0.030	0.000	0.000	0.000	0.000	0.000
78	U	1.345	1.155	1.105	1.202	0.127	0.710	0.825	0.725	0.753	0.063
79	U	1.830	1.590	1.595	1.672	0.137	1.085	1.215	1.170	1.157	0.066
80	U	1.050	0.770	0.960	0.927	0.143	0.450	0.550	0.915	0.638	0.245
81	C	0.000	0.000	0.000	0.000	0.000	0.345	0.000	0.305	0.217	0.189
82	C	0.000	0.000	0.000	0.000	0.000	0.100	0.000	0.060	0.053	0.050
83	A	0.975	0.510	1.005	0.830	0.278	1.015	1.160	1.115	1.097	0.074
84	G	1.590	1.695	1.680	1.655	0.057	1.370	0.125	1.290	0.928	0.697
85	A	0.095	0.110	0.110	0.105	0.009	0.000	0.010	0.140	0.050	0.078
86	G	0.000	0.000	0.000	0.000	0.000	0.000	0.000	0.000	0.000	0.000
87	G	0.000	0.000	0.000	0.000	0.000	0.000	0.000	0.000	0.000	0.000
88	G	0.000	0.000	0.000	0.000	0.000	0.000	0.000	0.000	0.000	0.000
89	U	0.000	0.000	0.000	0.000	0.000	0.000	0.000	0.000	0.000	0.000
90	C	0.000	0.000	0.000	0.000	0.000	0.000	0.000	0.000	0.000	0.000
91	C	0.000	0.000	0.000	0.000	0.000	0.000	0.000	0.000	0.000	0.000
92	C	0.000	0.000	0.000	0.000	0.000	0.000	0.000	0.000	0.000	0.000
93	C	0.000	0.000	0.000	0.000	0.000	0.000	0.000	0.000	0.000	0.000
94	C	0.000	0.000	0.000	0.000	0.000	0.000	0.000	0.000	0.000	0.000
95	C	0.000	0.000	0.000	0.000	0.000	0.000	0.000	0.000	0.000	0.000
96	G	0.110	0.010	0.100	0.073	0.055	0.020	0.050	0.170	0.080	0.079

hSHAPE Reactivities from 3 independent experiments (SP105*i*)											
Nucleotides		In the Absence of Pr77^{Gag}					In the Presence of Pr77^{Gag}				
Number	Sequence	Expt 1	Expt 2	Expt 3	Mean	SD	Expt 1	Expt 2	Expt 3	Mean	SD
97	C	0.000	0.000	0.000	0.000	0.000	0.000	0.000	0.000	0.000	0.000
98	A	0.415	0.300	0.465	0.393	0.085	0.150	0.000	0.255	0.135	0.128
99	G	0.770	0.600	0.710	0.693	0.086	0.355	0.315	0.390	0.353	0.038
100	A	1.855	1.610	1.950	1.805	0.175	1.325	1.360	1.400	1.362	0.038
101	C	0.205	0.135	0.300	0.213	0.083	0.135	0.105	0.185	0.142	0.040
102	C	0.035	0.035	0.040	0.037	0.003	0.000	0.000	0.000	0.000	0.000
103	C	0.000	0.000	0.000	0.000	0.000	0.000	0.000	0.000	0.000	0.000
104	C	0.000	0.000	0.000	0.000	0.000	0.000	0.000	0.000	0.000	0.000
105	G	0.000	0.000	0.000	0.000	0.000	0.000	0.000	0.035	0.012	0.020
106	G	0.000	0.000	0.000	0.000	0.000	0.000	0.000	0.000	0.000	0.000
107	U	0.060	0.040	0.075	0.058	0.018	0.000	0.000	0.000	0.000	0.000
108	G	0.000	0.000	0.005	0.002	0.003	0.000	0.000	0.000	0.000	0.000
109	A	0.035	0.040	0.045	0.040	0.005	0.000	0.015	0.000	0.005	0.009
110	C	0.000	0.000	0.000	0.000	0.000	0.000	0.000	0.000	0.000	0.000
111	C	0.000	0.000	0.000	0.000	0.000	0.000	0.000	0.000	0.000	0.000
112	C	0.000	0.000	0.000	0.000	0.000	0.000	0.000	0.000	0.000	0.000
113	U	1.555	1.560	1.620	1.578	0.036	1.140	1.610	1.495	1.415	0.245
114	C	0.375	0.255	0.395	0.342	0.076	0.360	0.125	0.805	0.430	0.345
115	A	2.975	2.115	2.735	2.608	0.444	2.320	2.540	2.800	2.553	0.240
116	G	0.180	0.330	0.215	0.242	0.078	0.125	0.280	0.225	0.210	0.079
117	G	0.020	0.060	0.060	0.047	0.023	0.000	0.000	0.000	0.000	0.000
118	U	0.000	0.000	0.000	0.000	0.000	0.040	0.090	0.020	0.050	0.036
119	C	0.165	0.120	0.150	0.145	0.023	0.010	0.105	0.190	0.102	0.090
120	G	0.150	0.085	0.360	0.198	0.144	0.150	0.270	0.090	0.170	0.092
121	G	0.450	0.505	0.210	0.388	0.157	0.370	0.505	0.520	0.465	0.083
122	C	0.000	0.000	0.000	0.000	0.000	0.000	0.000	0.000	0.000	0.000
123	C	0.000	0.000	0.000	0.000	0.000	0.000	0.000	0.000	0.000	0.000
124	G	0.000	0.000	0.000	0.000	0.000	0.000	0.040	0.000	0.013	0.023
125	A	0.335	0.360	0.380	0.358	0.023	0.160	0.185	0.155	0.167	0.016
126	C	0.000	0.000	0.000	0.000	0.000	0.000	0.090	0.000	0.030	0.052
127	U	0.115	0.120	0.075	0.103	0.025	0.015	0.145	0.075	0.078	0.065
128	G	0.265	0.245	0.295	0.268	0.025	0.120	0.050	0.075	0.082	0.035
129	C	0.000	0.000	0.000	0.000	0.000	0.000	0.000	0.000	0.000	0.000
130	G	0.000	0.000	0.000	0.000	0.000	0.000	0.000	0.000	0.000	0.000
131	G	0.000	0.000	0.000	0.000	0.000	0.000	0.000	0.000	0.000	0.000
132	C	0.000	0.000	0.000	0.000	0.000	0.000	0.000	0.240	0.080	0.139
133	A	0.000	0.000	0.000	0.000	0.000	0.000	0.000	0.000	0.000	0.000
134	G	0.510	0.320	0.940	0.590	0.318	0.220	0.230	0.525	0.325	0.173
135	C	0.460	0.275	0.430	0.388	0.099	0.360	0.030	0.410	0.267	0.206
136	U	0.680	0.515	0.625	0.607	0.084	0.365	0.355	0.640	0.453	0.162
137	G	0.300	0.290	0.340	0.310	0.026	0.055	0.090	0.195	0.113	0.073
138	G	0.000	0.000	0.000	0.000	0.000	0.000	0.000	0.000	0.000	0.000
139	C	0.000	0.000	0.010	0.003	0.006	0.000	0.000	0.000	0.000	0.000
140	G	0.000	0.090	0.150	0.080	0.075	0.000	0.000	0.000	0.000	0.000
141	C	0.000	0.000	0.000	0.000	0.000	0.000	0.000	0.000	0.000	0.000
142	C	0.000	0.000	0.000	0.000	0.000	0.000	0.000	0.000	0.000	0.000
143	C	0.000	0.000	0.000	0.000	0.000	0.000	0.000	0.000	0.000	0.000
144	G	0.305	0.450	0.560	0.438	0.128	0.215	0.200	0.135	0.183	0.043

hSHAPE Reactivities from 3 independent experiments (SP105*i*)											
Nucleotides		In the Absence of Pr77^{Gag}					In the Presence of Pr77^{Gag}				
Number	Sequence	Expt 1	Expt 2	Expt 3	Mean	SD	Expt 1	Expt 2	Expt 3	Mean	SD
145	A	1.045	0.920	0.820	0.928	0.113	1.045	0.880	0.965	0.963	0.083
146	A	0.800	0.805	1.140	0.915	0.195	1.315	1.360	1.095	1.257	0.142
147	C	0.000	0.000	0.000	0.000	0.000	0.770	0.000	0.580	0.450	0.401
148	A	0.655	0.640	0.755	0.683	0.063	0.655	0.000	0.510	0.388	0.344
149	G	0.000	0.015	0.000	0.005	0.009	0.000	0.000	0.000	0.000	0.000
150	G	0.000	0.000	0.000	0.000	0.000	0.000	0.025	0.000	0.008	0.014
151	G	0.000	0.000	0.000	0.000	0.000	0.000	0.000	0.000	0.000	0.000
152	A	0.000	0.000	0.000	0.000	0.000	0.000	0.570	0.000	0.190	0.329
153	C	0.000	0.000	0.000	0.000	0.000	0.000	0.000	0.000	0.000	0.000
154	C	0.000	0.000	0.000	0.000	0.000	0.000	0.000	0.000	0.000	0.000
155	C	0.000	0.000	0.000	0.000	0.000	0.000	0.000	0.000	0.000	0.000
156	U	0.000	0.000	0.000	0.000	0.000	0.000	0.000	0.030	0.010	0.017
157	C	0.245	0.195	0.285	0.242	0.045	0.090	0.225	0.180	0.165	0.069
158	G	0.000	0.000	0.000	0.000	0.000	0.000	0.000	0.000	0.000	0.000
159	G	0.000	0.000	0.000	0.000	0.000	0.000	0.000	0.000	0.000	0.000
160	A	0.000	0.000	0.000	0.000	0.000	0.000	0.000	0.000	0.000	0.000
161	U	0.070	0.110	0.100	0.093	0.021	0.025	0.000	0.000	0.008	0.014
162	A	0.035	0.025	0.030	0.030	0.005	0.000	0.060	0.000	0.020	0.035
163	A	0.045	0.025	0.075	0.048	0.025	0.030	0.080	0.000	0.037	0.040
164	G	0.000	0.000	0.000	0.000	0.000	0.000	0.000	0.000	0.000	0.000
165	U	0.000	0.000	0.000	0.000	0.000	0.000	0.000	0.000	0.000	0.000
166	G	0.000	0.000	0.000	0.000	0.000	0.000	0.000	0.000	0.000	0.000
167	A	0.000	0.000	0.000	0.000	0.000	0.000	0.000	0.000	0.000	0.000
168	C	0.000	0.000	0.000	0.000	0.000	0.000	0.000	0.000	0.000	0.000
169	C	0.000	0.000	0.000	0.000	0.000	0.000	0.000	0.000	0.000	0.000
170	C	0.000	0.000	0.000	0.000	0.000	0.000	0.000	0.000	0.000	0.000
171	U	0.100	0.195	0.030	0.108	0.083	0.000	0.000	0.000	0.000	0.000
172	U	0.495	0.585	0.580	0.553	0.051	0.230	0.045	0.225	0.167	0.105
173	G	0.860	0.795	0.990	0.882	0.099	0.385	0.410	0.305	0.367	0.055
174	U	0.400	0.425	0.485	0.437	0.044	0.235	0.235	0.255	0.242	0.012
175	C	0.000	0.000	0.000	0.000	0.000	0.000	0.000	0.000	0.000	0.000
176	U	0.000	0.000	0.000	0.000	0.000	0.000	0.000	0.000	0.000	0.000
177	C	0.000	0.000	0.000	0.000	0.000	0.000	0.000	0.000	0.000	0.000
178	U	0.000	0.000	0.000	0.000	0.000	0.000	0.000	0.000	0.000	0.000
179	A	0.255	0.215	0.480	0.317	0.143	0.000	0.000	0.000	0.000	0.000
180	U	0.060	0.060	0.100	0.073	0.023	0.000	0.050	0.000	0.017	0.029
181	U	0.140	0.140	0.205	0.162	0.038	0.020	0.160	0.050	0.077	0.074
182	U	0.090	0.090	0.120	0.100	0.017	0.080	0.040	0.050	0.057	0.021
183	C	0.000	0.000	0.000	0.000	0.000	0.000	0.000	0.000	0.000	0.000
184	U	0.000	0.000	0.000	0.000	0.000	0.000	0.000	0.045	0.015	0.026
185	A	0.175	0.175	0.135	0.162	0.023	0.000	0.005	0.000	0.002	0.003
186	C	0.000	0.000	0.000	0.000	0.000	0.000	0.010	0.000	0.003	0.006
187	U	0.000	0.000	0.000	0.000	0.000	0.000	0.000	0.000	0.000	0.000
188	A	0.780	0.725	0.700	0.735	0.041	0.340	0.230	0.435	0.335	0.103
189	U	0.165	0.135	0.120	0.140	0.023	0.170	0.160	0.110	0.147	0.032
190	U	0.125	0.100	0.035	0.087	0.046	0.075	0.000	0.110	0.062	0.056
191	U	0.220	0.185	0.140	0.182	0.040	0.150	0.135	0.135	0.140	0.009
192	G	0.055	0.040	0.135	0.077	0.051	0.030	0.125	0.050	0.068	0.050

hSHAPE Reactivities from 3 independent experiments (SP105*i*)											
Nucleotides		In the Absence of Pr77^{Gag}					In the Presence of Pr77^{Gag}				
Number	Sequence	Expt 1	Expt 2	Expt 3	Mean	SD	Expt 1	Expt 2	Expt 3	Mean	SD
193	G	0.130	0.110	0.245	0.162	0.073	0.000	0.385	0.000	0.128	0.222
194	U	0.295	0.320	0.410	0.342	0.060	0.135	0.620	0.120	0.292	0.284
195	G	0.635	0.620	0.650	0.635	0.015	0.090	0.230	0.115	0.145	0.075
196	U	0.420	0.450	0.455	0.442	0.019	0.260	0.390	0.270	0.307	0.072
197	U	0.710	0.630	0.570	0.637	0.070	0.485	0.585	0.460	0.510	0.066
198	U	0.395	0.410	0.320	0.375	0.048	0.290	0.345	0.295	0.310	0.030
199	G	0.100	0.130	0.070	0.100	0.030	0.000	0.085	0.000	0.028	0.049
200	U	0.055	0.060	0.100	0.072	0.025	0.060	0.045	0.040	0.048	0.010
201	C	0.000	0.000	0.000	0.000	0.000	0.000	0.005	0.000	0.002	0.003
202	U	0.115	0.175	0.090	0.127	0.044	0.070	0.000	0.030	0.033	0.035
203	U	0.245	0.290	0.225	0.253	0.033	0.135	0.225	0.090	0.150	0.069
204	G	0.170	0.185	0.170	0.175	0.009	0.000	0.215	0.000	0.072	0.124
205	U	0.045	0.065	0.000	0.037	0.033	0.030	0.000	0.020	0.017	0.015
206	A	0.235	0.240	0.220	0.232	0.010	0.030	0.000	0.030	0.020	0.017
207	U	0.270	0.285	0.230	0.262	0.028	0.130	0.145	0.110	0.128	0.018
208	U	0.345	0.320	0.310	0.325	0.018	0.185	0.360	0.170	0.238	0.106
209	G	0.685	0.695	0.820	0.733	0.075	0.580	0.575	0.470	0.542	0.062
210	U	0.130	0.130	0.190	0.150	0.035	0.160	0.115	0.100	0.125	0.031
211	C	0.000	0.000	0.000	0.000	0.000	0.000	0.000	0.000	0.000	0.000
212	U	0.000	0.000	0.000	0.000	0.000	0.000	0.000	0.000	0.000	0.000
213	C	0.000	0.000	0.000	0.000	0.000	0.000	0.000	0.000	0.000	0.000
214	U	0.000	0.000	0.000	0.000	0.000	0.000	0.000	0.000	0.000	0.000
215	U	0.000	0.000	0.000	0.000	0.000	0.000	0.000	0.000	0.000	0.000
216	U	0.025	0.070	0.070	0.055	0.026	0.000	0.030	0.000	0.010	0.017
217	C	0.000	0.000	0.000	0.000	0.000	0.000	0.000	0.000	0.000	0.000
218	U	0.160	0.170	0.095	0.142	0.041	0.050	0.055	0.060	0.055	0.005
219	U	0.415	0.415	0.355	0.395	0.035	0.355	0.350	0.285	0.330	0.039
220	G	0.580	0.615	0.545	0.580	0.035	0.460	0.340	0.380	0.393	0.061
221	U	0.165	0.210	0.110	0.162	0.050	0.180	0.100	0.080	0.120	0.053
222	C	0.055	0.085	0.060	0.067	0.016	0.145	0.030	0.110	0.095	0.059
223	U	0.220	0.260	0.255	0.245	0.022	0.235	0.285	0.205	0.242	0.040
224	G	0.190	0.185	0.370	0.248	0.105	0.190	0.280	0.195	0.222	0.051
225	G	0.000	0.000	0.000	0.000	0.000	0.000	0.000	0.000	0.000	0.000
226	C	0.000	0.000	0.000	0.000	0.000	0.000	0.000	0.000	0.000	0.000
227	U	0.350	0.305	0.240	0.298	0.055	0.320	0.260	0.385	0.322	0.063
228	A	0.750	0.695	0.725	0.723	0.028	0.555	0.490	0.505	0.517	0.034
229	U	0.125	0.290	0.210	0.208	0.083	0.385	0.000	0.340	0.242	0.210
230	C	0.000	0.000	0.000	0.000	0.000	0.000	0.000	0.000	0.000	0.000
231	A	0.570	0.545	0.375	0.497	0.106	0.640	0.000	0.680	0.440	0.382
232	U	0.000	0.085	0.080	0.055	0.048	0.365	0.350	0.330	0.348	0.018
233	C	0.000	0.000	0.000	0.000	0.000	0.000	0.000	0.000	0.000	0.000
234	A	0.120	0.250	0.000	0.123	0.125	0.535	0.490	0.530	0.518	0.025
235	C	0.000	0.000	0.000	0.000	0.000	0.000	0.000	0.000	0.000	0.000
236	A	0.460	0.465	0.160	0.362	0.175	0.560	0.000	0.600	0.387	0.335
237	A	1.090	1.060	0.980	1.043	0.057	1.020	0.825	0.870	0.905	0.102
238	G	0.710	0.765	0.595	0.690	0.087	0.585	0.440	0.480	0.502	0.075
239	A	0.295	0.290	0.265	0.283	0.016	0.275	0.140	0.215	0.210	0.068
240	G	0.105	0.105	0.100	0.103	0.003	0.095	0.000	0.060	0.052	0.048

hSHAPE Reactivities from 3 independent experiments (SP105*i*)											
Nucleotides		In the Absence of Pr77^{Gag}					In the Presence of Pr77^{Gag}				
Number	Sequence	Expt 1	Expt 2	Expt 3	Mean	SD	Expt 1	Expt 2	Expt 3	Mean	SD
241	C	0.010	0.025	0.000	0.012	0.013	0.000	0.000	0.010	0.003	0.006
242	G	0.285	0.275	0.460	0.340	0.104	0.000	0.190	0.000	0.063	0.110
243	G	0.090	0.175	0.145	0.137	0.043	0.000	0.540	0.000	0.180	0.312
244	A	0.480	0.485	0.505	0.490	0.013	0.500	0.455	0.335	0.430	0.085
245	A	0.645	0.625	0.640	0.637	0.010	0.615	0.615	0.475	0.568	0.081
246	C	0.170	0.170	0.250	0.197	0.046	0.290	0.260	0.230	0.260	0.030
247	G	0.000	0.000	0.000	0.000	0.000	0.000	0.000	0.000	0.000	0.000
248	G	0.000	0.000	0.000	0.000	0.000	0.000	0.000	0.000	0.000	0.000
249	A	2.910	2.845	3.205	2.987	0.192	3.735	3.135	3.055	3.308	0.372
250	C	0.000	0.000	0.000	0.000	0.000	0.000	0.000	0.000	0.000	0.000
251	U	0.000	0.000	0.000	0.000	0.000	0.050	0.000	0.010	0.020	0.026
252	C	0.000	0.030	0.000	0.010	0.017	0.055	0.000	0.095	0.050	0.048
253	A	0.240	0.265	0.390	0.298	0.080	0.195	0.140	0.150	0.162	0.029
254	C	0.000	0.000	0.000	0.000	0.000	0.000	0.000	0.000	0.000	0.000
255	C	0.000	0.000	0.000	0.000	0.000	0.000	0.000	0.000	0.000	0.000
256	A	0.325	0.270	0.250	0.282	0.039	0.545	0.245	0.700	0.497	0.231
257	U	0.540	0.480	0.375	0.465	0.084	0.795	0.710	0.860	0.788	0.075
258	A	0.955	0.900	1.000	0.952	0.050	1.030	1.020	0.935	0.995	0.052
259	G	0.155	0.090	0.125	0.123	0.033	0.125	0.115	0.120	0.120	0.005
260	G	0.050	0.000	0.020	0.023	0.025	0.000	0.010	0.000	0.003	0.006
261	G	0.090	0.010	0.040	0.047	0.040	0.000	0.000	0.065	0.022	0.038
262	A	0.570	0.485	0.465	0.507	0.056	0.340	0.425	0.460	0.408	0.062
263	G	0.520	0.545	0.580	0.548	0.030	0.325	0.410	0.345	0.360	0.044
264	C	0.235	0.430	0.395	0.353	0.104	0.120	0.230	0.150	0.167	0.057
265	U	1.435	1.720	1.720	1.625	0.165	1.300	1.355	1.060	1.238	0.157
266	G	1.130	1.345	1.425	1.300	0.153	0.510	0.560	0.640	0.570	0.066
267	C	0.000	0.000	0.000	0.000	0.000	0.000	0.000	0.000	0.000	0.000
268	A	1.560	1.285	1.550	1.465	0.156	0.925	0.945	0.910	0.927	0.018
269	G	0.000	0.000	0.000	0.000	0.000	0.000	0.150	0.000	0.050	0.087
270	U	0.000	0.000	0.030	0.010	0.017	0.000	0.000	0.000	0.000	0.000
271	C	0.000	0.000	0.000	0.000	0.000	0.000	0.015	0.000	0.005	0.009
272	C	0.000	0.000	0.000	0.000	0.000	0.000	0.000	0.000	0.000	0.000
273	C	0.000	0.000	0.000	0.000	0.000	0.000	0.000	0.000	0.000	0.000
274	G	0.000	0.000	0.000	0.000	0.000	0.000	0.000	0.000	0.000	0.000
275	C	0.000	0.000	0.000	0.000	0.000	0.000	0.000	0.000	0.000	0.000
276	C	0.000	0.000	0.000	0.000	0.000	0.000	0.000	0.025	0.008	0.014
277	U	0.050	0.055	0.020	0.042	0.019	0.015	0.000	0.045	0.020	0.023
278	A	0.075	0.070	0.055	0.067	0.010	0.025	0.070	0.025	0.040	0.026
279	C	0.130	0.105	0.130	0.122	0.014	0.290	0.230	0.150	0.223	0.070
280	G	0.330	0.320	0.405	0.352	0.046	0.480	0.505	0.450	0.478	0.028
281	G	1.390	1.470	1.840	1.567	0.240	1.905	1.940	1.490	1.778	0.250
282	A	2.620	2.640	3.125	2.795	0.286	3.235	3.555	2.875	3.222	0.340
283	G	1.075	1.085	1.485	1.215	0.234	1.405	1.330	1.150	1.295	0.131
284	A	2.760	2.670	2.755	2.728	0.051	2.440	2.505	2.785	2.577	0.183
285	A	2.640	2.485	2.535	2.553	0.079	2.675	1.675	2.445	2.265	0.524
286	G	1.350	1.700	2.010	1.687	0.330	1.790	1.420	2.000	1.737	0.294
287	A	2.330	2.550	3.015	2.632	0.350	2.910	3.050	2.930	2.963	0.076
288	G	1.145	1.260	1.900	1.435	0.407	1.250	1.465	1.220	1.312	0.134

hSHAPE Reactivities from 3 independent experiments (SP105*i*)											
Nucleotides		In the Absence of Pr77^{Gag}					In the Presence of Pr77^{Gag}				
**Number**	**Sequence**	**Expt 1**	**Expt 2**	**Expt 3**	**Mean**	**SD**	**Expt 1**	**Expt 2**	**Expt 3**	**Mean**	**SD**
289	G	0.000	0.000	0.000	0.000	0.000	0.000	0.645	0.000	0.215	0.372
290	U	0.000	0.000	0.000	0.000	0.000	0.000	0.155	0.000	0.052	0.089
291	A	0.000	0.005	0.115	0.040	0.065	0.000	0.000	0.000	0.000	0.000
292	G	0.000	0.000	0.000	0.000	0.000	0.000	0.000	0.000	0.000	0.000
293	G	0.000	0.000	0.000	0.000	0.000	0.000	0.000	0.000	0.000	0.000
294	U	0.000	0.035	0.000	0.012	0.020	0.130	0.120	0.150	0.133	0.015
295	U	0.500	0.680	0.610	0.597	0.091	0.400	0.290	0.355	0.348	0.055
296	A	1.135	1.200	1.470	1.268	0.178	1.320	1.010	0.950	1.093	0.199
297	C	0.675	0.740	0.820	0.745	0.073	1.240	1.260	1.270	1.257	0.015
298	G	0.020	0.020	0.040	0.027	0.012	0.755	0.705	0.725	0.728	0.025
299	G	0.000	0.000	0.000	0.000	0.000	0.000	0.145	0.000	0.048	0.084
300	U	0.000	0.000	0.000	0.000	0.000	0.000	0.025	0.000	0.008	0.014
301	G	0.000	0.000	0.000	0.000	0.000	0.000	0.000	0.000	0.000	0.000
302	A	0.000	0.000	0.000	0.000	0.000	0.000	0.000	0.000	0.000	0.000
303	G	0.000	0.000	0.000	0.000	0.000	0.000	0.000	0.000	0.000	0.000
304	C	0.000	0.000	0.000	0.000	0.000	0.000	0.000	0.000	0.000	0.000
305	C	0.000	0.000	0.000	0.000	0.000	0.000	0.000	0.000	0.000	0.000
306	A	0.320	0.400	0.540	0.420	0.111	0.290	0.360	0.410	0.353	0.060
307	U	0.780	0.870	0.815	0.822	0.045	1.055	0.995	1.010	1.020	0.031
308	U	1.205	1.240	1.270	1.238	0.033	1.465	2.130	1.255	1.617	0.457
309	G	1.310	1.150	1.410	1.290	0.131	1.100	0.850	1.060	1.003	0.134
310	G	1.190	1.010	1.290	1.163	0.142	0.915	0.750	0.950	0.872	0.107
311	A	1.010	0.870	0.970	0.950	0.072	0.900	0.830	0.860	0.863	0.035
312	A	0.700	0.640	0.740	0.693	0.050	0.700	0.500	0.650	0.617	0.104
313	A	0.520	0.460	0.520	0.500	0.035	0.570	0.500	0.500	0.523	0.040
314	U	0.500	0.440	0.480	0.473	0.031	0.550	0.560	0.520	0.543	0.021
315	G	0.000	0.030	0.030	0.020	0.017	0.000	0.040	0.000	0.013	0.023
316	G	0.000	0.000	0.050	0.017	0.029	0.000	0.000	0.000	0.000	0.000
317	G	0.000	0.000	0.000	0.000	0.000	0.000	0.000	0.000	0.000	0.000
318	G	0.000	0.000	0.000	0.000	0.000	0.000	4.220	0.000	1.407	2.436
319	G	0.000	0.000	0.000	0.000	0.000	0.000	0.000	0.000	0.000	0.000
320	U	0.210	0.270	0.230	0.237	0.031	0.000	0.000	0.000	0.000	0.000
321	C	0.000	0.000	0.000	0.000	0.000	0.000	0.000	0.000	0.000	0.000
322	U	0.290	0.260	0.180	0.243	0.057	0.190	0.170	0.190	0.183	0.012
323	C	0.150	0.170	0.070	0.130	0.053	0.000	0.000	0.050	0.017	0.029
324	G	0.430	0.320	0.360	0.370	0.056	0.170	0.250	0.320	0.247	0.075
325	G	0.160	0.280	0.410	0.283	0.125	0.000	0.000	0.160	0.053	0.092
326	G	0.000	0.000	0.000	0.000	0.000	0.000	0.000	0.000	0.000	0.000
327	C	0.000	0.000	0.000	0.000	0.000	0.000	0.000	0.000	0.000	0.000
328	U	0.010	0.110	0.170	0.097	0.081	0.000	0.000	0.200	0.067	0.115
329	C	0.000	0.420	0.000	0.140	0.242	0.720	0.000	0.340	0.353	0.360
330	A	0.810	0.710	0.670	0.730	0.072	0.720	0.310	0.800	0.610	0.263
331	A	1.500	1.220	1.280	1.333	0.147	1.370	1.060	1.290	1.240	0.161
332	A	1.440	1.210	1.110	1.253	0.169	1.230	1.000	1.140	1.123	0.116
333	A	1.230	1.060	0.970	1.087	0.132	0.980	0.700	0.950	0.877	0.154
334	G	0.330	0.270	0.180	0.260	0.075	0.120	0.120	0.150	0.130	0.017
335	G	0.030	0.000	0.010	0.013	0.015	0.000	0.000	0.000	0.000	0.000
336	G	0.000	0.000	0.000	0.000	0.000	0.000	0.000	0.000	0.000	0.000

hSHAPE Reactivities from 3 independent experiments (SP105*i*)											
Nucleotides		In the Absence of Pr77^{Gag}					In the Presence of Pr77^{Gag}				
Number	Sequence	Expt 1	Expt 2	Expt 3	Mean	SD	Expt 1	Expt 2	Expt 3	Mean	SD
337	C	0.040	0.040	0.070	0.050	0.017	0.120	0.000	0.120	0.080	0.069
338	A	0.120	0.030	0.100	0.083	0.047	0.030	0.000	0.090	0.040	0.046
339	G	0.000	0.000	0.010	0.003	0.006	0.000	0.000	0.000	0.000	0.000
340	A	0.230	0.160	0.200	0.197	0.035	0.100	0.150	0.200	0.150	0.050
341	A	0.410	0.310	0.320	0.347	0.055	0.270	0.280	0.330	0.293	0.032
342	A	0.210	0.190	0.130	0.177	0.042	0.100	0.190	0.200	0.163	0.055
343	C	0.000	0.050	0.000	0.017	0.029	0.010	0.010	0.020	0.013	0.006
344	U	0.870	0.810	0.580	0.753	0.153	1.130	0.970	1.080	1.060	0.082
345	C	0.580	0.560	0.480	0.540	0.053	0.770	0.530	0.640	0.647	0.120
346	U	0.520	0.480	0.560	0.520	0.040	0.710	0.390	0.650	0.583	0.170
347	U	1.010	0.900	0.850	0.920	0.082	1.240	1.010	1.170	1.140	0.118
348	U	0.870	0.770	0.710	0.783	0.081	1.180	1.120	1.140	1.147	0.031
349	G	0.150	0.120	0.160	0.143	0.021	0.000	0.060	0.060	0.040	0.035
350	U	0.370	0.340	0.250	0.320	0.062	0.300	0.310	0.420	0.343	0.067
351	U	0.450	0.380	0.300	0.377	0.075	0.270	0.340	0.350	0.320	0.044
352	U	0.000	0.000	0.010	0.003	0.006	0.000	0.030	0.010	0.013	0.015
353	C	0.000	0.000	0.000	0.000	0.000	0.000	0.000	0.000	0.000	0.000
354	U	0.640	0.290	0.380	0.437	0.182	0.560	0.590	0.590	0.580	0.017
355	G	2.020	1.690	1.880	1.863	0.166	2.480	1.370	2.060	1.970	0.560
356	U	0.240	0.180	0.220	0.213	0.031	0.320	0.260	0.320	0.300	0.035
357	U	0.380	0.280	0.320	0.327	0.050	0.430	0.380	0.470	0.427	0.045
358	U	0.350	0.320	0.310	0.327	0.021	0.490	1.480	0.500	0.823	0.569
359	U	0.370	0.440	0.030	0.280	0.219	0.510	3.180	0.530	1.407	1.536
360	A	0.550	0.530	0.460	0.513	0.047	0.770	0.760	0.740	0.757	0.015
361	C	0.000	0.220	0.000	0.073	0.127	0.610	0.490	0.350	0.483	0.130
362	A	0.360	0.330	0.270	0.320	0.046	0.390	0.140	0.370	0.300	0.139
363	A	0.180	0.160	0.140	0.160	0.020	0.170	0.130	0.190	0.163	0.031
364	A	0.060	0.060	0.040	0.053	0.012	0.050	0.040	0.070	0.053	0.015
365	G	0.000	0.000	0.000	0.000	0.000	0.000	0.000	0.000	0.000	0.000
366	G	0.000	0.030	0.000	0.010	0.017	0.000	0.000	0.000	0.000	0.000
367	C	0.000	0.000	0.000	0.000	0.000	0.000	0.000	0.000	0.000	0.000
368	U	0.000	0.020	0.000	0.007	0.012	0.000	0.000	0.000	0.000	0.000
369	C	0.000	0.000	0.000	0.000	0.000	0.000	0.000	0.000	0.000	0.000
370	C	0.000	0.000	0.000	0.000	0.000	0.000	0.000	0.000	0.000	0.000
371	U	0.000	0.000	0.040	0.013	0.023	0.000	0.010	0.030	0.013	0.015
372	C	0.030	0.020	0.060	0.037	0.021	0.090	0.080	0.130	0.100	0.026
373	U	0.300	0.370	0.210	0.293	0.080	0.540	0.390	0.580	0.503	0.100
374	C	1.360	1.600	1.530	1.497	0.123	2.170	3.680	2.190	2.680	0.866
375	A	3.100	2.960	2.730	2.930	0.187	3.860	2.760	3.360	3.327	0.551
376	G	2.070	1.530	1.790	1.797	0.270	2.670	1.940	2.500	2.370	0.382
377	A	2.160	1.950	1.960	2.023	0.118	2.740	1.960	2.420	2.373	0.392
378	G	0.000	0.000	0.000	0.000	0.000	0.000	0.000	0.020	0.007	0.012
379	A	0.000	0.000	0.000	0.000	0.000	0.000	0.000	0.000	0.000	0.000
380	G	0.000	0.000	0.000	0.000	0.000	0.000	0.000	0.000	0.000	0.000
381	G	0.000	0.000	0.050	0.017	0.029	0.000	0.000	0.000	0.000	0.000
382	G	0.000	0.000	0.000	0.000	0.000	0.000	6.690	0.000	2.230	3.862
383	G	0.000	0.000	0.000	0.000	0.000	0.000	0.000	0.000	0.000	0.000
384	U	0.320	0.450	0.390	0.387	0.065	0.000	0.120	0.000	0.040	0.069

hSHAPE Reactivities from 3 independent experiments (SP105*i*)											
**Nucleotides**		**In the Absence of Pr77^{Gag}**					**In the Presence of Pr77^{Gag}**				
**Number**	**Sequence**	**Expt 1**	**Expt 2**	**Expt 3**	**Mean**	**SD**	**Expt 1**	**Expt 2**	**Expt 3**	**Mean**	**SD**
385	C	0.000	0.000	0.000	0.000	0.000	0.000	0.000	0.000	0.000	0.000
386	U	0.180	0.160	0.130	0.157	0.025	0.160	0.000	0.060	0.073	0.081
387	U	0.230	0.780	0.520	0.510	0.275	1.110	1.270	0.910	1.097	0.180
388	C	1.100	1.410	0.900	1.137	0.257	1.880	0.520	1.000	1.133	0.690
389	A	0.900	0.830	0.860	0.863	0.035	1.190	0.070	0.990	0.750	0.597
390	U	0.100	0.100	0.140	0.113	0.023	0.140	0.910	0.100	0.383	0.457
391	G	0.160	0.030	0.020	0.070	0.078	0.020	0.000	0.000	0.007	0.012
392	U	0.280	0.070	0.030	0.127	0.134	0.150	0.140	0.120	0.137	0.015
393	G	0.090	0.070	0.050	0.070	0.020	0.090	0.480	0.050	0.207	0.238
394	A	0.170	0.140	0.110	0.140	0.030	0.210	0.760	0.140	0.370	0.340
395	A	0.260	0.280	0.220	0.253	0.031	0.380	0.600	0.300	0.427	0.155
396	A	0.330	0.520	0.580	0.477	0.131	0.850	0.000	0.720	0.523	0.458
397	G	0.650	0.590	0.640	0.627	0.032	0.810	0.800	0.730	0.780	0.044
398	A	0.470	0.300	0.230	0.333	0.123	0.300	12.350	0.260	4.303	6.969
399	G	0.230	0.340	0.290	0.287	0.055	0.250	0.000	0.170	0.140	0.128
400	A	0.190	0.450	0.350	0.330	0.131	0.370	0.000	0.330	0.233	0.203
401	G	0.150	0.140	0.950	0.413	0.465	0.300	0.480	0.020	0.267	0.232
402	U	0.510	1.130	0.870	0.837	0.311	1.060	0.330	1.010	0.800	0.408
403	A	0.550	0.330	0.480	0.453	0.112	0.460	0.000	0.400	0.287	0.250
404	G	0.410	0.130	0.220	0.253	0.143	0.200	0.000	0.220	0.140	0.122
405	U	0.540	0.180	0.000	0.240	0.275	0.450	0.000	0.490	0.313	0.272
406	G	0.660	0.520	0.100	0.427	0.291	0.120	0.780	0.080	0.327	0.393
407	C	0.510	0.890	0.750	0.717	0.192	1.060	4.760	0.850	2.223	2.199
408	A	0.440	0.390	0.120	0.317	0.172	0.820	0.620	0.870	0.770	0.132
409	A	0.500	0.490	0.450	0.480	0.026	0.710	0.390	0.690	0.597	0.179
410	U	0.470	0.940	0.680	0.697	0.235	0.760	0.670	0.820	0.750	0.075
411	A	0.390	0.440	0.410	0.413	0.025	0.780	0.740	0.700	0.740	0.040
412	G	0.200	0.340	0.380	0.307	0.095	0.470	0.700	0.520	0.563	0.121
413	A	0.500	0.400	0.420	0.440	0.053	0.640	0.650	0.630	0.640	0.010
414	A	0.520	0.380	0.430	0.443	0.071	0.680	0.550	0.600	0.610	0.066
415	U	0.550	0.320	0.380	0.417	0.119	0.600	5.480	0.560	2.213	2.829
416	U	0.150	0.230	0.270	0.217	0.061	0.360	0.370	0.300	0.343	0.038
417	U	0.470	0.540	0.450	0.487	0.047	0.970	0.280	0.860	0.703	0.371
418	U	0.730	1.000	0.870	0.867	0.135	0.640	2.990	0.710	1.447	1.337
419	A	0.380	0.570	0.600	0.517	0.119	0.830	0.590	0.780	0.733	0.127
420	U	0.200	0.270	0.290	0.253	0.047	0.360	0.500	0.330	0.397	0.091
421	C	0.500	1.080	0.570	0.717	0.317	1.320	0.370	0.530	0.740	0.509
422	A	0.210	0.690	0.620	0.507	0.259	1.480	4.750	1.500	2.577	1.882
423	G	0.400	0.340	0.330	0.357	0.038	0.890	0.810	0.870	0.857	0.042
424	U	0.390	0.310	0.300	0.333	0.049	0.760	0.760	0.760	0.760	0.000
425	U	0.210	0.210	0.150	0.190	0.035	0.450	0.610	0.400	0.487	0.110
426	U	0.280	0.030	0.030	0.113	0.144	0.870	0.980	0.150	0.667	0.451
427	C	-999	-999	-999	-999	0.000	-999	-999	-999	-999	0.000
428	U	-999	-999	-999	-999	0.000	-999	-999	-999	-999	0.000
429	A	-999	-999	-999	-999	0.000	-999	-999	-999	-999	0.000
430	A	-999	-999	-999	-999	0.000	-999	-999	-999	-999	0.000
431	U	-999	-999	-999	-999	0.000	-999	-999	-999	-999	0.000
432	A	-999	-999	-999	-999	0.000	-999	-999	-999	-999	0.000

hSHAPE Reactivities from 3 independent experiments (SP106*i*)											
Nucleotides		In the Absence of Pr77^{Gag}					In the Presence of Pr77^{Gag}				
Number	Sequence	Expt 1	Expt 2	Expt 3	Mean	SD	Expt 1	Expt 2	Expt 3	Mean	SD
1	G	-999	-999	-999	-999	0.000	-999	-999	-999	-999	0.000
2	C	-999	-999	-999	-999	0.000	-999	-999	-999	-999	0.000
3	A	-999	-999	-999	-999	0.000	-999	-999	-999	-999	0.000
4	A	-999	-999	-999	-999	0.000	-999	-999	-999	-999	0.000
5	C	-999	-999	-999	-999	0.000	-999	-999	-999	-999	0.000
6	A	-999	-999	-999	-999	0.000	-999	-999	-999	-999	0.000
7	G	-999	-999	-999	-999	0.000	-999	-999	-999	-999	0.000
8	U	-999	-999	-999	-999	0.000	-999	-999	-999	-999	0.000
9	C	-999	-999	-999	-999	0.000	-999	-999	-999	-999	0.000
10	C	-999	-999	-999	-999	0.000	-999	-999	-999	-999	0.000
11	U	-999	-999	-999	-999	0.000	-999	-999	-999	-999	0.000
12	A	-999	-999	-999	-999	0.000	-999	-999	-999	-999	0.000
13	A	-999	-999	-999	-999	0.000	-999	-999	-999	-999	0.000
14	U	-999	-999	-999	-999	0.000	-999	-999	-999	-999	0.000
15	A	-999	-999	-999	-999	0.000	-999	-999	-999	-999	0.000
16	U	-999	-999	-999	-999	0.000	-999	-999	-999	-999	0.000
17	U	-999	-999	-999	-999	0.000	-999	-999	-999	-999	0.000
18	C	-999	-999	-999	-999	0.000	-999	-999	-999	-999	0.000
19	A	0.160	0.200	0.160	0.173	0.023	0.175	0.185	0.260	0.207	0.046
20	C	0.060	0.060	0.070	0.063	0.006	0.160	0.200	0.400	0.253	0.129
21	G	0.370	0.490	0.570	0.477	0.101	0.570	0.715	0.485	0.590	0.116
22	U	1.830	1.830	1.920	1.860	0.052	2.040	2.530	1.480	2.017	0.525
23	C	0.520	0.535	0.695	0.583	0.097	0.405	0.835	0.655	0.632	0.216
24	U	2.760	2.840	3.040	2.880	0.144	3.395	3.530	2.780	3.235	0.400
25	C	0.880	0.695	0.850	0.808	0.099	1.095	1.150	0.825	1.023	0.174
26	G	0.270	0.390	0.365	0.342	0.063	0.445	0.275	0.220	0.313	0.117
27	U	0.130	0.140	0.110	0.127	0.015	0.040	0.055	0.070	0.055	0.015
28	G	0.000	0.000	0.000	0.000	0.000	0.355	0.000	0.270	0.208	0.185
29	U	0.145	0.320	0.295	0.253	0.095	0.530	0.405	0.350	0.428	0.092
30	G	0.190	0.350	0.395	0.312	0.108	0.475	0.380	0.390	0.415	0.052
31	U	0.170	0.140	0.165	0.158	0.016	0.285	0.130	0.050	0.155	0.119
32	U	0.370	0.410	0.515	0.432	0.075	0.420	0.405	0.290	0.372	0.071
33	U	0.320	0.330	0.395	0.348	0.041	0.395	0.360	0.295	0.350	0.051
34	G	0.300	0.450	0.365	0.372	0.075	0.560	0.475	0.300	0.445	0.133
35	U	1.610	1.800	1.620	1.677	0.107	2.500	2.270	2.120	2.297	0.191
36	G	0.415	0.450	0.470	0.445	0.028	0.550	0.445	0.360	0.452	0.095
37	U	0.185	0.200	0.100	0.162	0.054	0.050	0.130	0.060	0.080	0.044
38	C	0.160	0.200	0.130	0.163	0.035	0.090	0.140	0.070	0.100	0.036
39	U	0.000	0.035	0.075	0.037	0.038	0.265	0.100	0.075	0.147	0.103
40	G	0.010	0.090	0.040	0.047	0.040	0.200	0.385	0.080	0.222	0.154
41	U	0.000	0.000	0.000	0.000	0.000	0.295	0.190	0.090	0.192	0.103
42	U	0.420	0.250	0.265	0.312	0.094	0.310	0.435	0.495	0.413	0.094
43	C	0.685	0.540	0.545	0.590	0.082	0.700	0.765	0.795	0.753	0.049
44	G	0.535	0.470	0.250	0.418	0.149	0.520	0.620	0.385	0.508	0.118
45	C	0.000	0.000	0.000	0.000	0.000	0.000	0.000	0.000	0.000	0.000
46	C	0.000	0.000	0.000	0.000	0.000	0.000	0.000	0.000	0.000	0.000
47	A	0.970	1.020	1.105	1.032	0.068	0.910	0.680	0.345	0.645	0.284
48	U	1.320	1.330	1.350	1.333	0.015	0.945	1.020	0.985	0.983	0.038

hSHAPE Reactivities from 3 independent experiments (SP106*i*)											
Nucleotides		In the Absence of Pr77^{Gag}					In the Presence of Pr77^{Gag}				
Number	Sequence	Expt 1	Expt 2	Expt 3	Mean	SD	Expt 1	Expt 2	Expt 3	Mean	SD
49	C	0.575	0.810	0.680	0.688	0.118	0.610	0.570	0.495	0.558	0.058
50	C → G	0.400	0.520	0.475	0.465	0.061	0.500	0.275	0.240	0.338	0.141
51	C	0.000	0.040	0.100	0.047	0.050	0.175	0.090	0.100	0.122	0.046
52	G → U	0.555	0.600	0.770	0.642	0.113	0.760	0.555	0.490	0.602	0.141
53	U	0.685	0.720	0.735	0.713	0.026	0.675	0.635	0.520	0.610	0.080
54	C	0.285	0.340	0.360	0.328	0.039	0.400	0.450	0.260	0.370	0.098
55	U	0.130	0.130	0.080	0.113	0.029	0.120	0.060	0.035	0.072	0.044
56	C	0.000	0.000	0.000	0.000	0.000	0.000	0.000	0.000	0.000	0.000
57	C	0.000	0.000	0.000	0.000	0.000	0.000	0.000	0.000	0.000	0.000
58	G	0.000	0.000	0.000	0.000	0.000	0.000	0.000	0.000	0.000	0.000
59	C	0.000	0.000	0.000	0.000	0.000	0.100	0.000	0.000	0.033	0.058
60	U	0.470	0.400	0.355	0.408	0.058	0.445	0.440	0.405	0.430	0.022
61	C	0.060	0.175	0.200	0.145	0.075	0.620	0.510	0.335	0.488	0.144
62	G	0.000	0.000	0.000	0.000	0.000	0.000	0.000	0.000	0.000	0.000
63	U	0.010	0.125	0.145	0.093	0.073	0.000	0.000	0.000	0.000	0.000
64	C	0.230	0.230	0.350	0.270	0.069	0.000	0.000	0.000	0.000	0.000
65	A	0.180	0.315	0.315	0.270	0.078	0.000	0.125	0.000	0.042	0.072
66	C	0.000	0.000	0.000	0.000	0.000	0.065	0.000	0.000	0.022	0.038
67	U	0.355	0.295	0.310	0.320	0.031	0.305	0.210	0.175	0.230	0.067
68	U	0.190	0.340	0.255	0.262	0.075	0.310	0.360	0.210	0.293	0.076
69	A	0.555	0.475	0.545	0.525	0.044	0.450	0.340	0.325	0.372	0.068
70	U	0.275	0.250	0.235	0.253	0.020	0.205	0.105	0.085	0.132	0.064
71	C	0.015	0.120	0.000	0.045	0.065	0.000	0.000	0.000	0.000	0.000
72	C	0.000	0.000	0.000	0.000	0.000	0.085	0.000	0.190	0.092	0.095
73	U	0.310	0.735	0.465	0.503	0.215	0.820	0.565	0.850	0.745	0.157
74	U	1.330	1.490	1.470	1.430	0.087	1.580	1.845	1.240	1.555	0.303
75	C	0.000	0.000	0.055	0.018	0.032	0.000	0.000	0.000	0.000	0.000
76	A	0.980	1.190	1.165	1.112	0.115	0.550	0.650	0.640	0.613	0.055
77	C	0.060	0.170	0.160	0.130	0.061	0.010	0.000	0.100	0.037	0.055
78	U	1.015	1.055	1.150	1.073	0.069	0.865	0.830	0.430	0.708	0.242
79	U	1.330	1.450	1.430	1.403	0.064	1.080	0.975	0.720	0.925	0.185
80	U	1.050	0.950	1.255	1.085	0.155	0.690	0.665	0.780	0.712	0.060
81	C	0.545	0.735	0.640	0.640	0.095	0.245	0.345	0.440	0.343	0.098
82	C	0.145	0.190	0.220	0.185	0.038	0.000	0.000	0.000	0.000	0.000
83	A	0.000	0.000	0.000	0.000	0.000	1.665	1.800	1.460	1.642	0.171
84	G	1.670	1.830	1.900	1.800	0.118	0.110	0.740	0.970	0.607	0.445
85	A	0.060	0.160	0.075	0.098	0.054	0.060	0.165	0.000	0.075	0.084
86	G	0.000	0.030	0.000	0.010	0.017	0.000	0.000	0.000	0.000	0.000
87	G	0.000	0.000	0.000	0.000	0.000	0.000	0.000	0.000	0.000	0.000
88	G	0.000	0.000	0.000	0.000	0.000	0.000	0.000	0.000	0.000	0.000
89	U	0.000	0.000	0.000	0.000	0.000	0.000	0.000	0.000	0.000	0.000
90	C	0.000	0.000	0.000	0.000	0.000	0.000	0.000	0.000	0.000	0.000
91	C	0.000	0.000	0.000	0.000	0.000	0.000	0.000	0.000	0.000	0.000
92	C	0.000	0.000	0.000	0.000	0.000	0.000	0.000	0.000	0.000	0.000
93	C	0.000	0.000	0.000	0.000	0.000	0.000	0.000	0.000	0.000	0.000
94	C	0.000	0.000	0.000	0.000	0.000	0.000	0.000	0.000	0.000	0.000
95	C	0.000	0.000	0.000	0.000	0.000	0.000	0.000	0.000	0.000	0.000
96	G	0.000	0.005	0.040	0.015	0.022	0.080	0.000	0.080	0.053	0.046

hSHAPE Reactivities from 3 independent experiments (SP106*i*)											
Nucleotides		In the Absence of Pr77^{Gag}					In the Presence of Pr77^{Gag}				
Number	Sequence	Expt 1	Expt 2	Expt 3	Mean	SD	Expt 1	Expt 2	Expt 3	Mean	SD
97	C	0.000	0.000	0.000	0.000	0.000	0.000	0.000	0.000	0.000	0.000
98	A	0.345	0.340	0.485	0.390	0.082	0.355	0.310	0.385	0.350	0.038
99	G	0.730	0.750	0.810	0.763	0.042	0.545	0.440	0.490	0.492	0.053
100	A	1.775	1.935	1.940	1.883	0.094	1.535	1.605	1.785	1.642	0.129
101	C	0.215	0.255	0.325	0.265	0.056	0.085	0.090	0.155	0.110	0.039
102	C	0.000	0.130	0.055	0.062	0.065	0.460	0.040	0.370	0.290	0.221
103	C	0.000	0.000	0.000	0.000	0.000	0.000	0.000	0.000	0.000	0.000
104	C	0.000	0.000	0.000	0.000	0.000	0.000	0.000	0.000	0.000	0.000
105	G	0.000	0.000	0.000	0.000	0.000	0.000	0.000	0.000	0.000	0.000
106	G	0.000	0.000	0.000	0.000	0.000	0.000	0.000	0.000	0.000	0.000
107	U	0.060	0.030	0.090	0.060	0.030	0.050	0.075	0.120	0.082	0.035
108	G	0.040	0.000	0.045	0.028	0.025	0.005	0.050	0.120	0.058	0.058
109	A	0.005	0.000	0.085	0.030	0.048	0.000	0.000	0.000	0.000	0.000
110	C	0.000	0.000	0.000	0.000	0.000	0.000	0.000	0.000	0.000	0.000
111	C	0.000	0.000	0.000	0.000	0.000	0.000	0.000	0.000	0.000	0.000
112	C	0.000	0.000	0.000	0.000	0.000	0.000	0.000	0.000	0.000	0.000
113	U	1.820	2.075	2.275	2.057	0.228	1.965	1.890	2.200	2.018	0.162
114	C	0.670	0.720	1.090	0.827	0.229	0.790	0.485	0.650	0.642	0.153
115	A	3.045	3.800	3.465	3.437	0.378	2.790	3.265	2.590	2.882	0.347
116	G	0.235	0.400	0.325	0.320	0.083	0.230	0.275	0.300	0.268	0.035
117	G	0.030	0.010	0.020	0.020	0.010	0.260	0.045	0.020	0.108	0.132
118	U	0.040	0.000	0.000	0.013	0.023	0.000	0.040	0.015	0.018	0.020
119	C	0.235	0.185	0.325	0.248	0.071	0.000	0.000	0.000	0.000	0.000
120	G	0.505	0.560	0.545	0.537	0.028	0.400	0.660	0.340	0.467	0.170
121	G	0.650	0.700	0.590	0.647	0.055	0.920	0.885	0.870	0.892	0.026
122	C	0.000	0.000	0.000	0.000	0.000	0.300	0.040	0.070	0.137	0.142
123	C	0.000	0.000	0.000	0.000	0.000	0.000	0.000	0.000	0.000	0.000
124	G	0.050	0.010	0.100	0.053	0.045	0.050	0.000	0.075	0.042	0.038
125	A	0.300	0.265	0.220	0.262	0.040	0.105	0.215	0.215	0.178	0.064
126	C	0.000	0.000	0.000	0.000	0.000	0.235	0.000	0.100	0.112	0.118
127	U	0.095	0.040	0.030	0.055	0.035	0.045	0.000	0.020	0.022	0.023
128	G	0.200	0.185	0.130	0.172	0.037	0.110	0.130	0.160	0.133	0.025
129	C	0.000	0.000	0.000	0.000	0.000	0.030	0.140	0.090	0.087	0.055
130	G	0.000	0.000	0.000	0.000	0.000	0.000	0.000	0.000	0.000	0.000
131	G	0.000	0.000	0.000	0.000	0.000	0.000	0.000	0.000	0.000	0.000
132	C	0.000	0.000	0.000	0.000	0.000	0.000	0.000	0.000	0.000	0.000
133	A	0.000	0.000	0.000	0.000	0.000	0.000	0.000	0.000	0.000	0.000
134	G	1.040	0.750	0.950	0.913	0.148	0.540	1.170	0.940	0.883	0.319
135	C	0.580	0.560	0.755	0.632	0.107	0.790	0.420	0.410	0.540	0.217
136	U	0.530	0.250	0.660	0.480	0.210	0.410	0.705	0.340	0.485	0.194
137	G	0.235	0.100	0.245	0.193	0.081	0.081	0.029	0.011	0.040	0.036
138	G	0.000	0.000	0.000	0.000	0.000	0.000	0.000	0.000	0.000	0.000
139	C	0.000	0.000	0.000	0.000	0.000	0.000	0.000	0.000	0.000	0.000
140	G	0.000	0.025	0.000	0.008	0.014	0.000	0.000	0.000	0.000	0.000
141	C	0.000	0.000	0.000	0.000	0.000	0.000	0.000	0.000	0.000	0.000
142	C	0.000	0.000	0.000	0.000	0.000	0.000	0.000	0.000	0.000	0.000
143	C	0.000	0.000	0.000	0.000	0.000	0.000	0.000	0.170	0.057	0.098
144	G	0.610	0.320	0.550	0.493	0.153	0.290	0.160	0.670	0.373	0.265

hSHAPE Reactivities from 3 independent experiments (SP106*i*)											
Nucleotides		In the Absence of Pr77^{Gag}					In the Presence of Pr77^{Gag}				
**Number**	**Sequence**	**Expt 1**	**Expt 2**	**Expt 3**	**Mean**	**SD**	**Expt 1**	**Expt 2**	**Expt 3**	**Mean**	**SD**
145	A	0.935	0.800	1.010	0.915	0.106	0.885	0.905	1.050	0.947	0.090
146	A	1.025	0.985	0.975	0.995	0.026	0.950	0.780	0.670	0.800	0.141
147	C	0.620	0.890	0.470	0.660	0.213	0.000	0.000	0.000	0.000	0.000
148	A	0.505	0.590	0.575	0.557	0.045	0.300	0.625	0.530	0.485	0.167
149	G	0.000	0.020	0.000	0.007	0.012	0.130	0.020	0.075	0.075	0.055
150	G	0.000	0.000	0.000	0.000	0.000	0.210	0.000	0.000	0.070	0.121
151	G	0.000	0.000	0.000	0.000	0.000	0.000	0.000	0.000	0.000	0.000
152	A	0.000	0.000	0.000	0.000	0.000	0.000	0.000	0.000	0.000	0.000
153	C	0.000	0.000	0.000	0.000	0.000	0.000	0.000	0.000	0.000	0.000
154	C	0.000	0.000	0.000	0.000	0.000	0.000	0.000	0.000	0.000	0.000
155	C	0.000	0.000	0.000	0.000	0.000	0.000	0.000	0.000	0.000	0.000
156	U	0.000	0.000	0.000	0.000	0.000	0.000	0.000	0.000	0.000	0.000
157	C	0.280	0.280	0.240	0.267	0.023	0.000	0.180	0.005	0.062	0.103
158	G	0.000	0.000	0.000	0.000	0.000	0.000	0.000	0.000	0.000	0.000
159	G	0.000	0.000	0.000	0.000	0.000	0.000	0.000	0.000	0.000	0.000
160	A	0.000	0.000	0.000	0.000	0.000	0.000	0.000	0.000	0.000	0.000
161	U	0.210	0.120	0.180	0.170	0.046	0.045	0.120	0.120	0.095	0.043
162	A	0.030	0.030	0.000	0.020	0.017	0.040	0.000	0.000	0.013	0.023
163	A	0.020	0.040	0.030	0.030	0.010	0.050	0.080	0.280	0.137	0.125
164	G	0.000	0.000	0.000	0.000	0.000	0.000	0.000	0.000	0.000	0.000
165	U	0.000	0.000	0.000	0.000	0.000	0.000	0.000	0.000	0.000	0.000
166	G	0.000	0.000	0.000	0.000	0.000	0.000	0.000	0.000	0.000	0.000
167	A	0.000	0.000	0.000	0.000	0.000	0.000	0.000	0.030	0.010	0.017
168	C	0.000	0.000	0.000	0.000	0.000	0.000	0.000	0.000	0.000	0.000
169	C	0.000	0.000	0.000	0.000	0.000	0.000	0.000	0.000	0.000	0.000
170	C	0.000	0.000	0.000	0.000	0.000	0.000	0.000	0.000	0.000	0.000
171	U	0.060	0.280	0.330	0.223	0.144	0.045	0.100	0.160	0.102	0.058
172	U	0.405	0.425	0.375	0.402	0.025	0.495	0.225	0.295	0.338	0.140
173	G	0.800	1.025	0.805	0.877	0.128	0.920	0.670	0.680	0.757	0.142
174	U	0.555	0.565	0.445	0.522	0.067	0.440	0.330	0.450	0.407	0.067
175	C	0.000	0.000	0.000	0.000	0.000	0.000	0.000	0.000	0.000	0.000
176	U	0.000	0.000	0.000	0.000	0.000	0.000	0.000	0.000	0.000	0.000
177	C	0.000	0.000	0.000	0.000	0.000	0.000	0.000	0.000	0.000	0.000
178	U	0.000	0.000	0.000	0.000	0.000	0.000	0.000	0.000	0.000	0.000
179	A	0.300	0.280	0.390	0.323	0.059	0.605	0.050	0.640	0.432	0.331
180	U	0.100	0.140	0.105	0.115	0.022	0.195	0.070	0.200	0.155	0.074
181	U	0.055	0.045	0.120	0.073	0.041	0.185	0.085	0.120	0.130	0.051
182	U	0.080	0.055	0.050	0.062	0.016	0.035	0.035	0.075	0.048	0.023
183	C	0.000	0.000	0.000	0.000	0.000	0.000	0.000	0.000	0.000	0.000
184	U	0.030	0.025	0.150	0.068	0.071	0.000	0.000	0.000	0.000	0.000
185	A	0.150	0.115	0.130	0.132	0.018	0.225	0.090	0.225	0.180	0.078
186	C	0.000	0.000	0.000	0.000	0.000	0.135	0.040	0.100	0.092	0.048
187	U	0.470	0.440	0.630	0.513	0.102	0.230	0.390	0.330	0.317	0.081
188	A	0.980	0.800	0.725	0.835	0.131	0.620	0.575	0.560	0.585	0.031
189	U	0.210	0.125	0.150	0.162	0.044	0.010	0.060	0.190	0.087	0.093
190	U	0.120	0.040	0.140	0.100	0.053	0.000	0.000	0.000	0.000	0.000
191	U	0.160	0.110	0.140	0.137	0.025	0.045	0.000	0.000	0.015	0.026
192	G	0.045	0.030	0.030	0.035	0.009	0.000	0.000	0.000	0.000	0.000

hSHAPE Reactivities from 3 independent experiments (SP106*i*)											
Nucleotides		In the Absence of Pr77^{Gag}					In the Presence of Pr77^{Gag}				
Number	Sequence	Expt 1	Expt 2	Expt 3	Mean	SD	Expt 1	Expt 2	Expt 3	Mean	SD
193	G	0.000	0.000	0.000	0.000	0.000	0.065	0.000	0.040	0.035	0.033
194	U	0.200	0.255	0.260	0.238	0.033	0.160	0.060	0.115	0.112	0.050
195	G	0.530	0.620	0.610	0.587	0.049	0.595	0.375	0.560	0.510	0.118
196	U	0.430	0.420	0.470	0.440	0.026	0.325	0.360	0.385	0.357	0.030
197	U	0.690	0.500	0.570	0.587	0.096	0.535	0.450	0.440	0.475	0.052
198	U	0.205	0.145	0.145	0.165	0.035	0.370	0.275	0.230	0.292	0.071
199	G	0.000	0.000	0.000	0.000	0.000	0.145	0.000	0.020	0.055	0.079
200	U	0.000	0.000	0.000	0.000	0.000	0.075	0.010	0.000	0.028	0.041
201	C	0.000	0.000	0.000	0.000	0.000	0.000	0.000	0.000	0.000	0.000
202	U	0.000	0.055	0.075	0.043	0.039	0.080	0.075	0.270	0.142	0.111
203	U	0.290	0.190	0.170	0.217	0.064	0.270	0.180	0.225	0.225	0.045
204	G	0.000	0.060	0.090	0.050	0.046	0.235	0.140	0.120	0.165	0.061
205	U	0.120	0.125	0.240	0.162	0.068	0.050	0.190	0.090	0.110	0.072
206	A	0.125	0.070	0.030	0.075	0.048	0.225	0.150	0.245	0.207	0.050
207	U	0.170	0.220	0.200	0.197	0.025	0.245	0.150	0.125	0.173	0.063
208	U	0.230	0.205	0.230	0.222	0.014	0.255	0.155	0.230	0.213	0.052
209	G	0.530	0.735	0.545	0.603	0.114	0.660	0.625	0.690	0.658	0.033
210	U	0.135	0.130	0.110	0.125	0.013	0.110	0.065	0.180	0.118	0.058
211	C	0.000	0.000	0.000	0.000	0.000	0.000	0.000	0.000	0.000	0.000
212	U	0.000	0.000	0.000	0.000	0.000	0.000	0.000	0.000	0.000	0.000
213	C	0.000	0.000	0.000	0.000	0.000	0.000	0.000	0.000	0.000	0.000
214	U	0.000	0.000	0.000	0.000	0.000	0.000	0.000	0.000	0.000	0.000
215	U	0.000	0.000	0.000	0.000	0.000	0.000	0.000	0.000	0.000	0.000
216	U	0.000	0.070	0.000	0.023	0.040	0.065	0.000	0.000	0.022	0.038
217	C	0.000	0.000	0.000	0.000	0.000	0.000	0.000	0.000	0.000	0.000
218	U	0.280	0.230	0.160	0.223	0.060	0.000	0.175	0.160	0.112	0.097
219	U	0.360	0.520	0.395	0.425	0.084	0.170	0.360	0.290	0.273	0.096
220	G	0.570	0.615	0.520	0.568	0.048	0.345	0.455	0.405	0.402	0.055
221	U	0.215	0.165	0.135	0.172	0.040	0.070	0.180	0.115	0.122	0.055
222	C	0.090	0.060	0.060	0.070	0.017	0.130	0.085	0.050	0.088	0.040
223	U	0.200	0.240	0.180	0.207	0.031	0.210	0.155	0.110	0.158	0.050
224	G	0.560	0.630	0.530	0.573	0.051	0.395	0.390	0.290	0.358	0.059
225	G	0.060	0.045	0.070	0.058	0.013	0.220	0.020	0.070	0.103	0.104
226	C	0.000	0.000	0.000	0.000	0.000	0.000	0.000	0.000	0.000	0.000
227	U	0.210	0.400	0.290	0.300	0.095	0.240	0.330	0.220	0.263	0.059
228	A	0.445	0.510	0.485	0.480	0.033	0.410	0.435	0.535	0.460	0.066
229	U	0.245	0.295	0.245	0.262	0.029	0.000	0.020	0.000	0.007	0.012
230	C	0.000	0.000	0.070	0.023	0.040	0.000	0.000	0.000	0.000	0.000
231	A	0.730	0.680	0.710	0.707	0.025	0.200	0.420	0.350	0.323	0.112
232	U	0.460	0.485	0.325	0.423	0.086	0.000	0.000	0.000	0.000	0.000
233	C	0.010	0.000	0.125	0.045	0.069	0.000	0.000	0.000	0.000	0.000
234	A	0.445	0.715	0.665	0.608	0.144	0.080	0.250	0.200	0.177	0.087
235	C	0.000	0.000	0.000	0.000	0.000	0.000	0.000	0.000	0.000	0.000
236	A	0.705	0.735	0.965	0.802	0.142	0.700	0.575	0.570	0.615	0.074
237	A	1.160	1.285	1.255	1.233	0.065	1.170	1.105	0.860	1.045	0.163
238	G	0.680	0.715	0.615	0.670	0.051	0.495	0.385	0.380	0.420	0.065
239	A	0.220	0.255	0.200	0.225	0.028	0.100	0.190	0.165	0.152	0.046
240	G	0.060	0.095	0.035	0.063	0.030	0.030	0.055	0.090	0.058	0.030

hSHAPE Reactivities from 3 independent experiments (SP106*i*)											
Nucleotides		In the Absence of Pr77^{Gag}					In the Presence of Pr77^{Gag}				
**Number**	**Sequence**	**Expt 1**	**Expt 2**	**Expt 3**	**Mean**	**SD**	**Expt 1**	**Expt 2**	**Expt 3**	**Mean**	**SD**
241	C	0.000	0.055	0.000	0.018	0.032	0.000	0.020	0.000	0.007	0.012
242	G	0.000	0.000	0.000	0.000	0.000	0.130	0.000	0.000	0.043	0.075
243	G	0.000	0.000	0.000	0.000	0.000	0.145	0.050	0.360	0.185	0.159
244	A	0.365	0.365	0.365	0.365	0.000	0.420	0.325	0.360	0.368	0.048
245	A	0.650	0.740	0.690	0.693	0.045	0.645	0.615	0.535	0.598	0.057
246	C	0.290	0.250	0.240	0.260	0.026	0.195	0.260	0.280	0.245	0.044
247	G	0.000	0.000	0.000	0.000	0.000	0.000	0.000	0.350	0.117	0.202
248	G	0.000	0.000	0.000	0.000	0.000	0.000	0.000	0.660	0.220	0.381
249	A	3.230	3.695	3.250	3.392	0.263	3.330	3.990	3.580	3.633	0.333
250	C	0.000	0.000	0.000	0.000	0.000	0.120	0.050	0.120	0.097	0.040
251	U	0.000	0.120	0.050	0.057	0.060	0.000	0.000	0.000	0.000	0.000
252	C	0.020	0.160	0.080	0.087	0.070	0.000	0.065	0.000	0.022	0.038
253	A	0.110	0.135	0.115	0.120	0.013	0.095	0.095	0.065	0.085	0.017
254	C	0.000	0.000	0.000	0.000	0.000	0.000	0.000	0.000	0.000	0.000
255	C	0.000	0.000	0.000	0.000	0.000	0.000	0.000	0.000	0.000	0.000
256	A	0.475	0.510	0.480	0.488	0.019	0.130	0.320	0.460	0.303	0.166
257	U	0.920	1.015	0.960	0.965	0.048	0.840	1.200	0.810	0.950	0.217
258	A	1.085	1.155	1.095	1.112	0.038	1.070	1.290	1.195	1.185	0.110
259	G	0.250	0.330	0.190	0.257	0.070	0.130	0.200	0.120	0.150	0.044
260	G	0.175	0.195	0.195	0.188	0.012	0.145	0.175	0.080	0.133	0.049
261	G	0.000	0.000	0.005	0.002	0.003	0.005	0.060	0.050	0.038	0.029
262	A	0.305	0.395	0.360	0.353	0.045	0.260	0.265	0.270	0.265	0.005
263	G	0.395	0.505	0.445	0.448	0.055	0.270	0.285	0.185	0.247	0.054
264	C	0.220	0.200	0.195	0.205	0.013	0.185	0.230	0.150	0.188	0.040
265	U	1.345	1.420	1.150	1.305	0.139	0.925	0.695	0.555	0.725	0.187
266	G	1.530	1.510	1.545	1.528	0.018	1.100	0.915	0.915	0.977	0.107
267	C	0.000	0.000	0.000	0.000	0.000	0.000	0.000	0.000	0.000	0.000
268	A	1.100	1.190	0.825	1.038	0.190	0.705	0.610	1.175	0.830	0.303
269	G	0.000	0.000	0.000	0.000	0.000	0.830	0.490	0.740	0.687	0.176
270	U	0.000	0.000	0.000	0.000	0.000	0.015	0.060	0.100	0.058	0.043
271	C	0.000	0.000	0.000	0.000	0.000	0.000	0.000	0.000	0.000	0.000
272	C	0.000	0.000	0.000	0.000	0.000	0.000	0.000	0.000	0.000	0.000
273	C	0.000	0.000	0.000	0.000	0.000	0.000	0.000	0.000	0.000	0.000
274	G	0.000	0.000	0.000	0.000	0.000	0.000	0.000	0.000	0.000	0.000
275	C	0.000	0.000	0.000	0.000	0.000	0.000	0.000	0.000	0.000	0.000
276	C	0.000	0.000	0.000	0.000	0.000	0.000	0.000	0.000	0.000	0.000
277	U	0.045	0.070	0.090	0.068	0.023	0.025	0.040	0.105	0.057	0.043
278	A	0.105	0.140	0.125	0.123	0.018	0.115	0.060	0.010	0.062	0.053
279	C	0.180	0.255	0.235	0.223	0.039	0.235	0.180	0.225	0.213	0.029
280	G	0.355	0.430	0.375	0.387	0.039	0.285	0.450	0.415	0.383	0.087
281	G	1.445	1.810	1.535	1.597	0.190	1.405	1.440	1.425	1.423	0.018
282	A	2.355	2.165	2.565	2.362	0.200	2.380	2.545	2.110	2.345	0.220
283	G	1.075	1.260	1.095	1.143	0.102	0.930	0.855	0.820	0.868	0.056
284	A	2.200	2.310	2.345	2.285	0.076	2.150	2.170	2.375	2.232	0.125
285	A	1.945	1.850	2.160	1.985	0.159	2.050	2.225	1.910	2.062	0.158
286	G	1.485	1.940	1.575	1.667	0.241	1.600	1.490	1.465	1.518	0.072
287	A	2.540	2.405	2.710	2.552	0.153	3.020	2.910	3.070	3.000	0.082
288	G	1.085	1.530	1.275	1.297	0.223	1.280	1.390	0.795	1.155	0.317

hSHAPE Reactivities from 3 independent experiments (SP106*i*)											
**Nucleotides**		**In the Absence of Pr77^{Gag}**					**In the Presence of Pr77^{Gag}**				
**Number**	**Sequence**	**Expt 1**	**Expt 2**	**Expt 3**	**Mean**	**SD**	**Expt 1**	**Expt 2**	**Expt 3**	**Mean**	**SD**
289	G	0.000	0.000	0.000	0.000	0.000	0.000	0.000	0.050	0.017	0.029
290	U	0.275	0.200	0.065	0.180	0.106	0.000	0.000	0.165	0.055	0.095
291	A	0.245	0.080	0.190	0.172	0.084	0.110	0.285	0.220	0.205	0.088
292	G	0.000	0.000	0.000	0.000	0.000	0.000	0.000	0.375	0.125	0.217
293	G	0.000	0.000	0.000	0.000	0.000	0.000	0.000	0.005	0.002	0.003
294	U	0.280	0.740	0.000	0.340	0.374	0.520	0.265	0.395	0.393	0.128
295	U	0.485	0.000	0.210	0.232	0.243	0.220	0.570	0.780	0.523	0.283
296	A	0.985	1.165	1.095	1.082	0.091	1.310	1.300	1.380	1.330	0.044
297	C	0.960	0.900	0.820	0.893	0.070	0.830	1.450	1.070	1.117	0.313
298	G	0.285	0.105	0.050	0.147	0.123	0.090	0.070	0.030	0.063	0.031
299	G	0.000	0.005	0.000	0.002	0.003	0.150	0.100	0.050	0.100	0.050
300	U	0.000	0.000	0.000	0.000	0.000	0.000	0.000	0.020	0.007	0.012
301	G	0.000	0.000	0.000	0.000	0.000	0.000	0.000	0.050	0.017	0.029
302	A	0.055	0.070	0.000	0.042	0.037	0.050	0.150	0.030	0.077	0.064
303	G	0.000	0.000	0.000	0.000	0.000	0.010	0.090	0.030	0.043	0.042
304	C	0.000	0.000	0.000	0.000	0.000	0.000	0.000	0.000	0.000	0.000
305	C	0.000	0.240	0.160	0.133	0.122	0.000	0.000	0.000	0.000	0.000
306	A	0.620	0.580	0.595	0.598	0.020	0.340	0.780	0.740	0.620	0.243
307	U	0.755	0.770	0.955	0.827	0.111	0.820	1.020	0.790	0.877	0.125
308	U	1.340	1.240	1.260	1.280	0.053	1.210	1.430	1.220	1.287	0.124
309	G	0.860	0.750	0.670	0.760	0.095	0.550	0.510	0.550	0.537	0.023
310	G	1.030	0.910	0.910	0.950	0.069	0.700	0.730	0.800	0.743	0.051
311	A	1.000	0.850	0.920	0.923	0.075	0.660	0.730	0.750	0.713	0.047
312	A	1.050	0.880	0.960	0.963	0.085	0.790	0.920	0.780	0.830	0.078
313	A	0.610	0.500	0.550	0.553	0.055	0.590	0.600	0.460	0.550	0.078
314	U → G	0.010	0.000	0.000	0.003	0.006	0.000	0.010	0.030	0.013	0.015
315	G	0.030	0.000	0.000	0.010	0.017	0.000	0.120	0.140	0.087	0.076
316	G → C	0.000	0.000	0.000	0.000	0.000	0.000	0.040	0.000	0.013	0.023
317	G	0.000	0.000	0.000	0.000	0.000	0.000	0.000	0.000	0.000	0.000
318	G	0.000	0.000	0.000	0.000	0.000	0.000	0.000	0.000	0.000	0.000
319	G	0.000	0.000	0.000	0.000	0.000	0.000	0.000	0.000	0.000	0.000
320	U	0.000	0.220	0.000	0.073	0.127	0.000	0.000	0.890	0.297	0.514
321	C	0.000	0.000	0.000	0.000	0.000	0.000	0.000	0.100	0.033	0.058
322	U	0.410	0.430	0.420	0.420	0.010	0.140	0.400	0.450	0.330	0.166
323	C	0.130	0.180	0.140	0.150	0.026	0.050	0.010	0.160	0.073	0.078
324	G	0.240	0.260	0.180	0.227	0.042	0.100	0.000	0.250	0.117	0.126
325	G	0.000	0.000	0.000	0.000	0.000	0.000	0.000	0.200	0.067	0.115
326	G	0.000	0.000	0.000	0.000	0.000	0.000	0.000	0.000	0.000	0.000
327	C	0.000	0.000	0.000	0.000	0.000	0.000	0.000	0.050	0.017	0.029
328	U	0.000	0.000	0.000	0.000	0.000	0.000	0.000	0.220	0.073	0.127
329	C	0.000	0.000	0.000	0.000	0.000	0.000	0.040	0.000	0.013	0.023
330	A	0.830	0.930	0.920	0.893	0.055	0.690	0.940	0.920	0.850	0.139
331	A	1.410	1.350	1.460	1.407	0.055	1.250	1.460	1.220	1.310	0.131
332	A	1.310	1.300	1.390	1.333	0.049	1.170	1.420	1.180	1.257	0.142
333	A	0.920	0.900	0.970	0.930	0.036	0.790	0.830	0.720	0.780	0.056
334	G	0.290	0.300	0.250	0.280	0.026	0.090	0.050	0.100	0.080	0.026
335	G	0.000	0.020	0.000	0.007	0.012	0.000	0.000	0.020	0.007	0.012
336	G	0.000	0.000	0.000	0.000	0.000	0.000	0.000	0.090	0.030	0.052

hSHAPE Reactivities from 3 independent experiments (SP106*i*)											
Nucleotides		In the Absence of Pr77^{Gag}					In the Presence of Pr77^{Gag}				
Number	Sequence	Expt 1	Expt 2	Expt 3	Mean	SD	Expt 1	Expt 2	Expt 3	Mean	SD
337	C	0.000	0.110	0.070	0.060	0.056	0.000	0.080	0.200	0.093	0.101
338	A	0.000	0.020	0.010	0.010	0.010	0.000	0.000	0.180	0.060	0.104
339	G	0.000	0.000	0.000	0.000	0.000	0.000	0.000	0.310	0.103	0.179
340	A	0.040	0.120	0.120	0.093	0.046	0.040	0.080	0.160	0.093	0.061
341	A	0.210	0.290	0.310	0.270	0.053	0.260	0.330	0.300	0.297	0.035
342	A	0.120	0.180	0.210	0.170	0.046	0.180	0.180	0.150	0.170	0.017
343	C	0.000	0.020	0.050	0.023	0.025	0.000	0.030	0.140	0.057	0.074
344	U	0.720	0.810	0.920	0.817	0.100	1.000	1.430	1.270	1.233	0.217
345	C	0.380	0.420	0.560	0.453	0.095	0.530	0.870	1.010	0.803	0.247
346	U	0.600	0.580	0.630	0.603	0.025	0.530	0.880	0.770	0.727	0.179
347	U	1.040	1.010	1.110	1.053	0.051	1.000	1.540	1.420	1.320	0.284
348	U	0.780	0.810	0.910	0.833	0.068	0.980	1.370	1.160	1.170	0.195
349	G	0.000	0.050	0.100	0.050	0.050	0.070	0.000	0.230	0.100	0.118
350	U	0.410	0.430	0.490	0.443	0.042	0.290	0.410	0.440	0.380	0.079
351	U	0.520	0.520	0.590	0.543	0.040	0.540	0.530	0.480	0.517	0.032
352	U	0.000	0.000	0.000	0.000	0.000	0.030	0.000	0.000	0.010	0.017
353	C	0.000	0.000	0.000	0.000	0.000	0.000	0.000	0.000	0.000	0.000
354	U	0.460	0.690	0.870	0.673	0.206	1.270	1.060	1.280	1.203	0.124
355	G	2.590	1.600	3.120	2.437	0.772	2.480	3.940	3.110	3.177	0.732
356	U	0.100	0.180	0.150	0.143	0.040	0.100	0.210	0.350	0.220	0.125
357	U	0.270	0.270	0.250	0.263	0.012	0.300	0.350	0.390	0.347	0.045
358	U	0.280	0.310	0.320	0.303	0.021	0.230	0.190	0.320	0.247	0.067
359	U	0.260	0.330	0.310	0.300	0.036	0.030	0.000	0.300	0.110	0.165
360	A	0.450	0.480	0.490	0.473	0.021	0.470	0.720	0.480	0.557	0.142
361	C	0.080	0.770	0.120	0.323	0.387	0.000	0.570	0.000	0.190	0.329
362	A	0.270	0.300	0.310	0.293	0.021	0.180	0.290	0.290	0.253	0.064
363	A	0.100	0.120	0.120	0.113	0.012	0.070	0.090	0.120	0.093	0.025
364	A	0.000	0.020	0.030	0.017	0.015	0.020	0.020	0.020	0.020	0.000
365	G	0.000	0.000	0.000	0.000	0.000	0.000	0.000	0.000	0.000	0.000
366	G	0.000	0.000	0.000	0.000	0.000	0.000	0.010	0.000	0.003	0.006
367	C	0.000	0.000	0.000	0.000	0.000	0.000	0.000	0.000	0.000	0.000
368	U	0.000	0.000	0.000	0.000	0.000	0.000	0.000	0.000	0.000	0.000
369	C	0.000	0.000	0.000	0.000	0.000	0.000	0.000	0.000	0.000	0.000
370	C	0.000	0.000	0.000	0.000	0.000	0.000	0.000	0.010	0.003	0.006
371	U	0.000	0.000	0.000	0.000	0.000	0.000	0.000	0.010	0.003	0.006
372	C	0.000	0.030	0.040	0.023	0.021	0.000	0.020	0.020	0.013	0.012
373	U	0.270	0.390	0.420	0.360	0.079	0.100	0.350	0.300	0.250	0.132
374	C	1.380	1.660	1.870	1.637	0.246	2.340	2.390	1.730	2.153	0.367
375	A	3.820	2.810	3.630	3.420	0.537	3.980	3.790	4.400	4.057	0.312
376	G	2.140	1.930	2.510	2.193	0.294	2.730	3.670	2.530	2.977	0.609
377	A	2.570	2.140	2.800	2.503	0.335	3.060	2.520	2.680	2.753	0.277
378	G	0.000	0.000	0.000	0.000	0.000	0.000	0.000	0.010	0.003	0.006
379	A	0.000	0.080	0.000	0.027	0.046	0.000	0.000	0.000	0.000	0.000
380	G	0.000	0.170	0.000	0.057	0.098	0.000	0.000	0.000	0.000	0.000
381	G	0.000	0.000	0.000	0.000	0.000	0.000	0.000	0.050	0.017	0.029
382	G	0.000	0.000	0.000	0.000	0.000	0.000	0.000	0.230	0.077	0.133
383	G	0.000	0.000	0.000	0.000	0.000	0.000	0.000	0.000	0.000	0.000
384	U	0.000	0.000	0.390	0.130	0.225	0.000	0.000	0.000	0.000	0.000

hSHAPE Reactivities from 3 independent experiments (SP106*i*)											
Nucleotides		In the Absence of $Pr77^{Gag}$					In the Presence of $Pr77^{Gag}$				
Number	Sequence	Expt 1	Expt 2	Expt 3	Mean	SD	Expt 1	Expt 2	Expt 3	Mean	SD
385	C	0.000	0.000	2.560	0.853	1.478	0.000	0.000	0.340	0.113	0.196
386	U	0.000	0.420	0.000	0.140	0.242	0.000	0.000	0.060	0.020	0.035
387	U	0.830	1.460	0.830	1.040	0.364	0.000	0.640	0.250	0.297	0.323
388	C	0.350	1.890	0.710	0.983	0.806	0.000	1.060	0.000	0.353	0.612
389	A	1.350	1.220	1.170	1.247	0.093	0.830	1.590	1.250	1.223	0.381
390	U	0.140	0.100	0.100	0.113	0.023	0.140	0.130	0.100	0.123	0.021
391	G	0.020	0.000	0.000	0.007	0.012	0.050	0.000	0.000	0.017	0.029
392	U	0.000	0.020	0.060	0.027	0.031	0.050	0.000	0.080	0.043	0.040
393	G	0.000	0.020	0.040	0.020	0.020	0.000	0.010	0.090	0.033	0.049
394	A	0.050	0.110	0.130	0.097	0.042	0.040	0.120	0.190	0.117	0.075
395	A	0.080	0.140	0.220	0.147	0.070	0.150	0.240	0.290	0.227	0.071
396	A	0.560	0.450	0.670	0.560	0.110	0.570	0.910	0.700	0.727	0.172
397	G	0.750	0.730	0.830	0.770	0.053	0.890	1.240	0.920	1.017	0.194
398	A	0.140	0.130	0.180	0.150	0.026	0.280	0.350	0.190	0.273	0.080
399	G	0.000	0.000	0.080	0.027	0.046	0.370	0.060	0.200	0.210	0.155
400	A	0.200	0.180	0.280	0.220	0.053	0.380	0.180	0.120	0.227	0.136
401	G	0.140	0.000	0.250	0.130	0.125	0.410	0.000	0.100	0.170	0.214
402	U	0.610	0.840	1.000	0.817	0.196	0.090	0.000	0.630	0.240	0.341
403	A	0.970	0.810	0.920	0.900	0.082	1.060	0.840	0.800	0.900	0.140
404	G	0.430	0.250	0.320	0.333	0.091	0.370	0.290	0.220	0.293	0.075
405	U	1.050	0.560	0.560	0.723	0.283	0.160	0.340	0.120	0.207	0.117
406	G	0.000	0.350	0.000	0.117	0.202	0.340	0.030	0.140	0.170	0.157
407	C	0.280	0.150	0.000	0.143	0.140	0.000	0.000	0.000	0.000	0.000
408	A	0.450	0.350	0.140	0.313	0.158	0.000	0.570	0.610	0.393	0.341
409	A	0.310	0.360	0.410	0.360	0.050	0.370	0.690	0.530	0.530	0.160
410	U	0.250	0.290	0.480	0.340	0.123	0.000	0.000	0.600	0.200	0.346
411	A	0.100	0.110	0.340	0.183	0.136	0.330	0.590	0.580	0.500	0.147
412	G	0.350	0.320	0.300	0.323	0.025	0.260	0.360	0.430	0.350	0.085
413	A	0.480	0.380	0.390	0.417	0.055	0.430	0.660	0.580	0.557	0.117
414	A	0.480	0.340	0.430	0.417	0.071	0.520	0.640	0.590	0.583	0.060
415	U	0.070	0.200	0.380	0.217	0.156	0.490	0.600	0.570	0.553	0.057
416	U	0.370	0.320	0.210	0.300	0.082	0.000	0.480	0.440	0.307	0.266
417	U	0.300	0.500	0.510	0.437	0.118	0.220	0.560	0.950	0.577	0.365
418	U	0.500	0.630	0.520	0.550	0.070	0.000	0.000	0.590	0.197	0.341
419	A	0.500	0.200	0.700	0.467	0.252	0.730	0.930	0.930	0.863	0.115
420	U	0.500	0.620	0.140	0.420	0.250	0.350	0.000	0.350	0.233	0.202
421	C	0.270	0.590	0.290	0.383	0.179	0.000	0.000	0.000	0.000	0.000
422	A	0.270	0.400	0.750	0.473	0.248	0.990	1.040	1.450	1.160	0.252
423	G	0.100	0.280	0.350	0.243	0.129	0.460	0.600	0.650	0.570	0.098
424	U	0.080	0.100	0.180	0.120	0.053	0.440	0.450	0.490	0.460	0.026
425	U	0.290	0.140	0.000	0.143	0.145	0.100	0.260	0.280	0.213	0.099
426	U	0.170	0.250	0.390	0.270	0.111	-999	-999	-999	-999	0.000
427	C	-999	-999	-999	-999	0.000	-999	-999	-999	-999	0.000
428	U	-999	-999	-999	-999	0.000	-999	-999	-999	-999	0.000
429	A	-999	-999	-999	-999	0.000	-999	-999	-999	-999	0.000
430	A	-999	-999	-999	-999	0.000	-999	-999	-999	-999	0.000
431	U	-999	-999	-999	-999	0.000	-999	-999	-999	-999	0.000
432	A	-999	-999	-999	-999	0.000	-999	-999	-999	-999	0.000

hSHAPE Reactivities from 3 independent experiments (SP107*i*)											
Nucleotides		In the Absence of Pr77^{Gag}					In the Presence of Pr77^{Gag}				
Number	Sequence	Expt 1	Expt 2	Expt 3	Mean	SD	Expt 1	Expt 2	Expt 3	Mean	SD
1	G	-999	-999	-999	-999	0.000	-999	-999	-999	-999	0.000
2	C	-999	-999	-999	-999	0.000	-999	-999	-999	-999	0.000
3	A	-999	-999	-999	-999	0.000	-999	-999	-999	-999	0.000
4	A	-999	-999	-999	-999	0.000	-999	-999	-999	-999	0.000
5	C	-999	-999	-999	-999	0.000	-999	-999	-999	-999	0.000
6	A	-999	-999	-999	-999	0.000	-999	-999	-999	-999	0.000
7	G	-999	-999	-999	-999	0.000	-999	-999	-999	-999	0.000
8	U	-999	-999	-999	-999	0.000	-999	-999	-999	-999	0.000
9	C	-999	-999	-999	-999	0.000	-999	-999	-999	-999	0.000
10	C	-999	-999	-999	-999	0.000	-999	-999	-999	-999	0.000
11	U	-999	-999	-999	-999	0.000	-999	-999	-999	-999	0.000
12	A	-999	-999	-999	-999	0.000	-999	-999	-999	-999	0.000
13	A	-999	-999	-999	-999	0.000	-999	-999	-999	-999	0.000
14	U	-999	-999	-999	-999	0.000	-999	-999	-999	-999	0.000
15	A	-999	-999	-999	-999	0.000	1.685	1.895	1.600	1.727	0.152
16	U	-999	-999	-999	-999	0.000	1.505	1.600	1.495	1.533	0.058
17	U	0.635	0.780	0.835	0.750	0.103	0.725	0.810	0.510	0.682	0.155
18	C	0.075	0.100	0.000	0.058	0.052	0.000	0.000	0.000	0.000	0.000
19	A	0.235	0.285	0.260	0.260	0.025	0.240	0.250	0.020	0.170	0.130
20	C	0.115	0.110	0.095	0.107	0.010	0.000	0.035	0.065	0.033	0.033
21	G	0.350	0.410	0.400	0.387	0.032	0.465	0.595	0.500	0.520	0.067
22	U	1.575	1.520	1.805	1.633	0.151	2.265	2.280	2.090	2.212	0.106
23	C	0.305	0.360	0.310	0.325	0.030	0.555	0.615	0.300	0.490	0.167
24	U	2.300	2.110	2.370	2.260	0.135	2.900	3.010	2.515	2.808	0.260
25	C	0.545	0.625	0.510	0.560	0.059	0.845	0.900	0.640	0.795	0.137
26	G	0.205	0.245	0.075	0.175	0.089	0.200	0.150	0.250	0.200	0.050
27	U	0.060	0.020	0.040	0.040	0.020	0.100	0.050	0.110	0.087	0.032
28	G	0.000	0.000	0.000	0.000	0.000	0.000	0.000	0.000	0.000	0.000
29	U	0.245	0.340	0.315	0.300	0.049	0.130	0.290	0.240	0.220	0.082
30	G	0.200	0.215	0.200	0.205	0.009	0.160	0.135	0.165	0.153	0.016
31	U	0.060	0.070	0.080	0.070	0.010	0.020	0.020	0.110	0.050	0.052
32	U	0.300	0.305	0.300	0.302	0.003	0.285	0.320	0.345	0.317	0.030
33	U	0.240	0.300	0.210	0.250	0.046	0.175	0.275	0.325	0.258	0.076
34	G	0.280	0.300	0.175	0.252	0.067	0.390	0.325	0.310	0.342	0.043
35	U	1.080	1.185	1.000	1.088	0.093	1.165	1.375	1.480	1.340	0.160
36	G	0.425	0.425	0.290	0.380	0.078	0.455	0.560	0.445	0.487	0.064
37	U	0.090	0.075	0.050	0.072	0.020	0.040	0.010	0.045	0.032	0.019
38	C	0.000	0.000	0.000	0.000	0.000	0.000	0.000	0.000	0.000	0.000
39	U	0.000	0.000	0.000	0.000	0.000	0.000	0.000	0.030	0.010	0.017
40	G	0.000	0.000	0.000	0.000	0.000	0.000	0.000	0.000	0.000	0.000
41	U	0.000	0.000	0.000	0.000	0.000	0.000	0.000	0.000	0.000	0.000
42	U	0.135	0.145	0.240	0.173	0.058	0.145	0.110	0.290	0.182	0.095
43	C	0.360	0.550	0.675	0.528	0.159	0.650	0.595	0.545	0.597	0.053
44	G	0.360	0.570	0.740	0.557	0.190	0.340	0.390	0.320	0.350	0.036
45	C	0.000	0.000	0.000	0.000	0.000	0.000	0.000	0.000	0.000	0.000
46	C	0.000	0.000	0.000	0.000	0.000	0.000	0.000	0.000	0.000	0.000
47	A	0.210	0.355	0.380	0.315	0.092	0.000	0.000	0.000	0.000	0.000
48	U	0.655	0.565	0.770	0.663	0.103	0.560	0.970	0.870	0.800	0.214

hSHAPE Reactivities from 3 independent experiments (SP107*i*)											
Nucleotides		In the Absence of Pr77^{Gag}					In the Presence of Pr77^{Gag}				
Number	Sequence	Expt 1	Expt 2	Expt 3	Mean	SD	Expt 1	Expt 2	Expt 3	Mean	SD
49	C	0.105	0.150	0.070	0.108	0.040	0.040	0.095	0.000	0.045	0.048
50	C	0.000	0.000	0.000	0.000	0.000	0.000	0.000	0.000	0.000	0.000
51	C	0.000	0.000	0.000	0.000	0.000	0.000	0.000	0.000	0.000	0.000
52	G	0.845	0.870	0.740	0.818	0.069	0.930	1.240	0.870	1.013	0.199
53	U	0.805	0.825	0.955	0.862	0.081	0.770	1.080	0.755	0.868	0.183
54	C	0.260	0.260	0.160	0.227	0.058	0.160	0.345	0.185	0.230	0.100
55	U	0.155	0.220	0.120	0.165	0.051	0.080	0.275	0.185	0.180	0.098
56	C → G	0.000	0.000	0.000	0.000	0.000	0.040	0.030	0.135	0.068	0.058
57	C	0.000	0.000	0.000	0.000	0.000	0.000	0.000	0.000	0.000	0.000
58	G → C	0.000	0.000	0.000	0.000	0.000	0.000	0.000	0.000	0.000	0.000
59	C	0.000	0.000	0.000	0.000	0.000	0.000	0.000	0.000	0.000	0.000
60	U→ A	0.610	1.050	0.960	0.873	0.232	0.510	0.660	0.525	0.565	0.083
61	C	0.000	0.000	0.000	0.000	0.000	0.000	0.005	0.000	0.002	0.003
62	G	0.000	0.000	0.000	0.000	0.000	0.000	0.000	0.000	0.000	0.000
63	U	0.000	0.000	0.000	0.000	0.000	0.000	0.000	0.000	0.000	0.000
64	C	0.000	0.400	0.000	0.133	0.231	0.000	0.000	0.000	0.000	0.000
65	A	0.200	0.255	0.225	0.227	0.028	0.095	0.245	0.200	0.180	0.077
66	C	0.000	0.000	0.000	0.000	0.000	0.000	0.000	0.000	0.000	0.000
67	U	0.155	0.205	0.210	0.190	0.030	0.110	0.280	0.100	0.163	0.101
68	U	0.320	0.395	0.245	0.320	0.075	0.000	0.110	0.000	0.037	0.064
69	A	0.355	0.425	0.390	0.390	0.035	0.240	0.480	0.200	0.307	0.151
70	U	0.205	0.245	0.120	0.190	0.064	0.160	0.335	0.150	0.215	0.104
71	C	0.000	0.000	0.000	0.000	0.000	0.000	0.020	0.000	0.007	0.012
72	C	0.000	0.000	0.000	0.000	0.000	0.000	0.000	0.000	0.000	0.000
73	U	0.630	0.850	0.595	0.692	0.138	0.505	0.330	0.345	0.393	0.097
74	U	1.510	1.230	1.935	1.558	0.355	1.840	1.485	1.270	1.532	0.288
75	C	0.000	0.000	0.000	0.000	0.000	0.000	0.000	0.000	0.000	0.000
76	A	1.370	1.290	1.365	1.342	0.045	0.900	1.075	0.000	0.658	0.577
77	C	0.285	0.100	0.150	0.178	0.096	0.000	0.040	0.000	0.013	0.023
78	U	1.245	1.080	1.390	1.238	0.155	1.040	1.170	1.050	1.087	0.072
79	U	1.640	1.665	2.050	1.785	0.230	1.880	1.790	1.815	1.828	0.046
80	U	1.080	1.195	1.190	1.155	0.065	0.815	1.220	1.135	1.057	0.214
81	C	0.165	0.170	0.120	0.152	0.028	0.000	0.000	0.000	0.000	0.000
82	C	0.075	0.000	0.000	0.025	0.043	0.000	0.000	0.000	0.000	0.000
83	A	1.560	1.740	1.840	1.713	0.142	2.320	1.975	1.650	1.982	0.335
84	G	0.110	0.185	0.140	0.145	0.038	0.280	0.110	0.040	0.143	0.123
85	A	0.085	0.020	0.020	0.042	0.038	0.005	0.020	0.020	0.015	0.009
86	G	0.000	0.000	0.000	0.000	0.000	0.000	0.000	0.000	0.000	0.000
87	G	0.000	0.000	0.000	0.000	0.000	0.000	0.000	0.000	0.000	0.000
88	G	0.000	0.000	0.000	0.000	0.000	0.000	0.000	0.000	0.000	0.000
89	U	0.000	0.000	0.000	0.000	0.000	0.000	0.000	0.000	0.000	0.000
90	C	0.000	0.000	0.000	0.000	0.000	0.000	0.000	0.000	0.000	0.000
91	C	0.000	0.000	0.000	0.000	0.000	0.000	0.000	0.000	0.000	0.000
92	C	0.000	0.000	0.000	0.000	0.000	0.000	0.000	0.000	0.000	0.000
93	C	0.000	0.000	0.000	0.000	0.000	0.000	0.000	0.000	0.000	0.000
94	C	0.000	0.000	0.000	0.000	0.000	0.000	0.000	0.000	0.000	0.000
95	C	0.000	0.000	0.000	0.000	0.000	0.000	0.000	0.000	0.000	0.000
96	G	0.000	0.000	0.000	0.000	0.000	0.000	0.000	0.090	0.030	0.052

hSHAPE Reactivities from 3 independent experiments (SP107*i*)											
Nucleotides		In the Absence of Pr77^{Gag}					In the Presence of Pr77^{Gag}				
Number	Sequence	Expt 1	Expt 2	Expt 3	Mean	SD	Expt 1	Expt 2	Expt 3	Mean	SD
97	C	0.000	0.000	0.000	0.000	0.000	0.000	0.000	0.000	0.000	0.000
98	A	0.435	0.485	0.530	0.483	0.048	0.165	0.150	0.140	0.152	0.013
99	G	0.745	0.765	0.980	0.830	0.130	0.520	0.515	0.465	0.500	0.030
100	A	1.885	1.915	2.275	2.025	0.217	1.915	1.825	1.810	1.850	0.057
101	C	0.100	0.215	0.190	0.168	0.060	0.325	0.200	0.255	0.260	0.063
102	C	0.000	0.000	0.000	0.000	0.000	0.000	0.000	0.000	0.000	0.000
103	C	0.000	0.000	0.000	0.000	0.000	0.000	0.000	0.000	0.000	0.000
104	C	0.000	0.000	0.000	0.000	0.000	0.000	0.000	0.000	0.000	0.000
105	G	0.000	0.000	0.000	0.000	0.000	0.000	0.000	0.000	0.000	0.000
106	G	0.000	0.000	0.000	0.000	0.000	0.000	0.000	0.000	0.000	0.000
107	U	0.000	0.160	0.000	0.053	0.092	0.000	0.000	0.030	0.010	0.017
108	G	0.000	0.000	0.000	0.000	0.000	0.000	0.000	0.075	0.025	0.043
109	A	0.030	0.070	0.035	0.045	0.022	0.035	0.070	0.080	0.062	0.024
110	C	0.000	0.000	0.000	0.000	0.000	0.000	0.000	0.000	0.000	0.000
111	C	0.000	0.000	0.000	0.000	0.000	0.000	0.000	0.000	0.000	0.000
112	C	0.000	0.000	0.000	0.000	0.000	0.000	0.000	0.000	0.000	0.000
113	U	1.165	1.635	1.030	1.277	0.318	1.890	2.335	1.705	1.977	0.324
114	C	0.240	0.635	0.000	0.292	0.321	0.455	0.785	0.000	0.413	0.394
115	A	2.835	3.465	3.170	3.157	0.315	3.330	3.990	3.275	3.532	0.398
116	G	0.195	0.380	0.080	0.218	0.151	0.590	0.505	0.490	0.528	0.054
117	G	0.000	0.000	0.000	0.000	0.000	0.195	0.125	0.180	0.167	0.037
118	U	0.000	0.000	0.000	0.000	0.000	0.000	0.000	0.000	0.000	0.000
119	C	0.140	0.175	0.140	0.152	0.020	0.000	0.000	0.000	0.000	0.000
120	G	0.405	0.210	0.370	0.328	0.104	0.140	0.185	0.235	0.187	0.048
121	G	0.615	0.450	0.800	0.622	0.175	0.155	0.110	0.430	0.232	0.173
122	C	0.000	0.000	0.000	0.000	0.000	0.000	0.000	0.000	0.000	0.000
123	C	0.000	0.000	0.000	0.000	0.000	0.000	0.000	0.000	0.000	0.000
124	G	0.000	0.000	0.000	0.000	0.000	0.000	0.000	0.000	0.000	0.000
125	A	0.355	0.330	0.325	0.337	0.016	0.220	0.260	0.315	0.265	0.048
126	C	0.000	0.000	0.000	0.000	0.000	0.000	0.000	0.000	0.000	0.000
127	U	0.180	0.000	0.145	0.108	0.095	0.000	0.000	0.000	0.000	0.000
128	G	0.405	0.230	0.400	0.345	0.100	0.160	0.115	0.095	0.123	0.033
129	C	0.000	0.000	0.000	0.000	0.000	0.000	0.000	0.000	0.000	0.000
130	G	0.000	0.000	0.000	0.000	0.000	0.000	0.000	0.000	0.000	0.000
131	G	0.000	0.000	0.000	0.000	0.000	0.000	0.000	0.000	0.000	0.000
132	C	0.000	0.000	0.000	0.000	0.000	0.000	0.000	0.000	0.000	0.000
133	A	0.000	0.000	0.000	0.000	0.000	0.000	0.000	0.000	0.000	0.000
134	G	0.260	0.370	0.580	0.403	0.163	0.000	0.000	0.000	0.000	0.000
135	C	0.000	0.000	0.000	0.000	0.000	0.000	0.000	0.290	0.097	0.167
136	U	0.515	0.270	0.710	0.498	0.220	0.760	0.540	0.880	0.727	0.172
137	G	0.230	0.220	0.445	0.298	0.127	0.080	0.200	0.310	0.197	0.115
138	G	0.000	0.000	0.000	0.000	0.000	0.000	0.000	0.000	0.000	0.000
139	C	0.000	0.000	0.000	0.000	0.000	0.000	0.000	0.000	0.000	0.000
140	G	0.080	0.080	0.075	0.078	0.003	0.000	0.000	0.000	0.000	0.000
141	C	0.000	0.000	0.000	0.000	0.000	0.000	0.000	0.000	0.000	0.000
142	C	0.000	0.000	0.000	0.000	0.000	0.000	0.000	0.000	0.000	0.000
143	C	0.000	0.000	0.000	0.000	0.000	0.000	0.000	0.000	0.000	0.000
144	G	0.200	0.230	0.370	0.267	0.091	0.375	0.125	0.505	0.335	0.193

hSHAPE Reactivities from 3 independent experiments (SP107*i*)											
**Nucleotides**		**In the Absence of Pr77^{Gag}**					**In the Presence of Pr77^{Gag}**				
**Number**	**Sequence**	**Expt 1**	**Expt 2**	**Expt 3**	**Mean**	**SD**	**Expt 1**	**Expt 2**	**Expt 3**	**Mean**	**SD**
145	A	0.750	0.845	0.850	0.815	0.056	1.150	0.890	1.065	1.035	0.133
146	A	0.840	0.955	0.965	0.920	0.069	0.745	0.585	0.590	0.640	0.091
147	C	0.000	0.080	0.000	0.027	0.046	0.000	0.000	0.000	0.000	0.000
148	A	0.630	0.715	0.435	0.593	0.144	0.570	0.540	0.870	0.660	0.182
149	G	0.000	0.000	0.000	0.000	0.000	0.000	0.000	0.000	0.000	0.000
150	G	0.000	0.000	0.000	0.000	0.000	0.000	0.000	0.000	0.000	0.000
151	G	0.000	0.000	0.000	0.000	0.000	0.000	0.000	0.000	0.000	0.000
152	A	0.000	0.000	0.000	0.000	0.000	0.000	0.000	0.000	0.000	0.000
153	C	0.000	0.000	0.000	0.000	0.000	0.000	0.000	0.000	0.000	0.000
154	C	0.000	0.000	0.000	0.000	0.000	0.000	0.000	0.000	0.000	0.000
155	C	0.000	0.000	0.000	0.000	0.000	0.000	0.000	0.000	0.000	0.000
156	U	0.000	0.000	0.000	0.000	0.000	0.000	0.000	0.000	0.000	0.000
157	C	0.140	0.295	0.200	0.212	0.078	0.010	0.160	0.230	0.133	0.112
158	G	0.000	0.000	0.000	0.000	0.000	0.015	0.000	0.000	0.005	0.009
159	G	0.000	0.000	0.000	0.000	0.000	0.000	0.000	0.000	0.000	0.000
160	A	0.000	0.000	0.000	0.000	0.000	0.000	0.000	0.035	0.012	0.020
161	U	0.000	0.000	0.135	0.045	0.078	0.170	0.095	0.070	0.112	0.052
162	A	0.000	0.000	0.000	0.000	0.000	0.005	0.090	0.090	0.062	0.049
163	A	0.070	0.020	0.090	0.060	0.036	0.000	0.000	0.090	0.030	0.052
164	G	0.000	0.000	0.000	0.000	0.000	0.000	0.000	0.000	0.000	0.000
165	U	0.000	0.000	0.000	0.000	0.000	0.000	0.000	0.000	0.000	0.000
166	G	0.000	0.000	0.000	0.000	0.000	0.000	0.000	0.000	0.000	0.000
167	A	0.000	0.000	0.000	0.000	0.000	0.000	0.000	0.000	0.000	0.000
168	C	0.000	0.000	0.000	0.000	0.000	0.000	0.000	0.000	0.000	0.000
169	C	0.000	0.000	0.000	0.000	0.000	0.000	0.000	0.000	0.000	0.000
170	C	0.000	0.000	0.000	0.000	0.000	0.000	0.000	0.000	0.000	0.000
171	U	0.000	0.000	0.000	0.000	0.000	0.020	0.060	0.060	0.047	0.023
172	U	0.305	0.500	0.425	0.410	0.098	0.235	0.280	0.465	0.327	0.122
173	G	0.790	0.925	0.870	0.862	0.068	0.805	0.750	0.985	0.847	0.123
174	U	0.425	0.525	0.530	0.493	0.059	0.580	0.395	0.535	0.503	0.096
175	C	0.000	0.000	0.000	0.000	0.000	0.000	0.000	0.000	0.000	0.000
176	U	0.000	0.000	0.000	0.000	0.000	0.000	0.000	0.000	0.000	0.000
177	C	0.000	0.000	0.000	0.000	0.000	0.000	0.000	0.000	0.000	0.000
178	U	0.000	0.000	0.000	0.000	0.000	0.000	0.000	0.000	0.000	0.000
179	A	0.430	0.450	0.350	0.410	0.053	0.345	0.440	0.100	0.295	0.175
180	U	0.070	0.120	0.085	0.092	0.026	0.115	0.215	0.100	0.143	0.063
181	U	0.055	0.115	0.150	0.107	0.048	0.175	0.165	0.180	0.173	0.008
182	U	0.030	0.095	0.085	0.070	0.035	0.095	0.060	0.100	0.085	0.022
183	C	0.000	0.000	0.000	0.000	0.000	0.000	0.000	0.000	0.000	0.000
184	U	0.000	0.100	0.135	0.078	0.070	0.000	0.000	0.000	0.000	0.000
185	A	0.045	0.075	0.075	0.065	0.017	0.185	0.080	0.205	0.157	0.067
186	C	0.000	0.000	0.000	0.000	0.000	0.000	0.000	0.000	0.000	0.000
187	U	0.610	0.680	1.000	0.763	0.208	0.330	0.340	0.200	0.290	0.078
188	A	0.705	0.775	0.810	0.763	0.053	0.760	0.775	0.950	0.828	0.106
189	U	0.115	0.150	0.120	0.128	0.019	0.140	0.190	0.250	0.193	0.055
190	U	0.000	0.000	0.000	0.000	0.000	0.160	0.120	0.240	0.173	0.061
191	U	0.140	0.145	0.145	0.143	0.003	0.115	0.100	0.150	0.122	0.026
192	G	0.000	0.050	0.080	0.043	0.040	0.000	0.000	0.015	0.005	0.009

hSHAPE Reactivities from 3 independent experiments (SP107*i*)											
Nucleotides		In the Absence of Pr77[Gag]					In the Presence of Pr77[Gag]				
Number	Sequence	Expt 1	Expt 2	Expt 3	Mean	SD	Expt 1	Expt 2	Expt 3	Mean	SD
193	G	0.095	0.010	0.070	0.058	0.044	0.220	0.325	0.350	0.298	0.069
194	U	0.350	0.305	0.360	0.338	0.029	0.120	0.175	0.315	0.203	0.101
195	G	0.675	0.735	0.595	0.668	0.070	0.635	0.570	0.845	0.683	0.144
196	U	0.545	0.425	0.475	0.482	0.060	0.240	0.455	0.575	0.423	0.170
197	U	0.685	0.605	0.575	0.622	0.057	0.435	0.690	0.625	0.583	0.133
198	U	0.355	0.365	0.325	0.348	0.021	0.380	0.450	0.420	0.417	0.035
199	G	0.080	0.055	0.055	0.063	0.014	0.065	0.160	0.165	0.130	0.056
200	U	0.070	0.070	0.120	0.087	0.029	0.140	0.070	0.105	0.105	0.035
201	C	0.000	0.000	0.000	0.000	0.000	0.020	0.000	0.160	0.060	0.087
202	U	0.010	0.075	0.125	0.070	0.058	0.075	0.265	0.340	0.227	0.137
203	U	0.325	0.355	0.385	0.355	0.030	0.395	0.420	0.570	0.462	0.095
204	G	0.060	0.160	0.185	0.135	0.066	0.170	0.235	0.290	0.232	0.060
205	U	0.220	0.360	0.295	0.292	0.070	0.240	0.260	0.160	0.220	0.053
206	A	0.195	0.330	0.270	0.265	0.068	0.335	0.325	0.320	0.327	0.008
207	U	0.230	0.250	0.285	0.255	0.028	0.255	0.250	0.185	0.230	0.039
208	U	0.405	0.325	0.370	0.367	0.040	0.300	0.355	0.385	0.347	0.043
209	G	0.955	0.940	1.015	0.970	0.040	0.995	0.885	1.000	0.960	0.065
210	U	0.140	0.195	0.220	0.185	0.041	0.295	0.230	0.220	0.248	0.041
211	C	0.000	0.000	0.000	0.000	0.000	0.000	0.000	0.000	0.000	0.000
212	U	0.000	0.000	0.000	0.000	0.000	0.000	0.000	0.000	0.000	0.000
213	C	0.000	0.000	0.000	0.000	0.000	0.000	0.000	0.000	0.000	0.000
214	U	0.000	0.000	0.000	0.000	0.000	0.000	0.000	0.000	0.000	0.000
215	U	0.000	0.000	0.000	0.000	0.000	0.075	0.180	0.270	0.175	0.098
216	U	0.150	0.090	0.105	0.115	0.031	0.135	0.145	0.180	0.153	0.024
217	C	0.000	0.000	0.000	0.000	0.000	0.000	0.000	0.000	0.000	0.000
218	U	0.100	0.130	0.095	0.108	0.019	0.130	0.040	0.135	0.102	0.053
219	U	0.475	0.455	0.475	0.468	0.012	0.345	0.345	0.320	0.337	0.014
220	G	0.660	0.685	0.700	0.682	0.020	0.545	0.495	0.505	0.515	0.026
221	U	0.130	0.210	0.170	0.170	0.040	0.195	0.215	0.185	0.198	0.015
222	C	0.105	0.160	0.115	0.127	0.029	0.025	0.075	0.020	0.040	0.030
223	U	0.345	0.315	0.375	0.345	0.030	0.315	0.295	0.345	0.318	0.025
224	G	0.400	0.405	0.515	0.440	0.065	0.295	0.330	0.265	0.297	0.033
225	G	0.000	0.000	0.000	0.000	0.000	0.000	0.000	0.000	0.000	0.000
226	C	0.000	0.000	0.000	0.000	0.000	0.000	0.000	0.000	0.000	0.000
227	U	0.740	0.930	1.025	0.898	0.145	0.430	0.420	0.350	0.400	0.044
228	A	0.805	0.820	1.030	0.885	0.126	0.835	0.820	0.845	0.833	0.013
229	U	0.445	0.470	0.560	0.492	0.060	0.470	0.320	0.150	0.313	0.160
230	C	0.000	0.000	0.000	0.000	0.000	0.000	0.000	0.000	0.000	0.000
231	A	0.715	0.785	0.730	0.743	0.037	0.765	0.680	0.610	0.685	0.078
232	U	0.180	0.325	0.195	0.233	0.080	0.000	0.000	0.000	0.000	0.000
233	C	0.000	0.170	0.000	0.057	0.098	0.000	0.000	0.000	0.000	0.000
234	A	0.480	0.640	0.500	0.540	0.087	0.215	0.058	0.647	0.307	0.305
235	C	0.000	0.000	0.000	0.000	0.000	0.000	0.000	0.000	0.000	0.000
236	A	0.665	0.805	0.665	0.712	0.081	0.255	0.330	0.280	0.288	0.038
237	A	1.045	1.110	1.105	1.087	0.036	0.665	1.295	0.705	0.888	0.353
238	G	0.865	1.080	1.030	0.992	0.113	0.920	1.040	0.720	0.893	0.162
239	A	0.345	0.390	0.395	0.377	0.028	0.380	0.395	0.225	0.333	0.094
240	G	0.155	0.150	0.125	0.143	0.016	0.155	0.100	0.080	0.112	0.039

hSHAPE Reactivities from 3 independent experiments (SP107*i*)											
Nucleotides		In the Absence of Pr77^{Gag}					In the Presence of Pr77^{Gag}				
Number	Sequence	Expt 1	Expt 2	Expt 3	Mean	SD	Expt 1	Expt 2	Expt 3	Mean	SD
241	C	0.170	0.170	0.130	0.157	0.023	0.070	0.035	0.010	0.038	0.030
242	G	0.550	0.535	0.435	0.507	0.063	0.485	0.410	0.385	0.427	0.052
243	G	0.495	0.510	0.535	0.513	0.020	0.630	0.520	0.565	0.572	0.055
244	A	0.530	0.595	0.615	0.580	0.044	0.495	0.440	0.425	0.453	0.037
245	A	0.630	0.710	0.720	0.687	0.049	0.580	0.560	0.505	0.548	0.039
246	C	0.195	0.330	0.235	0.253	0.069	0.285	0.345	0.170	0.267	0.089
247	G	0.000	0.000	0.000	0.000	0.000	0.000	0.000	0.000	0.000	0.000
248	G	0.000	0.000	0.000	0.000	0.000	0.080	0.205	0.000	0.095	0.103
249	A	3.525	3.825	3.860	3.737	0.184	4.390	4.060	3.805	4.085	0.293
250	C	0.000	0.000	0.000	0.000	0.000	0.000	0.275	0.000	0.092	0.159
251	U	0.000	0.000	0.000	0.000	0.000	0.000	0.010	0.000	0.003	0.006
252	C	0.045	0.095	0.020	0.053	0.038	0.000	0.090	0.000	0.030	0.052
253	A	0.305	0.340	0.360	0.335	0.028	0.258	0.161	0.055	0.158	0.102
254	C	0.000	0.000	0.000	0.000	0.000	0.000	0.000	0.000	0.000	0.000
255	C	0.000	0.000	0.000	0.000	0.000	0.000	0.000	0.000	0.000	0.000
256	A	0.130	0.290	0.295	0.238	0.094	0.000	0.000	0.000	0.000	0.000
257	U	0.830	0.800	0.825	0.818	0.016	0.825	0.860	0.620	0.768	0.130
258	A	0.920	1.320	1.260	1.167	0.216	1.215	1.460	1.160	1.278	0.160
259	G	0.120	0.310	0.255	0.228	0.098	0.205	0.215	0.150	0.190	0.035
260	G	0.000	0.105	0.020	0.042	0.056	0.040	0.075	0.190	0.102	0.078
261	G	0.000	0.155	0.000	0.052	0.089	0.080	0.070	0.050	0.067	0.015
262	A	0.585	0.600	0.750	0.645	0.091	0.505	0.530	0.440	0.492	0.046
263	G	0.630	0.735	0.730	0.698	0.059	0.430	0.430	0.485	0.448	0.032
264	C	0.565	0.460	0.410	0.478	0.079	0.304	0.190	0.210	0.235	0.061
265	U	2.050	1.845	2.150	2.015	0.155	1.185	1.210	0.930	1.108	0.155
266	G	2.035	1.730	1.910	1.892	0.153	0.960	0.985	1.065	1.003	0.055
267	C	0.000	0.000	0.000	0.000	0.000	0.000	0.000	0.000	0.000	0.000
268	A	2.130	1.900	1.425	1.818	0.360	1.100	1.410	1.230	1.247	0.156
269	G	0.000	0.000	0.000	0.000	0.000	0.255	0.050	0.000	0.102	0.135
270	U	0.065	0.000	0.000	0.022	0.038	0.110	0.180	0.220	0.170	0.056
271	C	0.000	0.000	0.000	0.000	0.000	0.000	0.000	0.000	0.000	0.000
272	C	0.000	0.000	0.000	0.000	0.000	0.000	0.000	0.000	0.000	0.000
273	C	0.000	0.000	0.000	0.000	0.000	0.000	0.000	0.000	0.000	0.000
274	G	0.000	0.000	0.000	0.000	0.000	0.000	0.095	0.045	0.047	0.048
275	C	0.000	0.000	0.000	0.000	0.000	0.000	0.000	0.000	0.000	0.000
276	C	0.000	0.000	0.000	0.000	0.000	0.000	0.000	0.000	0.000	0.000
277	U	0.040	0.115	0.140	0.098	0.052	0.010	0.140	0.090	0.080	0.066
278	A	0.060	0.155	0.140	0.118	0.051	0.080	0.010	0.030	0.040	0.036
279	C	0.240	0.150	0.260	0.217	0.059	0.250	0.310	0.175	0.245	0.068
280	G	0.480	0.345	0.420	0.415	0.068	0.515	0.360	0.520	0.465	0.091
281	G	1.840	2.255	2.130	2.075	0.213	2.165	2.795	2.105	2.355	0.382
282	A	3.250	3.360	3.870	3.493	0.331	3.755	3.810	3.350	3.638	0.251
283	G	1.550	1.475	1.650	1.558	0.088	1.315	1.740	1.370	1.475	0.231
284	A	3.420	3.240	3.600	3.420	0.180	3.450	3.960	3.760	3.723	0.257
285	A	2.700	2.570	3.000	2.757	0.221	3.155	3.100	3.040	3.098	0.058
286	G	1.930	2.305	2.170	2.135	0.190	2.245	2.575	1.930	2.250	0.323
287	A	3.625	3.815	3.790	3.743	0.103	4.485	3.900	4.045	4.143	0.305
288	G	1.820	1.700	1.995	1.838	0.148	2.705	2.785	2.470	2.653	0.164

hSHAPE Reactivities from 3 independent experiments (SP107*i*)											
Nucleotides		In the Absence of Pr77^{Gag}					In the Presence of Pr77^{Gag}				
Number	Sequence	Expt 1	Expt 2	Expt 3	Mean	SD	Expt 1	Expt 2	Expt 3	Mean	SD
289	G	0.000	0.000	0.000	0.000	0.000	0.000	0.000	0.000	0.000	0.000
290	U	0.000	0.000	0.000	0.000	0.000	0.000	0.000	0.000	0.000	0.000
291	A	0.005	0.050	0.010	0.022	0.025	0.020	0.000	0.000	0.007	0.012
292	G	0.000	0.000	0.000	0.000	0.000	0.110	0.070	0.170	0.117	0.050
293	G	0.000	0.000	0.000	0.000	0.000	0.000	0.000	0.000	0.000	0.000
294	U	0.180	0.290	0.120	0.197	0.086	0.245	0.230	0.070	0.182	0.097
295	U	0.580	0.475	0.540	0.532	0.053	0.520	0.330	0.340	0.397	0.107
296	A	1.380	1.730	1.860	1.657	0.248	1.385	1.380	1.510	1.425	0.074
297	C	0.840	1.040	1.120	1.000	0.144	1.140	1.475	1.500	1.372	0.201
298	G	0.010	0.020	0.100	0.043	0.049	0.000	0.000	0.050	0.017	0.029
299	G	0.000	0.000	0.000	0.000	0.000	0.000	0.000	0.000	0.000	0.000
300	U	0.000	0.000	0.000	0.000	0.000	0.180	0.050	0.310	0.180	0.130
301	G	0.000	0.000	0.000	0.000	0.000	0.060	0.170	0.210	0.147	0.078
302	A	0.000	0.000	0.000	0.000	0.000	0.000	0.000	0.000	0.000	0.000
303	G	0.000	0.000	0.000	0.000	0.000	0.000	0.000	0.000	0.000	0.000
304	C	0.000	0.000	0.000	0.000	0.000	0.000	0.000	0.000	0.000	0.000
305	C	0.000	0.000	0.000	0.000	0.000	0.000	0.000	0.000	0.000	0.000
306	A	0.190	0.420	0.210	0.273	0.127	0.530	0.770	1.090	0.797	0.281
307	U	0.640	0.850	0.930	0.807	0.150	1.240	0.930	0.810	0.993	0.222
308	U	1.240	1.330	1.630	1.400	0.204	1.750	1.590	1.720	1.687	0.085
309	G	1.010	1.190	1.470	1.223	0.232	1.370	1.150	1.070	1.197	0.155
310	G	0.890	1.070	1.300	1.087	0.206	1.220	1.050	1.030	1.100	0.104
311	A	0.660	0.790	0.940	0.797	0.140	0.840	0.700	0.490	0.677	0.176
312	A	0.490	0.520	0.680	0.563	0.102	0.550	0.520	0.400	0.490	0.079
313	A	0.330	0.330	0.450	0.370	0.069	0.370	0.380	0.390	0.380	0.010
314	U	0.320	0.270	0.330	0.307	0.032	0.480	0.410	0.460	0.450	0.036
315	G	0.200	0.010	0.080	0.097	0.096	0.180	0.310	0.120	0.203	0.097
316	G	0.000	0.000	0.000	0.000	0.000	0.340	0.290	0.310	0.313	0.025
317	G	0.000	0.000	0.000	0.000	0.000	0.000	0.000	0.000	0.000	0.000
318	G	0.000	0.000	0.000	0.000	0.000	0.000	0.000	0.000	0.000	0.000
319	G	0.000	0.490	0.000	0.163	0.283	0.000	0.000	0.000	0.000	0.000
320	U	0.000	0.030	0.000	0.010	0.017	0.000	0.000	0.000	0.000	0.000
321	C	0.000	0.000	0.000	0.000	0.000	0.000	0.000	0.000	0.000	0.000
322	U	0.180	0.280	0.160	0.207	0.064	0.290	0.360	0.160	0.270	0.101
323	C	0.140	0.110	0.110	0.120	0.017	0.390	0.280	0.140	0.270	0.125
324	G	0.420	0.330	0.430	0.393	0.055	0.550	0.580	0.410	0.513	0.091
325	G	0.070	0.280	0.130	0.160	0.108	0.520	0.470	0.550	0.513	0.040
326	G	0.000	0.000	0.000	0.000	0.000	0.000	0.000	0.000	0.000	0.000
327	C	0.000	0.000	0.000	0.000	0.000	0.000	0.000	0.000	0.000	0.000
328	U	0.000	0.060	0.000	0.020	0.035	0.000	0.030	0.000	0.010	0.017
329	C	0.000	0.000	0.000	0.000	0.000	0.000	0.000	0.000	0.000	0.000
330	A	1.010	0.880	1.000	0.963	0.072	0.840	1.110	0.970	0.973	0.135
331	A	1.660	1.450	1.860	1.657	0.205	1.400	1.980	1.930	1.770	0.321
332	A	1.580	1.410	1.730	1.573	0.160	1.770	1.920	1.970	1.887	0.104
333	A	1.570	1.430	1.820	1.607	0.198	2.060	2.070	2.200	2.110	0.078
334	G	0.270	0.260	0.400	0.310	0.078	0.200	0.130	0.190	0.173	0.038
335	G	0.000	0.000	0.000	0.000	0.000	0.000	0.000	0.000	0.000	0.000
336	G	0.000	0.000	0.000	0.000	0.000	0.000	0.000	0.000	0.000	0.000

hSHAPE Reactivities from 3 independent experiments (SP107*i*)											
Nucleotides		In the Absence of Pr77^{Gag}					In the Presence of Pr77^{Gag}				
Number	Sequence	Expt 1	Expt 2	Expt 3	Mean	SD	Expt 1	Expt 2	Expt 3	Mean	SD
337	C	0.000	0.000	0.000	0.000	0.000	0.000	0.000	0.000	0.000	0.000
338	A	0.000	0.000	0.000	0.000	0.000	0.000	0.000	0.000	0.000	0.000
339	G	0.000	0.000	0.000	0.000	0.000	0.000	0.000	0.000	0.000	0.000
340	A	0.100	0.200	0.170	0.157	0.051	0.150	0.320	0.310	0.260	0.095
341	A	0.560	0.610	0.710	0.627	0.076	0.650	0.870	0.730	0.750	0.111
342	A	0.370	0.370	0.470	0.403	0.058	0.550	0.640	0.470	0.553	0.085
343	C	0.000	0.000	0.000	0.000	0.000	0.020	0.000	0.000	0.007	0.012
344	U	0.680	0.570	0.720	0.657	0.078	1.070	1.000	1.050	1.040	0.036
345	C	0.380	0.270	0.180	0.277	0.100	0.460	0.280	0.280	0.340	0.104
346	U	0.300	0.320	0.390	0.337	0.047	0.540	0.530	0.440	0.503	0.055
347	U	0.770	0.720	0.930	0.807	0.110	0.970	1.020	1.010	1.000	0.026
348	U	0.730	0.630	0.800	0.720	0.085	0.880	0.920	0.820	0.873	0.050
349	G	0.050	0.000	0.000	0.017	0.029	0.000	0.000	0.000	0.000	0.000
350	U	0.340	0.350	0.370	0.353	0.015	0.580	0.550	0.510	0.547	0.035
351	U	0.490	0.350	0.480	0.440	0.078	0.680	0.630	0.520	0.610	0.082
352	U	0.000	0.000	0.000	0.000	0.000	0.110	0.010	0.000	0.040	0.061
353	C	0.000	0.000	0.000	0.000	0.000	0.000	0.000	0.000	0.000	0.000
354	U	0.780	0.760	0.690	0.743	0.047	1.190	1.170	1.080	1.147	0.059
355	G	2.370	2.280	2.640	2.430	0.187	3.870	3.600	4.090	3.853	0.245
356	U	0.270	0.290	0.310	0.290	0.020	0.460	0.390	0.330	0.393	0.065
357	U	0.290	0.320	0.390	0.333	0.051	0.470	0.330	0.340	0.380	0.078
358	U	0.150	0.260	0.370	0.260	0.110	0.360	0.240	0.330	0.310	0.062
359	U	0.080	0.180	0.180	0.147	0.058	0.000	0.140	0.150	0.097	0.084
360	A	0.570	0.510	0.590	0.557	0.042	0.480	0.480	0.450	0.470	0.017
361	C	0.000	0.250	0.000	0.083	0.144	0.000	0.000	0.000	0.000	0.000
362	A	0.320	0.310	0.380	0.337	0.038	0.170	0.160	0.220	0.183	0.032
363	A	0.100	0.120	0.150	0.123	0.025	0.130	0.040	0.060	0.077	0.047
364	A	0.040	0.030	0.050	0.040	0.010	0.000	0.000	0.010	0.003	0.006
365	G	0.000	0.000	0.000	0.000	0.000	0.000	0.000	0.000	0.000	0.000
366	G	0.010	0.000	0.000	0.003	0.006	0.000	0.000	0.000	0.000	0.000
367	C	0.000	0.000	0.000	0.000	0.000	0.000	0.000	0.000	0.000	0.000
368	U	0.030	0.000	0.000	0.010	0.017	0.000	0.000	0.000	0.000	0.000
369	C	0.000	0.000	0.000	0.000	0.000	0.000	0.000	0.000	0.000	0.000
370	C	0.000	0.000	0.000	0.000	0.000	0.000	0.000	0.000	0.000	0.000
371	U	0.000	0.000	0.000	0.000	0.000	0.000	0.000	0.000	0.000	0.000
372	C	0.000	0.060	0.000	0.020	0.035	0.000	0.010	0.000	0.003	0.006
373	U	0.300	0.230	0.370	0.300	0.070	0.000	0.410	0.340	0.250	0.219
374	C	1.150	1.190	1.250	1.197	0.050	1.230	1.690	1.860	1.593	0.326
375	A	3.910	3.380	4.260	3.850	0.443	5.200	4.820	5.840	5.287	0.515
376	G	1.960	1.990	2.420	2.123	0.257	2.780	3.210	3.270	3.087	0.267
377	A	2.540	2.150	2.870	2.520	0.360	3.260	3.610	3.670	3.513	0.221
378	G	0.000	0.000	0.000	0.000	0.000	0.000	0.000	0.000	0.000	0.000
379	A	0.000	0.000	0.000	0.000	0.000	0.000	0.000	0.000	0.000	0.000
380	G	0.000	0.000	0.000	0.000	0.000	0.100	0.000	0.000	0.033	0.058
381	G	0.000	0.000	0.000	0.000	0.000	0.000	0.000	0.000	0.000	0.000
382	G	0.000	0.000	0.000	0.000	0.000	0.000	0.000	0.000	0.000	0.000
383	G	0.000	0.000	0.000	0.000	0.000	0.000	0.000	11.770	3.923	6.795
384	U	0.000	0.000	0.050	0.017	0.029	0.000	0.000	0.000	0.000	0.000

hSHAPE Reactivities from 3 independent experiments (SP107*i*)											
Nucleotides		In the Absence of Pr77[Gag]					In the Presence of Pr77[Gag]				
Number	Sequence	Expt 1	Expt 2	Expt 3	Mean	SD	Expt 1	Expt 2	Expt 3	Mean	SD
385	C	0.000	0.000	0.000	0.000	0.000	0.000	0.000	0.000	0.000	0.000
386	U	0.030	0.480	0.000	0.170	0.269	0.000	0.000	0.000	0.000	0.000
387	U	0.120	0.140	0.690	0.317	0.323	0.000	0.000	0.000	0.000	0.000
388	C	0.000	0.000	0.000	0.000	0.000	0.000	0.000	0.000	0.000	0.000
389	A	0.600	0.870	0.720	0.730	0.135	0.880	0.400	0.900	0.727	0.283
390	U	0.060	0.050	0.340	0.150	0.165	0.000	0.000	0.210	0.070	0.121
391	G	0.000	0.000	0.140	0.047	0.081	0.000	0.000	0.000	0.000	0.000
392	U	0.030	0.040	0.140	0.070	0.061	0.100	0.000	0.000	0.033	0.058
393	G	0.050	0.060	0.090	0.067	0.021	0.020	0.000	0.000	0.007	0.012
394	A	0.150	0.170	0.140	0.153	0.015	0.210	0.000	0.000	0.070	0.121
395	A	0.390	0.290	0.280	0.320	0.061	0.350	0.200	0.370	0.307	0.093
396	A	0.800	0.560	0.740	0.700	0.125	0.940	0.750	0.650	0.780	0.147
397	G	0.760	0.600	0.800	0.720	0.106	0.750	0.940	0.690	0.793	0.131
398	A	0.430	0.260	0.370	0.353	0.086	0.860	0.990	0.710	0.853	0.140
399	G	0.580	0.300	0.450	0.443	0.140	0.300	0.120	0.160	0.193	0.095
400	A	0.540	0.290	0.440	0.423	0.126	0.210	0.470	0.350	0.343	0.130
401	G	0.150	0.280	0.140	0.190	0.078	0.000	0.230	0.000	0.077	0.133
402	U	0.000	0.060	0.540	0.200	0.296	0.120	0.000	0.000	0.040	0.069
403	A	0.790	0.390	0.720	0.633	0.214	1.030	1.110	0.800	0.980	0.161
404	G	0.000	0.080	0.180	0.087	0.090	0.520	0.680	0.190	0.463	0.250
405	U	0.280	0.210	0.400	0.297	0.096	0.840	0.420	0.360	0.540	0.262
406	G	0.620	0.650	0.790	0.687	0.091	0.000	0.000	0.000	0.000	0.000
407	C	0.000	0.000	0.000	0.000	0.000	0.000	0.000	0.000	0.000	0.000
408	A	0.070	0.060	0.000	0.043	0.038	0.000	0.000	0.500	0.167	0.289
409	A	0.160	0.070	0.150	0.127	0.049	0.460	0.100	0.610	0.390	0.262
410	U	0.000	0.000	0.050	0.017	0.029	0.000	0.000	0.000	0.000	0.000
411	A	0.000	0.340	0.060	0.133	0.181	0.210	0.210	0.360	0.260	0.087
412	G	0.680	0.300	0.430	0.470	0.193	0.200	0.350	0.320	0.290	0.079
413	A	0.640	0.300	0.160	0.367	0.247	0.330	0.340	0.410	0.360	0.044
414	A	0.470	0.290	0.390	0.383	0.090	0.390	0.420	0.360	0.390	0.030
415	U	0.420	0.370	0.350	0.380	0.036	0.250	0.380	0.330	0.320	0.066
416	U	0.340	0.270	0.340	0.317	0.040	0.000	0.000	0.000	0.000	0.000
417	U	0.000	0.060	0.000	0.020	0.035	0.210	0.390	0.520	0.373	0.156
418	U	0.460	0.080	0.480	0.340	0.225	0.090	0.000	0.000	0.030	0.052
419	A	0.130	0.210	0.130	0.157	0.046	0.490	0.270	0.410	0.390	0.111
420	U	0.260	0.190	0.000	0.150	0.135	0.120	0.010	0.190	0.107	0.091
421	C	0.000	0.270	0.000	0.090	0.156	0.000	0.000	0.000	0.000	0.000
422	A	0.610	0.190	0.650	0.483	0.255	0.220	0.290	0.100	0.203	0.096
423	G	0.230	0.050	0.390	0.223	0.170	0.690	0.200	0.460	0.450	0.245
424	U	0.080	0.370	0.390	0.280	0.173	0.270	0.480	0.510	0.420	0.131
425	U	0.000	0.000	0.190	0.063	0.110	-999	-999	-999	-999	0.000
426	U	0.000	0.160	0.000	0.053	0.092	-999	-999	-999	-999	0.000
427	C	0.000	0.000	0.030	0.010	0.017	-999	-999	-999	-999	0.000
428	U	-999	-999	-999	-999	0.000	-999	-999	-999	-999	0.000
429	A	-999	-999	-999	-999	0.000	-999	-999	-999	-999	0.000
430	A	-999	-999	-999	-999	0.000	-999	-999	-999	-999	0.000
431	U	-999	-999	-999	-999	0.000	-999	-999	-999	-999	0.000
432	A	-999	-999	-999	-999	0.000	-999	-999	-999	-999	0.000

hSHAPE Reactivities from 3 independent experiments (SP108*i*)											
Nucleotides		In the Absence of Pr77^{Gag}					In the Presence of Pr77^{Gag}				
Number	Sequence	Expt 1	Expt 2	Expt 3	Mean	SD	Expt 1	Expt 2	Expt 3	Mean	SD
1	G	-999	-999	-999	-999	0.000	-999	-999	-999	-999	0.000
2	C	-999	-999	-999	-999	0.000	-999	-999	-999	-999	0.000
3	A	-999	-999	-999	-999	0.000	-999	-999	-999	-999	0.000
4	A	-999	-999	-999	-999	0.000	-999	-999	-999	-999	0.000
5	C	-999	-999	-999	-999	0.000	-999	-999	-999	-999	0.000
6	A	-999	-999	-999	-999	0.000	-999	-999	-999	-999	0.000
7	G	-999	-999	-999	-999	0.000	-999	-999	-999	-999	0.000
8	U	-999	-999	-999	-999	0.000	-999	-999	-999	-999	0.000
9	C	-999	-999	-999	-999	0.000	-999	-999	-999	-999	0.000
10	C	-999	-999	-999	-999	0.000	-999	-999	-999	-999	0.000
11	U	-999	-999	-999	-999	0.000	-999	-999	-999	-999	0.000
12	A	-999	-999	-999	-999	0.000	-999	-999	-999	-999	0.000
13	A	-999	-999	-999	-999	0.000	-999	-999	-999	-999	0.000
14	U	-999	-999	-999	-999	0.000	-999	-999	-999	-999	0.000
15	A	-999	-999	-999	-999	0.000	-999	-999	-999	-999	0.000
16	U	-999	-999	-999	-999	0.000	-999	-999	-999	-999	0.000
17	U	0.750	1.165	1.090	1.002	0.221	-999	-999	-999	-999	0.000
18	C	1.690	1.430	0.290	1.137	0.745	-999	-999	-999	-999	0.000
19	A	0.210	0.205	0.200	0.205	0.005	0.145	0.105	0.100	0.117	0.025
20	C	0.120	0.145	0.115	0.127	0.016	0.110	0.120	0.085	0.105	0.018
21	G	0.270	0.720	0.635	0.542	0.239	0.715	0.670	0.675	0.687	0.025
22	U	1.140	2.475	2.405	2.007	0.751	2.125	1.895	2.170	2.063	0.148
23	C	0.300	0.585	0.555	0.480	0.157	0.575	0.685	0.525	0.595	0.082
24	U	1.770	3.475	3.560	2.935	1.010	2.845	2.595	3.150	2.863	0.278
25	C	0.450	0.865	0.830	0.715	0.230	0.875	0.830	0.895	0.867	0.033
26	G	0.080	0.210	0.195	0.162	0.071	0.080	0.160	0.080	0.107	0.046
27	U	0.010	0.060	0.115	0.062	0.053	0.050	0.085	0.070	0.068	0.018
28	G	0.000	0.000	0.000	0.000	0.000	0.000	0.000	0.000	0.000	0.000
29	U	0.230	0.415	0.660	0.435	0.216	0.390	0.355	0.270	0.338	0.062
30	G	0.110	0.245	0.355	0.237	0.123	0.135	0.230	0.265	0.210	0.067
31	U	0.030	0.105	0.130	0.088	0.052	0.215	0.170	0.060	0.148	0.080
32	U	0.140	0.370	0.365	0.292	0.131	0.330	0.345	0.350	0.342	0.010
33	U	0.070	0.290	0.350	0.237	0.147	0.220	0.235	0.275	0.243	0.028
34	G	0.040	0.310	0.410	0.253	0.191	0.235	0.280	0.280	0.265	0.026
35	U	1.415	1.595	1.760	1.590	0.173	1.455	1.245	1.615	1.438	0.186
36	G	0.325	0.375	0.565	0.422	0.127	0.605	0.435	0.660	0.567	0.117
37	U	0.005	0.050	0.125	0.060	0.061	0.055	0.110	0.050	0.072	0.033
38	C	0.045	0.000	0.025	0.023	0.023	0.000	0.000	0.000	0.000	0.000
39	U	0.010	0.000	0.000	0.003	0.006	0.000	0.000	0.000	0.000	0.000
40	G	0.000	0.000	0.000	0.000	0.000	0.000	0.000	0.000	0.000	0.000
41	U	0.040	0.000	0.000	0.013	0.023	0.000	0.000	0.000	0.000	0.000
42	U	0.190	0.230	0.255	0.225	0.033	0.130	0.255	0.175	0.187	0.063
43	C	0.570	0.700	0.910	0.727	0.172	0.585	0.625	0.680	0.630	0.048
44	G	0.495	0.605	0.840	0.647	0.176	0.280	0.230	0.310	0.273	0.040
45	C	0.000	0.000	0.000	0.000	0.000	0.000	0.000	0.000	0.000	0.000
46	C	0.000	0.000	0.000	0.000	0.000	0.000	0.000	0.000	0.000	0.000
47	A	0.890	0.770	0.765	0.808	0.071	0.290	0.505	0.755	0.517	0.233
48	U	0.860	0.925	0.755	0.847	0.086	1.005	0.840	0.980	0.942	0.089

hSHAPE Reactivities from 3 independent experiments (SP108*i*)											
Nucleotides		In the Absence of Pr77^{Gag}					In the Presence of Pr77^{Gag}				
Number	Sequence	Expt 1	Expt 2	Expt 3	Mean	SD	Expt 1	Expt 2	Expt 3	Mean	SD
49	C	0.235	0.210	0.195	0.213	0.020	0.265	0.105	0.175	0.182	0.080
50	C	0.050	0.000	0.000	0.017	0.029	0.000	0.000	0.000	0.000	0.000
51	C	0.070	0.000	0.015	0.028	0.037	0.000	0.000	0.000	0.000	0.000
52	G	0.955	1.005	1.110	1.023	0.079	0.665	0.635	1.135	0.812	0.280
53	U	0.670	0.880	0.860	0.803	0.116	0.520	0.510	0.765	0.598	0.144
54	C	0.310	0.400	0.395	0.368	0.051	0.325	0.365	0.400	0.363	0.038
55	U	0.150	0.180	0.210	0.180	0.030	0.000	0.055	0.215	0.090	0.112
56	C → G	0.030	0.000	0.000	0.010	0.017	0.000	0.010	0.060	0.023	0.032
57	C	0.000	0.000	0.000	0.000	0.000	0.000	0.000	0.030	0.010	0.017
58	G → C	0.050	0.000	0.000	0.017	0.029	0.000	0.000	0.225	0.075	0.130
59	C	0.090	0.000	0.000	0.030	0.052	0.000	0.000	0.225	0.075	0.130
60	U→ A	0.875	0.550	0.680	0.702	0.164	0.800	0.405	0.885	0.697	0.256
61	C	0.445	0.000	0.000	0.148	0.257	0.000	0.100	0.785	0.295	0.427
62	G	0.000	0.000	0.000	0.000	0.000	0.000	0.000	0.000	0.000	0.000
63	U	0.175	0.000	0.000	0.058	0.101	0.010	0.055	0.040	0.035	0.023
64	C	1.805	1.360	0.650	1.272	0.583	0.070	0.100	0.000	0.057	0.051
65	A	0.445	0.310	0.265	0.340	0.094	0.160	0.155	0.035	0.117	0.071
66	C	0.000	0.000	0.000	0.000	0.000	0.000	0.000	0.000	0.000	0.000
67	U	0.345	0.380	0.295	0.340	0.043	0.240	0.235	0.235	0.237	0.003
68	U	0.580	0.495	0.400	0.492	0.090	0.170	0.335	0.255	0.253	0.083
69	A	0.365	0.515	0.420	0.433	0.076	0.395	0.350	0.400	0.382	0.028
70	U	0.170	0.255	0.180	0.202	0.046	0.155	0.200	0.225	0.193	0.035
71	C	0.160	0.000	0.095	0.085	0.080	0.000	0.000	0.025	0.008	0.014
72	C	0.110	0.000	0.000	0.037	0.064	0.000	0.000	0.000	0.000	0.000
73	U	0.810	0.550	0.835	0.732	0.158	0.720	0.760	1.025	0.835	0.166
74	U	1.730	1.835	1.740	1.768	0.058	1.880	1.795	1.895	1.857	0.054
75	C	0.000	0.000	0.000	0.000	0.000	0.710	1.795	0.300	0.935	0.772
76	A	1.955	1.810	1.370	1.712	0.305	1.450	1.455	1.305	1.403	0.085
77	C	0.580	0.110	0.210	0.300	0.248	0.800	0.700	0.650	0.717	0.076
78	U	1.045	1.125	0.850	1.007	0.141	1.765	1.575	1.790	1.710	0.118
79	U	1.750	1.645	1.485	1.627	0.133	1.990	1.955	2.140	2.028	0.098
80	U	1.240	1.050	0.970	1.087	0.139	1.185	1.130	1.205	1.173	0.039
81	C	0.285	0.190	0.135	0.203	0.076	0.135	0.260	0.160	0.185	0.066
82	C	0.220	0.535	0.850	0.535	0.315	0.355	0.550	0.145	0.350	0.203
83	A	1.970	2.005	1.880	1.952	0.064	1.335	1.255	1.245	1.278	0.049
84	G	0.115	0.105	0.100	0.107	0.008	0.000	0.065	0.000	0.022	0.038
85	A	0.080	0.005	0.055	0.047	0.038	0.000	0.000	0.000	0.000	0.000
86	G	0.000	0.000	0.000	0.000	0.000	0.000	0.000	0.000	0.000	0.000
87	G	0.000	0.000	0.000	0.000	0.000	0.000	0.000	0.000	0.000	0.000
88	G	0.000	0.000	0.000	0.000	0.000	0.000	0.000	0.000	0.000	0.000
89	U	0.000	0.000	0.000	0.000	0.000	0.000	0.000	0.000	0.000	0.000
90	C	0.000	0.000	0.000	0.000	0.000	0.000	0.000	0.000	0.000	0.000
91	C	0.025	0.000	0.000	0.008	0.014	0.000	0.000	0.000	0.000	0.000
92	C	0.000	0.000	0.000	0.000	0.000	0.000	0.000	0.000	0.000	0.000
93	C	0.000	0.000	0.000	0.000	0.000	0.000	0.000	0.000	0.000	0.000
94	C	0.000	0.000	0.000	0.000	0.000	0.000	0.000	0.000	0.000	0.000
95	C	0.000	0.000	0.000	0.000	0.000	0.000	0.000	0.000	0.000	0.000
96	G	0.020	0.080	0.065	0.055	0.031	0.000	0.000	0.035	0.012	0.020

hSHAPE Reactivities from 3 independent experiments (SP108*i*)											
Nucleotides		In the Absence of Pr77^{Gag}					In the Presence of Pr77^{Gag}				
Number	Sequence	Expt 1	Expt 2	Expt 3	Mean	SD	Expt 1	Expt 2	Expt 3	Mean	SD
97	C	0.265	0.010	0.230	0.168	0.138	0.000	0.000	0.000	0.000	0.000
98	A	0.530	0.660	0.540	0.577	0.072	0.115	0.245	0.155	0.172	0.067
99	G	0.715	0.915	0.805	0.812	0.100	0.270	0.400	0.445	0.372	0.091
100	A	2.050	2.145	1.800	1.998	0.178	1.295	1.235	1.255	1.262	0.031
101	C	0.295	0.205	0.180	0.227	0.060	0.395	0.175	0.270	0.280	0.110
102	C	0.140	0.000	0.000	0.047	0.081	0.000	0.000	0.000	0.000	0.000
103	C	0.000	0.000	0.000	0.000	0.000	0.000	0.000	0.000	0.000	0.000
104	C	0.000	0.000	0.000	0.000	0.000	0.000	0.000	0.000	0.000	0.000
105	G	0.000	0.000	0.000	0.000	0.000	0.000	0.000	0.000	0.000	0.000
106	G	0.000	0.000	0.000	0.000	0.000	0.000	0.000	0.000	0.000	0.000
107	U	0.110	0.020	0.055	0.062	0.045	0.000	0.000	0.095	0.032	0.055
108	G	0.025	0.000	0.070	0.032	0.035	0.000	0.000	0.000	0.000	0.000
109	A	0.010	0.020	0.020	0.017	0.006	0.000	0.000	0.000	0.000	0.000
110	C	0.000	0.000	0.000	0.000	0.000	0.000	0.000	0.000	0.000	0.000
111	C	0.000	0.000	0.000	0.000	0.000	0.000	0.000	0.000	0.000	0.000
112	C	0.000	0.000	0.000	0.000	0.000	0.000	0.000	0.000	0.000	0.000
113	U	2.540	1.650	1.970	2.053	0.451	0.735	0.885	1.350	0.990	0.321
114	C	1.080	0.465	0.850	0.798	0.311	0.145	0.435	0.590	0.390	0.226
115	A	2.750	3.495	2.935	3.060	0.388	2.500	2.515	2.920	2.645	0.238
116	G	0.260	0.195	0.095	0.183	0.083	0.115	0.115	0.250	0.160	0.078
117	G	0.000	0.000	0.000	0.000	0.000	0.000	0.000	0.000	0.000	0.000
118	U	0.000	0.005	0.005	0.003	0.003	0.000	0.000	0.015	0.005	0.009
119	C	0.280	0.300	0.320	0.300	0.020	0.125	0.030	0.100	0.085	0.049
120	G	0.575	0.230	0.545	0.450	0.191	0.235	0.205	0.360	0.267	0.082
121	G	0.405	0.700	0.790	0.632	0.201	0.720	0.550	0.695	0.655	0.092
122	C	0.000	0.000	0.000	0.000	0.000	0.000	0.000	0.000	0.000	0.000
123	C	0.000	0.000	0.000	0.000	0.000	0.000	0.000	0.000	0.000	0.000
124	G	0.100	0.000	0.000	0.033	0.058	0.000	0.000	0.000	0.000	0.000
125	A	0.335	0.335	0.320	0.330	0.009	0.155	0.330	0.270	0.252	0.089
126	C	0.000	0.000	0.000	0.000	0.000	0.000	0.000	0.000	0.000	0.000
127	U	0.170	0.025	0.025	0.073	0.084	0.000	0.000	0.015	0.005	0.009
128	G	0.225	0.295	0.215	0.245	0.044	0.130	0.070	0.055	0.085	0.040
129	C	0.000	0.000	0.000	0.000	0.000	0.000	0.000	0.000	0.000	0.000
130	G	0.000	0.000	0.000	0.000	0.000	0.000	0.000	0.000	0.000	0.000
131	G	0.000	0.000	0.000	0.000	0.000	0.000	0.000	0.000	0.000	0.000
132	C	0.000	0.000	0.000	0.000	0.000	0.000	0.000	0.000	0.000	0.000
133	A	0.000	0.000	0.000	0.000	0.000	0.000	0.000	0.000	0.000	0.000
134	G	0.400	0.220	0.490	0.370	0.137	0.020	0.000	0.045	0.022	0.023
135	C	0.300	0.110	0.260	0.223	0.100	0.290	0.435	0.395	0.373	0.075
136	U	0.510	0.460	0.550	0.507	0.045	0.550	0.360	0.515	0.475	0.101
137	G	0.250	0.290	0.360	0.300	0.056	0.145	0.035	0.120	0.100	0.058
138	G	0.000	0.000	0.000	0.000	0.000	0.000	0.000	0.000	0.000	0.000
139	C	0.000	0.000	0.000	0.000	0.000	0.000	0.000	0.000	0.000	0.000
140	G	0.060	0.040	0.080	0.060	0.020	0.015	0.000	0.000	0.005	0.009
141	C	0.000	0.000	0.000	0.000	0.000	0.000	0.000	0.000	0.000	0.000
142	C	0.000	0.000	0.000	0.000	0.000	0.000	0.000	0.000	0.000	0.000
143	C	0.000	0.000	0.000	0.000	0.000	0.000	0.000	0.000	0.000	0.000
144	G	0.558	0.291	0.240	0.363	0.171	0.160	0.032	0.047	0.080	0.070

hSHAPE Reactivities from 3 independent experiments (SP108*i*)											
Nucleotides		In the Absence of Pr77^{Gag}					In the Presence of Pr77^{Gag}				
Number	Sequence	Expt 1	Expt 2	Expt 3	Mean	SD	Expt 1	Expt 2	Expt 3	Mean	SD
145	A	1.440	1.220	1.150	1.270	0.151	0.450	0.890	0.905	0.748	0.258
146	A	1.570	1.320	1.180	1.357	0.198	0.730	1.080	0.825	0.878	0.181
147	C	1.910	1.210	1.425	1.515	0.359	0.370	0.610	0.260	0.413	0.179
148	A	0.885	0.800	0.640	0.775	0.124	0.605	0.630	0.455	0.563	0.095
149	G	0.150	0.000	0.000	0.050	0.087	0.000	0.000	0.000	0.000	0.000
150	G	0.000	0.000	0.000	0.000	0.000	0.000	0.000	0.000	0.000	0.000
151	G	0.000	0.000	0.000	0.000	0.000	0.000	0.000	0.000	0.000	0.000
152	A	0.000	0.000	0.000	0.000	0.000	0.000	0.000	0.000	0.000	0.000
153	C	0.000	0.000	0.000	0.000	0.000	0.000	0.000	0.000	0.000	0.000
154	C	0.000	0.000	0.000	0.000	0.000	0.000	0.000	0.000	0.000	0.000
155	C	0.000	0.000	0.000	0.000	0.000	0.000	0.000	0.000	0.000	0.000
156	U	0.080	0.000	0.000	0.027	0.046	0.000	0.010	0.000	0.003	0.006
157	C	0.390	0.000	0.210	0.200	0.195	0.070	0.295	0.175	0.180	0.113
158	G	0.000	0.000	0.000	0.000	0.000	0.005	0.000	0.000	0.002	0.003
159	G	0.000	0.000	0.000	0.000	0.000	0.000	0.000	0.000	0.000	0.000
160	A	0.000	0.000	0.000	0.000	0.000	0.000	0.000	0.000	0.000	0.000
161	U	0.155	0.240	0.170	0.188	0.045	0.065	0.035	0.070	0.057	0.019
162	A	0.025	0.000	0.000	0.008	0.014	0.000	0.000	0.000	0.000	0.000
163	A	0.020	0.000	0.010	0.010	0.010	0.000	0.095	0.105	0.067	0.058
164	G	0.000	0.000	0.000	0.000	0.000	0.000	0.000	0.000	0.000	0.000
165	U	0.000	0.000	0.000	0.000	0.000	0.000	0.000	0.000	0.000	0.000
166	G	0.000	0.000	0.000	0.000	0.000	0.000	0.000	0.000	0.000	0.000
167	A	0.000	0.000	0.000	0.000	0.000	0.000	0.000	0.000	0.000	0.000
168	C	0.000	0.000	0.000	0.000	0.000	0.000	0.000	0.000	0.000	0.000
169	C	0.095	0.005	0.000	0.033	0.053	0.000	0.000	0.085	0.028	0.049
170	C	0.000	0.000	0.000	0.000	0.000	0.000	0.000	0.040	0.013	0.023
171	U	0.420	0.120	0.090	0.210	0.182	0.260	0.075	0.295	0.210	0.118
172	U	0.390	0.395	0.325	0.370	0.039	0.365	0.340	0.505	0.403	0.089
173	G	0.635	0.730	0.820	0.728	0.093	1.170	1.115	1.230	1.172	0.058
174	U	0.410	0.535	0.510	0.485	0.066	0.715	0.710	0.835	0.753	0.071
175	C	0.000	0.000	0.000	0.000	0.000	0.000	0.000	0.000	0.000	0.000
176	U	0.000	0.000	0.000	0.000	0.000	0.000	0.000	0.000	0.000	0.000
177	C	0.000	0.000	0.000	0.000	0.000	0.000	0.000	0.000	0.000	0.000
178	U	0.365	0.000	0.400	0.255	0.222	0.000	0.225	0.255	0.160	0.139
179	A	0.390	0.320	0.340	0.350	0.036	0.270	0.345	0.380	0.332	0.056
180	U	0.025	0.000	0.000	0.008	0.014	0.130	0.170	0.115	0.138	0.028
181	U	0.070	0.080	0.040	0.063	0.021	0.180	0.230	0.170	0.193	0.032
182	U	0.055	0.100	0.045	0.067	0.029	0.030	0.125	0.070	0.075	0.048
183	C	0.025	0.000	0.000	0.008	0.014	0.000	0.000	0.040	0.013	0.023
184	U	0.480	0.325	0.240	0.348	0.122	0.115	0.275	0.340	0.243	0.116
185	A	0.105	0.220	0.000	0.108	0.110	0.135	0.135	0.365	0.212	0.133
186	C	0.135	0.170	0.100	0.135	0.035	0.000	0.000	0.055	0.018	0.032
187	U	0.855	0.940	0.880	0.892	0.044	0.470	0.585	0.475	0.510	0.065
188	A	0.695	1.055	0.800	0.850	0.185	0.660	0.695	0.780	0.712	0.062
189	U	0.145	0.225	0.055	0.142	0.085	0.090	0.150	0.140	0.127	0.032
190	U	0.050	0.125	0.020	0.065	0.054	0.035	0.075	0.080	0.063	0.025
191	U	0.140	0.195	0.080	0.138	0.058	0.115	0.160	0.155	0.143	0.025
192	G	0.000	0.000	0.025	0.008	0.014	0.000	0.020	0.000	0.007	0.012

hSHAPE Reactivities from 3 independent experiments (SP108*i*)											
Nucleotides		In the Absence of Pr77^{Gag}					In the Presence of Pr77^{Gag}				
Number	Sequence	Expt 1	Expt 2	Expt 3	Mean	SD	Expt 1	Expt 2	Expt 3	Mean	SD
193	G	0.240	0.160	0.090	0.163	0.075	0.000	0.000	0.140	0.047	0.081
194	U	0.250	0.350	0.245	0.282	0.059	0.000	0.040	0.295	0.112	0.160
195	G	0.315	0.475	0.585	0.458	0.136	0.490	0.340	0.690	0.507	0.176
196	U	0.470	0.440	0.480	0.463	0.021	0.175	0.305	0.425	0.302	0.125
197	U	0.630	0.505	0.515	0.550	0.069	0.290	0.355	0.535	0.393	0.127
198	U	0.360	0.350	0.220	0.310	0.078	0.095	0.205	0.330	0.210	0.118
199	G	0.120	0.070	0.030	0.073	0.045	0.000	0.000	0.160	0.053	0.092
200	U	0.060	0.075	0.055	0.063	0.010	0.000	0.050	0.225	0.092	0.118
201	C	0.000	0.000	0.000	0.000	0.000	0.000	0.000	0.000	0.000	0.000
202	U	0.175	0.120	0.150	0.148	0.028	0.270	0.145	0.270	0.228	0.072
203	U	0.335	0.330	0.280	0.315	0.030	0.465	0.300	0.365	0.377	0.083
204	G	0.350	0.290	0.310	0.317	0.031	0.315	0.235	0.290	0.280	0.041
205	U	0.415	0.400	0.405	0.407	0.008	0.385	0.465	0.405	0.418	0.042
206	A	0.300	0.355	0.380	0.345	0.041	0.305	0.275	0.430	0.337	0.082
207	U	0.360	0.240	0.310	0.303	0.060	0.290	0.270	0.430	0.330	0.087
208	U	0.340	0.280	0.255	0.292	0.044	0.375	0.270	0.400	0.348	0.069
209	G	0.410	0.490	0.560	0.487	0.075	0.900	0.785	0.940	0.875	0.080
210	U	0.035	0.055	0.060	0.050	0.013	0.140	0.100	0.125	0.122	0.020
211	C	0.000	0.000	0.000	0.000	0.000	0.000	0.000	0.000	0.000	0.000
212	U	0.000	0.000	0.000	0.000	0.000	0.000	0.000	0.000	0.000	0.000
213	C	0.000	0.000	0.000	0.000	0.000	0.000	0.000	0.000	0.000	0.000
214	U	0.000	0.000	0.000	0.000	0.000	0.000	0.000	0.000	0.000	0.000
215	U	0.045	0.000	0.000	0.015	0.026	0.000	0.100	0.095	0.065	0.056
216	U	0.155	0.060	0.090	0.102	0.049	0.155	0.240	0.140	0.178	0.054
217	C	0.000	0.000	0.000	0.000	0.000	0.000	0.000	0.000	0.000	0.000
218	U	0.230	0.140	0.160	0.177	0.047	0.230	0.205	0.305	0.247	0.052
219	U	0.285	0.335	0.445	0.355	0.082	0.540	0.500	0.460	0.500	0.040
220	G	0.565	0.650	0.775	0.663	0.106	0.940	0.845	0.945	0.910	0.056
221	U	0.250	0.180	0.260	0.230	0.044	0.355	0.325	0.375	0.352	0.025
222	C	0.130	0.080	0.140	0.117	0.032	0.145	0.185	0.190	0.173	0.025
223	U	0.200	0.210	0.245	0.218	0.024	0.260	0.300	0.310	0.290	0.026
224	G	0.370	0.430	0.430	0.410	0.035	0.550	0.475	0.400	0.475	0.075
225	G	0.075	0.000	0.035	0.037	0.038	0.095	0.120	0.175	0.130	0.041
226	C	0.045	0.000	0.000	0.015	0.026	0.000	0.000	0.070	0.023	0.040
227	U	0.630	0.705	0.590	0.642	0.058	0.490	0.645	0.575	0.570	0.078
228	A	0.785	1.030	0.865	0.893	0.125	0.700	0.715	0.835	0.750	0.074
229	U	0.445	0.460	0.315	0.407	0.080	0.340	0.385	0.410	0.378	0.035
230	C	0.000	0.000	0.000	0.000	0.000	0.670	0.850	0.220	0.580	0.324
231	A	0.775	0.965	0.625	0.788	0.170	1.030	0.890	0.845	0.922	0.096
232	U	0.400	0.225	0.350	0.325	0.090	0.325	0.385	0.275	0.328	0.055
233	C	0.000	0.450	0.940	0.463	0.470	0.580	0.955	0.340	0.625	0.310
234	A	0.890	0.755	0.820	0.822	0.068	0.690	0.620	0.680	0.663	0.038
235	C	0.000	0.000	0.000	0.000	0.000	0.460	0.910	0.350	0.573	0.297
236	A	1.190	0.990	0.940	1.040	0.132	0.580	0.590	0.590	0.587	0.006
237	A	0.865	0.975	0.960	0.933	0.060	0.500	0.480	0.580	0.520	0.053
238	G	0.210	0.295	0.305	0.270	0.052	0.110	0.140	0.105	0.118	0.019
239	A → U	0.175	0.315	0.295	0.262	0.076	0.110	0.115	0.125	0.117	0.008
240	G	0.000	0.035	0.000	0.012	0.020	0.000	0.000	0.000	0.000	0.000

hSHAPE Reactivities from 3 independent experiments (SP108*i*)											
**Nucleotides**		**In the Absence of Pr77^{Gag}**					**In the Presence of Pr77^{Gag}**				
**Number**	**Sequence**	**Expt 1**	**Expt 2**	**Expt 3**	**Mean**	**SD**	**Expt 1**	**Expt 2**	**Expt 3**	**Mean**	**SD**
241	C → G	0.000	0.000	0.000	0.000	0.000	0.000	0.000	0.000	0.000	0.000
242	G	0.000	0.000	0.000	0.000	0.000	0.000	0.000	0.055	0.018	0.032
243	G → C	0.000	0.000	0.000	0.000	0.000	0.000	0.000	0.000	0.000	0.000
244	A	0.135	0.135	0.210	0.160	0.043	0.240	0.220	0.270	0.243	0.025
245	A	0.480	0.595	0.640	0.572	0.083	0.550	0.555	0.620	0.575	0.039
246	C	0.155	0.040	0.215	0.137	0.089	0.000	0.085	0.220	0.102	0.111
247	G	0.000	0.000	0.000	0.000	0.000	0.000	0.000	0.000	0.000	0.000
248	G	0.000	0.000	0.000	0.000	0.000	0.000	0.000	0.000	0.000	0.000
249	A	2.545	3.140	3.290	2.992	0.394	2.675	2.595	2.800	2.690	0.103
250	C	0.000	0.000	0.000	0.000	0.000	0.000	0.000	0.000	0.000	0.000
251	U	0.035	0.000	0.000	0.012	0.020	0.000	0.105	0.000	0.035	0.061
252	C	0.235	0.180	0.165	0.193	0.037	0.000	0.100	0.000	0.033	0.058
253	A	0.260	0.325	0.355	0.313	0.049	0.035	0.140	0.130	0.102	0.058
254	C	0.000	0.000	0.000	0.000	0.000	0.000	0.000	0.000	0.000	0.000
255	C	0.250	0.020	0.010	0.093	0.136	0.010	0.035	0.000	0.015	0.018
256	A	0.475	0.525	0.590	0.530	0.058	0.590	0.560	0.530	0.560	0.030
257	U	0.890	0.740	1.045	0.892	0.153	1.310	1.400	1.180	1.297	0.111
258	A	1.540	1.705	1.590	1.612	0.085	1.560	1.465	1.385	1.470	0.088
259	G	0.555	0.705	0.655	0.638	0.076	0.520	0.520	0.440	0.493	0.046
260	G	0.200	0.230	0.215	0.215	0.015	0.135	0.170	0.110	0.138	0.030
261	G	0.220	0.230	0.215	0.222	0.008	0.135	0.150	0.190	0.158	0.028
262	A	0.670	0.820	0.750	0.747	0.075	0.635	0.595	0.610	0.613	0.020
263	G	0.400	0.320	0.390	0.370	0.044	0.175	0.230	0.230	0.212	0.032
264	C	0.330	0.175	0.265	0.257	0.078	0.130	0.090	0.185	0.135	0.048
265	U	1.065	0.830	0.965	0.953	0.118	0.275	0.285	0.320	0.293	0.024
266	G	0.970	0.770	0.770	0.837	0.115	0.000	0.000	0.225	0.075	0.130
267	C	0.000	0.000	0.000	0.000	0.000	0.000	0.000	0.005	0.002	0.003
268	A	1.180	0.630	0.890	0.900	0.275	0.000	0.000	0.215	0.072	0.124
269	G	0.590	0.480	0.360	0.477	0.115	0.000	0.000	0.000	0.000	0.000
270	U	0.050	0.000	0.070	0.040	0.036	0.005	0.000	0.000	0.002	0.003
271	C	0.000	0.000	0.000	0.000	0.000	0.000	0.000	0.000	0.000	0.000
272	C	0.000	0.000	0.000	0.000	0.000	0.000	0.000	0.000	0.000	0.000
273	C	0.000	0.000	0.000	0.000	0.000	0.000	0.000	0.000	0.000	0.000
274	G	0.000	0.000	0.000	0.000	0.000	0.000	0.000	0.000	0.000	0.000
275	C	0.000	0.000	0.000	0.000	0.000	0.000	0.000	0.000	0.000	0.000
276	C	0.000	0.000	0.000	0.000	0.000	0.025	0.010	0.000	0.012	0.013
277	U	0.030	0.030	0.000	0.020	0.017	0.070	0.105	0.010	0.062	0.048
278	A	0.040	0.040	0.065	0.048	0.014	0.040	0.045	0.050	0.045	0.005
279	C	0.045	0.145	0.120	0.103	0.052	0.000	0.000	0.060	0.020	0.035
280	G	0.320	0.345	0.420	0.362	0.052	0.100	0.075	0.190	0.122	0.060
281	G	1.790	1.760	1.800	1.783	0.021	1.265	1.170	1.100	1.178	0.083
282	A	2.860	2.830	3.140	2.943	0.171	2.120	1.900	2.000	2.007	0.110
283	G	1.190	1.115	1.320	1.208	0.104	0.790	0.905	0.850	0.848	0.058
284	A	2.940	2.840	3.060	2.947	0.110	1.790	1.690	1.590	1.690	0.100
285	A	2.420	2.460	2.680	2.520	0.140	1.370	1.500	1.270	1.380	0.115
286	G	1.585	1.615	1.810	1.670	0.122	1.020	1.295	1.295	1.203	0.159
287	A	3.230	3.510	3.525	3.422	0.166	2.280	2.310	1.990	2.193	0.177
288	G	1.420	1.090	1.030	1.180	0.210	0.580	0.700	0.440	0.573	0.130

hSHAPE Reactivities from 3 independent experiments (SP108*i*)											
Nucleotides		In the Absence of Pr77^{Gag}					In the Presence of Pr77^{Gag}				
Number	Sequence	Expt 1	Expt 2	Expt 3	Mean	SD	Expt 1	Expt 2	Expt 3	Mean	SD
289	G	0.175	0.000	0.100	0.092	0.088	0.000	0.000	0.000	0.000	0.000
290	U	0.000	0.000	0.000	0.000	0.000	0.000	0.000	0.000	0.000	0.000
291	A	0.050	0.060	0.045	0.052	0.008	0.105	0.035	0.040	0.060	0.039
292	G	0.420	0.540	0.460	0.473	0.061	0.080	0.140	0.000	0.073	0.070
293	G	0.240	0.200	0.000	0.147	0.129	0.000	0.000	0.000	0.000	0.000
294	U	0.460	0.000	0.290	0.250	0.233	0.170	0.370	0.470	0.337	0.153
295	U	0.690	0.090	0.470	0.417	0.304	0.620	0.690	0.795	0.702	0.088
296	A	1.400	1.030	1.040	1.157	0.211	1.005	1.130	1.265	1.133	0.130
297	C	0.880	0.730	0.770	0.793	0.078	0.835	0.820	0.910	0.855	0.048
298	G	0.060	0.000	0.010	0.023	0.032	0.045	0.100	0.105	0.083	0.033
299	G	0.120	0.010	0.050	0.060	0.056	0.015	0.085	0.050	0.050	0.035
300	U	0.180	0.150	0.130	0.153	0.025	0.160	0.080	0.160	0.133	0.046
301	G	0.080	0.000	0.000	0.027	0.046	0.000	0.000	0.000	0.000	0.000
302	A	0.010	0.000	0.000	0.003	0.006	0.000	0.000	0.000	0.000	0.000
303	G	0.000	0.000	0.000	0.000	0.000	0.000	0.000	0.000	0.000	0.000
304	C	0.000	0.000	0.000	0.000	0.000	0.000	0.000	0.000	0.000	0.000
305	C	0.000	0.000	0.000	0.000	0.000	0.000	0.000	0.000	0.000	0.000
306	A	0.320	0.610	0.210	0.380	0.207	0.090	0.280	0.040	0.137	0.127
307	U	0.900	0.240	0.610	0.583	0.331	0.820	0.860	0.940	0.873	0.061
308	U	1.230	1.150	1.030	1.137	0.101	1.150	1.200	1.330	1.227	0.093
309	G	1.030	0.920	0.750	0.900	0.141	0.760	0.840	0.820	0.807	0.042
310	G	1.030	0.930	0.720	0.893	0.158	0.630	0.560	0.770	0.653	0.107
311	A	0.750	0.550	0.580	0.627	0.108	0.480	0.450	0.650	0.527	0.108
312	A	0.540	0.350	0.390	0.427	0.100	0.320	0.310	0.370	0.333	0.032
313	A	0.470	0.320	0.300	0.363	0.093	0.320	0.280	0.330	0.310	0.026
314	U	0.510	0.440	0.290	0.413	0.112	0.240	0.160	0.300	0.233	0.070
315	G	0.220	0.010	0.000	0.077	0.124	0.000	0.000	0.030	0.010	0.017
316	G	0.000	0.220	0.000	0.073	0.127	0.000	0.000	0.000	0.000	0.000
317	G	0.000	3.800	0.000	1.267	2.194	0.000	0.000	1.540	0.513	0.889
318	G	0.000	0.000	0.000	0.000	0.000	0.000	0.000	0.000	0.000	0.000
319	G	0.000	0.000	0.000	0.000	0.000	0.000	0.000	1.350	0.450	0.779
320	U	0.000	0.000	0.000	0.000	0.000	0.000	0.000	0.780	0.260	0.450
321	C	0.000	0.000	0.000	0.000	0.000	0.000	0.000	0.060	0.020	0.035
322	U	0.310	0.210	0.200	0.240	0.061	0.180	0.110	0.300	0.197	0.096
323	C	0.170	0.200	0.130	0.167	0.035	0.050	0.080	0.100	0.077	0.025
324	G	0.460	0.570	0.310	0.447	0.131	0.060	0.060	0.120	0.080	0.035
325	G	0.300	0.060	0.110	0.157	0.127	0.000	0.000	0.020	0.007	0.012
326	G	0.000	0.000	0.000	0.000	0.000	0.000	0.000	0.000	0.000	0.000
327	C	0.000	0.000	0.000	0.000	0.000	0.000	0.000	0.000	0.000	0.000
328	U	0.130	0.000	0.300	0.143	0.150	0.210	0.410	0.590	0.403	0.190
329	C	0.000	0.000	0.980	0.327	0.566	0.000	0.880	0.000	0.293	0.508
330	A	1.140	0.880	0.820	0.947	0.170	0.760	0.830	0.890	0.827	0.065
331	A	1.800	1.610	1.310	1.573	0.247	1.130	1.040	1.270	1.147	0.116
332	A	1.700	1.630	1.290	1.540	0.219	1.150	1.090	1.300	1.180	0.108
333	A	1.640	1.520	1.250	1.470	0.200	1.290	1.250	1.440	1.327	0.100
334	G	0.310	0.230	0.220	0.253	0.049	0.100	0.160	0.130	0.130	0.030
335	G	0.020	0.000	0.000	0.007	0.012	0.000	0.000	0.000	0.000	0.000
336	G	0.000	0.000	0.000	0.000	0.000	0.000	0.000	0.000	0.000	0.000

hSHAPE Reactivities from 3 independent experiments (SP108*i*)											
Nucleotides		In the Absence of Pr77^{Gag}					In the Presence of Pr77^{Gag}				
Number	Sequence	Expt 1	Expt 2	Expt 3	Mean	SD	Expt 1	Expt 2	Expt 3	Mean	SD
337	C	0.000	0.000	0.000	0.000	0.000	0.000	0.000	0.000	0.000	0.000
338	A	0.020	0.000	0.000	0.007	0.012	0.000	0.000	0.000	0.000	0.000
339	G	0.000	0.000	0.000	0.000	0.000	0.000	0.000	0.000	0.000	0.000
340	A	0.320	0.140	0.180	0.213	0.095	0.160	0.190	0.000	0.117	0.102
341	A	0.760	0.630	0.580	0.657	0.093	0.670	0.650	0.500	0.607	0.093
342	A	0.390	0.330	0.320	0.347	0.038	0.340	0.350	0.150	0.280	0.113
343	C	0.000	0.000	0.010	0.003	0.006	0.000	0.000	0.000	0.000	0.000
344	U	0.840	0.780	0.720	0.780	0.060	0.750	0.670	0.770	0.730	0.053
345	C	0.630	0.480	0.470	0.527	0.090	0.160	0.080	0.090	0.110	0.044
346	U	0.590	0.520	0.470	0.527	0.060	0.260	0.270	0.200	0.243	0.038
347	U	1.030	1.040	0.870	0.980	0.095	0.470	0.470	0.550	0.497	0.046
348	U	0.830	0.830	0.680	0.780	0.087	0.480	0.490	0.510	0.493	0.015
349	G	0.160	0.000	0.000	0.053	0.092	0.000	0.000	0.000	0.000	0.000
350	U	0.480	0.360	0.300	0.380	0.092	0.120	0.120	0.090	0.110	0.017
351	U	0.590	0.440	0.350	0.460	0.121	0.220	0.210	0.130	0.187	0.049
352	U	0.000	0.000	0.000	0.000	0.000	0.000	0.000	0.000	0.000	0.000
353	C	0.000	0.000	0.000	0.000	0.000	0.000	0.000	0.000	0.000	0.000
354	U	1.230	1.280	1.150	1.220	0.066	0.480	0.620	0.860	0.653	0.192
355	G	3.150	3.100	2.600	2.950	0.304	2.400	2.340	3.140	2.627	0.446
356	U	0.330	0.180	0.220	0.243	0.078	0.520	0.420	0.550	0.497	0.068
357	U	0.330	0.160	0.160	0.217	0.098	0.510	0.450	0.500	0.487	0.032
358	U	0.270	0.130	0.230	0.210	0.072	0.450	0.420	0.490	0.453	0.035
359	U	0.120	0.220	0.270	0.203	0.076	0.310	0.340	0.470	0.373	0.085
360	A	0.420	0.500	0.440	0.453	0.042	0.510	0.520	0.570	0.533	0.032
361	C	0.000	0.000	0.500	0.167	0.289	0.120	0.430	0.000	0.183	0.222
362	A	0.300	0.250	0.230	0.260	0.036	0.290	0.270	0.250	0.270	0.020
363	A	0.070	0.080	0.060	0.070	0.010	0.050	0.090	0.040	0.060	0.026
364	A	0.020	0.000	0.000	0.007	0.012	0.000	0.000	0.000	0.000	0.000
365	G	0.000	0.000	0.000	0.000	0.000	0.000	0.000	0.000	0.000	0.000
366	G	0.000	0.000	0.000	0.000	0.000	0.000	0.000	0.000	0.000	0.000
367	C	0.000	0.000	0.000	0.000	0.000	0.000	0.000	0.000	0.000	0.000
368	U	0.000	0.000	0.000	0.000	0.000	0.000	0.000	0.000	0.000	0.000
369	C	0.000	0.000	0.000	0.000	0.000	0.000	0.000	0.000	0.000	0.000
370	C	0.000	0.000	0.000	0.000	0.000	0.000	0.000	0.000	0.000	0.000
371	U	0.000	0.000	0.000	0.000	0.000	0.000	0.000	0.000	0.000	0.000
372	C	0.050	0.000	0.030	0.027	0.025	0.060	0.110	0.040	0.070	0.036
373	U	0.280	0.420	0.400	0.367	0.076	0.540	0.580	0.430	0.517	0.078
374	C	1.310	1.980	2.010	1.767	0.396	2.160	2.170	1.800	2.043	0.211
375	A	4.280	4.940	3.530	4.250	0.705	3.370	3.080	3.950	3.467	0.443
376	G	2.520	2.330	1.990	2.280	0.269	2.160	2.050	2.510	2.240	0.240
377	A	2.740	2.770	2.270	2.593	0.280	2.540	2.470	3.120	2.710	0.357
378	G	0.020	0.000	0.000	0.007	0.012	0.000	0.000	0.000	0.000	0.000
379	A	0.000	0.020	0.060	0.027	0.031	0.000	0.000	0.000	0.000	0.000
380	G	0.000	0.000	0.010	0.003	0.006	0.000	0.000	0.000	0.000	0.000
381	G	0.000	0.000	0.000	0.000	0.000	0.000	0.000	0.000	0.000	0.000
382	G	7.450	0.000	0.000	2.483	4.301	0.000	0.000	0.000	0.000	0.000
383	G	0.000	0.000	0.000	0.000	0.000	0.000	0.000	0.000	0.000	0.000
384	U	0.000	0.000	0.000	0.000	0.000	0.000	0.000	0.000	0.000	0.000

hSHAPE Reactivities from 3 independent experiments (SP108*i*)											
Nucleotides		In the Absence of Pr77^{Gag}					In the Presence of Pr77^{Gag}				
Number	Sequence	Expt 1	Expt 2	Expt 3	Mean	SD	Expt 1	Expt 2	Expt 3	Mean	SD
385	C	0.000	3.640	0.300	1.313	2.021	0.000	0.000	0.000	0.000	0.000
386	U	0.030	0.000	1.110	0.380	0.632	0.880	0.000	0.140	0.340	0.473
387	U	0.100	0.070	1.780	0.650	0.979	0.680	0.850	0.960	0.830	0.141
388	C	0.000	0.000	1.110	0.370	0.641	1.340	1.130	0.500	0.990	0.437
389	A	0.980	0.920	0.230	0.710	0.417	0.190	1.090	1.270	0.850	0.579
390	U	0.100	0.090	0.050	0.080	0.026	0.050	0.110	0.100	0.087	0.032
391	G	0.010	0.020	0.060	0.030	0.026	0.090	0.010	0.000	0.033	0.049
392	U	0.020	0.040	0.000	0.020	0.020	0.070	0.040	0.000	0.037	0.035
393	G	0.070	0.050	0.020	0.047	0.025	0.090	0.070	0.010	0.057	0.042
394	A	0.160	0.080	0.110	0.117	0.040	0.200	0.060	0.030	0.097	0.091
395	A	0.230	0.190	0.530	0.317	0.186	0.630	0.170	0.170	0.323	0.266
396	A	0.610	0.590	0.510	0.570	0.053	0.620	0.470	0.570	0.553	0.076
397	G	0.760	0.600	0.150	0.503	0.316	0.280	0.610	0.710	0.533	0.225
398	A	0.250	0.170	0.120	0.180	0.066	0.380	0.300	0.210	0.297	0.085
399	G	0.310	0.130	0.210	0.217	0.090	0.470	0.390	0.320	0.393	0.075
400	A	0.410	0.220	0.080	0.237	0.166	0.370	0.430	0.290	0.363	0.070
401	G	0.070	0.030	0.760	0.287	0.410	0.970	0.370	0.130	0.490	0.433
402	U	0.630	1.040	0.000	0.557	0.524	0.720	0.970	0.770	0.820	0.132
403	A	0.160	0.590	0.000	0.250	0.305	0.330	0.710	0.660	0.567	0.206
404	G	0.000	0.260	0.020	0.093	0.145	0.460	0.350	0.270	0.360	0.095
405	U	0.000	0.360	0.140	0.167	0.181	0.780	0.490	0.320	0.530	0.233
406	G	0.110	0.000	0.120	0.077	0.067	0.040	0.760	0.650	0.483	0.388
407	C	0.000	0.000	0.810	0.270	0.468	0.330	0.540	0.000	0.290	0.272
408	A	0.220	0.000	0.340	0.187	0.172	0.480	0.340	0.180	0.333	0.150
409	A	0.370	0.220	0.310	0.300	0.075	0.480	0.430	0.460	0.457	0.025
410	U	0.250	0.230	0.410	0.297	0.099	0.400	0.450	0.520	0.457	0.060
411	A	0.430	0.290	0.270	0.330	0.087	0.420	0.360	0.520	0.433	0.081
412	G	0.360	0.160	0.200	0.240	0.106	0.420	0.290	0.290	0.333	0.075
413	A	0.380	0.250	0.260	0.297	0.072	0.420	0.350	0.410	0.393	0.038
414	A	0.440	0.270	0.270	0.327	0.098	0.380	0.370	0.430	0.393	0.032
415	U	0.350	0.200	0.220	0.257	0.081	0.000	0.340	0.310	0.217	0.188
416	U	0.160	0.080	0.000	0.080	0.080	0.540	0.140	0.140	0.273	0.231
417	U	0.590	0.020	0.320	0.310	0.285	0.290	0.410	0.380	0.360	0.062
418	U	0.050	0.260	0.580	0.297	0.267	0.320	0.380	0.460	0.387	0.070
419	A	0.430	0.000	0.120	0.183	0.222	0.120	0.480	0.580	0.393	0.242
420	U	0.000	0.000	0.200	0.067	0.115	0.260	0.150	0.190	0.200	0.056
421	C	0.000	0.000	0.520	0.173	0.300	0.480	0.760	0.000	0.413	0.384
422	A	0.660	0.060	0.250	0.323	0.307	0.330	0.430	0.590	0.450	0.131
423	G	0.340	0.180	0.180	0.233	0.092	0.170	0.220	0.190	0.193	0.025
424	U	0.260	0.040	0.060	0.120	0.122	0.130	0.180	0.060	0.123	0.060
425	U	0.140	0.050	0.000	0.063	0.071	0.060	0.090	0.000	0.050	0.046
426	U	-999	-999	-999	-999	0.000	-999	-999	-999	-999	0.000
427	C	-999	-999	-999	-999	0.000	-999	-999	-999	-999	0.000
428	U	-999	-999	-999	-999	0.000	-999	-999	-999	-999	0.000
429	A	-999	-999	-999	-999	0.000	-999	-999	-999	-999	0.000
430	A	-999	-999	-999	-999	0.000	-999	-999	-999	-999	0.000
431	U	-999	-999	-999	-999	0.000	-999	-999	-999	-999	0.000
432	A	-999	-999	-999	-999	0.000	-999	-999	-999	-999	0.000

hSHAPE Reactivities from 3 independent experiments (SP109*i*)											
Nucleotides		In the Absence of Pr77^{Gag}					In the Presence of Pr77^{Gag}				
Number	Sequence	Expt 1	Expt 2	Expt 3	Mean	SD	Expt 1	Expt 2	Expt 3	Mean	SD
1	G	-999	-999	-999	-999	0.000	-999	-999	-999	-999	0.000
2	C	-999	-999	-999	-999	0.000	-999	-999	-999	-999	0.000
3	A	-999	-999	-999	-999	0.000	-999	-999	-999	-999	0.000
4	A	-999	-999	-999	-999	0.000	-999	-999	-999	-999	0.000
5	C	-999	-999	-999	-999	0.000	-999	-999	-999	-999	0.000
6	A	-999	-999	-999	-999	0.000	-999	-999	-999	-999	0.000
7	G	-999	-999	-999	-999	0.000	-999	-999	-999	-999	0.000
8	U	-999	-999	-999	-999	0.000	-999	-999	-999	-999	0.000
9	C	-999	-999	-999	-999	0.000	-999	-999	-999	-999	0.000
10	C	-999	-999	-999	-999	0.000	-999	-999	-999	-999	0.000
11	U	-999	-999	-999	-999	0.000	-999	-999	-999	-999	0.000
12	A	-999	-999	-999	-999	0.000	-999	-999	-999	-999	0.000
13	A	-999	-999	-999	-999	0.000	-999	-999	-999	-999	0.000
14	U	-999	-999	-999	-999	0.000	-999	-999	-999	-999	0.000
15	A	-999	-999	-999	-999	0.000	-999	-999	-999	-999	0.000
16	U	1.810	2.000	1.960	1.923	0.100	2.215	1.670	2.215	2.033	0.315
17	U	0.940	0.755	1.075	0.923	0.161	1.415	0.910	0.990	1.105	0.271
18	C	0.140	0.160	0.010	0.103	0.081	0.120	0.350	0.220	0.230	0.115
19	A	0.215	0.105	0.220	0.180	0.065	0.020	0.160	0.110	0.097	0.071
20	C	0.150	0.105	0.075	0.110	0.038	0.235	0.285	0.300	0.273	0.034
21	G	0.630	0.625	0.450	0.568	0.103	0.425	0.370	0.795	0.530	0.231
22	U	1.920	2.045	1.970	1.978	0.063	1.480	1.470	1.670	1.540	0.113
23	C	0.545	0.640	0.530	0.572	0.060	0.520	0.485	0.715	0.573	0.124
24	U	2.670	2.880	2.975	2.842	0.156	2.420	2.390	2.710	2.507	0.177
25	C	0.590	0.685	0.665	0.647	0.050	0.430	0.510	0.480	0.473	0.040
26	G	0.120	0.145	0.085	0.117	0.030	0.080	0.080	0.100	0.087	0.012
27	U	0.085	0.000	0.000	0.028	0.049	0.160	0.160	0.200	0.173	0.023
28	G	0.000	0.000	0.000	0.000	0.000	0.100	0.000	0.140	0.080	0.072
29	U	0.240	0.375	0.315	0.310	0.068	0.425	0.210	0.410	0.348	0.120
30	G	0.370	0.340	0.305	0.338	0.033	0.470	0.330	0.540	0.447	0.107
31	U	0.150	0.120	0.040	0.103	0.057	0.195	0.060	0.050	0.102	0.081
32	U	0.355	0.425	0.345	0.375	0.044	0.495	0.340	0.345	0.393	0.088
33	U	0.280	0.260	0.305	0.282	0.023	0.175	0.295	0.350	0.273	0.089
34	G	0.330	0.420	0.430	0.393	0.055	0.445	0.320	0.350	0.372	0.065
35	U	1.670	1.780	2.250	1.900	0.308	1.775	1.740	2.150	1.888	0.227
36	G	0.500	0.430	0.780	0.570	0.185	0.525	0.425	0.490	0.480	0.051
37	U	0.170	0.000	0.265	0.145	0.134	0.150	0.250	0.250	0.217	0.058
38	C	0.040	0.000	0.070	0.037	0.035	0.080	0.095	0.160	0.112	0.043
39	U	0.000	0.000	0.145	0.048	0.084	0.120	0.090	0.030	0.080	0.046
40	G	0.000	0.000	0.015	0.005	0.009	0.105	0.000	0.000	0.035	0.061
41	U	0.030	0.230	0.000	0.087	0.125	0.075	0.000	0.210	0.095	0.106
42	U	0.435	0.470	0.680	0.528	0.133	0.525	0.430	0.595	0.517	0.083
43	C	1.005	0.815	0.825	0.882	0.107	0.560	0.400	0.640	0.533	0.122
44	G	0.620	0.360	0.530	0.503	0.132	0.500	0.320	0.400	0.407	0.090
45	C	0.000	0.000	0.000	0.000	0.000	0.000	0.000	0.000	0.000	0.000
46	C	0.000	0.000	0.000	0.000	0.000	0.000	0.000	0.000	0.000	0.000
47	A	1.065	1.065	1.400	1.177	0.193	1.035	1.090	0.960	1.028	0.065
48	U	1.275	1.605	1.705	1.528	0.225	0.870	0.840	1.055	0.922	0.116

hSHAPE Reactivities from 3 independent experiments (SP109*i*)											
Nucleotides		In the Absence of Pr77[Gag]					In the Presence of Pr77[Gag]				
**Number**	**Sequence**	**Expt 1**	**Expt 2**	**Expt 3**	**Mean**	**SD**	**Expt 1**	**Expt 2**	**Expt 3**	**Mean**	**SD**
49	C	0.880	1.000	1.240	1.040	0.183	1.010	1.010	0.740	0.920	0.156
50	C → G	0.240	0.230	0.150	0.207	0.049	0.310	0.190	0.290	0.263	0.064
51	C	0.130	0.095	0.230	0.152	0.070	0.180	0.000	0.115	0.098	0.091
52	G → U	0.375	0.350	0.280	0.335	0.049	0.290	0.085	0.130	0.168	0.108
53	U	0.570	0.395	0.535	0.500	0.093	0.455	0.470	0.495	0.473	0.020
54	C	0.490	0.405	0.580	0.492	0.088	0.410	0.420	0.595	0.475	0.104
55	U	0.035	0.045	0.000	0.027	0.024	0.110	0.190	0.045	0.115	0.073
56	C	0.060	0.030	0.120	0.070	0.046	0.065	0.210	0.000	0.092	0.108
57	C	0.000	0.000	0.000	0.000	0.000	0.210	0.000	0.145	0.118	0.108
58	G	0.000	0.000	0.000	0.000	0.000	0.385	0.200	0.130	0.238	0.132
59	C	0.000	0.000	0.000	0.000	0.000	0.030	0.070	0.060	0.053	0.021
60	U	0.040	0.120	0.240	0.133	0.101	0.380	0.170	0.050	0.200	0.167
61	C	0.150	0.180	0.140	0.157	0.021	0.455	0.230	0.270	0.318	0.120
62	G	0.000	0.000	0.100	0.033	0.058	0.120	0.000	0.000	0.040	0.069
63	U	0.045	0.150	0.545	0.247	0.264	0.140	0.135	0.000	0.092	0.079
64	C	0.000	0.000	0.000	0.000	0.000	0.000	0.000	0.000	0.000	0.000
65	A	0.250	0.255	0.270	0.258	0.010	0.000	0.330	0.150	0.160	0.165
66	C	0.000	0.000	0.010	0.003	0.006	0.015	0.000	0.000	0.005	0.009
67	U	0.275	0.350	0.200	0.275	0.075	0.320	0.285	0.250	0.285	0.035
68	U	0.570	0.440	0.540	0.517	0.068	0.400	0.500	0.430	0.443	0.051
69	A	0.400	0.550	0.420	0.457	0.081	0.290	0.215	0.325	0.277	0.056
70	U	0.210	0.240	0.220	0.223	0.015	0.080	0.030	0.015	0.042	0.034
71	C	0.100	0.100	0.130	0.110	0.017	0.020	0.110	0.040	0.057	0.047
72	C	0.000	0.035	0.045	0.027	0.024	0.330	0.030	0.220	0.193	0.152
73	U	1.090	1.355	1.160	1.202	0.137	1.425	1.050	1.310	1.262	0.192
74	U	1.445	1.740	1.680	1.622	0.156	1.655	1.080	1.275	1.337	0.292
75	C	0.000	0.000	0.000	0.000	0.000	0.000	0.000	0.000	0.000	0.000
76	A	1.330	1.275	1.685	1.430	0.223	0.770	0.740	0.840	0.783	0.051
77	C	0.600	0.560	0.545	0.568	0.028	0.490	0.500	0.350	0.447	0.084
78	U	1.270	1.020	1.080	1.123	0.131	1.295	0.810	1.060	1.055	0.243
79	U	1.830	1.350	1.560	1.580	0.241	1.540	1.285	1.565	1.463	0.155
80	U	0.685	0.475	0.625	0.595	0.108	0.620	0.625	0.680	0.642	0.033
81	C	0.775	0.425	0.350	0.517	0.227	0.000	0.085	0.000	0.028	0.049
82	C	0.260	0.010	0.440	0.237	0.216	0.400	0.590	0.650	0.547	0.131
83	A	1.980	1.610	1.510	1.700	0.248	1.370	1.290	1.570	1.410	0.144
84	G	1.740	2.070	1.700	1.837	0.203	0.155	0.020	0.160	0.112	0.079
85	A	0.035	0.030	0.020	0.028	0.008	0.010	0.025	0.015	0.017	0.008
86	G	0.000	0.060	0.080	0.047	0.042	0.105	0.000	0.000	0.035	0.061
87	G	0.000	0.000	0.000	0.000	0.000	0.390	0.000	0.350	0.247	0.215
88	G	0.000	0.000	0.000	0.000	0.000	0.000	0.000	0.000	0.000	0.000
89	U	0.000	0.000	0.000	0.000	0.000	0.000	0.000	0.000	0.000	0.000
90	C	0.000	0.000	0.000	0.000	0.000	0.020	0.010	0.000	0.010	0.010
91	C	0.000	0.000	0.000	0.000	0.000	0.040	0.350	0.020	0.137	0.185
92	C	0.000	0.000	0.000	0.000	0.000	0.145	0.000	0.115	0.087	0.077
93	C	0.000	0.000	0.000	0.000	0.000	0.000	0.000	0.000	0.000	0.000
94	C	0.000	0.000	0.000	0.000	0.000	0.070	0.000	0.000	0.023	0.040
95	C	0.000	0.000	0.000	0.000	0.000	0.135	0.000	0.000	0.045	0.078
96	G	0.000	0.000	0.010	0.003	0.006	0.040	0.000	0.010	0.017	0.021

hSHAPE Reactivities from 3 independent experiments (SP109*i*)											
Nucleotides		In the Absence of Pr77^{Gag}					In the Presence of Pr77^{Gag}				
Number	Sequence	Expt 1	Expt 2	Expt 3	Mean	SD	Expt 1	Expt 2	Expt 3	Mean	SD
97	C	0.400	0.620	0.760	0.593	0.181	0.305	0.285	0.170	0.253	0.073
98	A	0.180	0.375	0.285	0.280	0.098	0.400	0.260	0.200	0.287	0.103
99	G	0.750	0.750	0.670	0.723	0.046	0.875	0.345	0.630	0.617	0.265
100	A	1.630	1.760	1.860	1.750	0.115	0.900	0.840	1.450	1.063	0.336
101	C	0.135	0.210	0.200	0.182	0.041	0.140	0.105	0.135	0.127	0.019
102	C	0.220	0.240	0.315	0.258	0.050	0.285	0.200	0.255	0.247	0.043
103	C	0.000	0.000	0.000	0.000	0.000	0.000	0.000	0.000	0.000	0.000
104	C	0.000	0.000	0.000	0.000	0.000	0.145	0.000	0.045	0.063	0.074
105	G	0.080	0.060	0.000	0.047	0.042	0.045	0.000	0.005	0.017	0.025
106	G	0.000	0.000	0.000	0.000	0.000	0.080	0.000	0.000	0.027	0.046
107	U	0.200	0.180	0.110	0.163	0.047	0.250	0.000	0.080	0.110	0.128
108	G	0.070	0.070	0.000	0.047	0.040	0.075	0.000	0.000	0.025	0.043
109	A	0.000	0.040	0.010	0.017	0.021	0.100	0.000	0.000	0.033	0.058
110	C	0.000	0.000	0.000	0.000	0.000	0.020	0.000	0.000	0.007	0.012
111	C	0.000	0.000	0.000	0.000	0.000	0.335	0.000	0.020	0.118	0.188
112	C	0.000	0.000	0.000	0.000	0.000	0.000	0.000	0.000	0.000	0.000
113	U	1.660	2.180	2.260	2.033	0.326	1.785	1.195	2.120	1.700	0.468
114	C	0.465	1.215	1.110	0.930	0.406	1.170	0.665	0.545	0.793	0.332
115	A	3.210	3.230	3.000	3.147	0.127	1.750	1.730	1.800	1.760	0.036
116	G	0.050	0.100	0.070	0.073	0.025	0.160	0.095	0.390	0.215	0.155
117	G	0.000	0.000	0.000	0.000	0.000	0.000	0.000	0.065	0.022	0.038
118	U	0.010	0.000	0.080	0.030	0.044	0.000	0.000	0.040	0.013	0.023
119	C	0.320	0.370	0.540	0.410	0.115	0.400	0.000	0.080	0.160	0.212
120	G	0.360	0.550	0.290	0.400	0.135	0.535	0.490	0.590	0.538	0.050
121	G	0.430	0.670	0.995	0.698	0.284	0.000	0.000	0.250	0.083	0.144
122	C	0.000	0.000	0.105	0.035	0.061	0.000	0.000	0.000	0.000	0.000
123	C	0.000	0.000	0.000	0.000	0.000	0.085	0.000	0.000	0.028	0.049
124	G	0.030	0.170	0.070	0.090	0.072	0.020	0.080	0.140	0.080	0.060
125	A	0.405	0.355	0.435	0.398	0.040	0.200	0.320	0.310	0.277	0.067
126	C	0.000	0.020	0.030	0.017	0.015	0.200	0.120	0.050	0.123	0.075
127	U	0.200	0.170	0.070	0.147	0.068	0.085	0.000	0.090	0.058	0.051
128	G	0.290	0.100	0.110	0.167	0.107	0.025	0.000	0.055	0.027	0.028
129	C	0.000	0.000	0.000	0.000	0.000	0.000	0.000	0.000	0.000	0.000
130	G	0.000	0.000	0.000	0.000	0.000	0.000	0.000	0.000	0.000	0.000
131	G	0.000	0.000	0.000	0.000	0.000	0.000	0.000	0.000	0.000	0.000
132	C	0.000	0.000	0.105	0.035	0.061	0.000	0.000	0.000	0.000	0.000
133	A	0.350	0.465	0.470	0.428	0.068	0.310	0.230	0.280	0.273	0.040
134	G	0.420	0.525	0.600	0.515	0.090	0.345	0.080	0.290	0.238	0.140
135	C	0.500	0.910	0.990	0.800	0.263	0.460	0.350	0.425	0.412	0.056
136	U	0.770	0.680	0.600	0.683	0.085	0.130	0.280	0.325	0.245	0.102
137	G	0.130	0.240	0.110	0.160	0.070	0.000	0.000	0.000	0.000	0.000
138	G	0.000	0.000	0.000	0.000	0.000	0.045	0.000	0.000	0.015	0.026
139	C	0.000	0.015	0.000	0.005	0.009	0.195	0.000	0.000	0.065	0.113
140	G	0.010	0.085	0.015	0.037	0.042	0.160	0.050	0.040	0.083	0.067
141	C	0.000	0.000	0.000	0.000	0.000	0.000	0.000	0.000	0.000	0.000
142	C	0.000	0.000	0.000	0.000	0.000	0.000	0.000	0.000	0.000	0.000
143	C	0.000	0.000	0.000	0.000	0.000	0.000	0.000	0.000	0.000	0.000
144	G	0.390	0.590	0.440	0.473	0.104	0.122	0.360	0.312	0.265	0.126

hSHAPE Reactivities from 3 independent experiments (SP109*i*)											
Nucleotides		In the Absence of Pr77^{Gag}					In the Presence of Pr77^{Gag}				
**Number**	**Sequence**	**Expt 1**	**Expt 2**	**Expt 3**	**Mean**	**SD**	**Expt 1**	**Expt 2**	**Expt 3**	**Mean**	**SD**
145	A	1.510	1.620	1.330	1.487	0.146	0.980	0.530	1.230	0.913	0.355
146	A	1.150	1.200	1.330	1.227	0.093	0.755	0.700	0.610	0.688	0.073
147	C	1.590	1.110	1.640	1.447	0.293	0.280	0.300	0.760	0.447	0.272
148	A	0.510	0.580	0.700	0.597	0.096	0.355	0.170	0.355	0.293	0.107
149	G	0.000	0.000	0.000	0.000	0.000	0.000	0.000	0.000	0.000	0.000
150	G	0.000	0.000	0.000	0.000	0.000	0.000	0.000	0.000	0.000	0.000
151	G	0.000	0.000	0.000	0.000	0.000	0.130	0.000	0.000	0.043	0.075
152	A	0.000	0.000	0.000	0.000	0.000	0.355	0.000	0.000	0.118	0.205
153	C	0.000	0.000	0.000	0.000	0.000	0.000	0.000	0.000	0.000	0.000
154	C	0.000	0.000	0.000	0.000	0.000	0.000	0.000	0.000	0.000	0.000
155	C	0.000	0.000	0.000	0.000	0.000	0.000	0.000	0.000	0.000	0.000
156	U	0.000	0.000	0.000	0.000	0.000	0.360	0.010	0.170	0.180	0.175
157	C	0.150	0.225	0.130	0.168	0.050	0.130	0.280	0.175	0.195	0.077
158	G	0.000	0.000	0.000	0.000	0.000	0.000	0.000	0.000	0.000	0.000
159	G	0.000	0.000	0.000	0.000	0.000	0.000	0.000	0.000	0.000	0.000
160	A	0.000	0.000	0.000	0.000	0.000	0.000	0.000	0.000	0.000	0.000
161	U	0.400	0.390	0.680	0.490	0.165	0.230	0.060	0.310	0.200	0.128
162	A	0.000	0.000	0.000	0.000	0.000	0.020	0.000	0.000	0.007	0.012
163	A	0.000	0.000	0.130	0.043	0.075	0.015	0.000	0.000	0.005	0.009
164	G	0.000	0.000	0.000	0.000	0.000	0.000	0.000	0.000	0.000	0.000
165	U	0.000	0.000	0.000	0.000	0.000	0.000	0.000	0.000	0.000	0.000
166	G	0.000	0.000	0.000	0.000	0.000	0.000	0.000	0.000	0.000	0.000
167	A	0.000	0.000	0.000	0.000	0.000	0.000	0.000	0.000	0.000	0.000
168	C	0.000	0.000	0.000	0.000	0.000	0.000	0.000	0.000	0.000	0.000
169	C	0.000	0.035	0.000	0.012	0.020	0.000	0.000	0.000	0.000	0.000
170	C	0.000	0.000	0.000	0.000	0.000	0.000	0.000	0.000	0.000	0.000
171	U	0.355	0.166	0.474	0.332	0.155	0.155	0.070	0.265	0.163	0.098
172	U	0.310	0.410	0.385	0.368	0.052	0.210	0.245	0.315	0.257	0.053
173	G	0.840	0.920	1.150	0.970	0.161	0.475	0.420	0.710	0.535	0.154
174	U	0.230	0.375	0.570	0.392	0.171	0.225	0.305	0.320	0.283	0.051
175	C	0.000	0.000	0.000	0.000	0.000	0.000	0.000	0.000	0.000	0.000
176	U	0.000	0.000	0.000	0.000	0.000	0.000	0.110	0.050	0.053	0.055
177	C	0.000	0.000	0.000	0.000	0.000	0.160	0.000	0.040	0.067	0.083
178	U	0.000	0.100	0.000	0.033	0.058	1.110	0.910	0.730	0.917	0.190
179	A	0.540	0.610	0.190	0.447	0.225	0.485	0.265	0.645	0.465	0.191
180	U	0.250	0.280	0.000	0.177	0.154	0.260	0.220	0.350	0.277	0.067
181	U	0.255	0.180	0.005	0.147	0.128	0.265	0.235	0.320	0.273	0.043
182	U	0.165	0.175	0.090	0.143	0.046	0.135	0.280	0.020	0.145	0.130
183	C	0.000	0.130	0.070	0.067	0.065	0.005	0.140	0.060	0.068	0.068
184	U	0.150	0.280	0.335	0.255	0.095	0.625	0.520	0.260	0.468	0.188
185	A	0.250	0.245	0.370	0.288	0.071	0.140	0.105	0.150	0.132	0.024
186	C	0.100	0.120	0.140	0.120	0.020	0.110	0.000	0.140	0.083	0.074
187	U	0.390	0.530	0.355	0.425	0.093	0.860	0.830	0.650	0.780	0.114
188	A	0.430	0.540	0.425	0.465	0.065	0.330	0.350	0.435	0.372	0.056
189	U	0.110	0.130	0.130	0.123	0.012	0.010	0.115	0.040	0.055	0.054
190	U	0.100	0.070	0.080	0.083	0.015	0.000	0.000	0.000	0.000	0.000
191	U	0.030	0.050	0.045	0.042	0.010	0.000	0.060	0.105	0.055	0.053
192	G	0.000	0.000	0.000	0.000	0.000	0.000	0.045	0.000	0.015	0.026

hSHAPE Reactivities from 3 independent experiments (SP109*i*)											
Nucleotides		In the Absence of Pr77^{Gag}					In the Presence of Pr77^{Gag}				
Number	Sequence	Expt 1	Expt 2	Expt 3	Mean	SD	Expt 1	Expt 2	Expt 3	Mean	SD
193	G	0.000	0.000	0.000	0.000	0.000	0.000	0.000	0.055	0.018	0.032
194	U	0.000	0.100	0.010	0.037	0.055	0.085	0.000	0.030	0.038	0.043
195	G	0.010	0.220	0.550	0.260	0.272	0.250	0.320	0.490	0.353	0.123
196	U	0.080	0.190	0.305	0.192	0.113	0.285	0.310	0.200	0.265	0.058
197	U	0.260	0.185	0.355	0.267	0.085	0.280	0.370	0.255	0.302	0.060
198	U	0.020	0.080	0.245	0.115	0.117	0.145	0.210	0.110	0.155	0.051
199	G	0.000	0.000	0.005	0.002	0.003	0.150	0.000	0.050	0.067	0.076
200	U	0.060	0.110	0.255	0.142	0.101	0.180	0.020	0.225	0.142	0.108
201	C	0.000	0.000	0.000	0.000	0.000	0.000	0.000	0.000	0.000	0.000
202	U	0.330	0.300	0.230	0.287	0.051	0.110	0.170	0.330	0.203	0.114
203	U	0.230	0.320	0.390	0.313	0.080	0.135	0.100	0.300	0.178	0.107
204	G	0.140	0.300	0.320	0.253	0.099	0.125	0.160	0.280	0.188	0.081
205	U	0.330	0.550	0.225	0.368	0.166	0.525	0.360	0.410	0.432	0.085
206	A	0.010	0.105	0.145	0.087	0.069	0.165	0.020	0.420	0.202	0.203
207	U	0.070	0.080	0.155	0.102	0.046	0.190	0.000	0.420	0.203	0.210
208	U	0.225	0.365	0.355	0.315	0.078	0.375	0.240	0.425	0.347	0.096
209	G	0.800	0.885	1.065	0.917	0.135	0.830	0.440	1.360	0.877	0.462
210	U	0.110	0.165	0.210	0.162	0.050	0.135	0.150	0.255	0.180	0.065
211	C	0.000	0.000	0.000	0.000	0.000	0.170	0.000	0.025	0.065	0.092
212	U	0.000	0.000	0.000	0.000	0.000	0.450	0.000	0.420	0.290	0.252
213	C	0.000	0.000	0.000	0.000	0.000	0.000	0.000	0.000	0.000	0.000
214	U	0.000	0.000	0.000	0.000	0.000	0.000	0.000	0.000	0.000	0.000
215	U	0.010	0.160	0.005	0.058	0.088	0.275	0.145	0.165	0.195	0.070
216	U	0.110	0.150	0.000	0.087	0.078	0.125	0.245	0.040	0.137	0.103
217	C	0.000	0.000	0.000	0.000	0.000	0.035	0.000	0.000	0.012	0.020
218	U	0.120	0.105	0.070	0.098	0.026	0.140	0.100	0.030	0.090	0.056
219	U	0.190	0.250	0.145	0.195	0.053	0.315	0.175	0.180	0.223	0.079
220	G	0.360	0.470	0.320	0.383	0.078	0.350	0.270	0.205	0.275	0.073
221	U	0.100	0.180	0.125	0.135	0.041	0.175	0.260	0.180	0.205	0.048
222	C	0.170	0.180	0.170	0.173	0.006	0.120	0.100	0.065	0.095	0.028
223	U	0.220	0.285	0.210	0.238	0.041	0.185	0.110	0.195	0.163	0.046
224	G	0.000	0.280	0.150	0.143	0.140	0.145	0.000	0.205	0.117	0.105
225	G	0.000	0.000	0.000	0.000	0.000	0.110	0.000	0.200	0.103	0.100
226	C	0.000	0.000	0.000	0.000	0.000	0.170	0.200	0.000	0.123	0.108
227	U	1.020	1.200	0.850	1.023	0.175	1.130	0.860	1.050	1.013	0.139
228	A	1.125	1.220	1.230	1.192	0.058	0.880	0.815	1.065	0.920	0.130
229	U	0.500	0.430	0.700	0.543	0.140	0.485	0.535	0.570	0.530	0.043
230	C	0.000	0.000	0.000	0.000	0.000	0.320	0.920	0.510	0.583	0.307
231	A	0.745	0.820	0.910	0.825	0.083	0.770	0.610	0.595	0.658	0.097
232	U	0.230	0.220	0.380	0.277	0.090	0.110	0.265	0.250	0.208	0.085
233	C	0.000	0.000	0.000	0.000	0.000	0.000	0.000	0.000	0.000	0.000
234	A	0.555	0.490	0.830	0.625	0.180	0.290	0.545	0.580	0.472	0.158
235	C	0.000	0.000	0.000	0.000	0.000	0.000	0.000	0.000	0.000	0.000
236	A	0.760	0.740	0.855	0.785	0.061	0.590	0.670	0.590	0.617	0.046
237	A	0.560	0.570	0.780	0.637	0.124	0.480	0.330	0.420	0.410	0.075
238	G	0.670	0.770	0.780	0.740	0.061	0.590	0.410	0.590	0.530	0.104
239	A	0.300	0.330	0.460	0.363	0.085	0.180	0.330	0.200	0.237	0.081
240	G	0.100	0.075	0.070	0.082	0.016	0.055	0.225	0.090	0.123	0.090

hSHAPE Reactivities from 3 independent experiments (SP109*i*)											
Nucleotides		In the Absence of $Pr77^{Gag}$					In the Presence of $Pr77^{Gag}$				
Number	Sequence	Expt 1	Expt 2	Expt 3	Mean	SD	Expt 1	Expt 2	Expt 3	Mean	SD
241	C	0.095	0.165	0.080	0.113	0.045	0.210	0.000	0.165	0.125	0.111
242	G	0.000	0.000	0.000	0.000	0.000	0.230	0.240	0.225	0.232	0.008
243	G	0.000	0.000	0.000	0.000	0.000	0.000	0.000	0.000	0.000	0.000
244	A	0.580	0.475	0.515	0.523	0.053	1.035	0.490	0.960	0.828	0.295
245	A	0.415	0.530	0.380	0.442	0.078	0.800	0.370	0.685	0.618	0.223
246	C → A	0.660	0.620	0.430	0.570	0.123	0.600	0.490	0.660	0.583	0.086
247	G	0.000	0.000	0.000	0.000	0.000	0.220	0.220	0.130	0.190	0.052
248	G → C	0.000	0.000	0.000	0.000	0.000	0.000	0.000	0.000	0.000	0.000
249	A	0.000	0.110	0.100	0.070	0.061	0.330	0.010	0.070	0.137	0.170
250	C	0.000	0.000	0.000	0.000	0.000	0.040	0.000	0.000	0.013	0.023
251	U	0.080	0.050	0.220	0.117	0.091	0.030	0.185	0.000	0.072	0.099
252	C	0.280	0.290	0.420	0.330	0.078	0.250	0.365	0.260	0.292	0.064
253	A	0.165	0.220	0.235	0.207	0.037	0.180	0.235	0.230	0.215	0.030
254	C	0.000	0.010	0.010	0.007	0.006	0.000	0.000	0.000	0.000	0.000
255	C	0.270	0.345	0.305	0.307	0.038	0.100	0.420	0.230	0.250	0.161
256	A	0.800	0.745	0.620	0.722	0.092	0.440	0.135	0.540	0.372	0.211
257	U	1.160	1.660	1.085	1.302	0.313	1.525	1.780	1.360	1.555	0.212
258	A	1.680	1.600	1.970	1.750	0.195	0.950	0.920	0.880	0.917	0.035
259	G	0.065	0.060	0.090	0.072	0.016	0.020	0.035	0.060	0.038	0.020
260	G	0.000	0.000	0.000	0.000	0.000	0.000	0.000	0.000	0.000	0.000
261	G	0.000	0.000	0.000	0.000	0.000	0.000	0.000	0.000	0.000	0.000
262	A	0.135	0.135	0.135	0.135	0.000	0.120	0.180	0.220	0.173	0.050
263	G	0.200	0.150	0.210	0.187	0.032	0.195	0.130	0.010	0.112	0.094
264	C	0.210	0.320	0.305	0.278	0.060	0.280	0.200	0.155	0.212	0.063
265	U	0.310	0.380	0.375	0.355	0.039	0.140	0.150	0.115	0.135	0.018
266	G	0.170	0.320	0.180	0.223	0.084	0.120	0.050	0.000	0.057	0.060
267	C	0.000	0.000	0.000	0.000	0.000	0.000	0.000	0.000	0.000	0.000
268	A	0.140	0.660	0.360	0.387	0.261	0.390	0.270	0.220	0.293	0.087
269	G	0.000	0.000	0.000	0.000	0.000	0.325	0.640	0.475	0.480	0.158
270	U	0.000	0.130	0.105	0.078	0.069	0.295	0.195	0.150	0.213	0.074
271	C	0.160	0.230	0.330	0.240	0.085	0.000	0.000	0.060	0.020	0.035
272	C	0.000	0.000	0.000	0.000	0.000	0.000	0.000	0.085	0.028	0.049
273	C	0.000	0.000	0.000	0.000	0.000	0.000	0.000	0.310	0.103	0.179
274	G	0.000	0.000	0.000	0.000	0.000	0.000	0.000	0.000	0.000	0.000
275	C	0.000	0.000	0.000	0.000	0.000	0.000	0.000	0.000	0.000	0.000
276	C	0.000	0.000	0.000	0.000	0.000	0.000	0.060	0.010	0.023	0.032
277	U	0.050	0.110	0.055	0.072	0.033	0.000	0.110	0.030	0.047	0.057
278	A	0.065	0.035	0.090	0.063	0.028	0.040	0.050	0.055	0.048	0.008
279	C	0.185	0.150	0.135	0.157	0.026	0.200	0.170	0.155	0.175	0.023
280	G	0.310	0.330	0.400	0.347	0.047	0.145	0.065	0.180	0.130	0.059
281	G	1.920	1.900	1.990	1.937	0.047	1.290	0.960	1.335	1.195	0.205
282	A	2.930	2.960	3.110	3.000	0.096	1.890	1.660	1.945	1.832	0.151
283	G	1.090	1.090	1.230	1.137	0.081	0.560	0.480	0.640	0.560	0.080
284	A	2.730	2.730	2.630	2.697	0.058	1.585	1.720	1.625	1.643	0.069
285	A	1.820	1.810	1.940	1.857	0.072	1.145	1.180	1.240	1.188	0.048
286	G	1.560	1.540	1.540	1.547	0.012	0.950	0.820	1.020	0.930	0.101
287	A	3.090	3.010	2.820	2.973	0.139	2.040	1.815	1.940	1.932	0.113
288	G	1.371	1.754	1.735	1.620	0.216	0.720	0.444	1.123	0.762	0.341

hSHAPE Reactivities from 3 independent experiments (SP109*i*)											
Nucleotides		In the Absence of $Pr77^{Gag}$					In the Presence of $Pr77^{Gag}$				
Number	Sequence	Expt 1	Expt 2	Expt 3	Mean	SD	Expt 1	Expt 2	Expt 3	Mean	SD
289	G	0.000	0.100	0.250	0.117	0.126	0.290	0.250	0.355	0.298	0.053
290	U	0.000	0.000	0.000	0.000	0.000	0.040	0.000	0.020	0.020	0.020
291	A	0.030	0.050	0.100	0.060	0.036	0.000	0.000	0.150	0.050	0.087
292	G	0.000	0.000	0.000	0.000	0.000	0.000	0.000	0.000	0.000	0.000
293	G	0.000	0.000	0.000	0.000	0.000	0.280	0.000	0.000	0.093	0.162
294	U	0.690	0.680	0.590	0.653	0.055	0.800	0.540	0.490	0.610	0.166
295	U	0.850	1.100	1.220	1.057	0.189	0.940	0.300	0.910	0.717	0.361
296	A	1.830	1.680	1.790	1.767	0.078	0.990	0.830	1.390	1.070	0.288
297	C	1.370	1.190	1.450	1.337	0.133	0.960	0.570	1.110	0.880	0.279
298	G	0.700	0.180	0.670	0.517	0.292	0.020	0.000	0.050	0.023	0.025
299	G	0.415	0.290	0.130	0.278	0.143	0.130	0.000	0.130	0.087	0.075
300	U	0.020	0.070	0.090	0.060	0.036	0.080	0.000	0.110	0.063	0.057
301	G	0.000	0.000	0.000	0.000	0.000	0.110	0.120	0.180	0.137	0.038
302	A	0.000	0.000	0.000	0.000	0.000	0.000	0.000	0.000	0.000	0.000
303	G	0.000	0.000	0.000	0.000	0.000	0.150	0.000	0.080	0.077	0.075
304	C	0.000	0.000	0.000	0.000	0.000	0.090	0.020	0.090	0.067	0.040
305	C	0.000	0.000	0.000	0.000	0.000	0.650	0.700	0.510	0.620	0.098
306	A	1.280	1.280	1.190	1.250	0.052	0.850	0.910	1.220	0.993	0.199
307	U	1.510	1.420	1.460	1.463	0.045	1.060	1.340	1.300	1.233	0.151
308	U	1.760	1.680	1.780	1.740	0.053	1.240	1.360	1.590	1.397	0.178
309	G	0.700	0.670	0.680	0.683	0.015	0.530	0.420	0.500	0.483	0.057
310	G	0.610	0.610	0.620	0.613	0.006	0.510	0.290	0.460	0.420	0.115
311	A	0.540	0.520	0.490	0.517	0.025	0.400	0.250	0.460	0.370	0.108
312	A	0.360	0.370	0.440	0.390	0.044	0.240	0.140	0.360	0.247	0.110
313	A	0.420	0.410	0.490	0.440	0.044	0.250	0.180	0.380	0.270	0.101
314	U	0.430	0.430	0.610	0.490	0.104	0.400	0.380	0.460	0.413	0.042
315	G	0.000	0.000	0.030	0.010	0.017	0.000	0.050	0.040	0.030	0.026
316	G	0.000	0.000	0.000	0.000	0.000	0.030	0.000	0.000	0.010	0.017
317	G	0.000	0.000	0.000	0.000	0.000	0.000	0.000	0.000	0.000	0.000
318	G	0.000	0.000	0.000	0.000	0.000	0.000	0.000	0.230	0.077	0.133
319	G	0.000	0.000	0.000	0.000	0.000	1.310	0.000	1.440	0.917	0.797
320	U	0.260	0.300	0.510	0.357	0.134	0.510	0.450	0.500	0.487	0.032
321	C	0.000	0.000	0.010	0.003	0.006	0.070	0.000	0.000	0.023	0.040
322	U	0.150	0.170	0.260	0.193	0.059	0.120	0.460	0.100	0.227	0.202
323	C	0.020	0.070	0.110	0.067	0.045	0.080	0.400	0.020	0.167	0.204
324	G	0.100	0.210	0.170	0.160	0.056	0.170	0.630	0.140	0.313	0.275
325	G	0.000	0.000	0.000	0.000	0.000	0.280	0.000	0.030	0.103	0.154
326	G	0.000	0.000	0.000	0.000	0.000	0.000	0.000	0.000	0.000	0.000
327	C	0.000	0.000	0.000	0.000	0.000	0.000	0.000	0.000	0.000	0.000
328	U	0.000	0.290	0.050	0.113	0.155	0.520	0.690	0.440	0.550	0.128
329	C	0.000	0.000	0.000	0.000	0.000	0.100	0.300	0.550	0.317	0.225
330	A	0.850	0.890	0.890	0.877	0.023	0.750	0.660	0.840	0.750	0.090
331	A	1.310	1.240	1.430	1.327	0.096	0.890	0.810	1.170	0.957	0.189
332	A	1.110	1.120	1.140	1.123	0.015	0.690	0.710	0.970	0.790	0.156
333	A	0.850	0.830	0.910	0.863	0.042	0.470	0.560	0.760	0.597	0.148
334	G	0.160	0.160	0.230	0.183	0.040	0.050	0.000	0.190	0.080	0.098
335	G	0.000	0.000	0.110	0.037	0.064	0.030	0.000	0.040	0.023	0.021
336	G	0.110	0.170	0.200	0.160	0.046	0.200	0.170	0.150	0.173	0.025

hSHAPE Reactivities from 3 independent experiments (SP109*i*)											
**Nucleotides**		**In the Absence of Pr77^{Gag}**					**In the Presence of Pr77^{Gag}**				
**Number**	**Sequence**	**Expt 1**	**Expt 2**	**Expt 3**	**Mean**	**SD**	**Expt 1**	**Expt 2**	**Expt 3**	**Mean**	**SD**
337	C	0.350	0.490	0.420	0.420	0.070	0.340	0.000	0.430	0.257	0.227
338	A	0.090	0.210	0.130	0.143	0.061	0.120	0.210	0.230	0.187	0.059
339	G	0.000	0.110	0.130	0.080	0.070	0.110	0.220	0.210	0.180	0.061
340	A	0.110	0.260	0.130	0.167	0.081	0.100	0.180	0.220	0.167	0.061
341	A	0.340	0.610	0.450	0.467	0.136	0.240	0.350	0.510	0.367	0.136
342	A	0.000	0.220	0.080	0.100	0.111	0.110	0.000	0.100	0.070	0.061
343	C	0.000	0.100	0.000	0.033	0.058	0.210	0.000	0.040	0.083	0.112
344	U	1.440	1.510	1.760	1.570	0.168	1.230	1.070	1.450	1.250	0.191
345	C	0.490	0.390	0.980	0.620	0.316	0.710	0.200	0.960	0.623	0.387
346	U	0.760	0.540	0.760	0.687	0.127	0.510	0.290	0.720	0.507	0.215
347	U	1.230	1.090	1.240	1.187	0.084	0.850	0.610	1.220	0.893	0.307
348	U	1.180	1.050	1.200	1.143	0.081	0.740	0.570	1.140	0.817	0.293
349	G	0.000	0.000	0.020	0.007	0.012	0.090	0.000	0.070	0.053	0.047
350	U	0.320	0.360	0.270	0.317	0.045	0.290	0.000	0.250	0.180	0.157
351	U	0.400	0.400	0.460	0.420	0.035	0.130	0.090	0.180	0.133	0.045
352	U	0.000	0.000	0.000	0.000	0.000	0.010	0.000	0.000	0.003	0.006
353	C	0.000	0.000	0.000	0.000	0.000	0.270	0.000	0.010	0.093	0.153
354	U	0.980	0.950	0.890	0.940	0.046	1.080	0.250	1.140	0.823	0.497
355	G	3.660	3.420	3.850	3.643	0.215	2.310	1.800	3.200	2.437	0.709
356	U	0.290	0.360	0.180	0.277	0.091	0.540	0.080	0.290	0.303	0.230
357	U	0.410	0.460	0.280	0.383	0.093	0.510	0.190	0.330	0.343	0.160
358	U	0.420	0.440	0.270	0.377	0.093	0.360	0.300	0.360	0.340	0.035
359	U	0.370	0.440	0.160	0.323	0.146	0.440	0.240	0.450	0.377	0.118
360	A	0.430	0.480	0.510	0.473	0.040	0.380	0.310	0.510	0.400	0.101
361	C	0.000	0.000	0.000	0.000	0.000	0.180	0.250	0.280	0.237	0.051
362	A	0.110	0.240	0.330	0.227	0.111	0.220	0.170	0.200	0.197	0.025
363	A	0.000	0.170	0.140	0.103	0.091	0.110	0.000	0.040	0.050	0.056
364	A	0.000	0.100	0.040	0.047	0.050	0.050	0.000	0.000	0.017	0.029
365	G	0.000	0.000	0.000	0.000	0.000	0.000	0.000	0.000	0.000	0.000
366	G	0.000	0.000	0.000	0.000	0.000	0.000	0.000	0.000	0.000	0.000
367	C	0.000	0.000	0.000	0.000	0.000	0.070	0.000	0.000	0.023	0.040
368	U	0.000	0.000	0.000	0.000	0.000	0.090	0.000	0.000	0.030	0.052
369	C	0.000	0.000	0.000	0.000	0.000	0.110	0.000	0.000	0.037	0.064
370	C	0.000	0.000	0.000	0.000	0.000	0.330	0.000	0.020	0.117	0.185
371	U	0.000	0.000	0.000	0.000	0.000	0.190	0.000	0.000	0.063	0.110
372	C	0.070	0.000	0.030	0.033	0.035	0.300	0.000	0.000	0.100	0.173
373	U	0.660	0.600	0.360	0.540	0.159	0.540	0.550	0.580	0.557	0.021
374	C	3.110	2.940	1.630	2.560	0.810	2.640	2.780	3.000	2.807	0.181
375	A	3.630	3.360	4.680	3.890	0.697	3.120	2.330	4.160	3.203	0.918
376	G	3.100	2.620	3.330	3.017	0.362	3.170	1.830	3.280	2.760	0.807
377	A	3.770	3.500	4.390	3.887	0.456	4.110	1.900	3.500	3.170	1.141
378	G	0.000	0.000	0.000	0.000	0.000	0.000	0.000	0.040	0.013	0.023
379	A	0.000	0.000	0.000	0.000	0.000	0.050	0.160	0.000	0.070	0.082
380	G	0.000	0.000	0.160	0.053	0.092	0.000	0.400	0.000	0.133	0.231
381	G	0.000	0.000	0.000	0.000	0.000	0.000	0.000	0.000	0.000	0.000
382	G	0.000	0.000	6.160	2.053	3.556	0.000	0.000	0.050	0.017	0.029
383	G	0.120	0.000	0.000	0.040	0.069	0.000	0.000	0.000	0.000	0.000
384	U	0.000	0.000	0.000	0.000	0.000	0.000	0.000	0.000	0.000	0.000

hSHAPE Reactivities from 3 independent experiments (SP109*i*)											
**Nucleotides**		**In the Absence of Pr77^{Gag}**					**In the Presence of Pr77^{Gag}**				
**Number**	**Sequence**	**Expt 1**	**Expt 2**	**Expt 3**	**Mean**	**SD**	**Expt 1**	**Expt 2**	**Expt 3**	**Mean**	**SD**
385	C	0.040	0.060	0.000	0.033	0.031	0.400	0.500	0.670	0.523	0.137
386	U	0.590	0.410	0.110	0.370	0.242	0.600	0.320	0.500	0.473	0.142
387	U	0.000	0.000	0.650	0.217	0.375	1.500	1.710	0.630	1.280	0.573
388	C	1.520	1.310	1.110	1.313	0.205	1.180	4.610	1.130	2.307	1.995
389	A	0.050	0.010	1.400	0.487	0.791	0.600	0.840	0.970	0.803	0.188
390	U	0.000	0.000	0.130	0.043	0.075	0.000	0.000	0.030	0.010	0.017
391	G	0.010	0.000	0.040	0.017	0.021	0.060	0.330	0.000	0.130	0.176
392	U	0.020	0.000	0.000	0.007	0.012	0.120	0.120	0.000	0.080	0.069
393	G	0.060	0.000	0.000	0.020	0.035	0.120	0.140	0.020	0.093	0.064
394	A	0.150	0.110	0.000	0.087	0.078	0.100	0.140	0.050	0.097	0.045
395	A	0.550	0.400	0.380	0.443	0.093	0.180	0.570	0.190	0.313	0.222
396	A	0.950	0.830	0.370	0.717	0.306	0.330	0.680	0.460	0.490	0.177
397	G	0.200	0.170	0.830	0.400	0.373	0.000	0.000	0.870	0.290	0.502
398	A	0.350	0.190	0.120	0.220	0.118	0.190	0.250	0.100	0.180	0.075
399	G	0.390	0.230	0.100	0.240	0.145	0.000	0.130	0.200	0.110	0.101
400	A	0.360	0.140	0.290	0.263	0.112	0.670	0.000	0.170	0.280	0.348
401	G	1.010	0.710	0.450	0.723	0.280	0.390	0.100	0.180	0.223	0.150
402	U	0.990	0.560	0.250	0.600	0.372	0.200	0.070	0.690	0.320	0.327
403	A	0.410	0.260	0.120	0.263	0.145	0.450	1.320	0.450	0.740	0.502
404	G	0.510	0.050	0.030	0.197	0.272	0.250	0.940	0.570	0.587	0.345
405	U	0.000	0.000	0.020	0.007	0.012	0.000	0.000	0.380	0.127	0.219
406	G	0.000	0.000	0.150	0.050	0.087	0.040	0.590	0.030	0.220	0.320
407	C	0.040	0.220	0.000	0.087	0.117	0.000	0.000	0.000	0.000	0.000
408	A	0.420	0.540	0.400	0.453	0.076	1.050	0.470	0.580	0.700	0.308
409	A	0.490	0.490	0.390	0.457	0.058	0.140	0.000	0.150	0.097	0.084
410	U	0.310	0.640	0.420	0.457	0.168	0.610	0.180	0.370	0.387	0.215
411	A	0.380	0.250	0.400	0.343	0.081	0.330	0.710	0.510	0.517	0.190
412	G	0.440	0.500	0.220	0.387	0.147	0.200	0.330	0.630	0.387	0.221
413	A	0.570	0.510	0.320	0.467	0.131	0.240	0.420	0.510	0.390	0.137
414	A	0.640	0.550	0.230	0.473	0.215	0.230	0.430	0.390	0.350	0.106
415	U	0.340	0.220	0.350	0.303	0.072	0.360	0.420	0.420	0.400	0.035
416	U	0.720	0.740	0.370	0.610	0.208	0.560	0.390	0.420	0.457	0.091
417	U	0.700	0.650	0.490	0.613	0.110	0.000	0.250	0.320	0.190	0.168
418	U	1.020	0.500	0.650	0.723	0.268	0.270	0.910	0.140	0.440	0.412
419	A	0.320	0.200	1.070	0.530	0.471	0.470	0.800	0.650	0.640	0.165
420	U	0.740	0.620	0.550	0.637	0.096	0.110	0.900	0.720	0.577	0.414
421	C	0.730	0.810	0.850	0.797	0.061	0.300	0.850	0.580	0.577	0.275
422	A	0.420	0.400	0.210	0.343	0.116	0.050	0.670	0.390	0.370	0.310
423	G	0.280	0.280	0.350	0.303	0.040	1.020	0.300	0.790	0.703	0.368
424	U	0.250	0.040	0.520	0.270	0.241	0.610	0.460	0.820	0.630	0.181
425	U	0.240	0.410	0.340	0.330	0.085	0.080	0.210	0.370	0.220	0.145
426	U	0.150	0.260	0.150	0.187	0.064	0.150	0.250	0.270	0.223	0.064
427	C	-999	-999	-999	-999	0.000	-999	-999	-999	-999	0.000
428	U	-999	-999	-999	-999	0.000	-999	-999	-999	-999	0.000
429	A	-999	-999	-999	-999	0.000	-999	-999	-999	-999	0.000
430	A	-999	-999	-999	-999	0.000	-999	-999	-999	-999	0.000
431	U	-999	-999	-999	-999	0.000	-999	-999	-999	-999	0.000
432	A	-999	-999	-999	-999	0.000	-999	-999	-999	-999	0.000
