# Supplementary material for: MMTV RNA packaging requires an extended long-range interaction for productive Gag binding to packaging signals
Source: PLoS Biol. 2024 Oct 3;22(10):e3002827. doi: 10.1371/journal.pbio.3002827 (PMC11449360; doi:10.1371/journal.pbio.3002827)
Supplement: S1 Raw Images — (PDF) [file pbio.3002827.s015.pdf]

Uncropped Gel Used to Make Panel I of Figure 2B

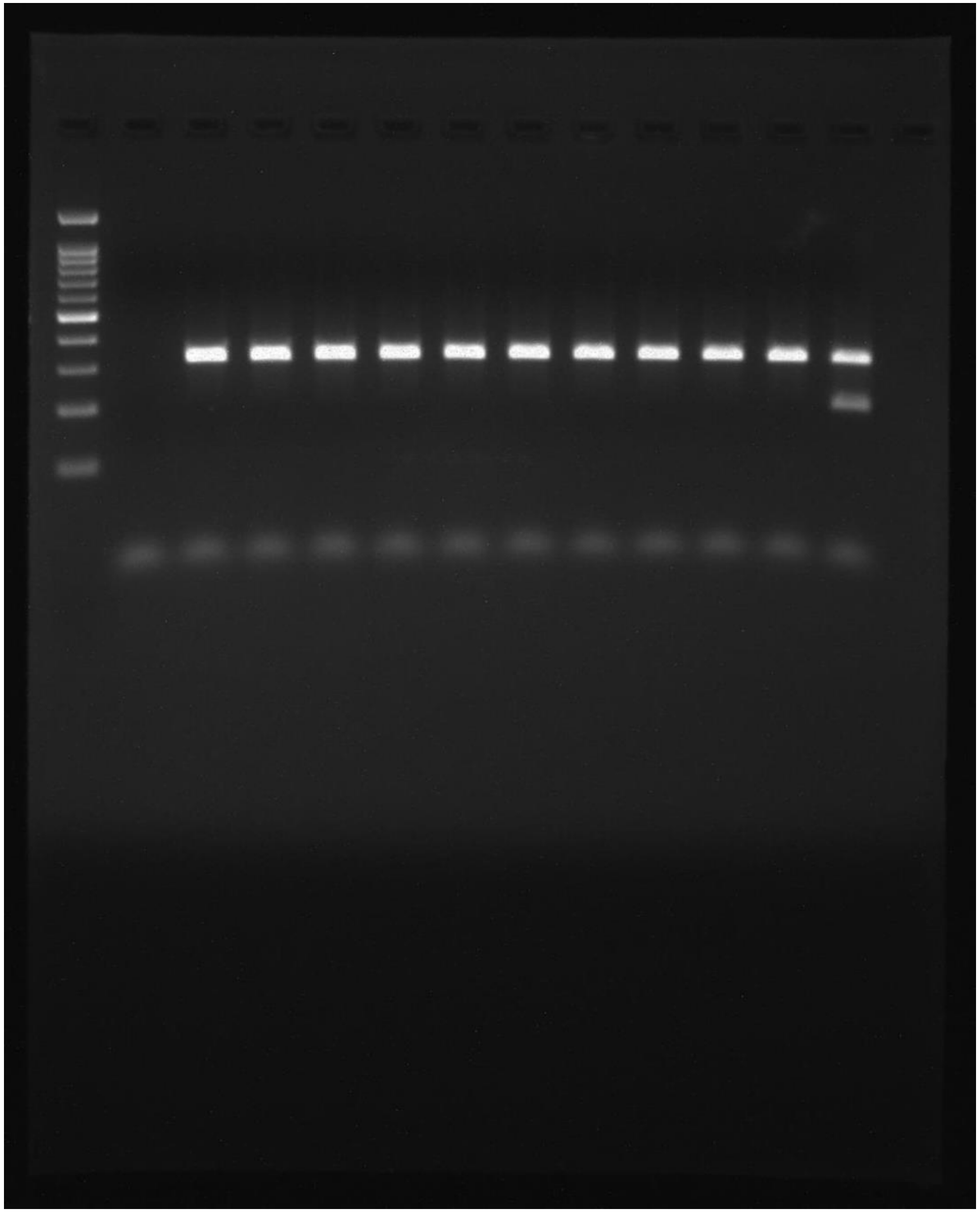

Uncropped Gel Used to Make Panel II & III of Figure 2B

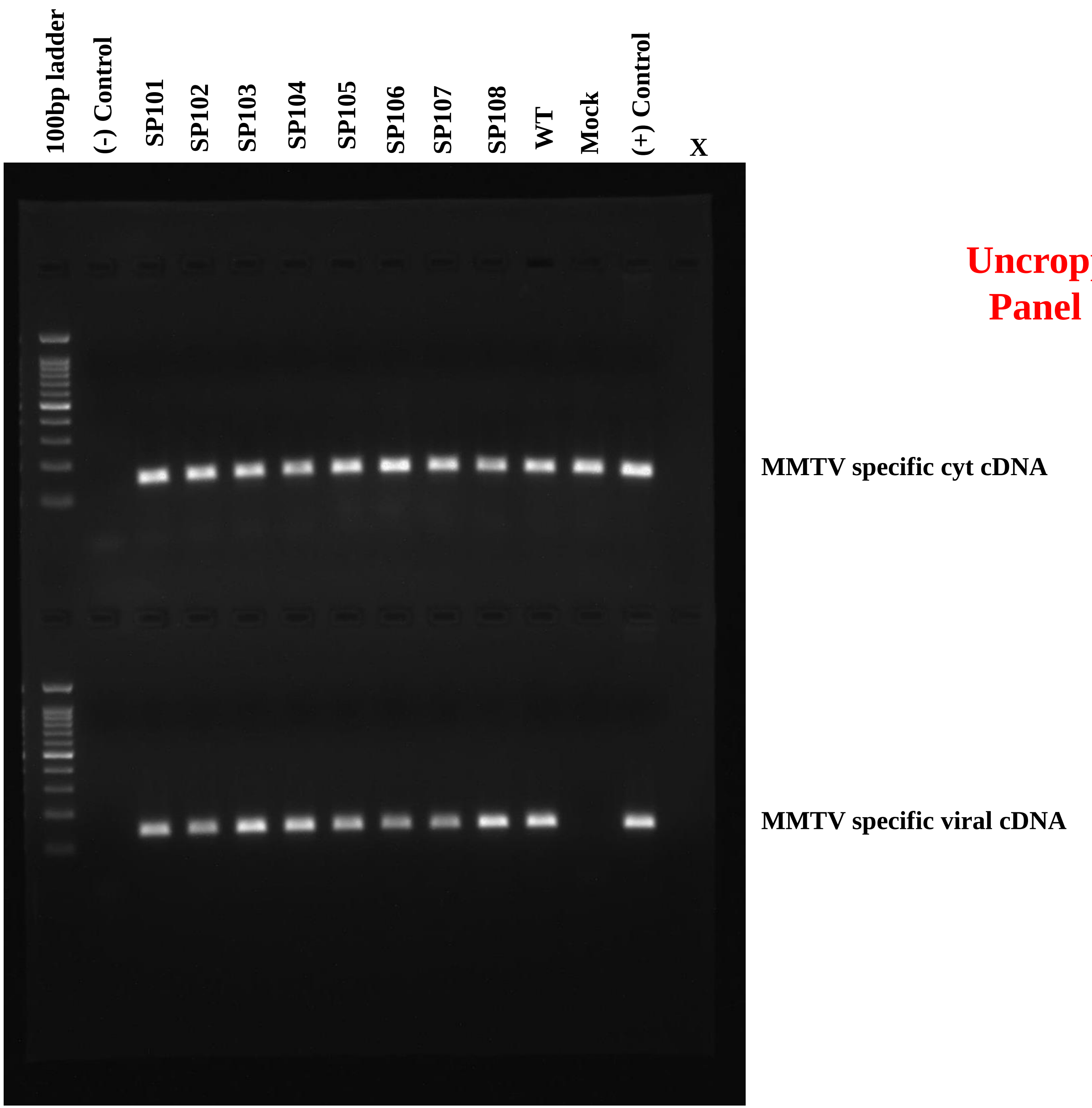

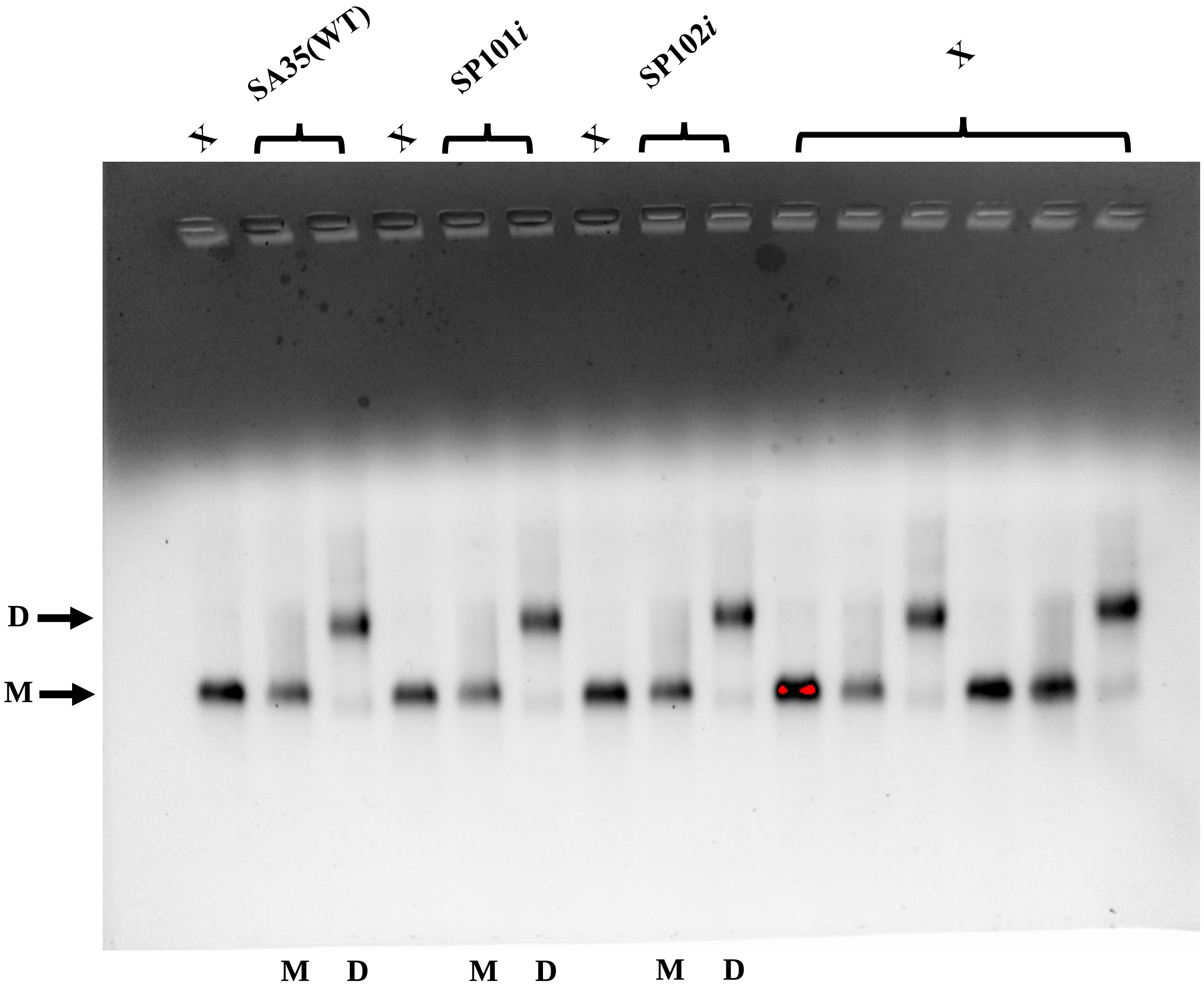

Uncropped Gel Used to Make Figure 3C

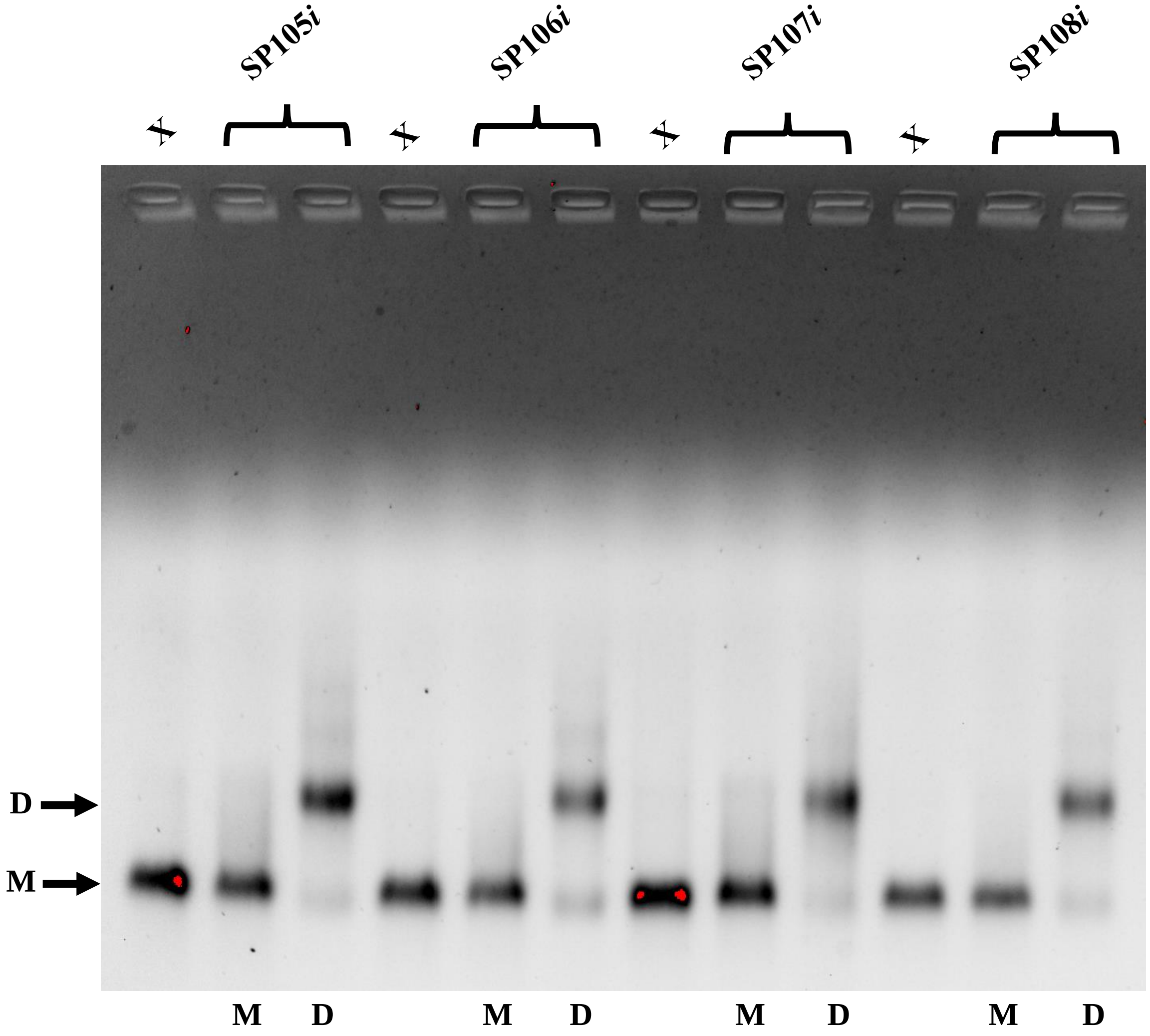

Uncropped Gel Used to Make Figure 3C

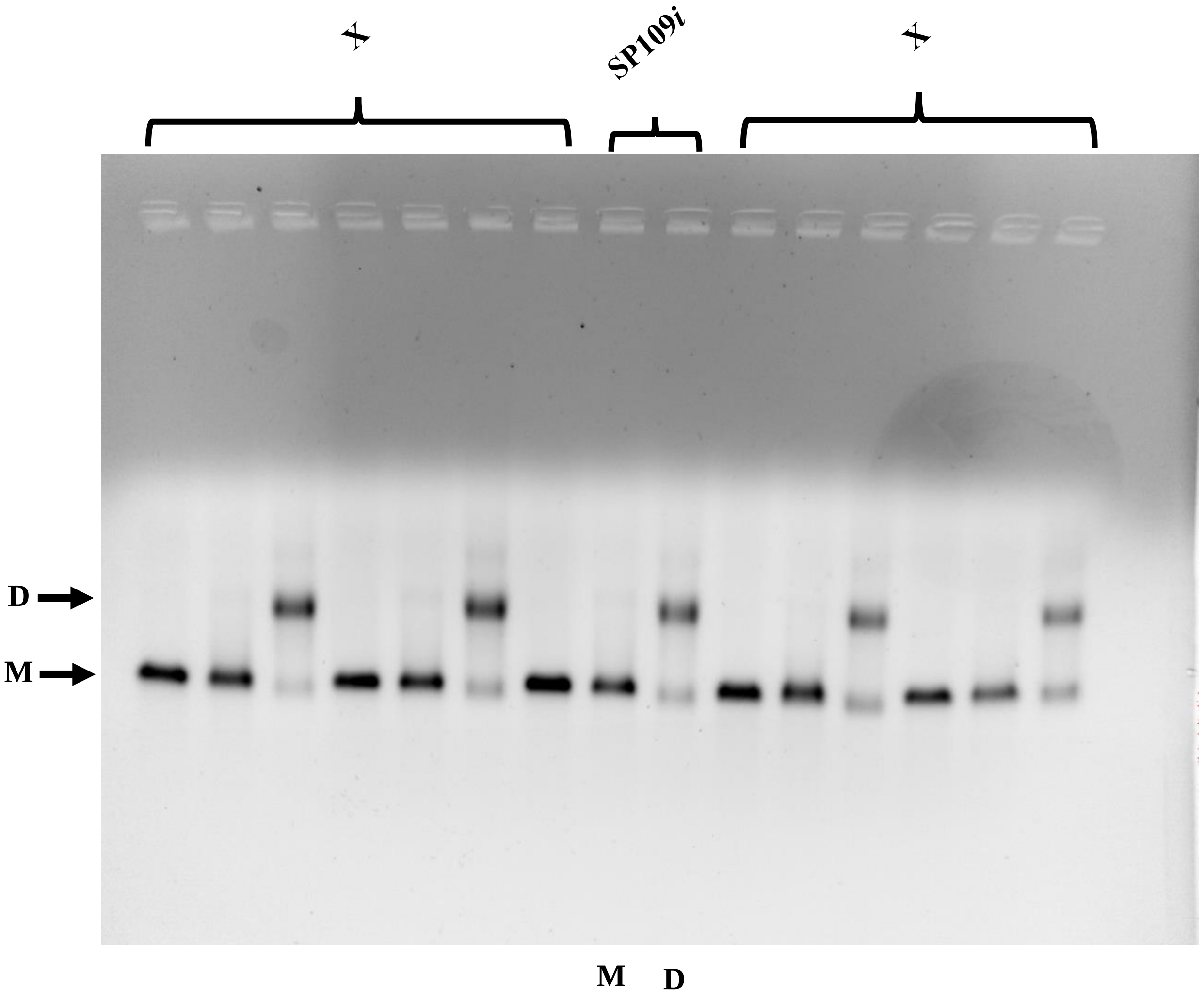

Uncropped Gel Used to Make Figure 3C

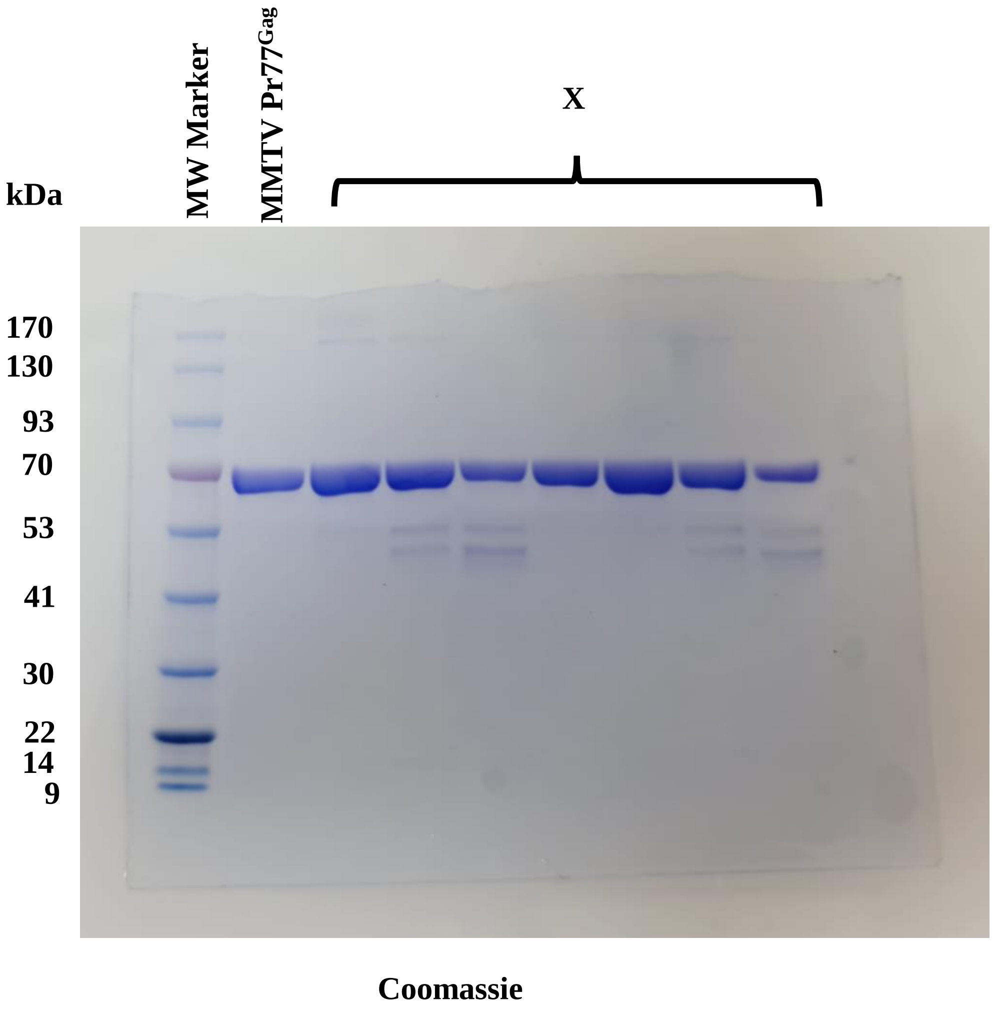

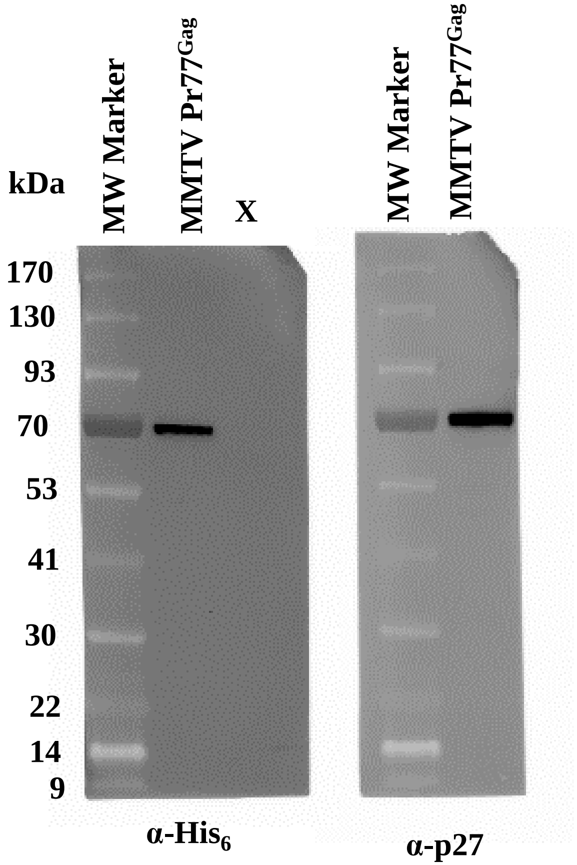

Uncropped Gel Used to Make Supplementary Figure 3A

Uncropped Western Blots Used to Make Supplementary Figure 3B
